# Mitochondria‐Targeting Moieties Based on N‐Tethered Pyridinium Cations

**DOI:** 10.1002/anie.7158257

**Published:** 2026-05-20

**Authors:** Ivan Džajić, Natalija Trunkelj, Jernej Repas, Maša Kandušer, Lara Smrdel, Stane Pajk, Lovro Žiberna, Irena Mlinarič‐Raščan, Bostjan Markelc, Tim Bozic, Masa Omerzel, Tanja Jesenko, Maja Cemazar, Katja Kološa, Bojana Žegura, Miha Virant, Matic Lozinšek, Hai M. Nguyen, Joshua A. Nasburg, Heike Wulff, Maxime Gueguinou, Valerije Vrček, Veronica Carpanese, Ildiko Szabo, Luis A. Pardo, Tihomir Tomašič, Lucija Peterlin Mašič, Andrej Emanuel Cotman

**Affiliations:** ^1^ Faculty of Pharmacy University of Ljubljana Ljubljana Slovenia; ^2^ Department of Experimental Oncology Institute of Oncology Ljubljana Ljubljana Slovenia; ^3^ Biotechnical Faculty, University of Ljubljana Ljubljana Slovenia; ^4^ Faculty of Health Sciences University of Ljubljana Ljubljana Slovenia; ^5^ Faculty of Medicine University of Ljubljana Ljubljana Slovenia; ^6^ Faculty of Health Sciences University of Primorska Izola Slovenia; ^7^ Department of Genetic Toxicology and Cancer Biology National Institute of Biology Ljubljana Slovenia; ^8^ Jožef Stefan Institute Ljubljana Slovenia; ^9^ Department of Pharmacology School of Medicine University of California Davis California USA; ^10^ Inserm UMR1069 “Niche Nutrition Cancer and Oxydative Metabolism (N2COx)” University of Tours Tours France; ^11^ Faculty of Pharmacy and Biochemistry University of Zagreb Zagreb Croatia; ^12^ Department of Biology University of Padova Padova Italy; ^13^ City Campus Max Planck Institute for Multidisciplinary Sciences Göttingen Germany

**Keywords:** cancer, cations, fluorescent probes, medicinal chemistry, mitochondria

## Abstract

Mitochondria‐targeting moieties (MTMs) are molecular fragments designed to deliver covalently tethered functional cargo to mitochondria, providing a modular strategy for chemical biology tools, imaging agents, and mitochondria‐targeted therapies. Phosphonium‐ or nitrogen cation‐based MTMs are not inert vectors and exhibit intrinsic bioactivity on mitochondrial and cellular levels to various extents. Here, we systematically evaluated a panel of N^+^‐based cations to determine how structural features influence subcellular distribution and inherent bioactivity. Live‐cell imaging of fluorescent dye conjugates revealed that 3,5‐diphenylpyridinium (DPPy^+^) exhibits cellular uptake and mitochondrial targeting comparable to the benchmark triphenylphosphonium (TPP^+^), whereas conjugates with unsubstituted pyridinium preferentially accumulate in lysosomes. Profiling of inert cargo derivatives showed that DPPy^+^ has lower intrinsic activity on mitochondrial membrane potential and oxidative phosphorylation, as well as on cellular respiration and viability than TPP^+^. The combination of efficient mitochondrial delivery and low intrinsic bioactivity translated to bioactive cargo: a Kv1.3 inhibitor conjugate with DPPy^+^ induced apoptosis in cancer cell lines and demonstrated improved cancer selectivity relative to the TPP^+^ conjugate in pancreatic organoid models. These results position lipophilic pyridinium cations as effective TPP^+^ surrogates with enhanced biocompatibility for mitochondria‐targeted therapeutic and diagnostic agents, while revealing the structure‐dependent competing lysosomal accumulation of permanent nitrogen cations.

## Graphical abstract

Pyridinium cations were benchmarked as mitochondria‐targeting moieties in a panel of N‐tethered fluorescent‐, bioactive‐, and inert‐cargo conjugates. 3,5‐Diphenylpyridinium (DPPy^+^) is a competent triphenylphosphonium (TPP^+^) surrogate, combining high mitochondria‐targeting efficiency with lower intrinsic effects on mitochondrial function. The unsubstituted pyridinium (Py^+^) conjugates show low cellular uptake and mostly lysosomal accumulation.

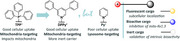

## 1 Introduction

**FIGURE 1:**
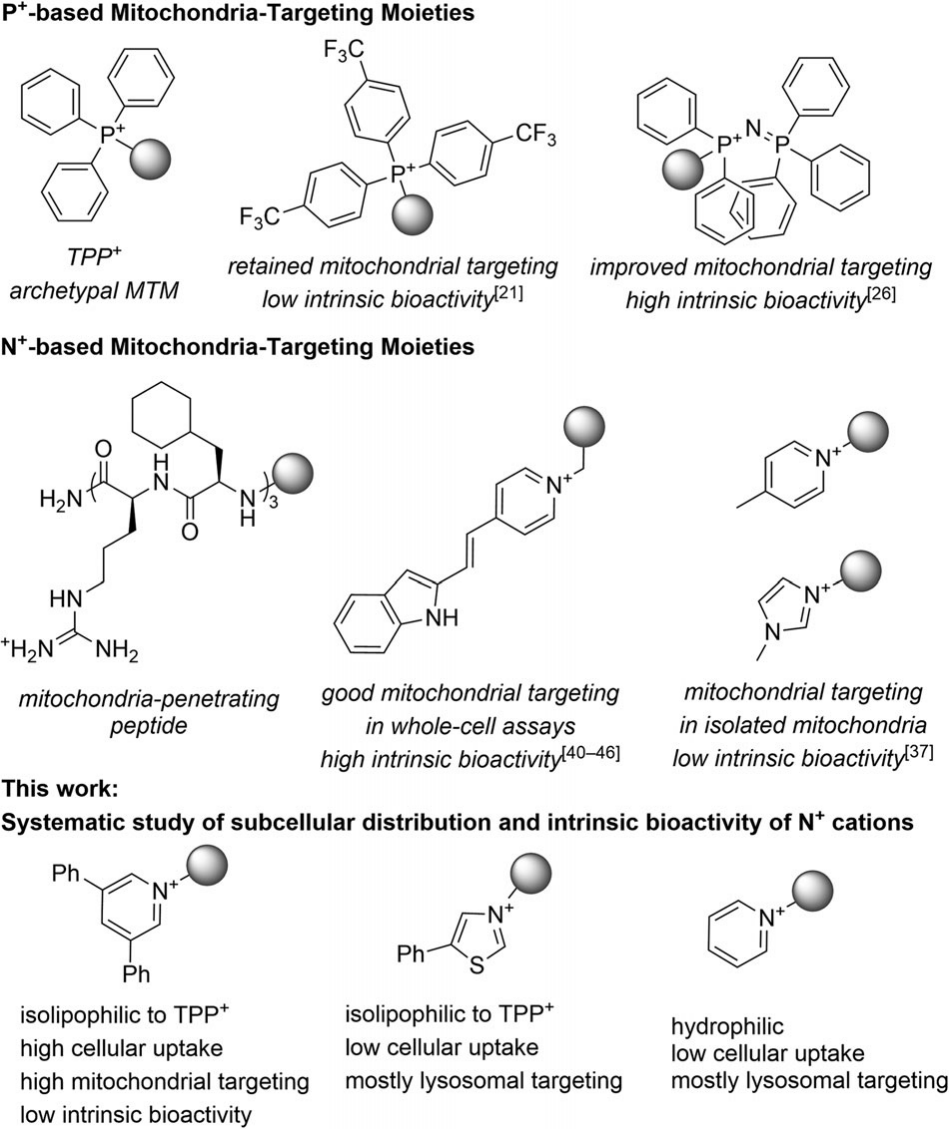
Overview of key literature precedents for phosphorus‐ and nitrogen‐based cations used as mitochondria‐targeting moieties. Grey spheres represent bioactive, fluorescent, or inert cargo intended for mitochondrial delivery.

Mitochondria are one of the most important subcellular structures for the development of organelle‐targeted therapies [1], due to their central roles in cellular signaling, metabolism, stress responses, survival, and programmed cell death [2, 3]. A defining feature of this organelle is its negative electrochemical potential across the inner mitochondrial membrane (ΔΨm) [4], which not only provides the thermodynamic driving force for ATP synthesis, but also supports multiple biosynthetic pathways, the import of nuclear‐encoded proteins and pre‐proteins, and the uptake of ions such as Ca^2+^, Na^+^, and Mn^2+^ [3, 5, 6, 7]. Importantly, the latter two ΔΨm‐driven processes are exploited by the principal mitochondria‐targeted cargo delivery strategies: the import of mitochondria‐targeting peptides [8], and the uptake of lipophilic cations [9]. MITochondria‐TArgeting Conjugates (MITACs) are designed by covalently linking mitochondria‐targeting moieties (MTMs) to functional cargo (such as modulators of mitochondrial targets, antioxidants, or organometallic catalysts), thereby enabling selective activity within mitochondria while minimizing effects in other cellular compartments [9, 10, 11, 12, 13, 14, 15, 16, 17]. Among MTMs, P‐tethered triphenylphosphonium (TPP^+^) remains the gold standard lipophilic cation (Figure 1) [18, 19]. However, TPP^+^ exhibits intrinsic bioactivity, which is reflected in the potent cytotoxicity of simple alkylated derivatives [20, 21], in its impact on cellular bioenergetics independent of the attached cargo [22], and in its contribution to interactions with mitochondrial proteins [23, 24].

An ideal MTM would deliver its cargo to mitochondria with high efficiency and specificity while exhibiting low intrinsic bioactivity. Optimization of TPP^+^ as an MTM has focused both on enhancing mitochondrial delivery efficiency [25, 26, 27, 28, 29, 30] and on reducing intrinsic cytotoxicity [21, 31]. For example, the tris‐(4‐trifluoromethylphenyl)phosphonium cation has been reported to display mitochondria‐targeting efficiency comparable to TPP^+^, but with a significantly reduced impact on mitochondrial oxidative phosphorylation and membrane potential [21]. A switch from phosphonium to other main group nonmetal permanent cations offers a promising, yet underexplored, alternative for developing MTMs with efficient mitochondrial targeting and potentially reduced intrinsic bioactivity. Permanent nitrogen cations are an intuitive first choice due to their natural presence, for example, in nicotinamide adenine dinucleotide phosphate (NADP) or carnitine.

Guanidinium, which is positively charged at physiological pH, has been utilized as a small‐molecule MTM [32] and incorporated into mitochondria‐penetrating peptides [8, 33]. N‐Tethered pyridinium cations have frequently been proposed as mitochondria‐targeting moieties [19, 34, 35]; however, unambiguous validation of their mitochondrial localization in intact cells remains limited. Moreover, recent work has demonstrated that permanently charged heterocycles can enhance cellular uptake without necessarily leading to mitochondrial accumulation, underscoring that subcellular localization of cationic species cannot be inferred from charge alone [36]. Conjugates incorporating N‐tethered isoquinolinium, 4‐picolinium, and N′‐methylimidazolium were shown to accumulate in isolated mouse liver mitochondria in a ΔΨm‐dependent manner, but their mitochondrial accumulation in whole cells was not examined [37]. To support the observed inhibition of mitochondrial cyclophilin D by cyclosporin conjugates incorporating quinolinium cations as MTMs [38], the mitochondria‐targeting ability of quinolinium cations was investigated by conjugating them to carboxyfluorescein and assessing colocalization with tetramethylrhodamine methyl ester. However, microscopy data supporting this colocalization were not provided in the original report [39]. Another notable example is the (*E*)‐4‐(2‐(1*H*‐indol‐3‐yl)vinyl)‐1‐methylpyridin‐1‐ium cation, known as F16 [40], which efficiently accumulates in mitochondria but exerts an uncoupling‐related mitochondrial toxicity that is independent of the pyridine N‐alkylation [41]. F16 has been employed as an MTM to deliver anticancer agents to mitochondria [42, 43, 44, 45], although in some cases, live‐cell imaging suggests relatively limited colocalization and overlap of intrinsically fluorescent F16 conjugates with MitoTracker Red [42, 43]. Interestingly, the pyridine‐3‐yl regioisomer of F16 demonstrates lower cytotoxicity, yet it has not been investigated as a potential MTM [46].

In the present study, we investigated the potential of N‐tethered heterocyclic permanent cations as MTMs. Key experiments were conducted in intact live cells, enabling direct assessment of subcellular distribution under physiologically relevant conditions while minimizing potential artefacts associated with simplified experimental systems. To enable a comprehensive evaluation, three classes of conjugates were synthesized [9]:

- conjugates with a non‐charged fluorescent dye to quantitatively assess mitochondrial delivery;
- conjugates with a mitochondrial ion channel inhibitor to examine the delivery of pharmacologically active cargo and the induction of biological activity within mitochondria;
- conjugates with an otherwise inert short alkyl chain to evaluate the intrinsic bioactivity of the permanent cations.

All cationic conjugates were systematically benchmarked against their TPP^+^ analogues in whole‐cell assays.

## 2 Results and Discussion

**FIGURE 2:**
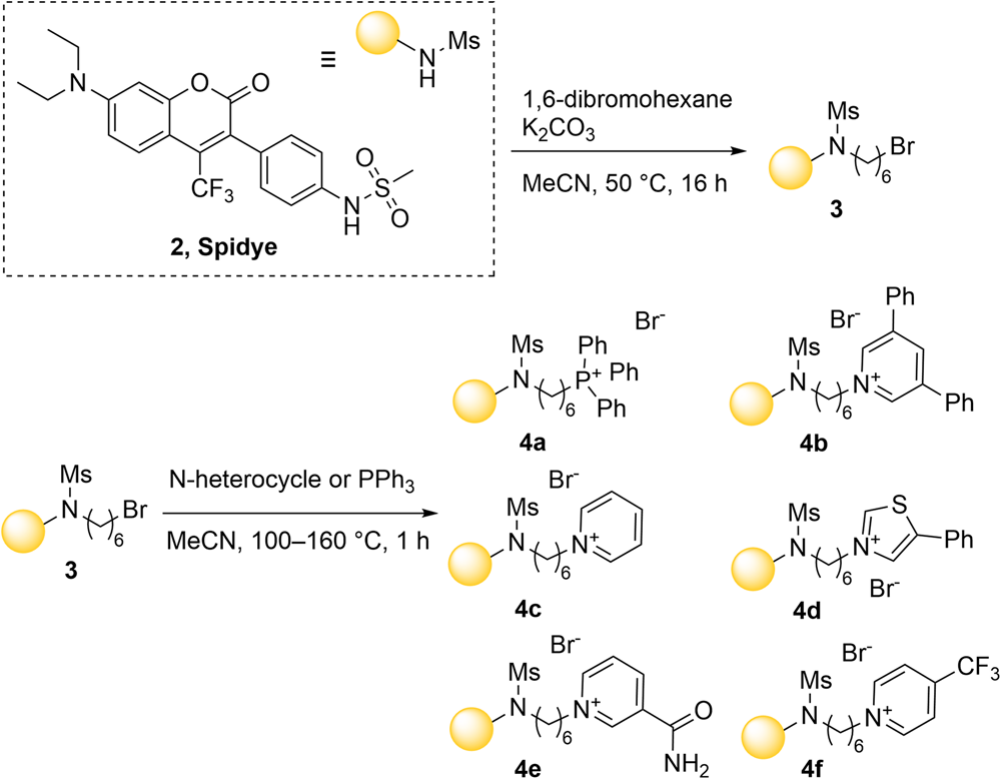
Synthesis of cationic conjugates with non‐charged large Stokes shift fluorescent dye.

To quantitatively assess the mitochondria‐targeting ability of N‐tethered heterocycles as cationic MTMs and compare them to the TPP^+^ benchmark, we first prepared the conjugates of triphenylphosphine (**1a**) and 3,5‐diphenylpyridine (**1b**) with fluorescein methyl ester (Figure S1). This strategy follows literature precedents for evaluating mitochondrial delivery efficiency of phosphonium‐based MTMs [25, 26]. The fluorescein conjugates **1a** and **1b** were co‐incubated with MitoTracker Deep Red in COLO‐357 cells, and colocalization of fluorescein fluorophore (excitation *λ* = 488 nm, emission *λ* = 523 nm) and MitoTracker Deep Red fluorophore (excitation *λ* = 633 nm, emission *λ* = 654 nm) was investigated by live‐cell imaging. In our hands, the fluorescein‐based MTM‐conjugates **1a** and **1b** were not suitable for reliable quantification of the mitochondria‐targeting ability, because of significant cell autofluorescence at the fluorescein excitation and emission wavelengths (Figure S1) [47, 48]. Concurrently, the 3‐(4‐mesylaminophenyl)‐7‐diethylamino‐4‐trifluoromethylcoumarin scaffold **2**, dubbed Spidye, was being developed in our laboratories as a non‐charged hydrolytically stable and photostable fluorescent probe with a large Stokes shift (excitation *λ* = 405 nm, emission *λ* = 550–600 nm) [49], which would eliminate the cell autofluorescence‐related issues. Its mesylamino derivatization handle further enables the convenient synthesis of conjugates (Figure 2).

Spidye was alkylated with 1,6‐dibromohexane to get the bromoalkyl derivative **3**, which was used as a reagent to prepare the benchmark mitochondria‐targeted TPP^+^ conjugate **4a**, and a series of quaternary N‐heterocycles **4b**–**4f** (Figure 2). The selection of cations was designed to span a broad lipophilicity range. The 3,5‐diphenylpyridinium (DPPy^+^) and thiazolium cations (**4b** and **4d**) were chosen for their high predicted lipophilicity, comparable to that of the TPP^+^ analogue **4a** [50], whereas the hydrophilic unsubstituted pyridinium **4c** and nicotinamide derivative **4e** represent the polar end of the series, completed by the **4d** with intermediate polarity and *p*‐CF_3_ substituent previously associated with reduced oxidative phosphorylation uncoupling in the context of phosphonium‐based MTMs [21].

**FIGURE 3:**
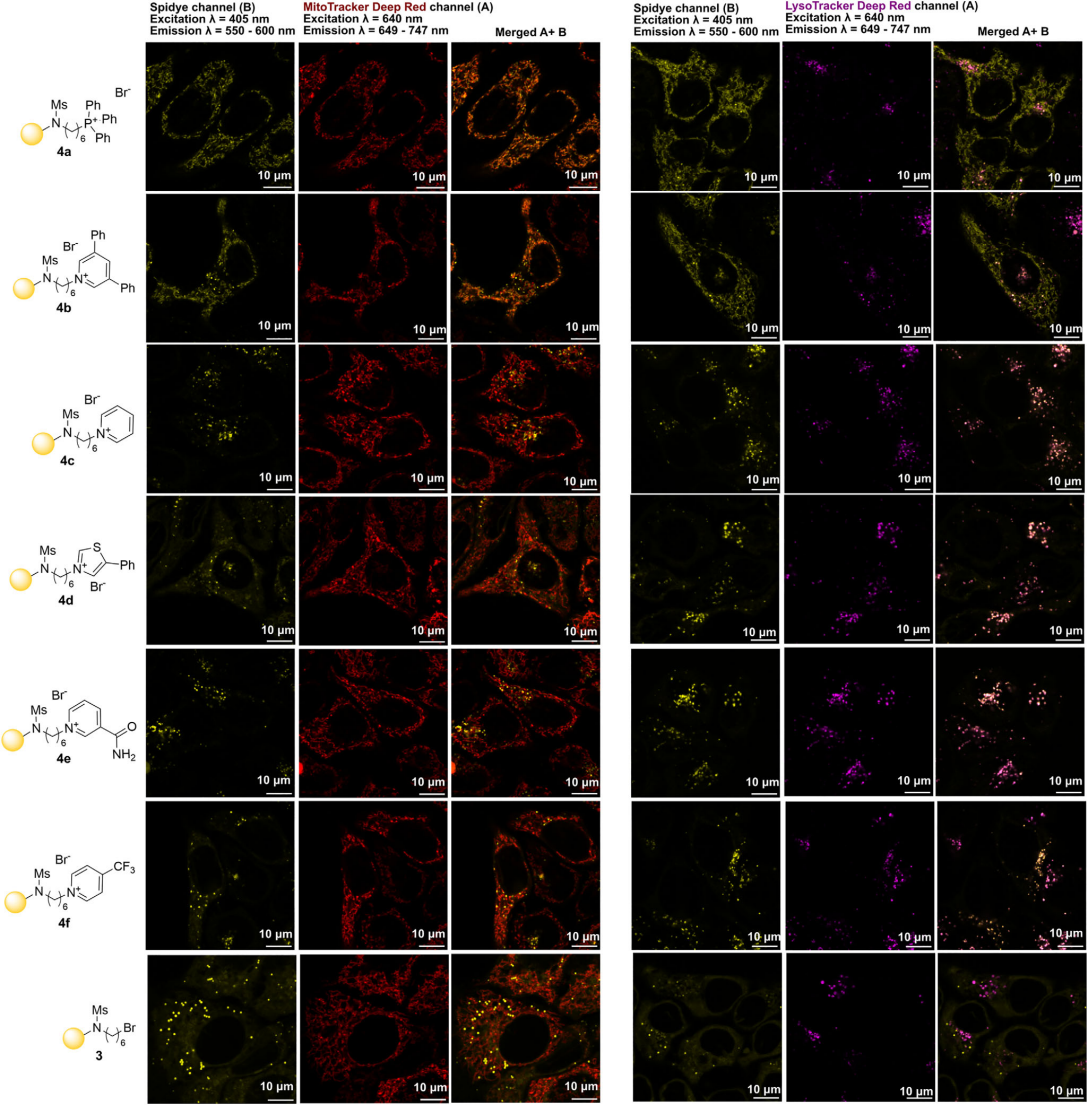
Live‐cell imaging study of mitochondrial and lysosomal localization of Spidye conjugates **3**–**4** (100 nM) in COLO‐357 cells. The fluorescent dye conjugates were not cytotoxic at the used concentration (see Figure S14). MitoTracker Deep Red and LysoTracker Deep Red were used as reference organelle‐specific stains (50 nM). Microscopy images were obtained using Nikon Ti2 AX confocal microscope and are representative of at least two biological replicates.

First, we showed by live‐cell imaging experiments that the non‐charged compound **3** does not accumulate in mitochondria, whereas the permanently charged TPP^+^ conjugate **4a** exhibited a very high degree of colocalization with the established mitochondrial dye MitoTracker Deep Red in COLO‐357 cells (Figure 3). The latter was selected as a model cell line because of its high mitochondrial mass (Figure S2). After establishing these benchmarks, we used live‐cell imaging to quantitatively assess the mitochondria‐targeting ability of quaternary N‐heterocycles. Cellular uptake and mitochondrial localization of the fluorescent conjugates **4** was evaluated in live COLO‐357 cells by confocal microscopy (Figure 3 and Table 1). Cellular uptake of the N^+^‐based conjugates **4b**–**4f** relative to the TPP^+^‐based conjugate **4a** was assessed by comparing the total fluorescence intensity of the Spidye channel (excitation *λ* = 405 nm, emission 550–600 nm) with that of the MitoTracker Deep Red channel. The DPPy^+^ conjugate **4b** had cellular uptake comparable to the TPP^+^ conjugate **4a**. The cellular uptake of the unsubstituted pyridinium conjugate **4c** and the phenylthiazolium conjugate **4d** was significantly lower, and even lower for the aminocarbonyl‐substituted **4e** and the trifluoromethyl‐substituted **4f**. To assess mitochondrial targeting, localization was quantified by Pearson's correlation coefficient and Manders’ co‐occurrence coefficients of MitoTracker Deep Red and Spidye fluorescence channels across pixels (Table 1) [51, 52, 53, 54]. The TPP^+^ conjugate **4a** and DPPy^+^ conjugate **4b** were enriched in mitochondria, exhibiting a high degree of colocalization and overlap with MitoTracker Deep Red (Pearson's and Manders’ coefficients > 0.7) [31]. The fluorescent conjugates **4c**–**4f** did not selectively distribute to mitochondria, as evidenced by low colocalization with MitoTracker Deep Red upon visual inspection and low Pearson's and Manders’ coefficients.

**TABLE 1:** Profiling of conjugates of permanently charged moieties with fluorescent dye.

Cpd	LogD^a^ (pH 7.4)	F_B_/F_A_ ^b^ (MitoTracker)	Pearson^c^ (MitoTracker)	M1^d^ (MitoTracker)	M2^e^ (MitoTracker)	Pearson^c^ (LysoTracker)	M1^d^ (LysoTracker)	M2^e^ (LysoTracker)
**4a**	2.99	0.94 ± 0.02	0.88 ± 0.06	0.85 ± 0.04	0.87 ± 0.04	0.25 ± 0.07	0.52 ± 0.20	0.06 ± 0.02
**4b**	3.08	0.83 ± 0.12	0.79 ± 0.07	0.71 ± 0.09	0.74 ± 0.06	0.75 ± 0.14	0.94 ± 0.04	0.4 ± 0.2
**4c**	−0.08	0.53 ± 0.11	0.43 ± 0.05	0.38 ± 0.19	0.38 ± 0.04	0.93 ± 0.07	0.86 ± 0.06	0.90 ± 0.03
**4d**	3.52	0.47 ± 0.07	0.42 ± 0.01	0.10 ± 0.03	0.51 ± 0.16	0.85 ± 0.08	0.76 ± 0.12	0.85 ± 0.09
**4e**	0.35	0.32 ± 0.08	0.27 ± 0.05	0.11 ± 0.06	0.44 ± 0.10	0.93 ± 0.02	0.87 ± 0.07	0.87 ± 0.08
**4f**	1.28	0.33 ± 0.10	0.53 ± 0.07	0.26 ± 0.18	0.67 ± 0.10	0.92 ± 0.03	0.84 ± 0.12	0.85 ± 0.06
**3**	>6	0.42 ± 0.09	0.43 ± 0.08	0.05 ± 0.02	0.34 ± 0.17	0.53 ± 0.21	0.79 ± 0.44	0.11 ± 0.07

^a^ ^)^Experimentally determined octanol/phosphate‐buffered saline (pH 7.4) distribution coefficient.

^b^ ^)^Fluorescence intensity ratio of channel A and channel B across the selected image region of interest, wherein A was used for MitoTracker Deep Red or LysoTracker Deep Red detection (excitation λ = 640 nm; emission *λ* = 649–747 nm), and B was used for detection of Spidye conjugates (excitation *λ* = 405 nm; emission *λ* = 550–600 nm).

^c^ ^)^Linear correlation of fluorescence intensities in A and B across pixels.

^d^ ^)^Fraction of pixels in channel A overlapping with positive pixels in channel B.

^e^ ^)^Fraction of positive pixels in channel B overlapping with positive pixels in channel A. Data in [b–e] represent mean ± SD of at least 3 images from at least two independent experiments.

The characteristic patterns (see Figure 3) in the fluorescence microscopy images of the unsubstituted pyridinium conjugate **4c** prompted us to also investigate the lysosomal localization of all the cationic fluorescent dye conjugates. Note that lysosome‐targeted molecular probes typically bear a weakly basic handle leading to their accumulation in acidic vesicles upon protonation [55], which was not expected for the permanently charged nitrogen heterocycles. Colocalization with LysoTracker Deep Red was investigated in separate experiments in live COLO‐357 cells. Because of the relatively high mobility of lysosomes [56], images were acquired using a line‐interleaved (fast sequential) scanning mode, which reduces temporal offset between channels. The large Stokes shift of Spidye was essential for minimizing fluorescence crosstalk. Indeed, for the Spidye–pyridinium conjugate **4c,** a high degree of correlation and overlap was observed with LysoTracker Deep Red, whereas the Pearson's and Manders’ coefficients were lower with MitoTracker Deep Red (Figure 3, Table 1). This suggests a preferential lysosomal, rather than mitochondrial, localization of the unsubstituted pyridinium conjugates in whole cells, in contrast to the hitherto accepted paradigm [19, 34, 35]. Similarly, the hydrophilic (logD = 0.35) nicotinamide conjugate **4e** displayed higher localization in lysosomes than in mitochondria, as did the lipophilic (logD = 3.52) phenylthiazolium conjugate **4d**. The DPPy^+^–Spidye conjugate **4b** was highly localized in mitochondria of COLO‐357 cells, based on its high Pearson's and Manders’ coefficients with MitoTracker Deep Red, but it also displayed a high M1 coefficient with LysoTracker Deep Red. The latter suggests that most of lysosomes contain **4b**, however the majority of **4b** is not localized in lysosomes (low M2), but in mitochondria. The TPP^+^ conjugate **4a** was highly mitochondria‐specific, with low correlation and overlap with LysoTracker Deep Red. Subcellular distribution of the fluorescent conjugates **4** was investigated in two additional cell lines HeLa (Figure S3, Table S1) and NHLF (Figure S4, Table S2). The observed trend in HeLa cells was similar to COLO‐357, i.e. **4b** was localized mostly in mitochondria, but also in lysosomes, and **4c**–**4f** were highly localized in lysosomes with low mitochondrial localization. Notably, the lysosomal localization of **4b** in NHLF cells was lower compared to the cancerous cell lines, whereas the mitochondrial localization was still high and comparable to **4a**.

To elucidate the mechanism underlying the unexpected lysosomal localization observed for the simple pyridinium conjugate **4c**, we examined the influence of lysosomal pH on its subcellular distribution in COLO‐357 cells. FITC‐dextran served as a lysosomal marker that accumulates in lysosomes irrespective of luminal pH, allowing their identification even upon pH perturbation, and bafilomycin A1 was used to raise lysosomal pH (Figure S5) [57]. In untreated cells, **4c** colocalized with FITC‐dextran‐labeled lysosomes. By contrast, lysosomal localization of **4c** was lost upon bafilomycin A1 treatment, irrespective of whether **4c** was added before or after bafilomycin (Figure S6), indicating pH‐dependent lysosomal localization. Furthermore, to examine whether pyridinium redox cycling [58, 59] in live cells, followed by ion trapping of the more basic reduced species, contributes to the observed pH‐dependent lysosomal localization, we prepared the Spidye conjugate of deuterated pyridine, **4c‐*d*_5_** (Figure S7). This compound exhibited a subcellular distribution similar to that of **4c** in the live‐cell imaging experiment (Figure S8, Table S3). COLO‐357 cells were then incubated with **4c‐*d*_5_** for 1 h or 24 h, and the cell lysate was analyzed by LC‐MS to detect possible formation of a reduced species or a rearomatized pyridinium cation arising from deuterium–proton exchange (Figures S9–S12). However, neither a reduced species nor D/H exchange of **4c‐*d*_5_** was detected, indicating that the *N*‐alkylpyridinium cations are redox‐stable under these conditions in live cells. The DPPy^+^ conjugate **4b** and the pyridinium conjugate **4c** were further analyzed by live‐cell imaging in the presence of carbonyl cyanide *p*‐(trifluoromethoxy)phenylhydrazone (FCCP) to depolarize mitochondria (Figure S13). This experiment was designed to confirm the ΔΨm‐dependence of mitochondrial localization and to assess a possible contribution of mitochondrion–lysosome trafficking (e.g., via mitophagy [60] or mitochondria–lysosome contacts [61]) to the observed lysosomal sequestration. In FCCP‐treated cells, both conjugates **4b** and **4c** exhibited reduced mitochondrial localization and increased lysosomal localization, as determined by colocalization analysis with LysoTracker Deep Red (Table S4). These results indicate that ΔΨm is the primary driver of mitochondrial accumulation of pyridinium cations, whereas the increased lysosomal localization upon mitochondrial depolarization argues against a major contribution of mitochondria‐derived trafficking processes to lysosomal sequestration.

**FIGURE 4:**
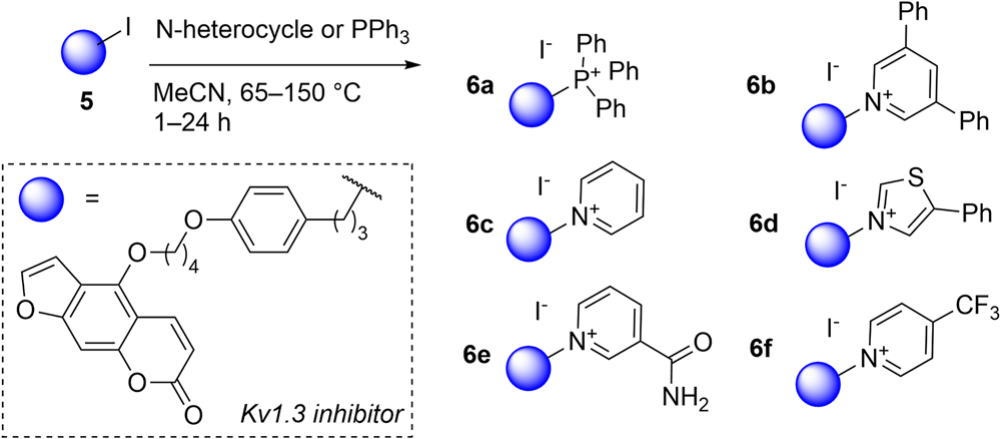
Synthesis of cationic conjugates with bioactive cargo, the Kv1.3 inhibitor PAP‐1.

Next, we evaluated the ability of permanently charged nitrogen heterocycles to deliver bioactive cargo to mitochondria and induce relevant biological effects on a cellular level. As a model for a bioactive functional cargo, we selected an inhibitor of the voltage‐gated potassium ion channel Kv1.3 [62]. Inhibitors without covalently attached MTM, such as the psoralen‐cored PAP‐1, exhibit only a modest effect on Kv1.3‐expressing cancerous cells (60% survival of B16F10 cells at 20 µM of PAP‐1 in the presence of multidrug resistance pump inhibitors) [63]. In contrast, their conjugates with TPP^+^ as MTM, such as compound **6a** (Figure 4), efficiently induce apoptosis and reduce cancer cell viability via inhibition of mitochondrial Kv1.3 [11, 64]. The conjugates **6a**–**6f** were prepared from the corresponding alkyl iodide **5** [11] by a convenient final‐stage introduction of MTM (Figure 4) [37]. We selected two cancer cell lines to study the bioactivity of conjugates **6**; COLO‐357 is a human pancreatic ductal adenocarcinoma line with confirmed Kv1.3 expression [11, 65], and B16F10 is a mouse melanoma cell line that was shown to undergo apoptosis upon exposure to membrane‐permeant mitochondrial Kv1.3 inhibitors [63]. We assessed the ability of the heterocyclic cations to deliver the Kv1.3 inhibitor into mitochondria and induce the expected biological response by measuring cell viability via a resazurin‐based assay after 72 h (Table 2), with dose–response curves shown in Figure S14.

**TABLE 2:** Profiling of cationic conjugates with psoralen‐based Kv1.3 inhibitor.^a^

Cpd	COLO‐357 IC_50_ (µM)^a^	B16F10 IC_50_ (µM)^a^	Log*D* ^b^ (pH 7.4)	cLog*P* ^c^ (SwissADME)
**6a**	0.53 ± 0.11	0.44 ± 0.01	2.41	7.94
**6b**	1.84 ± 0.59	0.45 ± 0.05	2.52	6.79
**6c**	13.4 ± 1.1	>5	0.12	4.28
**6d**	>30	>5	2.77	5.59
**6e**	13.8 ± 6.4	>5	0.02	3.25
**6f**	27.9 ± 1.5	>5	0.73	5.15

^a^ ^)^IC_50_ values correspond to half maximal inhibitory concentrations in resazurin assay after 72 h exposure and are given as mean ± SD (n ≥3 biological replicates, each with ≥3 technical replicates).

^b^ ^)^Experimentally determined octanol/phosphate‐buffered saline (pH 7.4) distribution coefficient.

^c^ ^)^cLog*P*, consensus octanol/water partition coefficient calculated in SwissADME [50].

The profiling of MTM conjugates with Kv1.3 channel inhibitor **6a**–**6f** revealed the same trend as for the fluorescent‐dye conjugates, that is, the DPPy^+^ conjugate **6b** was roughly equipotent to the benchmark TPP^+^ conjugate **6a** (PAPTP), and the conjugates with more polar pyridines **6c**, **6e**, **6f**, and with 5‐phenylthiazol **6d** were inactive. The two active compounds **6a** and **6b** were also tested on human and mouse non‐cancerous cell lines NHLF (normal human lung fibroblasts) and C2C12 (mouse myoblasts). In mouse fibroblasts, both compounds exhibited a degree of selectivity compared to the mouse melanoma cells B16F10; **6a** IC_50_ (C2C12) = 1.61 ± 0.02 µM, **6b** IC_50_ (C2C12) = 1.57 ± 0.02 µM. In NHLF, the activity was in the same range as in the human cancer cell line: **6a** IC_50_ (NHLF) = 0.72 ± 0.04 µM, **6b** IC_50_ (NHLF) = 1.96 ± 0.15 µM. (cf. COLO‐357, Table 2).

The effect of MTM variation on the on‐target activity was assessed by whole‐cell patch‐clamp recordings from cells stably expressing cloned mouse Kv1.3 channels on the plasma membrane (Figure S15, Table S5) [66]. Importantly, both the DPPy^+^ conjugate **6b** and the unsubstituted‐pyridinium conjugate **6c** inhibited Kv1.3 with IC_50_ = 0.89 µM (CI_95_ = 0.22–1.7 µM) and IC_50_ = 1.96 µM, respectively. The Kv1.3 inhibitory activity of the pyridinium conjugates **6b** and **6c** was lower than that of the TPP^+^ conjugate **6a**, IC_50_ = 0.23 µM (CI_95_ = 0.162–320 µM). Notably, the most probable binding site of the psoralene‐cored inhibitors is the inner pore of Kv1.3 [67], therefore the cell permeability of the compounds also affects the potency in the electrophysiology assay.

**FIGURE 5:**
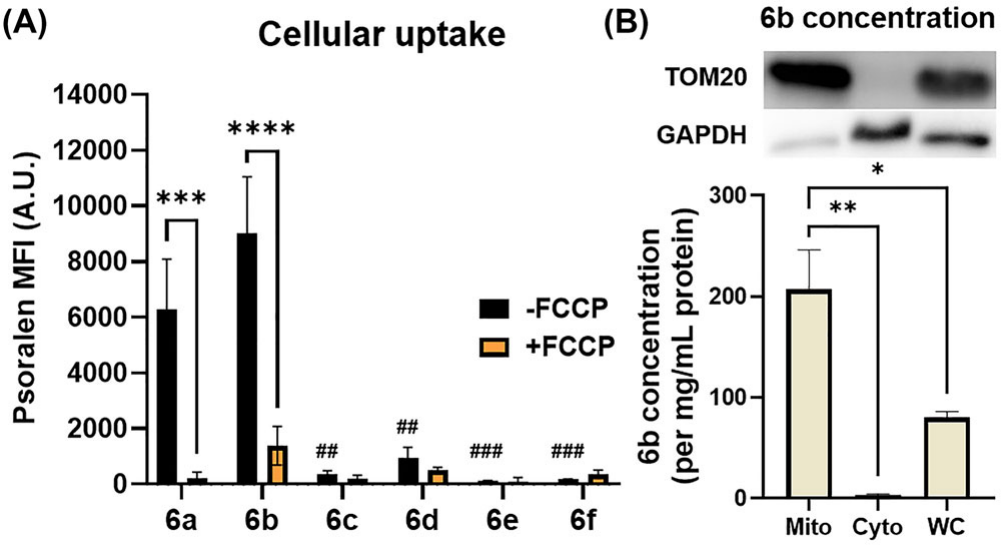
(A) Mitochondrial membrane potential‐dependent cellular uptake of psoralen‐based Kv1.3 inhibitors assessed by flow cytometry in COLO‐357 cells using FCCP to cause membrane depolarization prior to incubation with Kv1.3 inhibitors. ##*p* < 0.01, ###*p* < 0.001 versus **6a** as determined by two‐way ANOVA with Dunnett's post‐hoc test. ***p < 0.001, ****p < 0.0001 as determined by two‐way ANOVA with Šidak's post‐hoc test. (B) Determination of the concentration of **6b** in mitochondrial (Mito), cytosolic (Cyto) and whole cell (WC) fraction, by HPLC‐MS after fractionation of COLO‐357 cells treated by **6b** (1 µM for 1 h). Data represent the mean ± SEM for at least three independent experiments. *p < 0.05, **p < 0.01 as determined by one‐way ANOVA with Tukey's post‐hoc test. A representative western blot for mitochondrial (TOM20) and cytosolic (GAPDH) proteins in the corresponding cellular fractions is shown in (B). The full‐size gels are shown in Figures S17–S18.

To assess the cellular uptake of the bioactive cargo conjugates **6a**–**6f**, the total cell fluorescence of the weakly fluorescent psoralen core was measured by flow cytometry in COLO‐357 cells (Figure S16). To evaluate ΔΨm‐dependence of this cellular uptake, a parallel set of cells was pre‐treated with 50 µM FCCP to depolarize the mitochondria prior to fluorescence measurement. The cellular uptake of **6a** and **6b** was significantly higher, compared to the conjugates **6c**–**6f**. Moreover, the majority of the cellular uptake for both **6a** and **6b** was ΔΨm‐dependent, with **6b** exhibiting comparable cellular uptake and slightly lower ΔΨm‐dependent fraction than **6a** (87% vs. 98%, Figure 5A). In contrast, the low cellular uptake of other compounds was largely ΔΨm‐independent.

As an additional confirmation of the mitochondria‐targeting ability of the DPPy^+^ cation, mitochondrial accumulation of **6b** was assessed by isolating the mitochondrial and cytosolic fractions of COLO‐357 cells treated with 1 µM **6b** according to a modified published protocol [68]. Western blot analysis confirmed the successful fractionation, with the cytosolic fraction completely free from mitochondria and the mitochondrial containing trace cytosolic GAPDH (Figure 5B and Figures S17 and S18). The mitochondrial fraction contained the lysosomal LAMP1 (by western blot, Figure S19) therefore this method does not discriminate mitochondrial from the lysosomal accumulation. The amount of **6b** in the isolated fractions was determined by HPLC‐MS analysis (Figures S20 and S21) and normalized to the amount of protein as a proxy of subcellular compartment volume. The mean concentration of **6b** was 65‐fold (± 10) higher in the mitochondria/lysosomal versus the cytosolic fraction (Figure 5B). This is comparable to the mitochondrial versus cytosolic enrichment of TPP^+^ conjugates with the HSP90 inhibitor PU‐H71 and gamitrinib with 17‐fold and 106‐fold enrichment, respectively [69].

The cellular uptake of compounds might correlate to their lipophilicity [70], therefore the distribution coefficient in octanol and phosphate‐buffered saline (pH 7.4) was determined experimentally by the shake‐flask method. Notably, the experimentally determined Log*D* values for the permanent cations **4** (fluorescent cargo conjugates, see Table 1), as well as **6** (bioactive cargo conjugates, see Table 2) were lower than the calculated cLog*P*. The values from SwissADME are shown, but a similar trend was observed using the algorithms from ChemDraw or DataWarrior [71]. This indicates that the introduction of a lipophilic cation as MTM is not critically detrimental to the overall physicochemical properties of the whole conjugate. Moreover, the experimental Log*D* values of **4a**, **4b** and **4d** (Table 1), as well as **6a**, **6b** and **6d** (Table 2) are comparable. Therefore, the DPPy^+^ cation is a good model pyridinium cation to be benchmarked against TPP^+^ cation without the lipophilicity bias. The cellular uptake and the ΔΨm‐dependent fraction of compound **6d**, relative to the isolipophilic compounds **6a** and **6b**, were modest, which is consistent with the trends in cellular uptake and mitochondrial localization seen in fluorescence microscopy studies for the same cations. These results indicate that permanent cations with similar lipophilicity cannot be assumed to be uniformly mitochondriotropic with pure Nernstian behavior [9].

**FIGURE 6:**
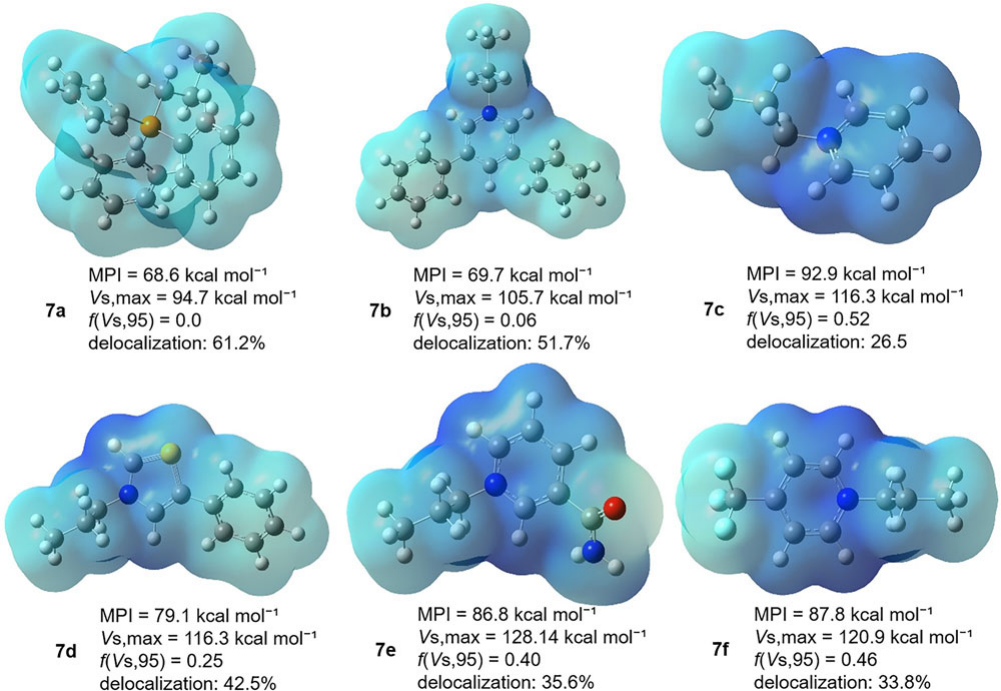
Electrostatic potential (ESP) mapped onto the electron‐density isosurface (*ρ* = 0.001 a.u.) for the propyl‐substituted cations **7a**–**7f**. The electrostatic and polarity descriptors are computed at the ωB97X‐D/def2‐TZVP level. MPI, molecular polarity index; *V*s, max, maximum positive surface potential; *f*(*V*s, 95), fraction of the surface area with *V*s > 95 kcal mol^−1^. Delocalization is quantified as the fraction of +1 charge residing outside the formal cationic headgroup based on the Hirshfeld fragment charges; for details, see Supporting Information.

Charge delocalization has frequently been discussed as a key parameter for enhanced membrane permeability of cationic MTMs [26, 38, 40, 72], although permanent cations without resonance‐stabilized charge delocalization, such as tri(cyclo)alkylphosphonium, have also been shown experimentally to accumulate efficiently in mitochondria [31, 73, 74]. Charge distribution in the investigated cations was assessed computationally at the ωB97X‐D/def2‐TZVP level of theory by Hirshfeld population analysis [75] of the propyl‐substituted cations **7a**–**7f** (Table S10), and by quantitative molecular surface analysis to generate Electrostatic potential (ESP) maps (Figure 6). Single‐crystal X‐ray diffraction analysis of *N*‐propyl‐3,5‐diphenylpyridinium chloride **7b** (Figures S22–S26, Tables S7–S9) [76] revealed the C─C bond distances between the phenyl rings and the pyridinium core (∼1.5 Å) consistent with single‐bond character; the phenyl and pyridinium aromatic rings are non‐coplanar in both crystallographically independent cations within the asymmetric unit, which is in good agreement with the in silico‐optimized structure of **7b**.

Hirshfeld fragment charges were used to quantify the charge distribution in the cationic frameworks and thus evaluate the extent of positive charge delocalization from the formal cationic headgroup (Table S10). The two efficient mitochondria‐targeting cations (**7a**, **7b**) retain 0.39 and 0.48 *e* on the formal headgroup (61% and 52% delocalization, respectively), whereas the other N^+^‐based cations (**7c**–**7f**) localize substantially more charge on the heterocyclic headgroup (26%–36% delocalization). The increased headgroup charge localization is consistent with the visualization of the ESP analysis (Figure 6). For quantitative analysis, the numerical descriptors were extracted from the ESP distributions. The molecular polarity index (MPI) [77] is a single‐number descriptor of ESP uniformity, essentially an average deviation of ESP over the surface. The mitochondria‐targeting cations **7a** and **7b** have similar MPI, whereas **7c**–**7f** exhibit more surface‐polarity heterogeneity (MPI ≥ 80 kcal/mol). The maximum positive surface potential (*V*s, max) has previously been used as a molecular descriptor correlating with mitochondrial targeting [26]. Additionally, we calculated the fraction of positive surface hotspots, defined as *V*s > 95 kcal mol^–^^1^, *f*(*V*s, 95). The cations with lower mitochondrial localization **7c**–**7f** display higher *V*s, max and higher *f*(*V*s, 95), whereas **7a** and **7b** exhibit a more diffuse, less hotspot‐enriched electrostatic field.

**FIGURE 7:**
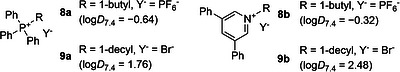
Conjugates of DPPy and TPP with otherwise inactive cargo.

Based on the profiling of MTM–(fluorescent dye) and MTM–(Kv1.3 inhibitor) conjugates, DPPy^+^ was identified as a promising model pyridinium‐based cationic MTM. To evaluate its fragment contribution to bioactivity in comparison to TPP^+^, a series of derivatives of DPPy^+^ and TPP^+^ with otherwise inactive cargo (alkyl chain) were prepared (Figure 7).

*P*‐Butyltriphenylphosphonium hexafluorophosphate **8a** indeed exhibited cytotoxic activity, IC_50_ = 2.88 ± 0.50 µM, in the resazurin assay in COLO‐357 cells, whereas *N*‐butyl‐3,5‐diphenylpyridinium **8b** was significantly less cytotoxic, IC_50_ = 16.2 ± 4.6 µM. Decyltriphenylphosphonium bromide **9a** was found very cytotoxic in the COLO‐357 cell line, IC_50_ = 0.55 ± 0.43 µM, in the range of the corresponding Kv1.3 inhibitor **6a**. Similarly, the *N*‐decyl‐3,5‐diphenylpyridinium bromide **9b**, IC_50_ = 1.89 ± 0.22 µM, was more cytotoxic than the butyl derivative **8b**, but less than the analogous triphenylphosphonium **9a**. For the inactive cargo conjugates **8** and **9**, the experimentally determined octanol/buffer partition coefficients of the DPPy^+^ conjugates were slightly higher than the corresponding TPP^+^ conjugates (Figure 7). The higher observed cytotoxicity of the TPP^+^ conjugates **8a** and **9a** is therefore not a simple function of lipophilicity.

**FIGURE 8:**
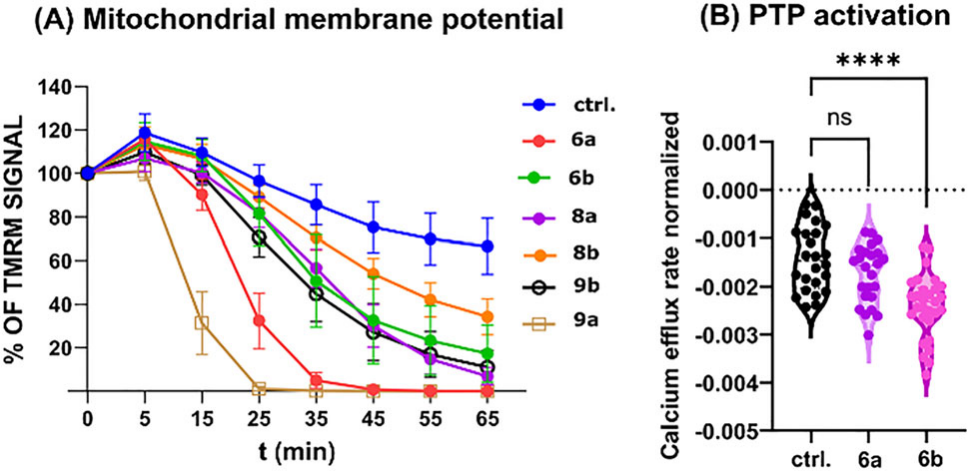
(A) Time‐dependent effects of bioactive and inactive cargo conjugates (10 µM) with TPP^+^ and DPPy^+^ on ΔΨm, determined in TMRM assay in COLO‐357 cells (three independent experiments; error bar represents the standard deviation). For concentration‐dependence experiments, see Figure S28. (B) For the mPTP opening experiment, COLO‐357 cells were loaded with calcein‐AM and CoCl_2_. mPTP opening was monitored by the decrease in calcein fluorescence induced by 10 µM ionomycin in COLO‐357 cells pre‐treated for 1 h with DMSO (black line), 10 µM conjugate **6a** (violet line), or 10 µM conjugate **6b** (pink line). mPTP opening occurs after the addition of ionomycin (6 independent experiments, *n* = 24–29). One‐way ANOVA with Dunnett's multiple comparisons test, ****p < 0.01 vs. DMSO.

To specifically elucidate the effects of the Kv1.3 inhibitor conjugates **6** and the alkyl conjugates **8** and **9** on mitochondrial function, the JC‐10 assay was used to evaluate their effect on ΔΨm in COLO‐357 cells after 45 min exposure (Figure S27) by spectrofluorimetric measurement of fluorescence. The butyl‐ and decyl‐TPP^+^ cations **8a** and **9a** induced strong mitochondrial depolarization at 10 µM, the latter already at 1 µM. The DPPy^+^ conjugates **8b** and **9b** induced significantly less depolarization at the tested concentrations. The effect of Kv1.3 inhibitor conjugates **6** on depolarization correlated with their resazurin assay activity in COLO‐357 cells; with compounds **6a** and **6b** decreasing both cell viability and ΔΨm in a dose‐dependent manner. The concentration‐ and time‐dependent effects of compounds **6a**, **6b, 8** and **9** on ΔΨm were evaluated by microscopy using tetramethylrhodamine methyl ester (TMRM) as a ΔΨm‐dependent fluorescent dye (Figures 8A and S28). In agreement with the complementary JC‐10 results, the TPP^+^‐based inactive cargo conjugates **8a** and **9a** caused stronger mitochondrial depolarization than the corresponding DPPy^+^ analogues **8b** and **9b**. For the Kv1.3 inhibitor conjugates **6a** and **6b**, mitochondrial depolarization may result from activation of the mitochondrial permeability transition pore (PTP), triggered by Kv1.3 inhibition and leading to apoptosis [11]. A significant PTP activation was observed in COLO‐357 cells after treatment with **6b** (10 µM), whereas the effect of **6a** was less pronounced (Figure 8B and S29). Both **6a** and **6b** efficiently induced apoptosis in COLO‐357 cells at 10 µM after 48 h as determined by the Caspase 3/7 assay (Figure S30).

**FIGURE 9:**
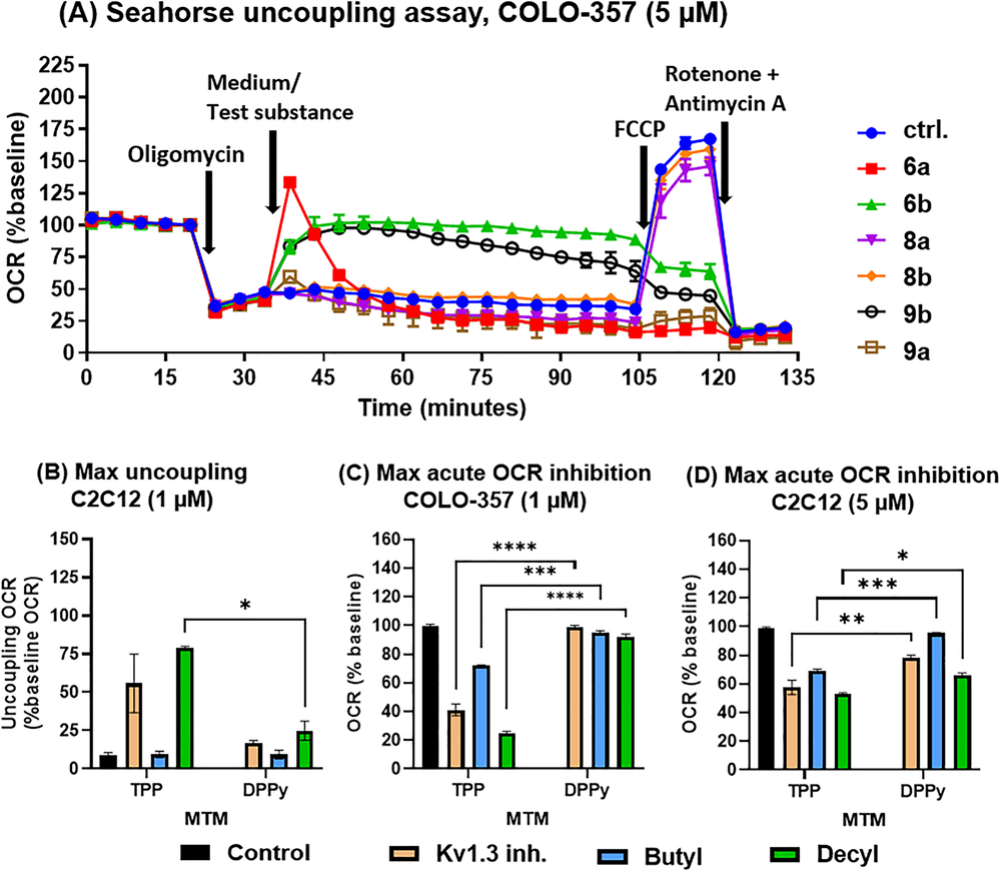
Effects of TPP^+^ and DPPy^+^ conjugates on mitochondrial uncoupling (A, B) and inhibition of cellular respiration (C, D) in COLO‐357 (A, C) and C2C12 (B, D) cells at 1 µM (B, C) and 5 µM (A, D). A representative time‐course for oxygen consumption rate (OCR) is shown in A. Maximum uncoupling or OCR inhibition ± SEM is shown in B–C for two independent experiments. *p < 0.05, **p < 0.01, ***p < 0.001, ****p < 0.0001 by two‐way ANOVA (Šidak's post‐hoc test). For comprehensive results, see Figures S31–S34.

The effect on the mitochondrial function was further evaluated by the extracellular flux (Seahorse) assay in cancerous COLO‐357 cells and C2C12 as non‐cancerous controls (Figure 9, Figure S31–S34) [11, 37, 78]. Both **6a** and **6b** exhibited a potent and comparable transient uncoupling effect at 5 µM in both cell lines, consistent with their mechanism of action as mitochondrial Kv1.3 inhibitors [11], with more gradual onset and more persistent elevation of oxygen consumption rate (OCR) for **6b** (Figure 9A). A similar uncoupling effect was observed for decyl conjugates **9a** and **9b**, with TPP^+^‐based **9a** inducing substantial uncoupling already at 1 µM in non‐tumor control cells (Figure 9B). In contrast, its DPPy^+^ analogue **9b** required 5 µM for strong uncoupling. The TPP^+^ conjugates exhibited stronger inhibition of mitochondrial respiration (Figure 9B–D). Notably, the butyl‐substituted TPP^+^ conjugate **8a** suppressed OCR in both cell lines, even at 1 µM in COLO‐357 cells, whereas the corresponding DPPy^+^ analogue **8b** showed no detectable inhibition of respiration at concentrations up to 5 µM. Similarly, both the DPPy^+^‐based decyl and Kv1.3 inhibitor conjugates **9b** and **6b** showed weaker OCR inhibition versus their TPP^+^ analogs **9a** and **6a**. To distinguish between specific mitochondrial effects and non‐specific cytotoxicity, extracellular acidification rate (ECAR) was monitored in parallel (Figures S33 and S34). For all compounds, ECAR remained elevated relative to baseline following OCR inhibition, indicating preserved glycolytic activity and supporting a mechanism involving selective mitochondrial inhibition rather than general loss of cell viability. To further elucidate the molecular basis of the differential respiratory inhibition, we investigated the effect of the butyl‐substituted conjugates **8a** and **8b** on Complex I activity. In line with literature reports describing the sensitivity of Complex I to TPP^+^ derivatives [79, 80], the conjugate **8a** reduced Complex I‐dependent respiration, whereas the corresponding DPPy^+^ analogue **8b** showed no measurable effect under the same conditions (Figure S35). Overall, DPPy^+^ conjugates exhibited lower intrinsic uncoupling and inhibitory activity on respiration, reduced dissipation of ΔΨm, and lower cytotoxicity when tethered to otherwise inactive cargo, suggesting a potentially improved safety profile relative to TPP^+^ while retaining comparable mitochondrial targeting capacity.

**FIGURE 10:**
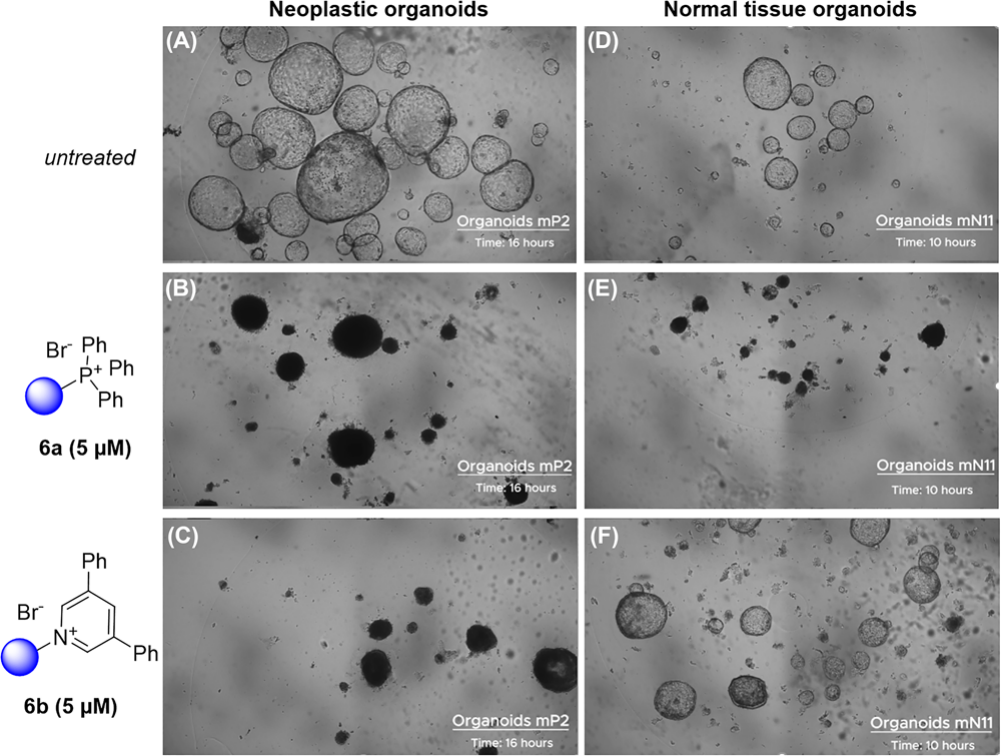
Bright‐field micrographs showing dynamic growth and morphological changes of murine pancreatic intraepithelial neoplasia organoids (mP model, left), and murine pancreatic duct normal tissue organoids (mN model, right), Viable organoids are observed as light objects and dead organoids as dark objects. (A) Untreated neoplastic organoids; (B) neoplastic organoids treated with **6a** (5 µM); (C) neoplastic organoids treated with **6b** (5 µM); (D) Untreated normal tissue organoids; (E) normal tissue organoids treated with **6a** (5 µM); (F) normal tissue organoids treated with **6b** (5 µM). Temporal sequences of microscopy images are presented as videos in the Supporting Information.

Promising activity of **6b** in the COLO‐357 cells prompted further investigation of its cancer selectivity in pancreatic models. As a model non‐tumoral cell line to assess cancer selectivity, we used HPDE, an immortalized human pancreatic ductal epithelial cell line generated by transformation with human papillomavirus 16 (HPV‐16). HPDE lacks typical malignant characteristics, apart from rapid proliferation, and has been shown to be non‐tumorigenic [81]. As a possible basis for selective cytotoxicity, HPDE expresses significantly lower levels of the Kv1.3 potassium channel than COLO‐357 [65]. Additionally, COLO‐357 exhibited a higher mitochondrial membrane potential than HPDE, as determined by the TMRM assay (Figure S36–S37), and showed basal production of reactive oxygen species (ROS), in contrast to HPDE, as determined by the MitoSOX assay (Figure S38). These features may contribute to tumor selectivity as **6a** has previously been shown to specifically kill cells that express higher levels of Kv1.3 and show enhanced ROS levels [11, 82]. Both **6a** and **6b** exhibited selective cytotoxicity toward COLO‐357 compared to HPDE in the SYTOX assay over the 0.625–5 µM concentration range (Figure S39). Moreover, **6b** induced less ΔΨm dissipation than **6a** in both cell lines at 5 µM, with a significantly smaller effect in HPDE than in COLO‐357 (Figure S36). Moving from 2D to 3D cell cultures, tumor spheroids derived from the human pancreatic cancer cell lines COLO‐357 and PANC‐1 were treated with compounds **6a** and **6b**, and cytotoxicity was monitored every 2 h by a fluorescent membrane integrity reporter. After 48 h, both compounds induced dose‐dependent cytotoxicity in COLO‐357 spheroids at 5–10 µM (Figure S40), and significant effects in PANC‐1 spheroids were observed at 10 µM for **6a** and at 25 µM for **6b** (Figure S41). To further assess the efficacy and selectivity of the compounds, we employed murine pancreatic intraepithelial neoplasia (mP) and normal pancreatic duct (mN) organoid models. In untreated and vehicle controls, both neoplastic and normal organoids remained viable throughout the 24 h period (Figure S42). At 5 µM, compounds **6a** and **6b** induced death of neoplastic organoids within 16 h (Figures 10 and S42). In normal tissue organoids, compound **6a** led to complete loss of viability within 10 h, whereas organoids treated with compound **6b** at the same concentration remained predominantly viable. These findings indicate that while both compounds are cytotoxic to neoplastic pancreatic organoids, compound **6b** exhibits improved selectivity compared to **6a** by sparing normal tissue organoids under identical conditions.

The stability of the Kv1.3 inhibitor conjugates **6a** and **6b** was evaluated in mouse (Table S11) and human (Table S12) plasma, where both showed less than 10% degradation after 120 min. In contrast, conjugate **6a** underwent rapid degradation in mouse liver microsomes, with *t*_1/2_ = 7.2 min (Table S15), whereas compound **6b** was more stable, with 79% remaining after 40 min (projected *t*_1/2_ = 130 min) (Table S16). The enhanced microsomal stability of the DPPy^+^ derivative is advantageous for in vivo studies in murine models. The thermodynamic solubility of the TPP^+^‐based compound **6a** and the DPPy^+^‐based compound **6b** was below 1 µM, whereas the bare pyridinium analogue **6c** exhibited dramatically higher solubility (1.6 mM). Due to the modular nature and synthetic availability of pyridines, the substituents on pyridinium‐based MTMs can be tuned for each particular cargo to balance bioactivity and physicochemical properties of the whole conjugate. Optimization of the substituted pyridinium–Kv1.3 inhibitor conjugates towards compounds with in vivo activity will be communicated in due course.

## 3 Conclusion

In conclusion, we identified the N‐tethered 3,5‐diphenylpyridinium (DPPy^+^) cation as an efficient and biocompatible mitochondria‐targeting moiety (MTM). When conjugated to either a bespoke large‐Stokes‐shift fluorescent dye or to a bioactive cargo, DPPy^+^ directed the conjugates predominantly to mitochondria, whereas the corresponding unsubstituted pyridinium analogues accumulated mainly in lysosomes, a property that may be exploited for lysosome‐targeted therapy [83, 84]. Compared with the benchmark triphenylphosphonium (TPP^+^), DPPy^+^ displayed a similar extent of cellular uptake and ΔΨm‐dependent mitochondrial enrichment, as demonstrated by live‐cell imaging and flow‐cytometry of the fluorescent conjugates and by fractionation of cells treated with the bioactive conjugate. Importantly, DPPy^+^ exerted a markedly lower intrinsic impact on mitochondrial function, evidenced by mitochondrial membrane potential, Seahorse, and viability assays using inactive‐cargo derivatives. The DPPy^+^ conjugate of the Kv1.3 inhibitor **6b** selectively induced death of pancreatic adenocarcinoma cells in 2D cultures and in organoids while sparing healthy cells and normal‐tissue organoids, indicating improved biocompatibility relative to the corresponding TPP^+^ analogue **6a**, alongside enhanced mouse microsomal stability. Although **6b** retained the low solubility characteristic of the parent compound **6a**, the synthetic versatility of the pyridinium platform offers opportunities for structural fine‐tuning to optimize physicochemical properties for each specific cargo. Overall, this study positions pyridinium cations as efficient and biocompatible surrogates for triphenylphosphonium in the mitochondria‐targeting toolbox and highlights the large‐Stokes‐shift fluorescent dye Spidye as an accessible molecular tool for the development and evaluation of mitochondria‐targeting moieties.

## Author Contributions

**Ivan Džajić**: conceptualization, investigation, writing – original draft, writing ‐ review and editing, visualization, methodology, data curation. **Natalija Trunkelj**: investigation, writing – review and editing, visualization, validation, methodology, formal analysis, data curation. **Jernej Repas**: investigation, writing – review and editing, visualization, validation, methodology, formal analysis, data curation. **Maša Kandušer**: conceptualization, investigation, writing – review and editing, methodology. **Lara Smrdel**: investigation. **Stane Pajk**: conceptualization, investigation, methodology, data curation, visualization, validation, formal analysis. **Lovro Žiberna**: supervision, investigation. **Irena Mlinarič‐Raščan**: funding acquisition, resources. **Bostjan Markelc**: visualization, investigation, formal analysis, validation, data curation. **Tim Bozic**: investigation, validation, visualization, formal analysis, data curation. **Masa Omerzel**: investigation, validation, visualization, formal analysis, data curation. **Tanja Jesenko**: investigation, validation, visualization, formal analysis, data curation. **Maja Cemazar**: supervision, resources. **Katja Kološa**: investigation, visualization, writing – review and editing, formal analysis, data curation, validation. **Bojana Žegura**: supervision, resources. **Miha Virant**: investigation, visualization, data curation, formal analysis. **Matic Lozinšek**: investigation, validation, supervision, resources. **Hai M. Nguyen**: investigation, visualization, formal analysis, data curation, validation. **Joshua A. Nasburg**: investigation, visualization, formal analysis, data curation, validation. **Heike Wulff**: supervision, resources, writing – review and editing. **Maxime Gueguinou**: investigation, methodology, validation, visualization, formal analysis, data curation, resources. **Valerije Vrček**: formal analysis, investigation, methodology, visualization, data curation. **Veronica Carpanese**: formal analysis, investigation, methodology, visualization, data curation. **Ildiko Szabo**: formal analysis, investigation, methodology, visualization, data curation, writing – review and editing. **Luis A. Pardo**: conceptualization, investigation, writing – review and editing, visualization, methodology, validation, formal analysis, data curation, supervision, resources. **Tihomir Tomašič**: conceptualization, writing – review and editing. **Lucija Peterlin Mašič**: conceptualization, funding acquisition, writing – review and editing, project administration, resources, supervision. **Andrej Emanuel Cotman**: conceptualization, investigation, funding acquisition, writing – original draft, writing – review and editing, methodology, visualization, project administration, supervision, data curation.

## Conflicts of Interest

I.D., N.T., S.P., L.A.P., T.T., L.P.M., and A.E.C. are inventors on pending patent applications related to aspects of the work presented in this article. The remaining authors declare no competing interests.

## Supporting information



The authors have cited additional references within the Supporting Information [85, 86, 87, 88, 89, 90, 91, 92, 93, 94, 95, 96, 97, 98]. **Supporting File 1**: anie72668‐sup‐0001‐SuppMat.pdf.

**Supporting File 2**: anie72668‐sup‐0002‐Supporting Videos.mp4.

**Supporting File 3**: anie72668‐sup‐0003‐Data.zip.

## Data Availability

The data that supports the findings of this study are available in the supplementary material of this article.
