# Supplementary material for: Mitochondria‐Targeting Moieties Based on N‐Tethered Pyridinium Cations
Source: Angew Chem Int Ed Engl. 2026 May 20;65(29):e7158257. doi: 10.1002/anie.7158257 (PMC13360495; doi:10.1002/anie.7158257)
Supplement: Supplementary file 1 — The authors have cited additional references within the Supporting Information [85, 86, 87, 88, 89, 90, 91, 92, 93, 94, 95, 96, 97, 98]. Supporting File 1: anie72668‐sup‐0001‐SuppMat.pdf. [file ANIE-65-e7158257-s001.pdf]

Mitochondria-Targeting Moieties Based on N-Tethered Pyridinium Cations

Ivan Džajić,[a] Natalija Trunkelj,[a] Jernej Repas,[a] Maša Kandušer,[a] Lara Smrdel,[a] Stane Pajk,[a] Lovro Žiberna,[a] Irena Mlinarič-Raščan,[a] Bostjan Markelc,[c,d] Tim Bozic,[c] Masa Omerzel,[c,e] Tanja Jesenko,[c,f] Maja Cemazar,[c,g] Katja Kološa,[h] Bojana Žegura,[h] Miha Virant,[i] Matic Lozinšek,[i] Hai M. Nguyen,[j] Joshua A. Nasburg,[j] Heike Wulff,[j] Maxime Gueguinou,[k] Valerije Vrček,[l] Veronica Carpanese,[m] Ildiko Szabo,[m] Luis A. Pardo,[b] Tihomir Tomašič,[a] Lucija Peterlin Mašič,*[a] and Andrej Emanuel Cotman*[a]

[a] Faculty of Pharmacy, University of Ljubljana, Aškerčeva cesta 7, SI-1000 Ljubljana, Slovenia
*E-mail: lucija.peterlinmasic@ffa.uni-lj.si, andrej.emanuel.cotman@ffa.uni-lj.si

[b] Max Planck Institute for Multidisciplinary Sciences, City Campus, Hermann-Rein-Straße 3, 37075 Göttingen, Germany

[c] Department of Experimental Oncology, Institute of Oncology Ljubljana, Zaloška cesta 2, SI-1000 Ljubljana, Slovenia

[d] Biotechnical Faculty, University of Ljubljana, Jamnikarjeva ulica 101, SI-1000 Ljubljana, Slovenia.

[e] Faculty of Health Sciences, University of Ljubljana, Zdravstvena pot 5, SI-1000 Ljubljana, Slovenia

[f] Faculty of Medicine, University of Ljubljana, Vrazov trg 2, SI-1000 Ljubljana, Slovenia

[g] Faculty of Health Sciences, University of Primorska, Polje 42, SI-6310 Izola, Slovenia

[h] Department of Genetic Toxicology and Cancer Biology, National Institute of Biology, Večna pot 121, SI-1000 Ljubljana, Slovenia

[i] Jožef Stefan Institute, Jamova Cesta 39, SI-1000 Ljubljana, Slovenia

[j] Department of Pharmacology, School of Medicine, University of California, Davis, 451 Health Sciences Drive, Davis, CA 95616

[k] Inserm UMR1069 “Niche Nutrition Cancer and Oxydative Metabolism (N2COx)”, Univeristy of Tours, 10 boulevard Tonnellé, 37032 Tours

[l] V. Vrček, University of Zagreb, Faculty of Pharmacy and Biochemistry, Ante Kovačića 1, Zagreb 10000, Croatia

[m] V. Carpanese, I. Szabo, University of Padova, Department of Biology, Via U. Bassi, 58, Padova 35121, Italy

Supporting Information

Contents

Cell culture conditions 3

Confocal microscopy 3

Live-cell-imaging of fluorescein conjugates 1a and 1b (Figure S1) 4

Mitochondrial mass determination (Figure S2) 5

Live-cell imaging of Spidye conjugates 4a–4f, additional results (Figures S3–S4, Tables S1–S2) 6

Mechanism of lysosomal localization (Figures S5–S13, Tables S3–S4) 8

Resazurin assay (Figure S14) 18

Electrophysiology (Figure S15, Table S5) 22

Cellular uptake of the Kv1.3 inhibitor conjugates by flow cytometry (Figure S16) 23

Determination of mitochondrial localization of 6b by LC-MS (Figures S17–S21, Table S6) 24

Partition coefficient, logD 29

Thermodynamic solubility. 29

Single-crystal X-ray diffraction analysis of 7b (Figures S22–S26, Tables S7–S9) 31

Computational studies (Table S10) 39

Mitochondrial membrane potential (Figures S 27–S28) 43

Mitochondrial permeability transition pore opening (Figure S29) 46

Determination of Caspase 3/7 activity,apoptosis assay (Figure S30) 47

Seahorse Extracellular flux real-time metabolic analysis (Figures S31–S35) 48

Cancer selectivity in pancreatic 2D models (Figures S36–39) 54

3-dimensional cell cultures (Figures S40–42) 56

Stability studies of Compounds 6a and 6b (Tables S11–S16) 60

Synthesis and characterization 69

NMR spectra (Figures S43–S104) 84

HPLC traces (Figures S105–S123) 115

HRMS spectra (Figures S124–S144) 134

References 155

Cell culture conditions

COLO-357 (ECACC, human female pancreatic adenocarcinoma) cells were cultured in complete Advanced RPMI medium containing Advanced RPMI-1640 (Gibco, Thermo Fischer Scientific, Waltham, MA, USA) supplemented with 5 % heat inactivated fetal bovine serum (Gibco, Thermo Fischer Scientific, Waltham, MA, USA), 100 U/mL penicillin and 100 U/mL streptomycin (Sigma-Aldrich, St. Louis, MO, USA), and 1 % GlutaMAX (Gibco, Thermo Fischer Scientific, Waltham, MA, USA). **NHLF** (Lonza, human normal lung fibroblasts) cells were maintained in FGM™-2 Fibroblast Growth Medium-2 BulletKit™ (Lonza, Basel, Switzerland). **B16F10** (ATCC, mouse, Mus musculus breed C57BL/6, male, melanoma), **C2C12** (ATCC, mouse, Mus musculus breed C3H, female, muscle), and **L929** (ECACC, mouse, Mus musculus breed C3H, male, fibroblast-like) cells were cultured in Advanced DMEM supplemented with 5% heat-inactivated FBS, 100 U/mL penicillin, 100 µg/mL streptomycin, and 1% GlutaMAX. **PANC-1** (human, male, Pancreatic ductal adenocarcinoma) cells were cultured in DMEM/F-12 + GlutaMAX (Gibco, Thermo Fischer Scientific, Waltham, MA, USA) supplemented with 10 % heat inactivated fetal bovine serum (Gibco, Thermo Fischer Scientific, Waltham, MA, USA), 100 U/mL penicillin and 100 U/mL streptomycin (Sigma-Aldrich, St. Louis, MO, USA). The cells were incubated at 37 ℃ in a 5 % CO_2 atmosphere. **HPDE** cells were cultured in 50% RPMI + 50% Keratinocyte-SFM (serum-free medium, Gibco) supplemented with 10% FBS, 100 U/ml penicillin G, 0.1 mg/mL streptomycin, 2.5 µg/L human recombinant epidermal growth factor (EGF, Gibco) and 0.025% bovine pituitary extract (BPE, Gibco), freshly added at each passage.

Confocal microscopy

Cells were seeded in 4-well ibidi chamber (iBidi #80426) and allowed to adhere overnight. Cells were first treated with selected compounds **3** and **4a-f** in full growth media in concentration 100 nM. Cells were then washed with PBS (Gibco, Thermo Fischer Scientific, Waltham, MA, USA) three times and incubated with MitoTracker™ Deep Red (Invitrogen™, Thermo Fischer Scientific, Waltham, MA, USA) in concentration 50 nM and then washed again with PBS three times. Live cell imaging was performed in LCIS (140 mM NaCl, 2.5 mM KCl, 1.8 mM $CaCl_2$, 1 mM $MgCl_2$, 20 mM Hepes, 20 mM Glucose Confocal imaging was performed using two systems: a Nikon Ti2 AX confocal microscope equipped with four lasers (405, 488, 561, and 638 nm) and a Zeiss LSM 880 equipped with six lasers (405, 458, 488, 514, 561, 639 nm). The Zeiss system features multiple detectors, including three fluorescence PMTs, a spectral detector, and an Airyscan Fast detector, with imaging conducted using a 40×/1.30 NA objective. It also includes an environmental chamber maintaining 37 °C and 90% humidity. The Nikon AX, integrated with a Nikon Ti2 platform, was used with a 60× oil immersion objective lens (NA 1.42).

Colocalization analysis was performed using the Just Another Colocalization Plugin (JaCoP) in Fiji,[8] employing both the original JaCoP implementation and the BIOP-revamped version.[1.] Background subtraction was applied prior to analysis to reduce noise. Thresholds were either set manually based on image histograms or determined automatically using Costes' method within JaCoP to exclude

[1] BIOP. JACoP – Just Another Co-localization Plugin (revamped). GitHub repository. Available from: https://github.com/BIOP/ijp-jacop-b. Accessed 2025-11-25.

background pixels. Cytofluorograms were generated with the Colocalization Finder[2] to visualize pixel intensity correlations between fluorescence channels. All analyses were performed on at least three representative images per condition to ensure reproducibility. The Manders' coefficients M1 and M2 represent the following: M1 = fraction of A overlapping B, and M2 = Fraction of B overlapping A, wherein the channel A was used for Mitotracker Deep red detection (excitation λ = 640 nm; emission λ = 649–747 nm), and the channel B was used for detection of compounds **4a-f** (excitation λ = 405 nm; emission λ = 550–600 nm)

Live-cell-imaging of fluorescein conjugates 1a and 1b (Figure S1)

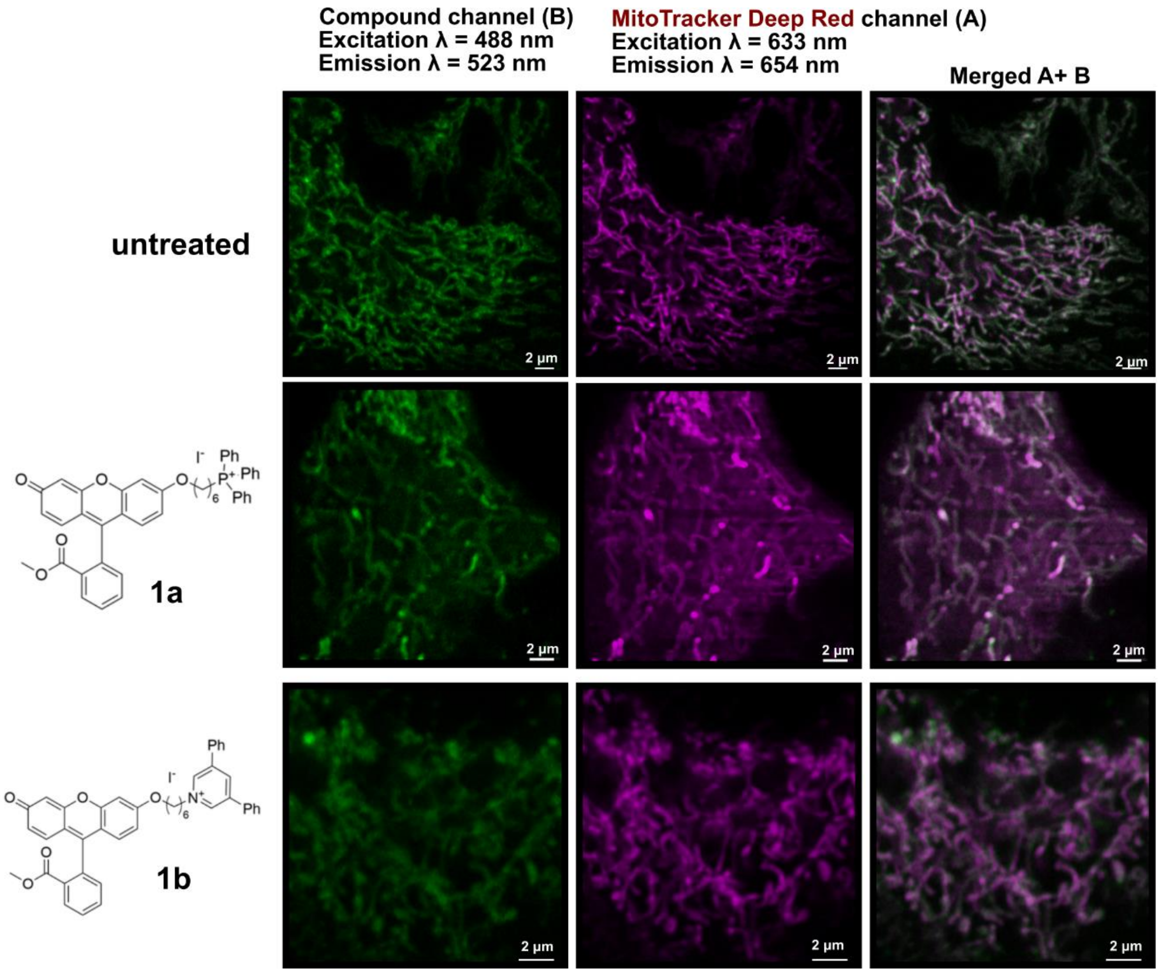

Figure S 1. Preliminary live-cell imaging study of mitochondrial localization of the fluorescein conjugates **1a** and **1b** (100 nM) **in COLO-357 cells**. MitoTracker Deep Red was used as reference organelle-specific stain (50 nM). Significant cell autofluorescence was observed in the fluorescein channel of the untreated cells.

[2] C. Laummonerie, J. Mutterer, P. Carl, *Colocalization Finder* plugin for ImageJ, CNRS UMR7213, Strasbourg, France, 2004–2021

Mitochondrial mass determination (Figure S2)

For mitochondrial mass determination, the following cell lines were used: COLO-357, PANC-1, C2C12, B16F10 and HeLa. Among the human cell lines, COLO-357 was found to have the highest mitochondrial mass, whereas PANC-1 and NHLF appear to be less rich in mitochondria. This is in contrast to the note of "few mitochondria" in the original publication describing the COLO-357 cell line.[15]

Cells were seeded 48 hours prior to the experiment in their appropriate culture media to reach optimal confluency. Cells were detached using Gibco™ Trypsin-EDTA (0.25%), phenol red (Thermo Fisher Scientific, Cat. No. 25200072). The duration of trypsinization was adjusted according to the specific cell line to ensure gentle detachment without compromising cell viability. After detachment, cells were centrifuged at 1300 rpm for 5 minutes at room temperature in their respective culture media. The cell pellet was resuspended, and cells were counted. For each cell line, 100,000 cells were transferred into 2.0 mL Eppendorf tubes for staining.

To evaluate mitochondrial mass, cells were incubated with nonyl acridine orange, NAO,[16] (3,6-bis(dimethylamino)-10-nonylacridin-10-ium bromide) at 6.0 µM for 20 min under standard culture conditions (37 °C, 5% CO_2). After staining, cells were immediately placed on ice. Samples were centrifuged at 230 × *g* for 5 min at 4 °C. The supernatant was carefully removed, and cells were washed once with ice-cold PBS to remove excess dye. Cells were centrifuged again under the same conditions, resuspended in 400 µL ice-cold PBS, and kept on ice until flow cytometry analysis.

Flow cytometric measurements were performed on an Attune NxT flow cytometer. NAO fluorescence was detected using the 488 nm laser for excitation and a 530/30 nm emission filter. Unstained controls for each cell line were included to set gates and control for autofluorescence. Data were exported and analyzed using Microsoft Excel and Graph Pad Prism 10.3.1. for quantification of median fluorescence intensity, representing relative mitochondrial mass.

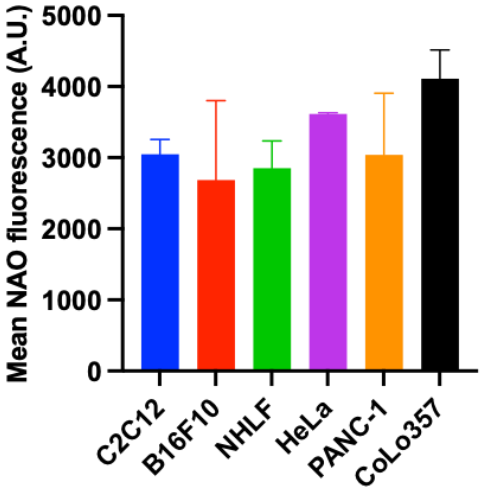

Figure S 2. Mitochondrial mass was assessed by measuring the mean fluorescence intensity of Nonyl Acridine Orange (NAO) with flow cytometry, a lipophilic fluorescent dye that selectively binds to cardiolipin-rich mitochondrial membranes. NAO accumulation correlates with mitochondrial membrane surface area and cardiolipin content, providing a reliable indicator of mitochondrial mass largely independent of mitochondrial membrane potential. Data are presented as mean ± SEM and represent two independent biological replicates.

Live-cell imaging of Spidye conjugates 4a–4f, additional results (Figures S3–S4, Tables S1–S2)

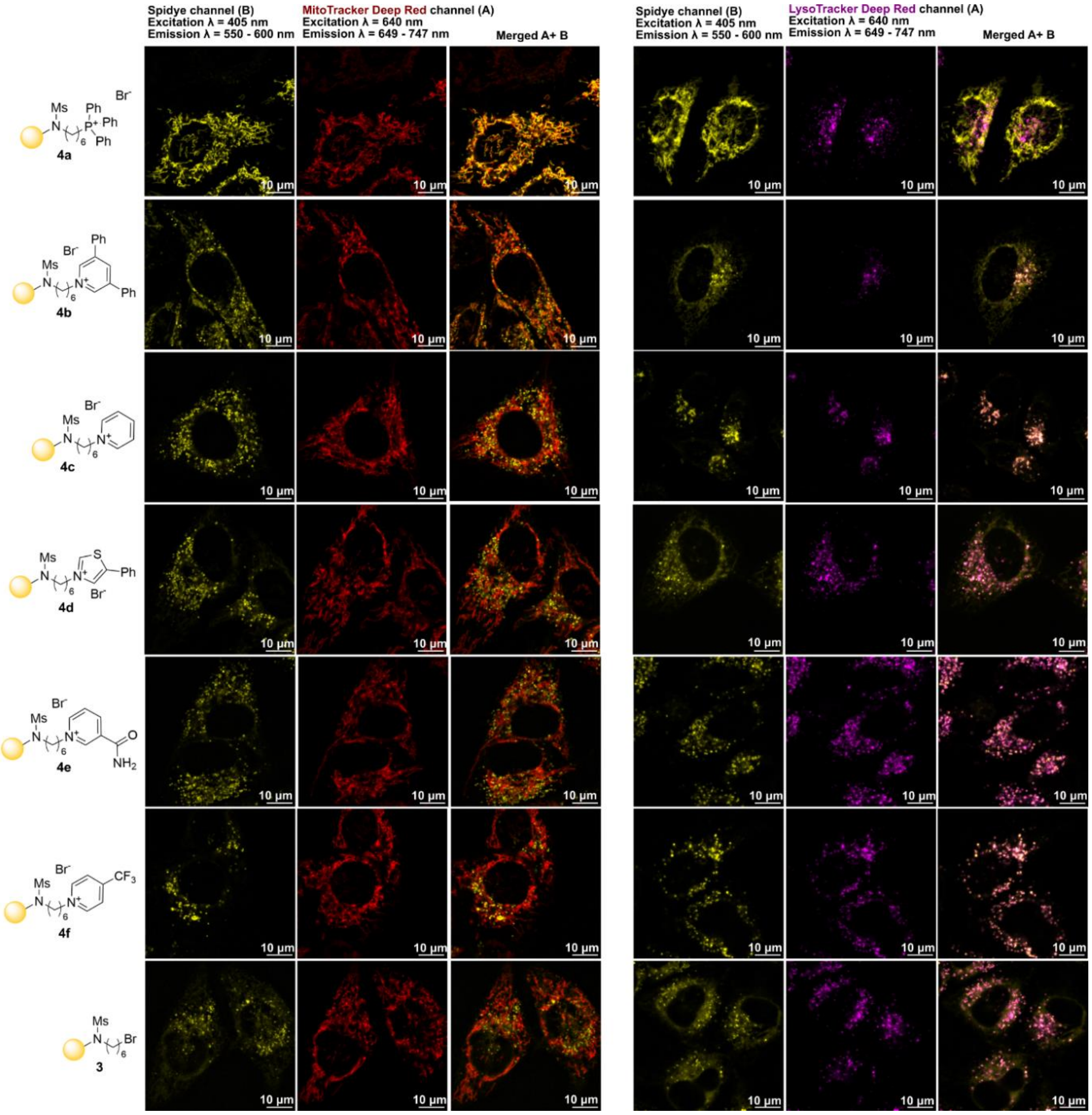

Figure S 3. Confocal microscopy-based study of mitochondrial and lysosomal localization of SpyDye conjugates **3**–**4** (100 nM) **in HeLa cells**. Mitotracker Deep Red and Lysotracker Deep Red were used as reference organelle-specific stains (50 nM). Microscopy images are representative of at least two biological replicates.

Table S 1. Quantification of live-cell (**HeLa**) images shown in Figure S3

Cpd	LogD[a] (pH 7.4)	Pearson[b] (MitoTracker)	M1[c] (MitoTracker)	M2[d] (MitoTracker)	Pearson[b] (LysoTracker)	M1[c] (LysoTracker)	M2[d] (LysoTracker)
**4a**	2.99	0.91 ± 0.01	0.84 ± 0.02	0.89 ± 0.02	0.41 ± 0.05	0.78 ± 0.14	0.17 ± 0.03
**4b**	3.08	0.73 ± 0.06	0.73 ± 0.11	0.66 ± 0.07	0.77 ± 0.14	0.93 ± 0.04	0.48 ± 0.22
**4c**	−0.08	0.38 ± 0.01	0.11 ± 0.03	0.30 ± 0.05	0.96 ± 0.01	0.93 ± 0.02	0.89 ± 0.01
**4d**	3.52	0.25 ± 0.03	0.11 ± 0.04	0.25 ± 0.02	0.90 ± 0.06	0.91 ± 0.06	0.78 ± 0.20
**4e**	0.35	0.44 ± 0.07	0.26 ± 0.13	0.47 ± 0.08	0.89 ± 0.04	0.78 ± 0.03	0.88 ± 0.07
**4f**	1.28	0.25 ± 0.04	0.076±0.005	0.30 ± 0.06	0.93 ± 0.01	0.83 ± 0.02	0.92 ±0.03
**3**	>6	0.43 ± 0.04	0.14 ± 0.07	0.36 ± 0.12	0.89 ± 0.02	0.91 ±0.05	0.79 ± 0.11

[a] Experimentally determined octanol/phosphate-buffered saline (pH 7.4) distribution coefficient. [b] linear correlation of fluorescence intensities in A and B across pixels. [c] fraction of pixels in channel A overlapping with positive pixels in channel B. [d] fraction of positive pixels in channel B overlapping with positive pixels in channel A. Data in [a–d] represent mean ± SEM of at least 3 images.

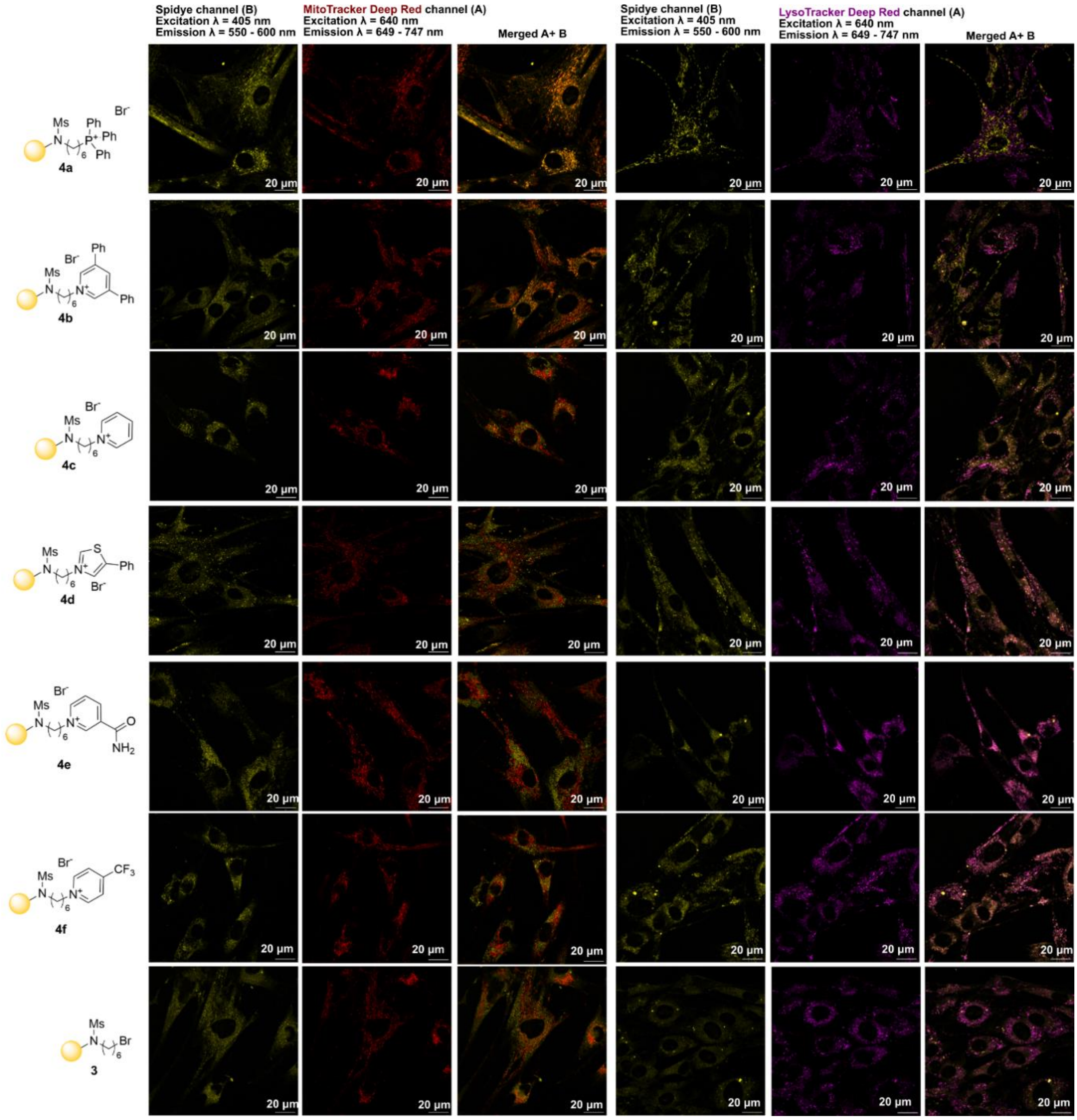

Figure S 4. Confocal microscopy-based study of mitochondrial and lysosomal localization of SpyDye conjugates **3**–**4** (100 nM) **in NHLF cells**. Mitotracker Deep Red and Lysotracker Deep Red were used as reference organelle-specific stains (50 nM). Microscopy images are representative of at least two biological replicates.

Table S 2. Quantification of live-cell (**NHLF**) images shown in Figure S4

Cpd	LogD[a] (pH 7.4)	Pearson[b] (MitoTracker)	M1[c] (MitoTracker)	M2[d] (MitoTracker)	Pearson[b] (LysoTracker)	M1[c] (LysoTracker)	M2[d] (LysoTracker)
**4a**	2.99	0.87 ± 0.02	0.85 ± 0.06	0.82 ± 0.05	0.06 ± 0.05	0.08 ± 0.04	0.05 ± 0.03
**4b**	3.08	0.78 ± 0.05	0.88 ± 0.05	0.57 ± 0.10	0.32 ± 0.04	0.36 ± 0.04	0.14 ± 0.03
**4c**	−0.08	0.14 ± 0.06	0.16 ± 0.07	0.10 ± 0.05	0.73 ± 0.06	0.92 ± 0.04	0.37± 0.09
**4d**	3.52	0.19 ± 0.04	0.15 ± 0.14	0.17 ± 0.04	0.89 ± 0.04	0.87 ± 0.04	0.77 ± 0.12
**4e**	0.35	0.08 ± 0.06	0.12 ± 0.06	0.06 ± 0.03	0.85 ± 0.05	0.82 ± 0.10	0.82 ± 0.06
**4f**	1.28	0.29 ± 0.08	0.18 ± 0.13	0.22 ± 0.14	0.82 ± 0.04	0.91 ± 0.05	0.50 ± 0.15
**3**	>6	0.41 ± 0.06	0.35 ± 0.11	0.33 ± 0.02	0.62 ± 0.10	0.56 ± 0.43	0.54 ± 0.25

[a] Experimentally determined octanol/phosphate-buffered saline (pH 7.4) distribution coefficient. [b] linear correlation of fluorescence intensities in A and B across pixels. [c] fraction of pixels in channel A overlapping with positive pixels in channel B. [d] fraction of positive pixels in channel B overlapping with positive pixels in channel A. Data in [a–d] represent mean ± SEM of at least 3 images.

Mechanism of lysosomal localization (Figures S5–S13, Tables S3–S4)

Lysosomal pH-dependent lysosomal uptake.

For lysosomal pH measurements, COLO-357 cells were in 8-well ibidi chamber (iBidi #80826) at a density of 2.5 × 10^4 cells per well or in 4-well ibidi chamber (iBidi #80426) at a density of 5 × 10^4 cells per well and allowed to adhere overnight before dye loading. To label lysosomes, cells were incubated for 24 h in complete medium containing FITC-dextran (0.125 mg/mL) and rhodamine-dextran (0.0625 mg/mL), allowing uptake of the probes by endocytosis. After the loading period, cells were washed and incubated for 3 h in dye-free medium to permit trafficking and sequestration of the dextran-conjugated probes within lysosomes. Where indicated, bafilomycin A1 (100 nM) was applied during the 3-hour chase period.[1] For compound treatment experiments, cells were incubated with compound **4c** (100 nM) for 10 minutes either before bafilomycin A1 addition (compound pre-treatment) or after bafilomycin A1 addition (compound post-treatment), followed by three PBS washes to remove residual compound.

To validate the lysosomal neutralization protocol in COLO-357 cells, we used two dextran probes: FITC-dextran, whose fluorescence intensity is pH-dependent, and Rhodamine B-dextran, whose fluorescence is largely pH-independent. The ratio of the two signals provides a reliable indicator of lysosomal pH changes.[1] Indeed, increase in FITC-dextran fluorescence relative to Rhodamine B-dextran was observed upon 3 h treatment with Bafilomycin A1 (100 nM), validating the protocol of lysosomal neutralization (Figure S5). For the subsequent colocalization experiments with the Spidye conjugate **4c** (Figure S6), only FITC-dextran was used because Rhodamine B exhibits strong absorption in the Spidye emission range, which interferes with accurate analysis of Spidye fluorescence.

All acquisitions were performed in live-cell imaging solution containing 140 mM NaCl, 2.5 mM KCl, 1.8 mM $CaCl_2$, 1 mM $MgCl_2$, 20 mM HEPES, and 20 mM glucose. Live-cell imaging was carried out on a Nikon Ti2 AX confocal microscope equipped with a 60×/1.42 NA Plan Apochromat oil-immersion objective. FITC-dextran was excited using a 488 nm laser, and fluorescence emission was collected between 507 and 525 nm. Rhodamine-dextran was excited using a 561 nm laser, and fluorescence emission was collected between 585 and 625 nm. Compound **4c** was excited using a 405 nm laser, and the corresponding emission was collected between 550 and 600 nm. At least 10 fields of view were acquired per chamber. Identical microscope acquisition settings were maintained across all experimental conditions.

Lysosomal uptake quantification

All wells were imaged under identical acquisition settings. Image analysis was performed using Fiji.[2] Fluorescence measurment was performed in the compound fluorescence channel (λ_{ex} = 405 nm, λ_{em} = 550–600 nm) under all experimental conditions.

Lysosomal ROIs were defined using a condition-dependent segmentation strategy. In Bafilomycin A1-treated conditions, inhibition of vacuolar-type H^+-ATPase results in lysosomal alkalization,[3] leading to an increase in FITC-dextran fluorescence intensity, as the fluorescence spectrum of FITC-dextran is pH-dependent, with fluorescence increasing upon alkalization of the lysosomal lumen.[4] Under these conditions, lysosomes became visible in the FITC-dextran channel (λ_{ex} = 488 nm, λ_{em} = 507–525 nm), and ROIs were defined from this channel. In the absence of Bafilomycin A1, FITC-dextran fluorescence

is quenched under the acidic lysosomal environment and cannot serve as a reliable segmentation reference. ROIs were therefore defined from the compound fluorescence channel, which had been previously validated by colocalization analysis with LysoTracker Deep Red to accumulate within lysosomal compartments (Figure 3). In both cases, ROIs were stored in the ROI Manager and applied to the compound fluorescence channel for fluorescence quantification without modification.

Individual lysosomal objects were identified using the Analyze Particles function (size filter: 0.3–∞ μm^2; circularity: 0.00–1.00; objects intersecting image edges were excluded). A fixed intensity threshold was determined on representative images of the segmentation channel and applied uniformly across all images of the respective condition. For each detected object, background fluorescence was estimated from a cell-free region of the same image and subtracted proportionally to the object area: $IntDen_{corr}$ = IntDen − (Area × Mean_background), integrated fluorescence intensity within the ROI (product of mean pixel intensity and ROI area expressed in μm^2), Area denotes the ROI surface area (μm^2), and Mean_background is the mean pixel intensity measured in a cell-free reference region of the same image. The sum of background-corrected integrated density values across all detected objects per image ($\Sigma IntDen_{corr}$) was used as a single quantitative value per field of view, reflecting the total compound fluorescence retained within lysosomal compartments.

All quantitative measurements were performed on raw, unprocessed images (12-bit depth, stored in 16-bit format). Five independent fields of view per condition were quantified per biological replicate, and experiments were performed in two biological replicates. Statistical analysis was performed in GraphPad Prism (version 10.3.1., GraphPad Software, San Diego, CA, USA). Data were tested for normality using the Shapiro–Wilk test; as not all groups met this assumption, a non-parametric Kruskal–Wallis test followed by Dunn's post-hoc test for multiple comparisons was applied. Data are presented as median ± IQR; each data point represents $\Sigma IntDen_{corr}$ per field of view (n = 5 fields per condition per biological replicate). Differences were considered statistically significant at $p < 0.05$ (Figure S6).

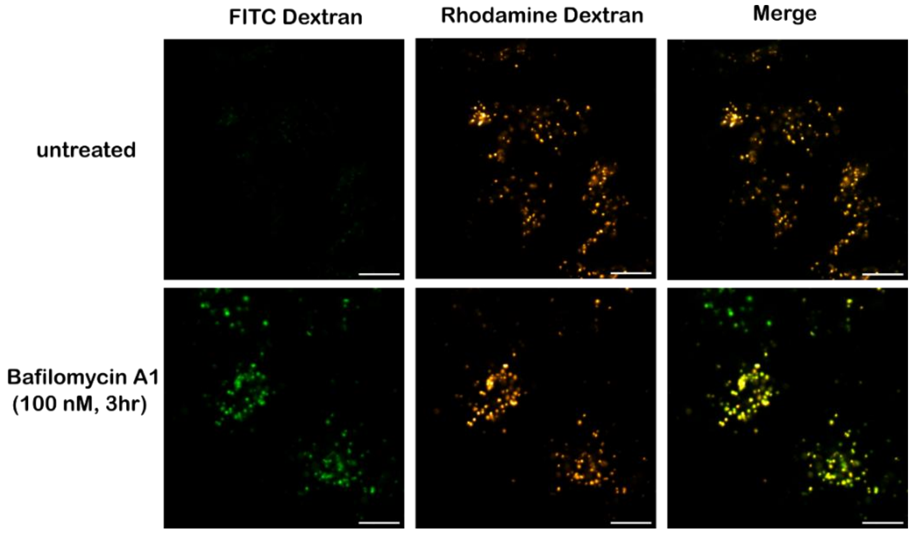

Figure S 5. Representative confocal fluorescence microscopy images of COLO-357 cells loaded with FITC dextran and Rhodamine dextran for ratiomeric lysosomal pH assessment. Cells were incubated with FITC dextran (0.125 mg/mL, 24 h) and Rhodamine dextran (0.0625 mg/mL, 24 h). Where indicated, bafilomycin A1 (BafA1; 100 nM, 3 h) was applied during the chase period. FITC-dextran: λ_{ex} = 488 nm, λ_{em} = 507 – 525 nm and Rhodamine dextran: λ_{ex} = 561 nm, λ_{em} = 585 – 625 nm. Images were acquired on Nikon Ti2 AX confocal microscope. Scale bars, 10 µm.

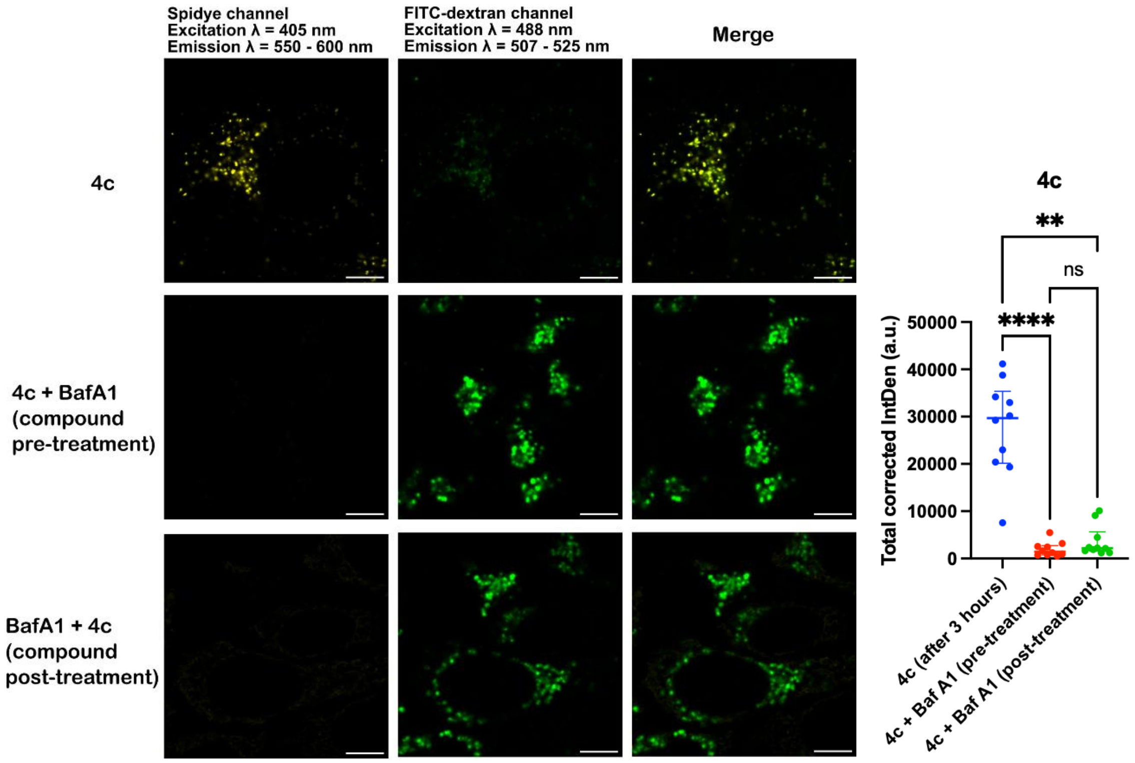

Figure S 6. Left: Representative confocal fluorescence microscopy images of COLO-357 cells loaded with FITC-dextran and treated with compound 4c. Cells were incubated with FITC-dextran (0.125 mg/mL) for 24 h to allow endocytic uptake, followed by a 3 h chase in probe-free medium. Where indicated, bafilomycin A1 (BafA1; 100 nM, 3 h) was applied during the chase period, and compound **4c** (100 nM, 10 min) was added either prior to (pre-treatment) or following (post-treatment) BafA1 addition. Spidye channel: λ_{ex} = 405 nm, λ_{em} = 550–600 nm, and FITC-dextran channel: λ_{ex} = 488 nm, λ_{em} = 507–525 nm. Images were acquired on a Nikon Ti2 AX confocal microscope. Scale bars, 10 µm. Images are representative of two independent biological replicates. **Right: Effect of Bafilomycin A1 on lysosomal accumulation of compound 4c.** Total corrected integrated density (ΣIntDen_corr) of compound **4c** fluorescence within lysosomal compartments. Data are presented as median± IQR; n = 10 (5 fields of view from 2 biological replicates). Kruskal–Wallis test with Dunn's post-hoc test. **p < 0.01, ****p < 0.0001; ns = not significant.

Study of potential pyridinium redox cycling in COLO-357 cells.

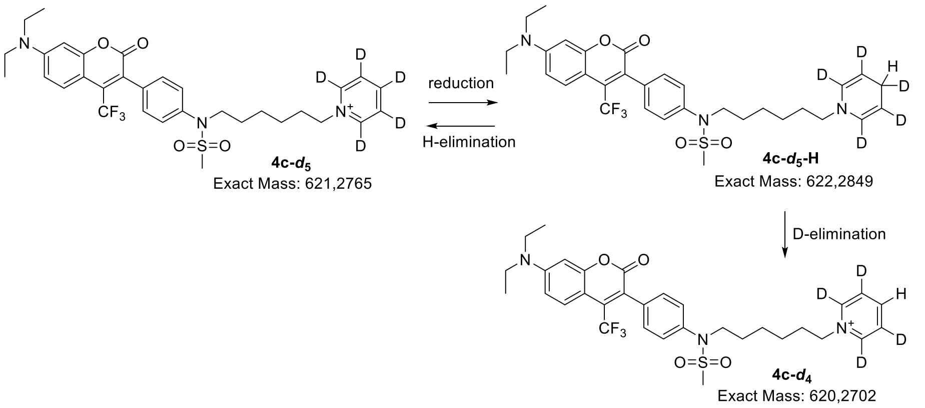

Figure S 7. The hypothesized redox cycling of N-tethered pyridinium cation in live cells, inspired by the NAD+/NADH redox pair. The reduced species **4c-*d*$_5$–H** would be weakly basic and thus prone to ion-trapping in the acidic lysosomal lumen.

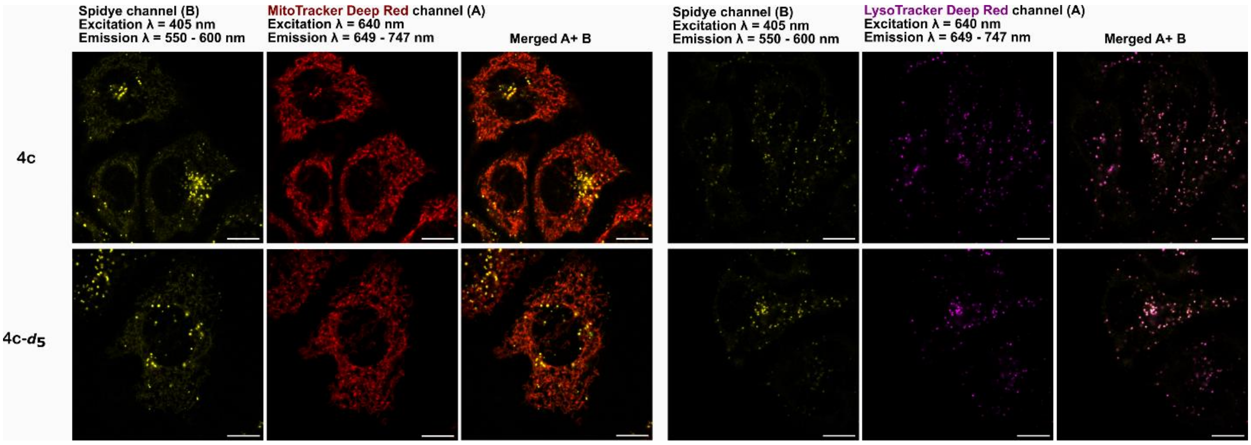

Figure S 8. Representative confocal fluorescence microscopy images of COLO-357 co-stained with 4c or 4c-d$_5$ and MitoTracker Deep Red or Lyso Tracker Deep Red. Cells were costained with **4c** or **4c-*d*$_5$** (100 nM, 10 min incubation) and (A) MitoTracker Deep Red or (B) LysoTracker Deep Red (50 nM, 10 min incubation). Spidye channel: λ_{ex} = 405 nm, λ_{em} = 550 – 600 nm; MitoTracker Deep Red and LysoTracker Deep Red: λ_{ex} = 640 nm, λ_{em} = 649 – 747 nm. Images were obtained with NikonTi2 AX confocal microscope. Scale bars, 10 µm. Images are representative of two independent biological replicates.

Table S 3. Colocalization analysis of **4c** and the deuterated probe **4c-*d*$_5$**.

Cpd	Pearson[a] (MitoTracker)	M1[b] (MitoTracker)	M2[c] (MitoTracker)	Pearson[a] (LysoTracker)	M1[b] (LysoTracker)	M2[c] (LysoTracker)
**4c**	0.52 ± 0.10	0.09 ± 0.06	0.76 ± 0.12	0.87 ± 0.03	0.82 ± 0.07	0.82 ± 0.10
**4c-*d*$_5$**	0.38 ± 0.04	0.06 ± 0.02	0.67 ± 0.14	0.88 ± 0.05	0.85 ± 0.04	0.84 ± 0.05

[a] linear correlation of fluorescence intensities in A and B across pixels. [b] fraction of pixels in channel A overlapping with positive pixels in channel B. [c] fraction of positive pixels in channel B overlapping with positive pixels in channel A. Data in [a–c] represent mean ± SEM of at least 10 images from two independent biological replicates.

LC-MS analysis of 4c-d$_5$ after 1-hour and 24-hour exposure to COLO-357 cells.

COLO-357 cells were seeded in 6 well plate at a density 250 × 10^3 in the well. After 48 hours the cell culture media was discarded and replaced with medium containing **4c-*d*$_5$** (100 nM, 1 µM or 5 µM) for 1 h or **4c-*d*$_5$** (100 nM or 1 µM) for 24 h at 37 °C. Following treatment, cells were detached, washed with PBS and resuspended in 1 mL of ice-cold PBS. The suspension was centrifuged, 4 °C; this wash step was repeated twice. The cell pellet was then resuspended in RIPA buffer supplemented with phosphatase and protease inhibitor cocktail. The lysate was sonicated, shaken for 30 min, centrifuged

at 15,000 g to extract the protein into the supernatant to get the whole cell lysate. The samples were stored at −80 °C until further use.

To 50 µL of the whole cell lysate, 200 µL of acetonitrile was added to precipitate proteins. The mixture was vortexed thoroughly and centrifuged at 18,000 × g. A 200 µL aliquot of the resulting supernatant was transferred into HPLC vials for LCMS analysis. The **4c-*d*$_5$** standard was diluted in acetonitrile from DMSO stock solution.

Chromatographic separation was performed on an Ultimate 3000 (Thermo Fisher Scientific) equipped with an Acquity UPLC® BEH C8 column (50 mm × 2.1 mm, 1.7 µm; Waters). The column temperature was maintained at 40 °C, and the injection volume was 10 µL. The mobile phase consisted of water/MeCN/formic acid (950:5:1, v/v/v) as solvent A and water/MeCN/formic acid (50:950:1, v/v/v) as solvent B. The gradient program was as follows: 0–5.0 min, 5–100% B; 5.0–6.0 min, 100% B; 6.0–6.1 min, 100–5% B; 6.1–8.0 min, 5% B; for re-equilibration. The flow rate was kept constant at 0.50 mL/min throughout the analysis, and from 1.1 to 6.9 min the eluate was directed to the mass spectrometer (compound **4c-*d*$_5$** eluted at 3.43 min). The autosampler temperature was maintained at 15 °C. HRMS analysis was carried out using a QExative plus mass spectrometer (Thermo Fisher Scientific) equipped with a heated electrospray ionization (H-ESI) source operated in positive ionization mode. Full MS spectrum was recorded in a range between 600 and 650 m/z with resolution set at 70000.

To investigate whether **4c-*d*$_5$** undergoes reduction of the pyridinium moiety and/or H/D exchange in COLO-357 cells, whole-cell lysates obtained after compound exposure were analyzed by HPLC-HRMS under the conditions described above. The analysis was focused on three species: the parent deuterated probe **4c-*d*$_5$**, the putative reduced species **4c-*d*$_5$–H**, and the partially deuterium/proton-exchanged species **4c-*d*$_4$**. To assess the possible formation of the reduced pyridinium species **4c-*d*$_5$–H**, the HRMS data were screened for the expected *m/z* value of the protonated reduced form, *m/z* = 623.2922 $[M + H]^+$, across the entire chromatographic run. No signal attributable to the reduced species was detected above background in any of the analyzed lysates (Figure S11).

To evaluate the extent of deuterium/protium exchange, extracted ion chromatograms were generated for the ions corresponding to the parent deuterated compound and the mono-exchanged analogue, i.e. *m/z* 621.2765 for **4c-*d*$_5$** and *m/z* 620.2702 for **4c-*d*$_4$**. The relative abundance of these ions was determined from the corresponding extracted ion chromatographic peak areas recorded for the standard sample and for the cell lysate samples following 1 h and 24 h incubation. Comparison of the **4c-*d*$_4$/4c-*d*$_5$** signal ratio between the standard and cell lysates was used to assess whether exposure to cells led to deuterium/proton exchange (Figure S12). No statistically significant differences in the fraction of **4c-*d*$_4$** were observed between the standard and cell lysate samples, indicating that no detectable H/D exchange occurred under the tested conditions in live cells.

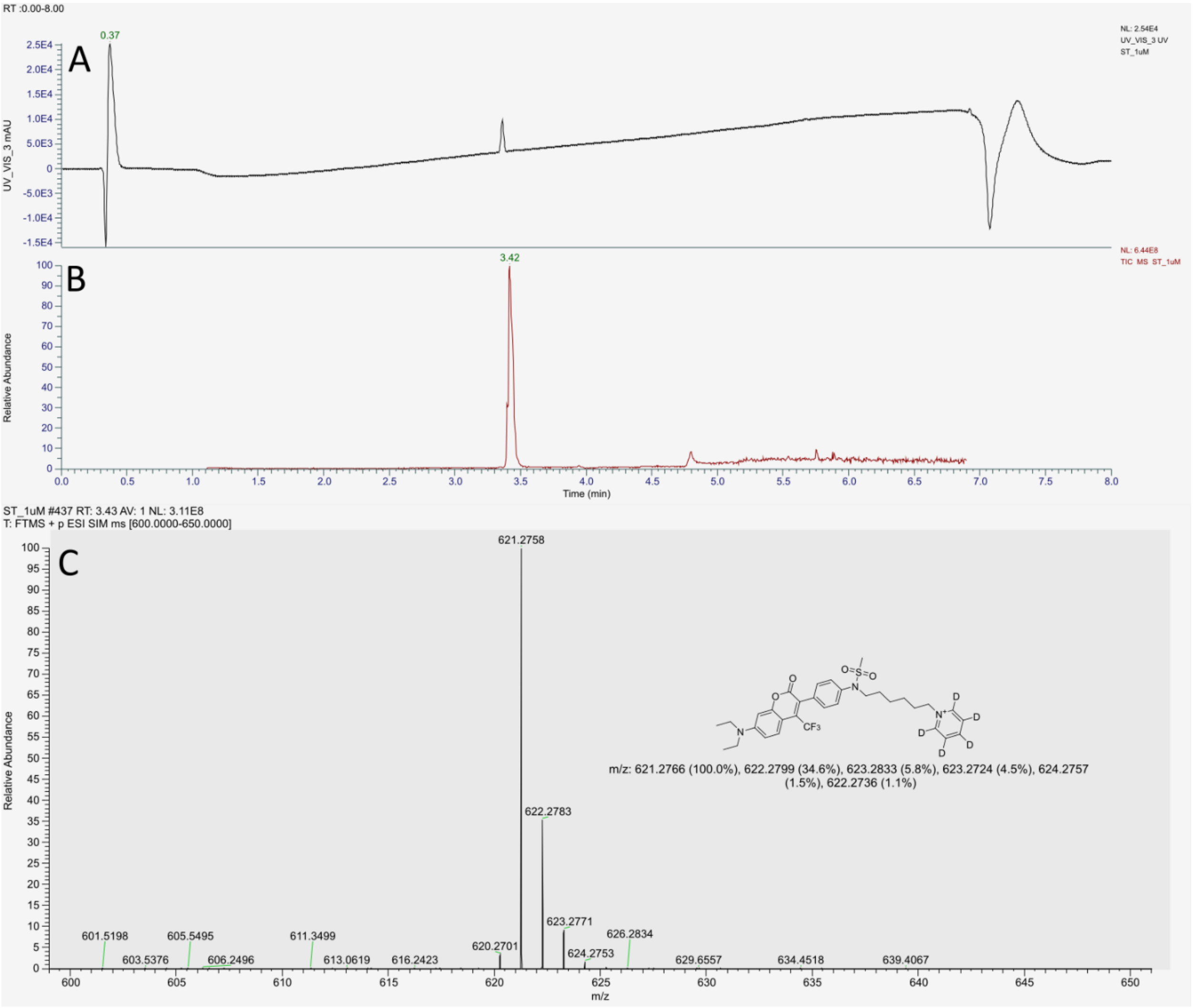

Figure S 9. (A) UV–Vis chromatogram of standard **4c-*d*$_5$**, recorded at 255 nm; (B) total ion chromatogram (TIC); and (C) mass spectrum obtained at a retention time of 3.43 min.

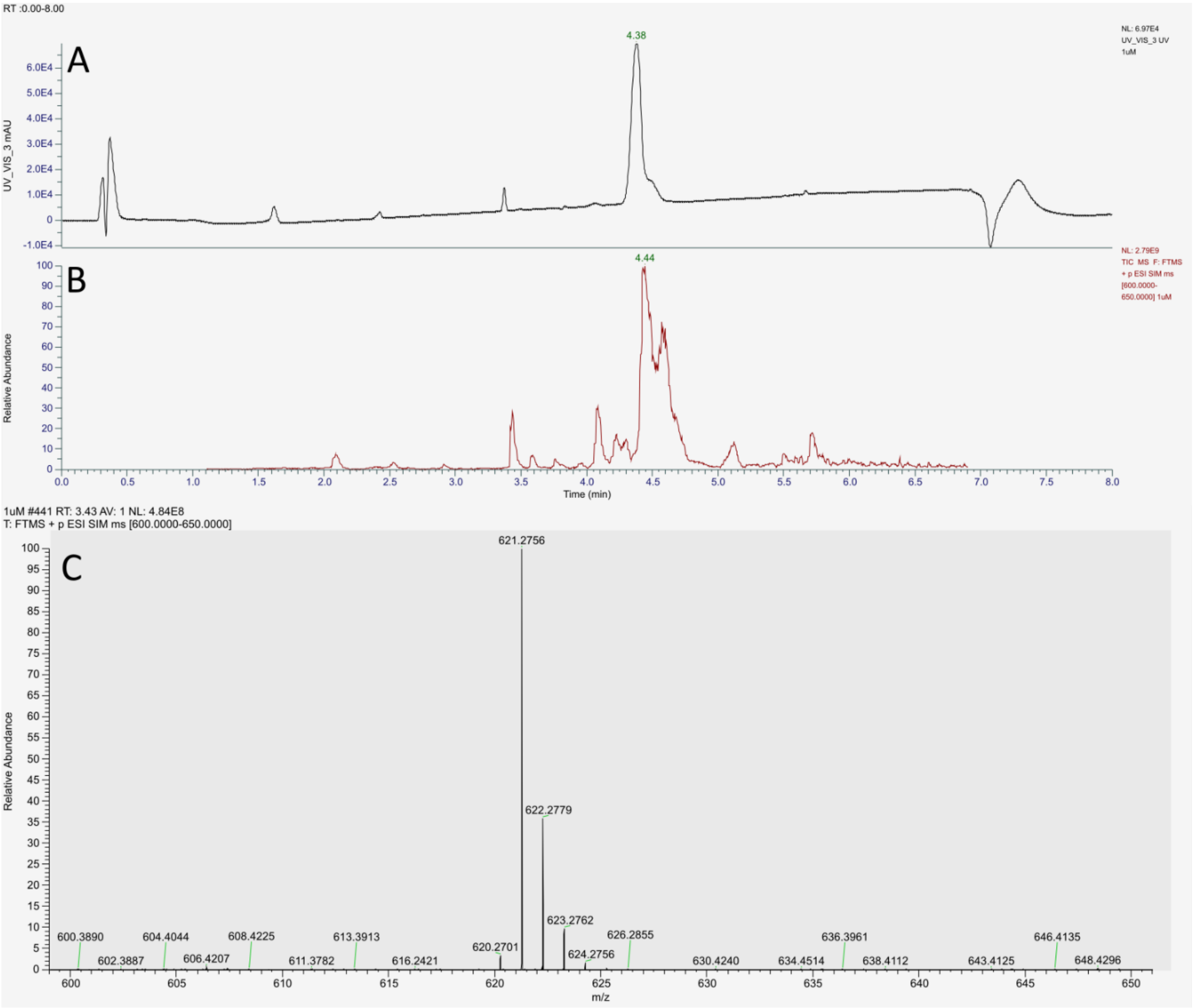

Figure S 10. (A) Representative UV–Vis chromatogram of cell lysate, 24 h incubation with **4c-*d*$_5$** (1 µM), recorded at 255 nm. (B) total ion chromatogram (TIC); and (C) mass spectrum obtained at a retention time of 3.43 min.

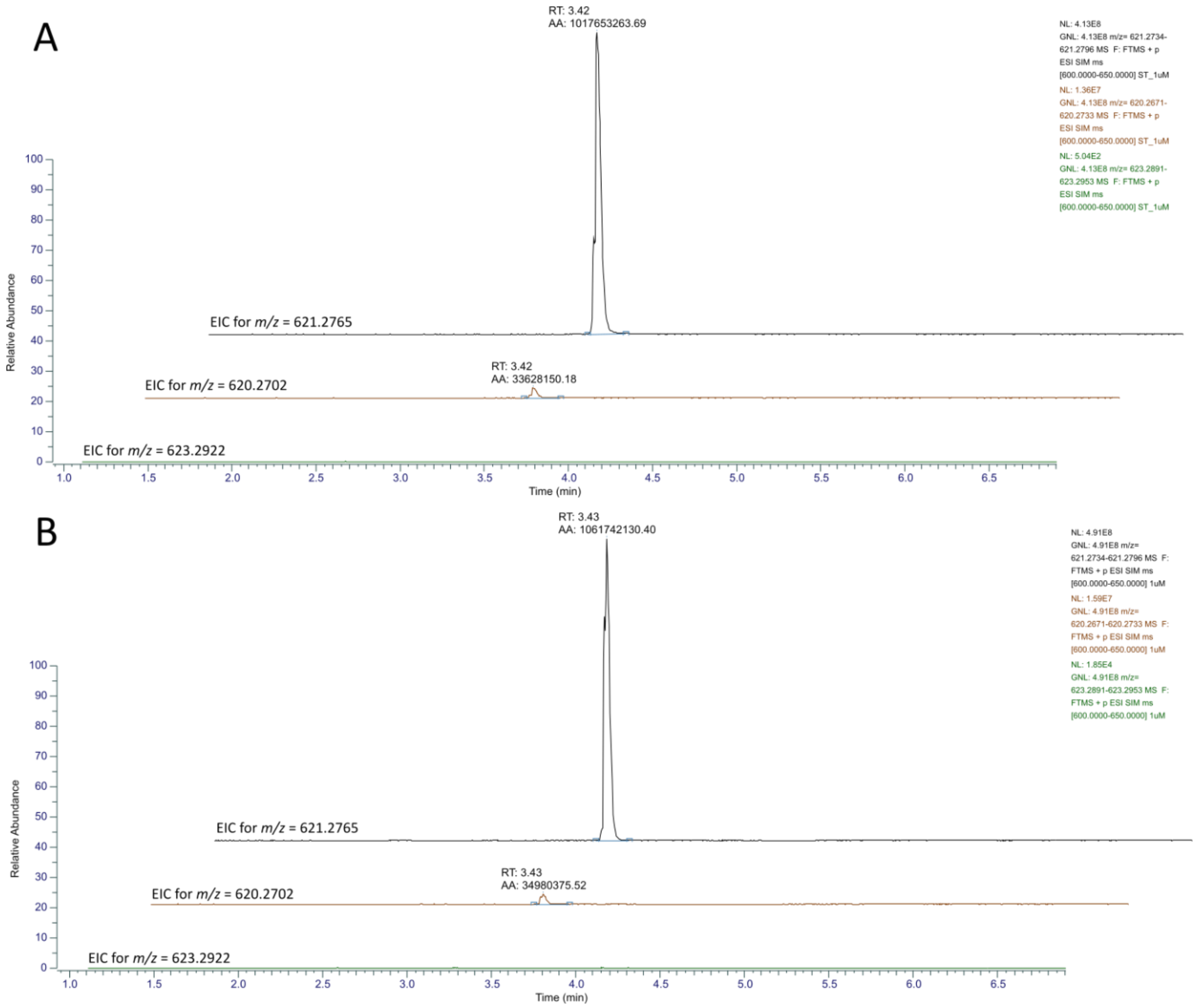

Figure S 11. Extracted ion chromatograms (EIC) of standard (A) and cell lysate sample after 24 h of incubation (B). Calculated *m/z* values for $[M+H]^+$ ions of **4c-*d*$_5$**, **4c-*d*$_4$** and **4c-*d*$_5$-H** are 621.2765, 620.2702 and 623.2922, respectively.

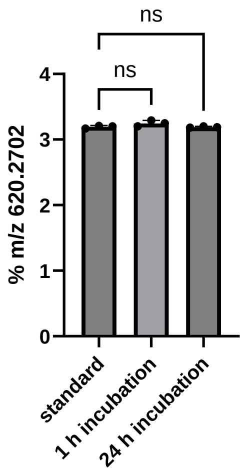

Figure S 12. Fraction of **4c-*d*$_4$**, *m/z* = 620.2702, relative to **4c-*d*$_5$**, *m/z* = 621.2765, in standard sample (**4c-*d*$_5$** diluted from DMSO stock, analyzed in triplicate) and in cell lysates following independent 1 h and 24 h incubations with **4c-*d*$_5$** in live COLO-357 cells. For the 1 h incubation, concentrations of 0.1, 1, and 5 µM were used; for the 24 h incubation, concentrations of 0.1 (duplicate) and 1 µM were used. No statistically significant differences in the fraction of **4c-*d*$_4$** were observed between the standard and cell lysate samples. Statistical analysis was performed using one-way ANOVA followed by Dunnett's multiple comparisons test.

Mitochondrial membrane potential-dependent cellular intracellular distribution by live cell imaging

To assess whether the intracellular distribution of the N-tethered pyridinium conjugates with fluorescent dye depended on mitochondrial membrane potential ($\Delta\psi m$), live-cell imaging experiments were performed in the presence or absence of carbonyl cyanide *p*-(trifluoromethoxy)phenylhydrazone (FCCP).

COLO-357 cells were seeded 24 h prior to experiment in 4-well ibidi chamber (iBidi #80426) at a density 1 × 10^5 cells per well. Cells were first incubated with Lyso Tracker Deep Red (100 nM, 10 min incubation) at 37 °C, followed by three washes with PBS. Where indicated, the cells were then treated with 50 µM FCCP for 30 min at 37 °C. After FCCP treatment, the cells were washed once with PBS and incubated with compounds **4b** or **4c** at a concentration of 100 nM for 10 min at 37 °C. Following the incubation period, the cells were washed with PBS three times. All acquisitions were performed in live-cell imaging solution containing 140 mM NaCl, 2.5 mM KCl, 1.8 mM $CaCl_2$, 1 mM $MgCl_2$, 20 mM HEPES, and 20 mM glucose (pH 7.4). Live-cell imaging was carried out on a Nikon Ti2 AX confocal microscope equipped with a 60×/1.42 NA Plan Apochromat oil-immersion objective. Compounds **4b** and **4c** were excited using a 405 nm laser, and the emitted fluorescence was collected between 550 and 600 nm. Lyso Tracker Deep red was excited using a 640 nm laser, and the corresponding emission was collected between 649 nm and 747 nm. At least 10 fields of view were acquired per chamber. Identical microscope acquisition settings were maintained across all experimental conditions.

Images were processed with NIS-elements software and were further analysed with FIJI (Fiji is just ImageJ) software.[2] First, the background subtraction was applied uniformly to all images. Colocalization analysis was performed in the BIOP JACoP plugin using fast sequential mode, with Channel A assigned to LysoTracker Deep Red and Channel B assigned to the compound signal (**4b** or **4c**).[5] Otsu threshold[6] was applied to all images prior to colocalization analysis. For each separate condition 10 images were analysed using identical image-processing parametrs.

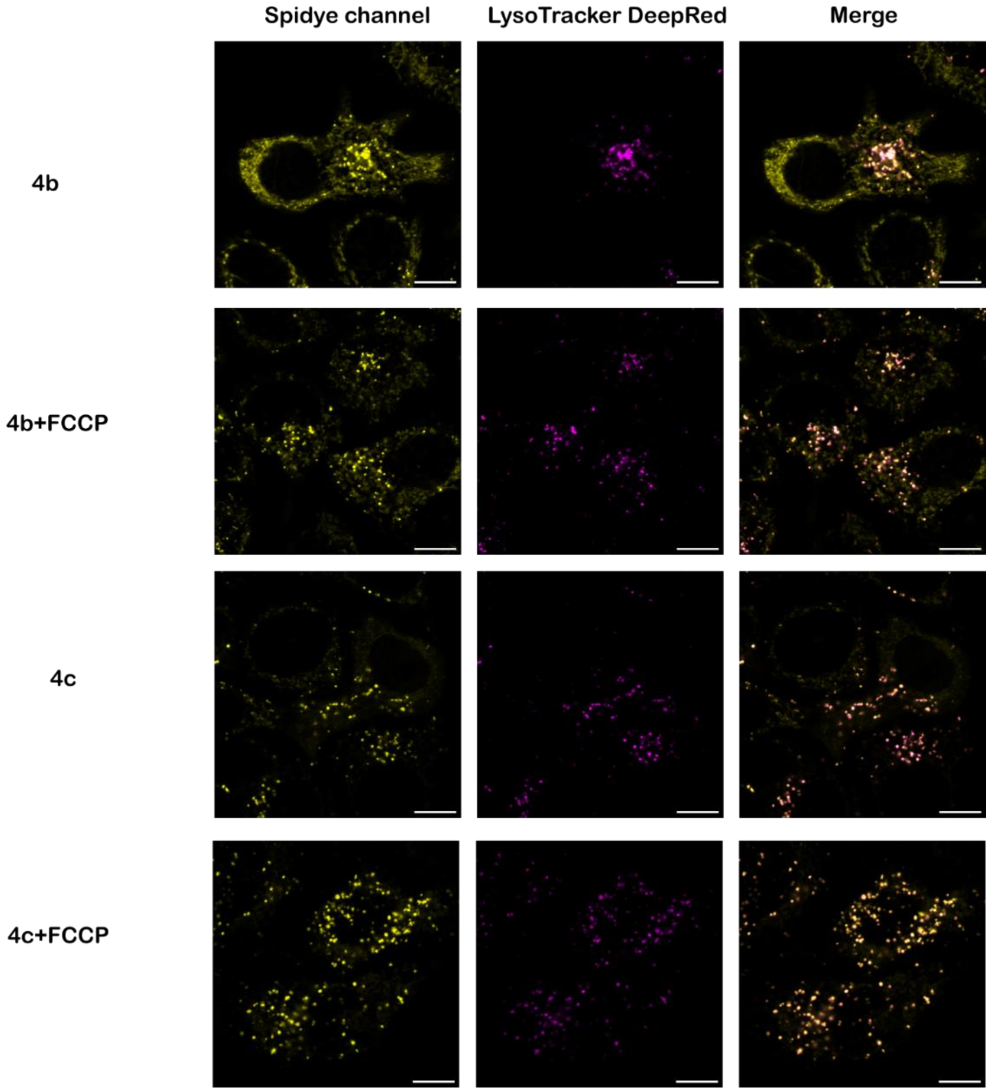

Figure S 13. Representative confocal fluorescence microscopy images of COLO-357 co-stained with 4b or 4c and LysoTracker Deep Red. Where indicated, cells were pretreated with FCCP (50 µM, 30 min) prior to staining with **4b** or **4c** (100 nM, 10 min). Spidye channel: λ_{ex} = 405 nm, λ_{em} = 550 – 600 nm and LysoTracker Deep Red: λ_{ex} = 640 nm, λ_{em} = 649 – 747 nm. Images were acquired using NikonTi2 AX confocal microscope. Scale bars, 10 µm. Images are representative of two independent biological replicates.

Table S 4. Colocalization analysis of **4b** and **4c** with LysoTracker Deep Red in untreated and FCCP-treated COLO-357 cells.

Cpd	Pearson[a] (LysoTracker)	M1[b] (LysoTracker)	M2[c] (LysoTracker)
**4b**	0.56 ± 0.11	0.96 ± 0.03	0.20 ± 0.23
**4b + FCCP**	0.78 ± 0.09	0.89 ± 0.08	0.57 ± 0.26
**4c**	0.88 ± 0.03	0.85 ± 0.03	0.80 ± 0.05
**4c + FCCP**	0.93 ± 0.01	0.88 ± 0.04	0.85 ± 0.05

[a] linear correlation of fluorescence intensities in A and B across pixels. [b] fraction of pixels in channel A overlapping with positive pixels in channel B. [c] fraction of positive pixels in channel B overlapping with positive pixels in channel A. Data in [a–c] represent mean ± SEM of at least 10 images from two independent biological replicates.

Resazurin assay (Figure S14)

Cell viability was assessed using several cell lines cultured under specific conditions. COLO-357 cells were maintained in Advanced RPMI-1640 medium (Gibco, Thermo Fisher Scientific, Waltham, MA, USA) supplemented with 5% heat-inactivated fetal bovine serum (FBS; Gibco), 100 U/mL penicillin, 100 µg/mL streptomycin (Sigma-Aldrich, St. Louis, MO, USA), and 1% GlutaMAX (Gibco). PANC-1 cells were cultured in DMEM with GlutaMAX (Gibco), supplemented with 10% heat-inactivated FBS, 100 U/mL penicillin, and 100 µg/mL streptomycin. NHLF cells were maintained in FGM™-2 Fibroblast Growth Medium-2 BulletKit™ (Lonza, Basel, Switzerland). B16F10, C2C12, and L929 cells were cultured in Advanced DMEM supplemented with 5% heat-inactivated FBS, 100 U/mL penicillin, 100 µg/mL streptomycin, and 1% GlutaMAX.

Cells were seeded in 96-well plates at appropriate densities based on cell type and allowed to adhere for 24 hours in their respective culture media. Following this, compounds were added at varying concentrations and incubated for 72 hours at 37 °C in a humidified atmosphere containing 5% CO_2. After incubation, 10 µL of PrestoBlue™ reagent (Thermo Fisher Scientific) was added to each well, and plates were incubated for an additional hour under the same conditions. Emitted fluorescence intensity was measured with Cytation 1 Cell Imaging Multimode Reader (BioTek, V, US). IC_{50} values were determined by fitting a four-parameter variable slope model using GraphPad Prism version 10.3.1 (Dotmatics, CA, US). All treatments were performed in at least three technical and three biological replications to ensure reliable and reproducible results.

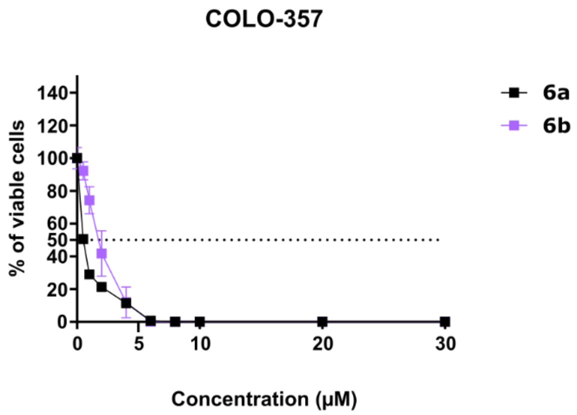

	6a	6b
IC_{50}	0,530	1,838
SD	0,111	0,590

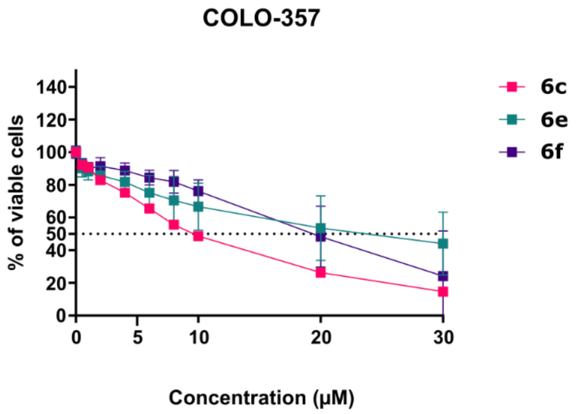

	6c	6e	6f
IC_{50}	13,377	13,814	24,323
SD	1,138	6,415	6,247

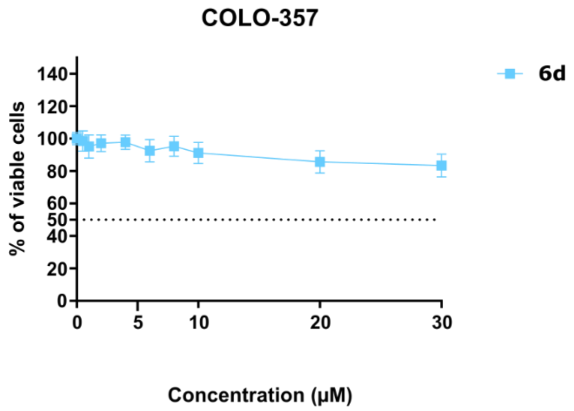

	6d
IC_{50}	/
SD	/

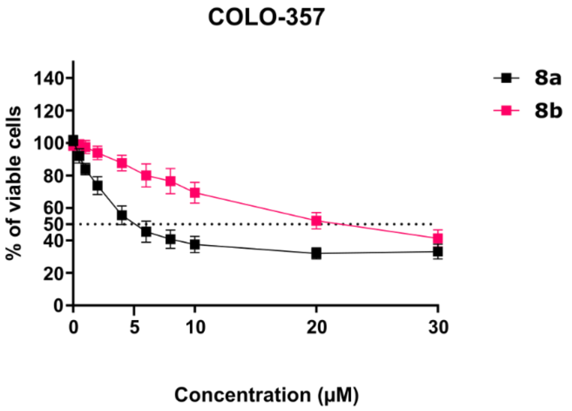

	8a	8b
IC_{50}	2,881	16,177
SD	0,500	4,597

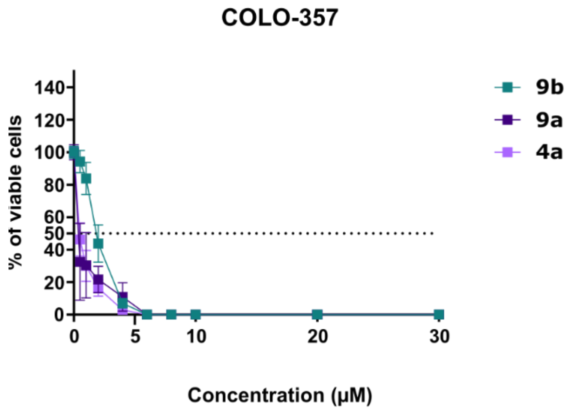

	9b	9a	4a
IC_{50}	1,892	0,546	0,528
SD	0,219	0,425	0,211

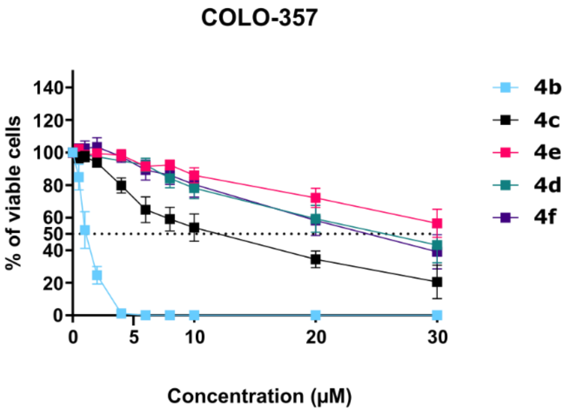

	4b	4c	4e	4d	4f
IC_{50}	0,935	11,020	>30	18,227	29,103
SD	0,143	5,470		4,800	7,922

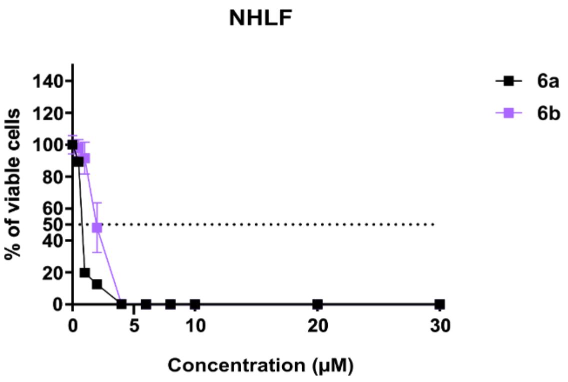

	6a	6b
IC_{50}	0,715	1,964
SD	0,037	0,153

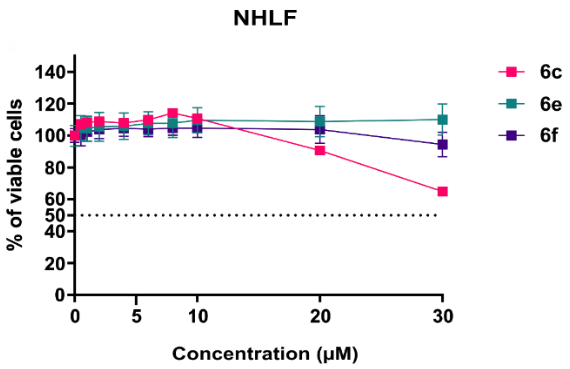

	6c	6e	6f
IC_{50}	/	/	/
SD	/	/	/

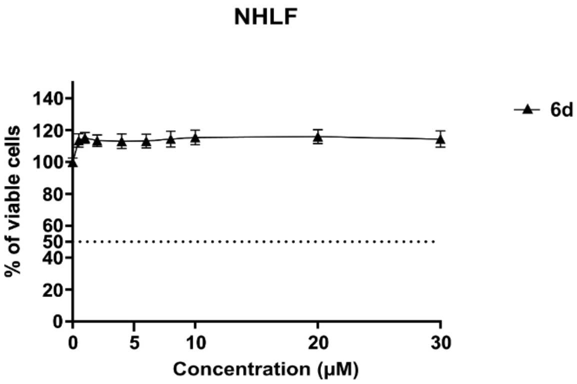

	6d
IC_{50}	/
SD	/

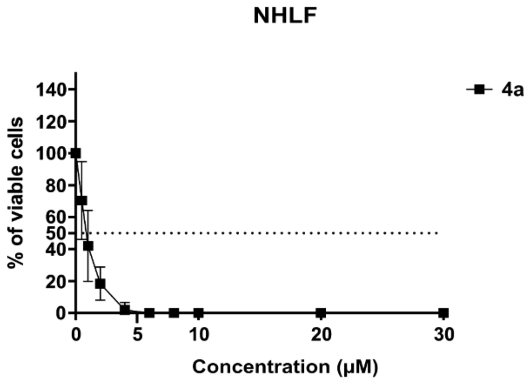

	4a
IC_{50}	0,957
SD	0,376

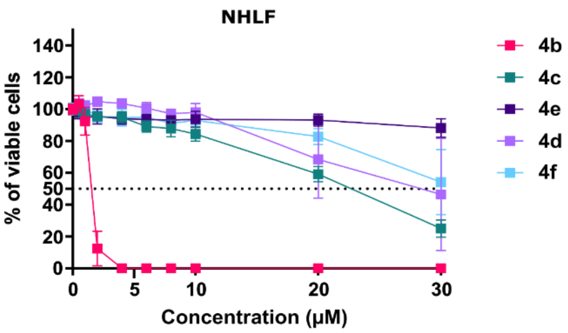

	4b	4c	4e	4d	4f
IC_{50}	1,431	>30	8,000	22,833	>30
SD	0,098		2,842	4,748	

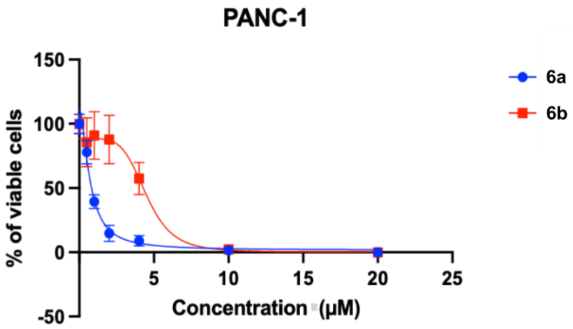

	6a	6b
IC50	0.7148	4.505

L929

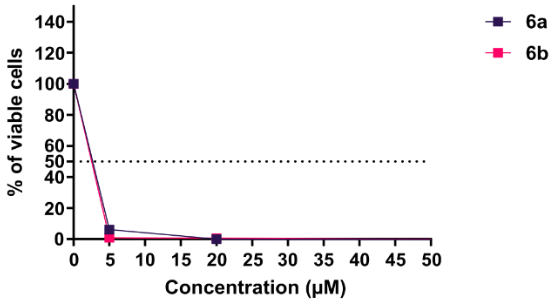

	6a	6b
IC50	1,816	1,583
SD	0,018	0,023

C2C12

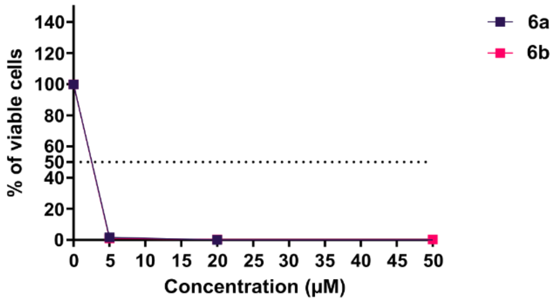

	6a	6b
IC_{50}	1,608	1,566
SD	0,017	0,017

B16F10

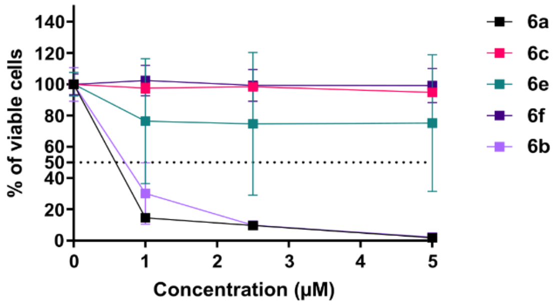

	6a	6c	6e	6f	6b
IC_{50}	0,443	/	/	/	0,454
SD	0,01	/	/	/	0,045

B16F10

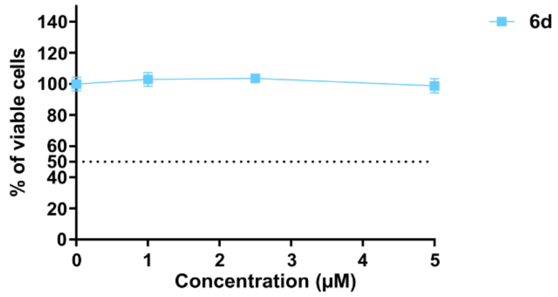

	6d
IC_{50}	/
SD	/

Figure S 14. Representative dose–response curves for Resazurin assay after 72 h exposure, and IC_{50} values corresponding to half maximal inhibitory concentrations given as mean and SD (n ≥3 biological replicates, each with ≥3 technical replicates).

Electrophysiology (Figure S15, Table S5)

Patch-clamp experiments.

Mouse L929 fibroblasts stably expression mKv1.3 were cultured as previously described.[9] All experiments were conducted with an EPC-10 amplifier (HEKA, Lambrecht/Pfalz, Germany) in the whole-cell configuration with a holding potential of −80 mV. Pipette resistances averaged around 2.5 MΩ. Compound solutions were prepared fresh immediately before the experiment in Na^+ Ringer from 10 mM stock solutions in DMSO. Current measurements were measured with an internal pipette solution containing 160 mM KF, 2 mM $MgCl_2$, 10 mM HEPES, and 10 mM EGTA, with a pH of 7.2 and an osmolarity of ~ 300 mOsm. Sodium Ringer was used as an external solution containing the following: 160 mM NaCl, 4.5 mM KCl, 2 mM $CaCl_2$, 1 mM $MgCl_2$, and 10 mM HEPES, with a pH of 7.4 and an osmolarity of ~ 300 mOsm. Currents were elicited with a 200-ms voltage step to +40 mV, followed by 30 seconds of holding at a resting membrane potential of −80 mV. If currents exceeded 2 nA 60–80% series resistance compensation was used. Concentration-dependent current inhibition as measured as reduction of area under the current curve was fitted with the Hill equation using GraphPad Prism8 (GraphPad Software, La Jolla, CA). All data points are presented as mean ± standard deviation (SD). IC_{50}s are reported with 95% confidence intervals (CI).

Table S 5. Percentage of Kv1.3 current inhibition at 10 µM

	**% inhibition**	**SD**	**cells (n)**
**6a (PAPTP)**	93.9	7.5	4
**6b**	87.1	3.6	3
**6c**	90.7	11.7	3
**6d**	60.7	12.5	3
**6e**	11.4	6.3	3
**6f**	17.4		1

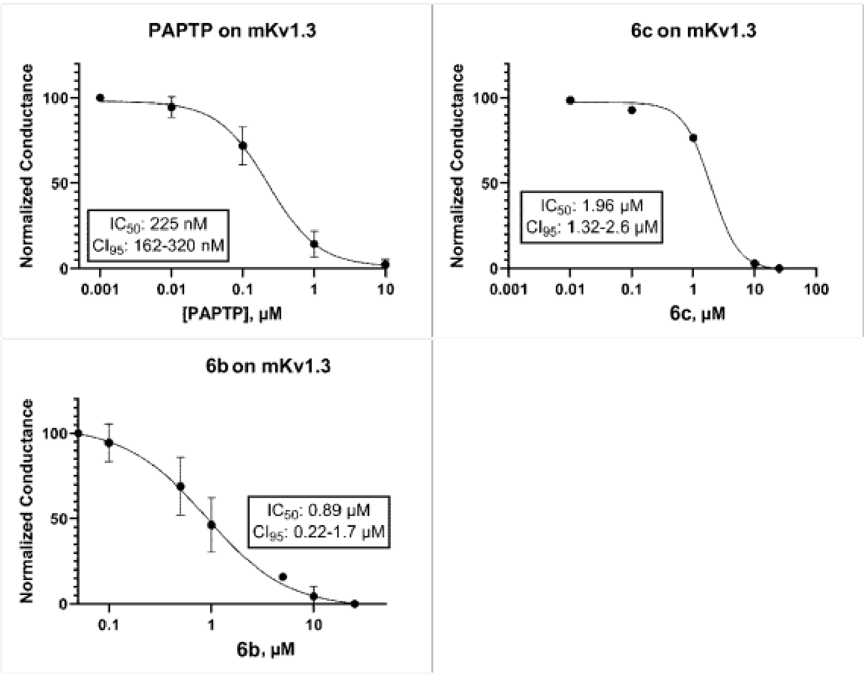

Figure S 15. Dose-response curves (patch-clamp experiments) for mKv1.3 inhibition of compounds **6a**, **6b** and **6c**.

Cellular uptake of the Kv1.3 inhibitor conjugates by flow cytometry (Figure S16)

In order to estimate the cellular uptake of the Kv1.3 inhibitor conjugates in coupled versus uncoupled conditions, the total cell fluorescence of the weakly fluorescent psoralene core was measured using flow cytometry. COLO-357 cells were detached using trypsin and 100,000 cells were pre-treated with 50 µM carbonyl cyanide-p-trifluoromethoxyphenylhydrazone (FCCP) in 0.5 mL of complete medium for 10 min at 37 °C. In parallel, a sample without FCCP pretreatment was prepared for every investigated compound. Following this, 10 µM of the investigated Kv1.3 inhibitors was added for a further 60 min incubation at 37 °C. The samples were then transferred to ice, spun down at 230 g for 5 min at 4 °C. The supernatant was removed, the cells washed once with cold 1× PBS, spun down again and resuspended in 400 µL of fresh cold 1× PBS. The cells were then kept on ice and the mean total cell fluorescence signal area immediately measured using the Attune™ NxT flow cytometer (channel VL-3, excitation 405 nm, emission filter 710 nm/50 nm). At least 10,000 events were obtained for each sample. The fluorescence of FCCP pre-treated or untreated cells was subtracted from the fluorescence signals obtained for Kv1.3 inhibitor treated cells with and without FCCP-pre-treatment, respectively, to correct for background (auto)fluorescence. Since the targeting of mitochondria with permanently charged lipophilic cations depends on the negative plasma-, and mitochondrial membrane potential, the signals obtained in the presence of FCCP, a protonophore and uncoupler of oxidative phosphorylation, were used to estimate the accumulation of Kv1.3 inhibitor that is membrane potential-dependent. Conversely, the difference between the total cellular uptake of Kv1.3 inhibitors in the absence and presence of FCCP was used to estimate the membrane potential-independent fraction of the compounds, if any.

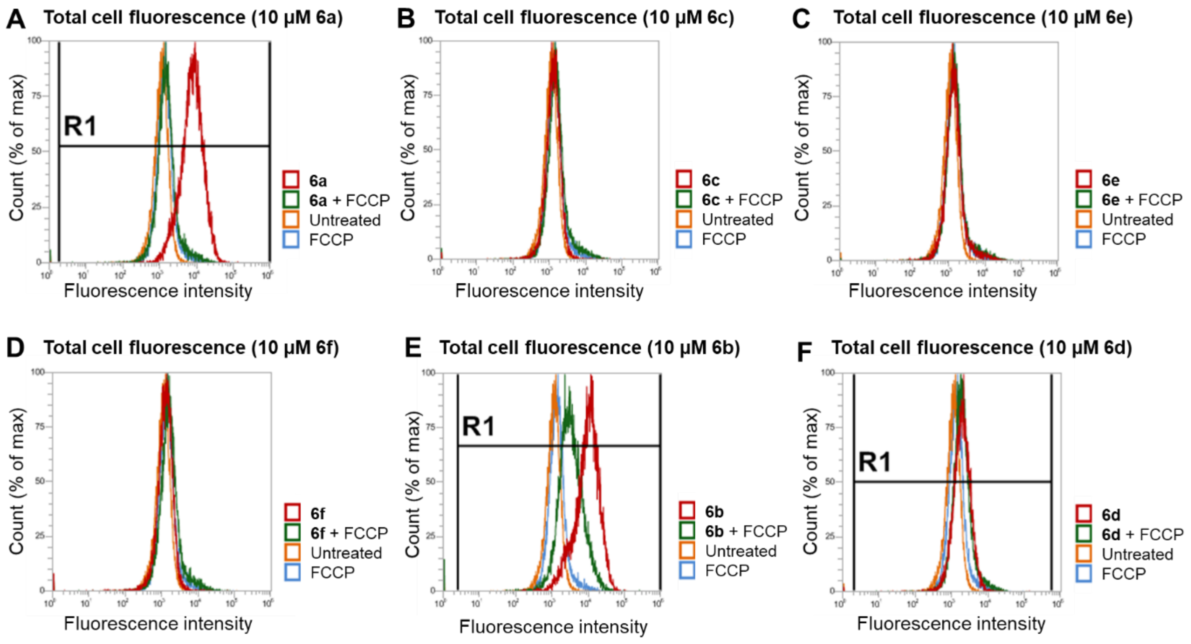

Figure S 16. Cellular uptake of cationic conjugates with psoralen-cored Kv1.3 inhibitor. COLO-357 cells were treated with 10 µM Kv1.3 inhibitors for 1 h at 37 °C with or without 50 µM FCCP pretreatment for 10 min prior as described above. Representative histograms of fluorescence intensity (log scale) are shown for one of three independent experiments. Untreated cells and cells treated only with 50 µM FCCP were used as additional controls. Substantial cellular uptake was only detected for **6a**, **6b** and **6d**. Both **6a** and **6b** exhibited a major drop in fluorescence in the presence of FCCP pretreatment,

but the residual, membrane potential-independent accumulation was somewhat larger for **6b**. On the contrary, the uptake of **6c**, **6e** and **6d** did not notably decrease in the presence of FCCP pretreatment.

Determination of mitochondrial localization of 6b by LC-MS (Figures S17–S21, Table S6)

In order to confirm the effective mitochondrial accumulation of the Kv1.3 inhibitor **6b**, its concentration in cytoplasmic and mitochondrial subcellular fractions was measured by LC-MS. COLO-357 cells were treated with 1 µM **6b** for 1 h at 37 °C. Three T150 cell culture flasks (yielding approximately 25×10^6 cells) were used for each independent experiment. After treatment, the cells were detached, washed with PBS and resuspended in 2 mL STM buffer (250 mM sucrose, 50 mM Tris–HCl pH 7.4, 5 mM $MgCl_2$, protease and phosphatase inhibitor cocktail). A 100 µL aliquot was removed, washed with PBS, lysed in 100 µL RIPA buffer, sonicated, shaken for 30 min, spun down at 15,000 g to extract the protein into the supernatant and labeled as the whole cell lysate (WCL).

The rest of the sample was used to perform subcellular fractionation according to a protocol adapted from Dimauro et al.[10] with the modifications described below. The cells were homogenized using a tight-fitting rotating Teflon pestle homogenizer. 4 cycles of 1 min homogenization at 1000 rpm with the movement of the pestle up and down the sample containing tube. These cycles were interspersed with 1 min breaks, with the sample kept on ice throughout the procedure. The homogenate was then left on ice for 30 min, transferred to a microcentrifuge tube and spun down for 15 min at 800 g. The supernatant was transferred to a new tube and three more centrifugations at 800 g for 10 min were performed to remove residual nuclei. After this, the final supernatant was spun down at 11,000 g for 10 min to separate the cytoplasmic and mitochondrial fractions. The supernatant was then spun down again at 11,000 g for 10 min and the new supernatant was labeled as the cytoplasmic fraction used to prepare the LC-MS sample. For western blot analysis, the proteins from this fraction were isolated by the addition of equal volume of cold acetone and overnight precipitation at −20 °C, followed by centrifugation at 12,000 g and resuspending of the resulting pellet in STM buffer.

The mitochondrial fraction (the pellet from the first 11,000 g centrifugation) was then further purified by resuspending it in 500 µL of STM buffer and additional centrifugation at 11,000 g for 10 min. The pellet was again resuspended in STM buffer and spun down twice at 3,500 g for 10 min to reduce the lysosome content in the mitochondrial fraction. The final pellet was resuspended in 75 µL of SOL buffer (50 mM Tris HCl pH 6.8, 1 mM EDTA, 0.5% Triton-X-100, protease and phosphatase inhibitors) and homogenized by sonification on ice; this was labeled as the final mitochondrial fraction. All the centrifugation steps for both fractions were performed at 4 °C and the samples kept on ice between these steps. Three independent experiments were performed on separate days. Additionally, a sample of untreated cells was prepared to exclude any matrix interference.

The samples were stored at −80 °C until further use. The protein content of all the fractions was determined using the DC protein method and diluted with their corresponding buffer to the total protein concentration of 20 µg/mL (mitochondrial fraction) or 200 µg/mL (whole cell lysate and cytoplasmic fraction) for further LC-MS sample processing.

SDS-PAGE and Western blot

To confirm the purity of the subcellular fractions, their content of protein markers characteristic for cytoplasm (GAPDH), mitochondria (TOM20) and lysosomes (LAMP1) were investigated by SDS-PAGE and Western blotting as described previously.[11] A total of 8.75 µg of protein of each fraction (whole cell lysate, mitochondrial and cytosolic fraction) were loaded onto 12.5 % (TOM20) and 10 % (GAPDH and LAMP1) polyacrylamide gells. The GAPDH (D4C6R, #97166), TOM20 (D8T4N, #42406) and LAMP1 (D2D11, #9091) antibodies were from Cell Signaling Technology (Danvers, MA, USA). The membranes were incubated with the primary antibody (1:1000 dilution; 5% BSA/TTBS) at 4 °C overnight. After washing, membranes were incubated with the appropriate secondary antibodies conjugated with horseradish peroxidase, for 1 h at room temperature. Secondary antibodies Horse Anti-mouse IgG, HRP-linked Antibody (#7076; Cell Signaling Technology, Danvers, MA, USA) or Goat Anti-Rabbit IgG Antibody, HRP-conjugate (12-348; EMD Millipore, Burlington, MA, USA) were prepared as 1:10,000 dilution in 5% BSA/TTBS. Then, the SuperSignal West Femto substrate (Thermo Fisher Scientific, Pierce, IL, USA) was added, following the manufacturer instructions, and the chemiluminescence was measured.

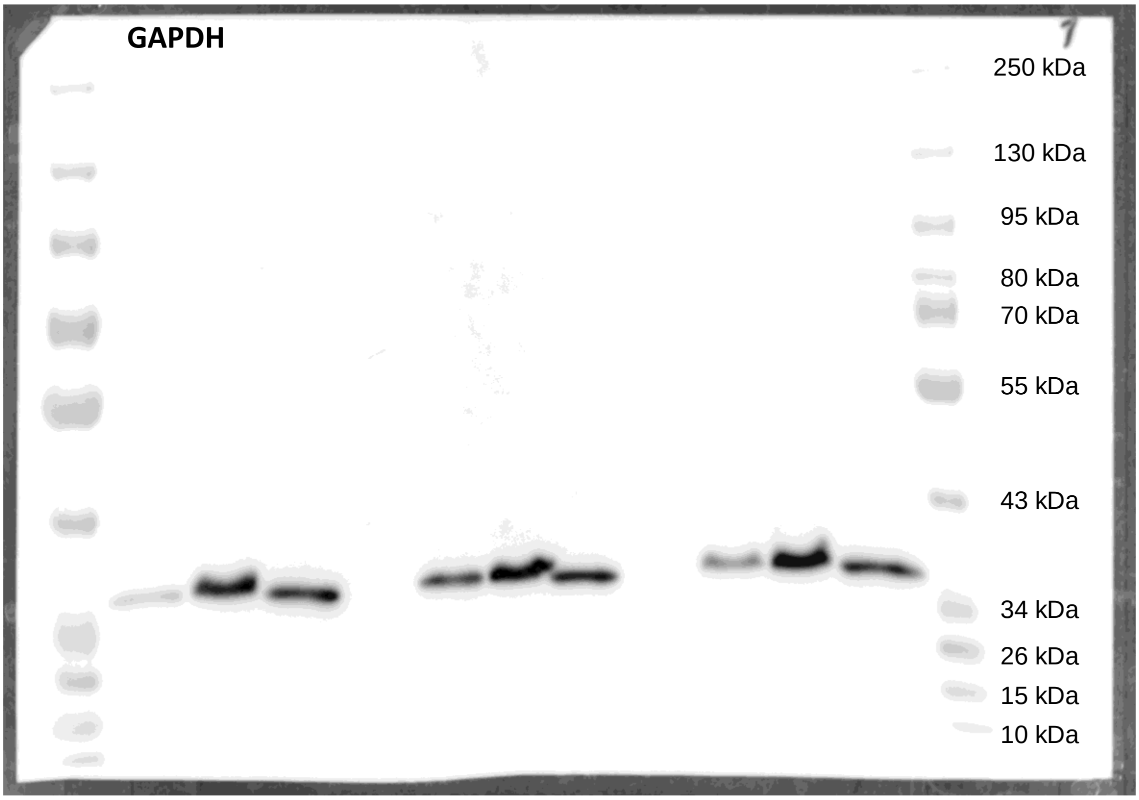

**Mito**	**Cyto**	**WCL**		**Mito**	**Cyto**	**WCL**		**Mito**	**Cyto**	**WCL**
Experiment 1				Experiment 2				Experiment 3		

Figure S 17. Western blot analysis of GAPDH protein content in subcellular fractions. COLO-357 cells were treated with 1 µM Kv1.3 inhibitor **6b** for 1 h at 37 °C, after which the subcellular fractionation procedure was performed as described above. The GAPDH content was determined by western blotting for three independent experiments. The cytoplasmic fraction (Cyto) showed a notable increase in GAPDH and intensity compared to whole cell lysate (WCL). Some trace GAPDH was still present in the mitochondrial fraction (Mito), particularly in experiment 2, but its content was considerably lower than in whole cell lysate. This is full-size blot corresponding to the cropped image shown in Figure 5 of the manuscript.

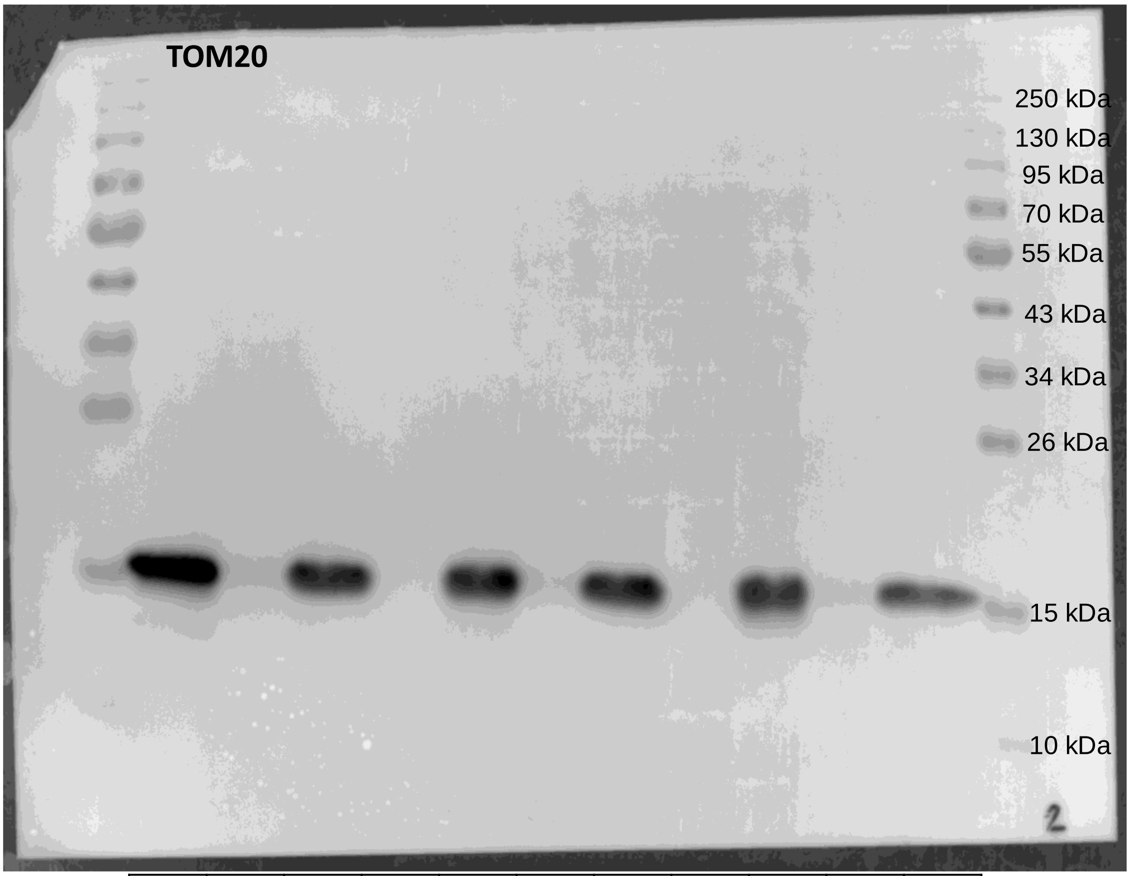

Mito	Cyto	WCL		Mito	Cyto	WCL		Mito	Cyto	WCL
Experiment 1				Experiment 2				Experiment 3		

Figure S 18. Western blot analysis of TOM20 protein content in subcellular fractions. COLO-357 cells were treated with 1 µM Kv1.3 inhibitor **6b** for 1 h at 37 °C, after which the subcellular fractionation procedure was performed as described above. The TOM20 content was determined by western blotting for three independent experiments. The mitochondrial fraction (Mito) showed a modest increase in TOM20 content compared to the whole cell lysate (WCL). Importantly, no TOM20 protein was detected in the cytoplasmic fraction (Cyto), indicating no mitochondria were present in the cytoplasmic fraction. This is full-size blot corresponding to the cropped image shown in Figure 5 of the manuscript.

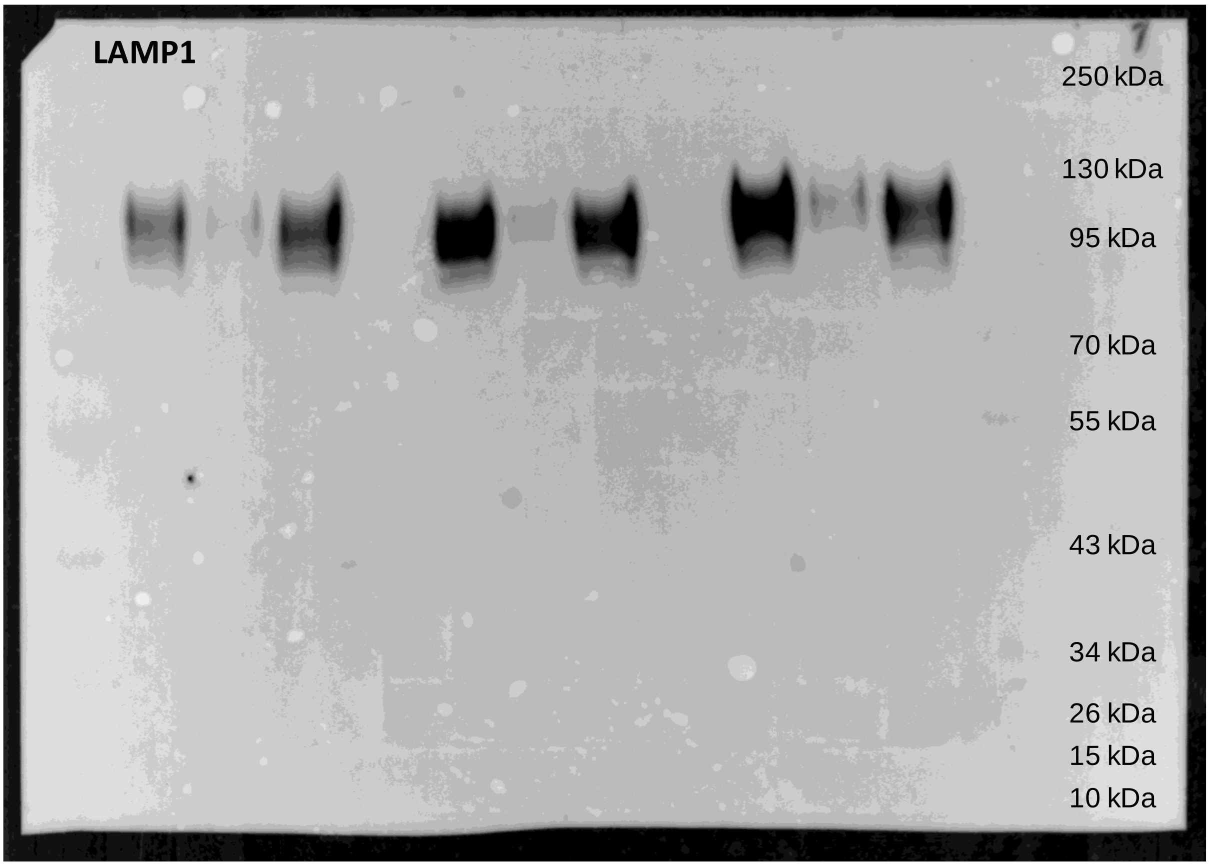

Mito	Cyto	WCL		Mito	Cyto	WCL		Mito	Cyto	WCL
Experiment 1				Experiment 2				Experiment 3		

Figure S 19. Western blot analysis of LAMP-1 protein content in subcellular fractions. COLO-357 cells were treated with 1 µM Kv1.3 inhibitor **6bS** for 1 h at 37 °C, after which the subcellular fractionation procedure was performed as described above. The LAMP1 content was determined by western blotting for three independent experiments. The mitochondrial fraction (Mito) showed lysosomal content aproximately comparable to the whole cell lysate (WCL). Only trace amounts of LAMP1 were detected in the cytoplasmic fraction (Cyto), indicating very few lysosomes were present in the cytoplasmic fraction.

LC-MS/MS analysis

To 50 µL of cell lysate, 200 µL of acetonitrile was added to precipitate proteins. The mixture was vortexed thoroughly and centrifuged at 18,000 × g. A 200 µL aliquot of the resulting supernatant was transferred into HPLC vials for LCMS analysis. When necessary, samples were further diluted with 20% MeCN to ensure that the analyte concentration fell within the calibration range.

Chromatographic separation was performed on a Vanquish Flex system (Thermo Fisher Scientific) equipped with an Acquity UPLC® BEH C8 column (50 mm × 2.1 mm, 1.7 µm; Waters). The column temperature was maintained at 40 °C, and the injection volume was 10 µL. The mobile phase consisted of water/MeCN/formic acid (950:5:1, v/v/v) as solvent A and water/MeCN/formic acid (50:950:1, v/v/v) as solvent B. The gradient program was as follows: 0–3.8 min, 20–80% B; 3.8–3.9 min, 80–100% B; 3.9–5.0 min, 100% B; 5.0–5.1 min, 100–20% B; 5.1–7.0 min, 20% B for re-equilibration. The flow rate was kept constant at 0.40 mL/min throughout the analysis, and from 3.0 to 4.0 min the eluate

was directed to the mass spectrometer (compound **6b** eluted at 3.48 min). The autosampler temperature was maintained at 5 °C.

MS/MS analysis was carried out using a TSQ Fortis triple quadrupole mass spectrometer (Thermo Fisher Scientific) equipped with a heated electrospray ionization (H-ESI) source operated in positive ionization mode and single-reaction monitoring (SRM). Optimized chromatographic and MS/MS parameters are summarized in Table S6. Instrument control, data acquisition, and quantification were performed using Xcalibur software (Thermo Fisher Scientific).

Table S 6. Optimized MS/MS parameters.

**Parameter**	**Value**
Precursor ion (m/z)	622.26
Product ion (m/z)	231.01
Ion source voltage (V)	4000
Vaporizer temperature (°C)	180
Sheath gas (AU)	40
Auxiliary gas (AU)	6
Sweep gas (AU)	1
Collision energy (V)	55
CID (argon gas) (mTorr)	2

The calibration curve was linear over the range of 0.01–1 ng/mL (see Figures S20 and S21). The LOQ was defined as the lowest calibration point. Accuracy and precision were determined to be 90.1% and 5.8% at 0.05 ng/mL, and 90.6% and 3.0% at 0.5 ng/mL, respectively.

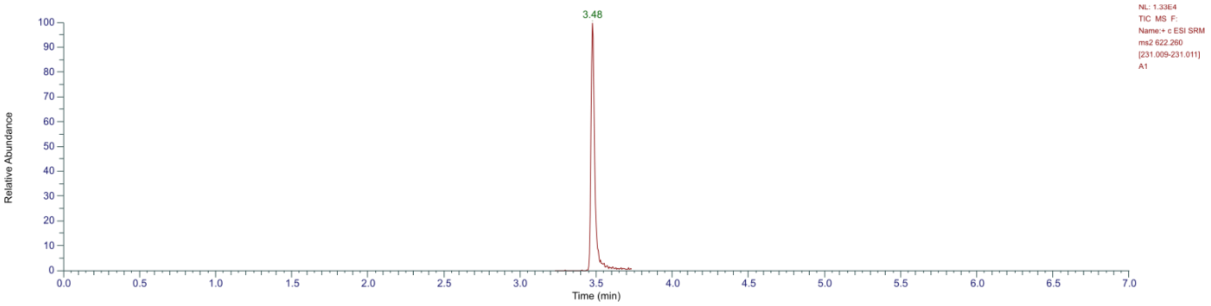

Figure S 20. Representative chromatogram of compound **6b**.

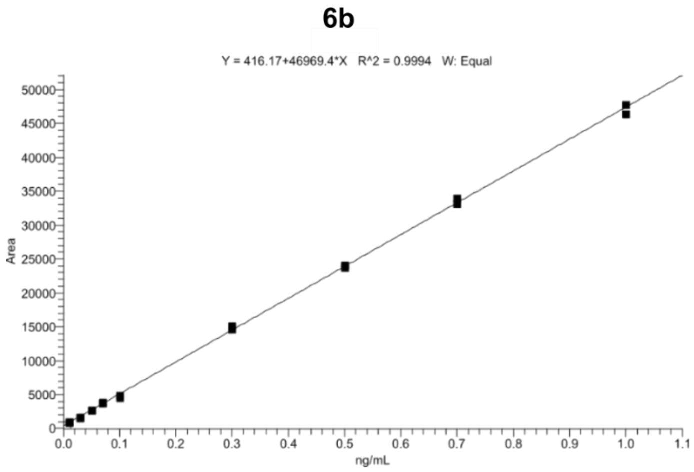

Figure S 21. Calibration curve for **6b**.

Partition coefficient, logD

Determinations of octanol/buffer logD were performed by a contract research organization WuXi AppTec, China.

1-Octanol-saturated buffer was prepared by vigorously shaking a mixture of 1-octanol (10 mL) and 100 mM phosphate buffer, pH 7.4 (100 mL) and letting it stand overnight at room temperature. The buffer layer was separated and stored at room temperature for a maximum of 1 week. Buffer-saturated 1-octanol was prepared by vigorously shaking a mixture of 100 mM phosphate buffer, pH 7.4 (10 mL) and 1-octanol (100 mL) and letting it stand overnight at room temperature. The octanol layer was separated and stored at room temperature for a maximum of 1 week. Internal standard (IS) was prepared as a 1.0 mg/mL solution in acetonitrile. 402 µL of IS solution and 50 mL of 1-octanol-saturated buffer was dissolved in 949 mL of methanol/water 1:1 to get 1-octanol IS solution. 421 µL of IS solution and 5.263 mL of buffer-saturated 1-octanol was dissolved in 994 mL of methanol/water 1:1 to get Buffer (pH 7.4) solution. Test compounds (10 mM in DMSO; 2 µL/well) were transferred in duplicate from storage tubes to the 96-well polypropylene cluster tubes. Buffer-saturated 1-octanol (150 µL/well) and 1-octanol saturated buffer (150 µL/well) were added to each well. Each of the tubes was vigorously mixed on their sides for 1 minute, shaken 1 hour at a speed of 600 rpm at 28 °C, then centrifuged at 4000 rpm for 10 minutes. The buffer layer sample was diluted by a factor of X-fold and 1-octanol layer sample by a factor of Y-fold with IS solution. Usually X and Y correspond to 20 and 200, respectively. The concentration of the test compounds was determined using LCMSMS. The LC/MS/MS parameters of the IS were as follows: precursor m/z 329.0; product m/z 311.2; Q1 Pre Bias (v) -20.0; CE -15; Q3 Pre Bias (v) -20.0. Sample analysis was performed using a triple quadrupole mass spectrometer. Diluted samples were injected onto a Xbridge RP C18 (2.1×50 mm, 5 µm) HPLC column using an aqueous isocratic loading solvent (water/formic acid 1000:1), then flushed into the mass spectrometer with a fast gradient to organic elution solvent (acetonitrile/formic acid 1000:1). Peak areas were corrected by dilution factors and incorporating internal standard, and the ratio of the corrected peak areas were used to calculate the results (Log D value). The LogD value for each compound was calculated using the following equation:

$$\text{Log D}_{\text{1-Octanol / buffer}} = \log_{10}\left(\frac{[\text{Y-fold dilution of compound}]_{\text{1-octanol}} \times Y}{[\text{X-fold compound}]_{\text{buffer}} \times X}\right)$$

Thermodynamic solubility.

Determinations of solubility were performed by a contract research organization WuXi AppTec, China.

About 2.0 mg samples were weighed into lower chamber of Whatman miniuniprep vials (Cytiva) and 450 µL of buffer (10 mM phosphate-buffered saline, pH 7.4) was added into each chamber. After buffer addition, filter pistons of miniuniprep vials were placed and compressed to the position of the liquid level to allow for contact of buffer and compound with the filter during incubation. The thermodynamic solubility samples were vortexed for 2 minutes and incubated at room temperature (25±2 °C) for 24 hours with shaking at 600 rpm. Miniunipreps were compressed to prepare the filtrates for injection into HPLC system. All vials were inspected for visible undissolved material before filtering and for leakage after filtering. The supernatant was dilluted with buffer by a factor of 100 to make diluents. UV calibration standard solutions (1 µM, 20 µM, 200 µM) were injected from low to high

concentration, followed by the diluents and thermodynamic solubility supernatant. Testing samples were injected in duplicate. The UV chromatograms were integrated, the calibration equation was simulated and thermodynamic solubility was calculated. HPLC chromatograms were acquired on Agilent 1200 instrument, equiped with diode array detector, using Xbridge C18 column.

Single-crystal X-ray diffraction analysis of 7b (Figures S22–S26, Tables S7–S9)

Crystal structure was measured on the Rigaku OD XtaLAB Synergy-S diffractometer equipped with Cu and Ag PhotonJet micro-focus sealed X-ray tubes and an Eiger2 R CdTe 1M hybrid pixel detector. The dataset was collected with Cu *K*α radiation at 100 K.

CrysAlis^{Pro} software (Rigaku OD, *CrysAlis PRO*, **2025**) was used for data collection and reduction. Crystal structures were solved by *olex2.solve* and refined by *ShelXL* algorithm[12] within the *Olex2* (version 1.5) software.[13] Crystal structures were visualized using the *Diamond* program (K. Brandenburg, *Diamond – Crystal and Molecular Structure Visualization*, **2025**)

The compound **7** crystallizes in the non-centrosymmetric monoclinic space group *Pc* with Z = 4 and Z' = 2. The asymmetric unit is composed of 2 pyridinium cations and 2 chloride anions. The hydrogen atoms were placed in the calculated positions using the standard riding model (AFIX 43 for aromatic CH groups, AFIX 23 for CH_2 groups, and AFIX 137 for CH_3 groups). The *n*-propyl group in one of the pyridinium cations is disordered with the $\underline{CH_3}$ group rotated around the N−$\mathbf{CH_2}$−$\mathbf{CH_2}$−$\underline{CH_3}$ bond (123.2(4)°), with partial occupancies of 0.610(9) and 0.390(9). In both cases the C−CH_3 bond was restrained to the distance of the non-disordered one (DFIX 1.533 0.01). One of the chloride anions is severely disordered along the voids formed between cations along the *a*-crystallographic axis, with partial occupancies of 0.640(4), 0.213(4), 0.062(3), 0.057(4), and 0.028(4).

The phenyl substituents are not coplanar with the pyridinium ring but slightly twisted in both, non-disordered (22.34(7)°, 21.77(7)°) and disordered (28.39(8)°, 50.19(8)°) cations. The pyridinium ring and one of the phenyl substituents on each cation are involved in π-stacking between pyridinium ring and one of the phenyl groups. The other phenyl group is not involved in π-stacking, but rather only in weak interactions with nearby hydrogen atoms.

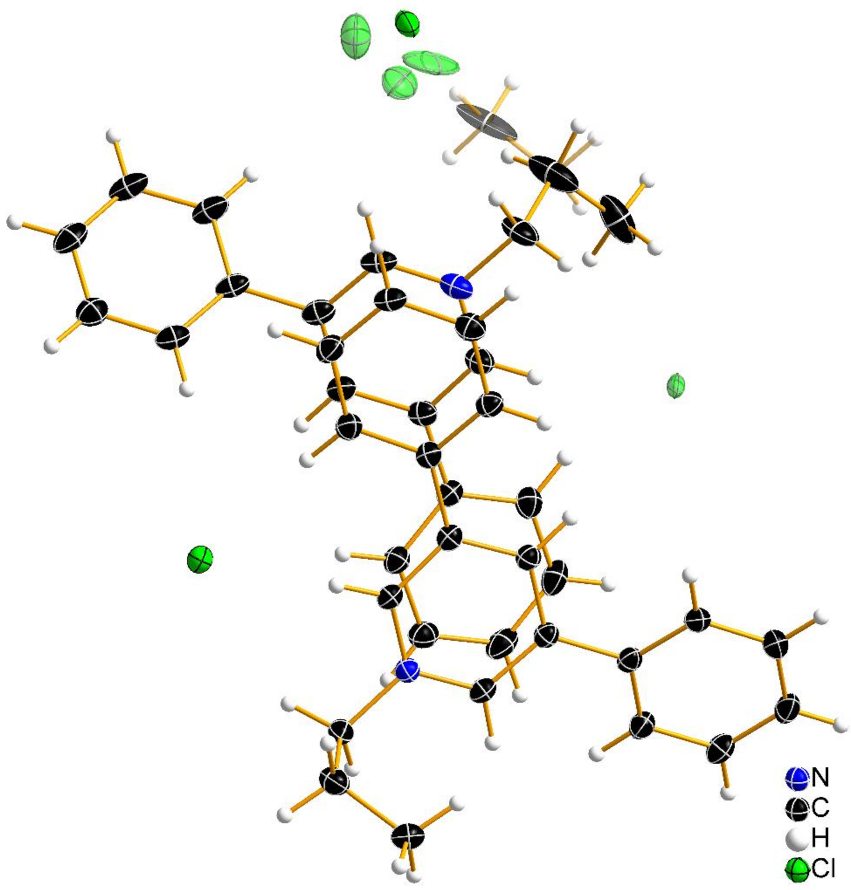

Figure S 22. The asymmetric unit of compound **7b** crystal structure. Displacement ellipsoids are depicted at the 50% probability level and hydrogen atoms are shown as small spheres of arbitrary radius. Minor disordered components are depicted as semitransparent.

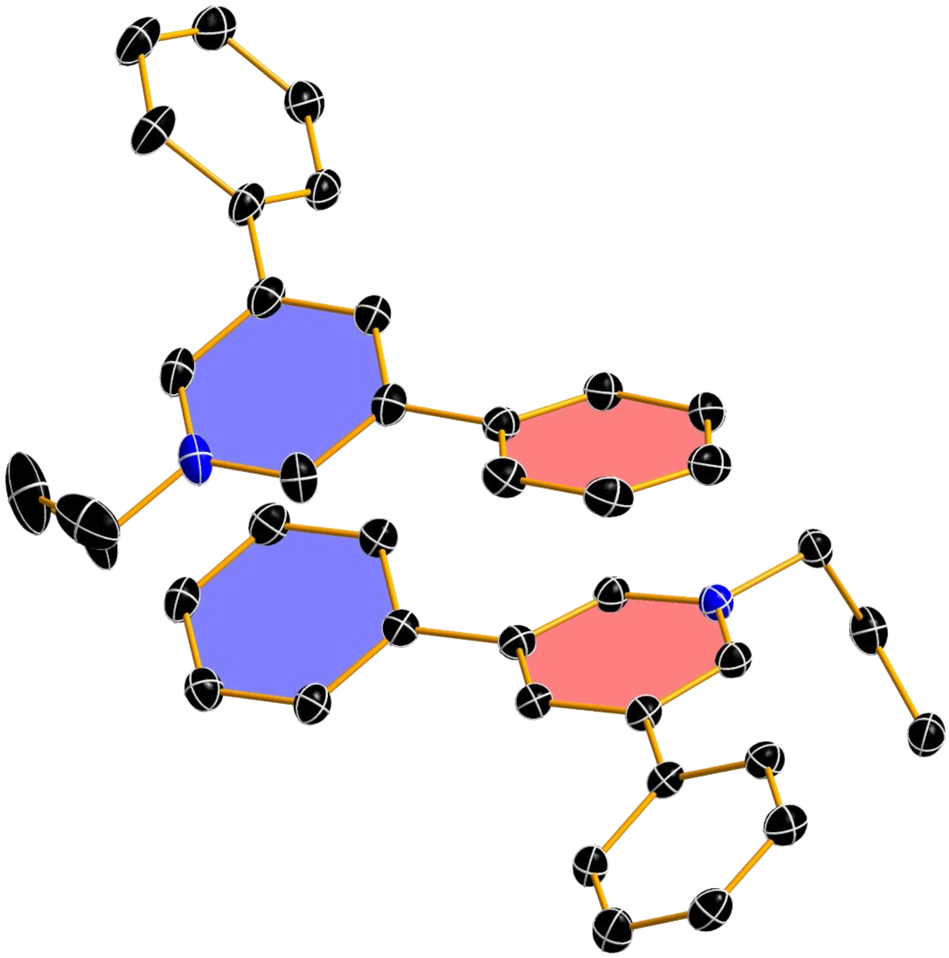

Parameter	π-π stacking 1	π-π stacking 2
Normal to normal angle [°]	0.72(8)	7.71(7)
Centroid to centroid distance [Å]	3.5914(13)	3.5807(13)
Plane to centroid distance [Å]	–3.4365(16)	3.4340(16)
Plane to plane shift [Å]	1.043(4)	1.014(3)
Plane to centroid distance [Å]	3.4356(16)	–3.3452(17)
Plane to plane shift [Å]	1.046(4)	1.277(4)
Plane twist angle [°]	2.5(3)	19.9(2)
Plane fold angle [°]	0.75(8)	7.99(8)

Figure S 23. The π-stacking motif in the cations of crystal structure of **7b**.

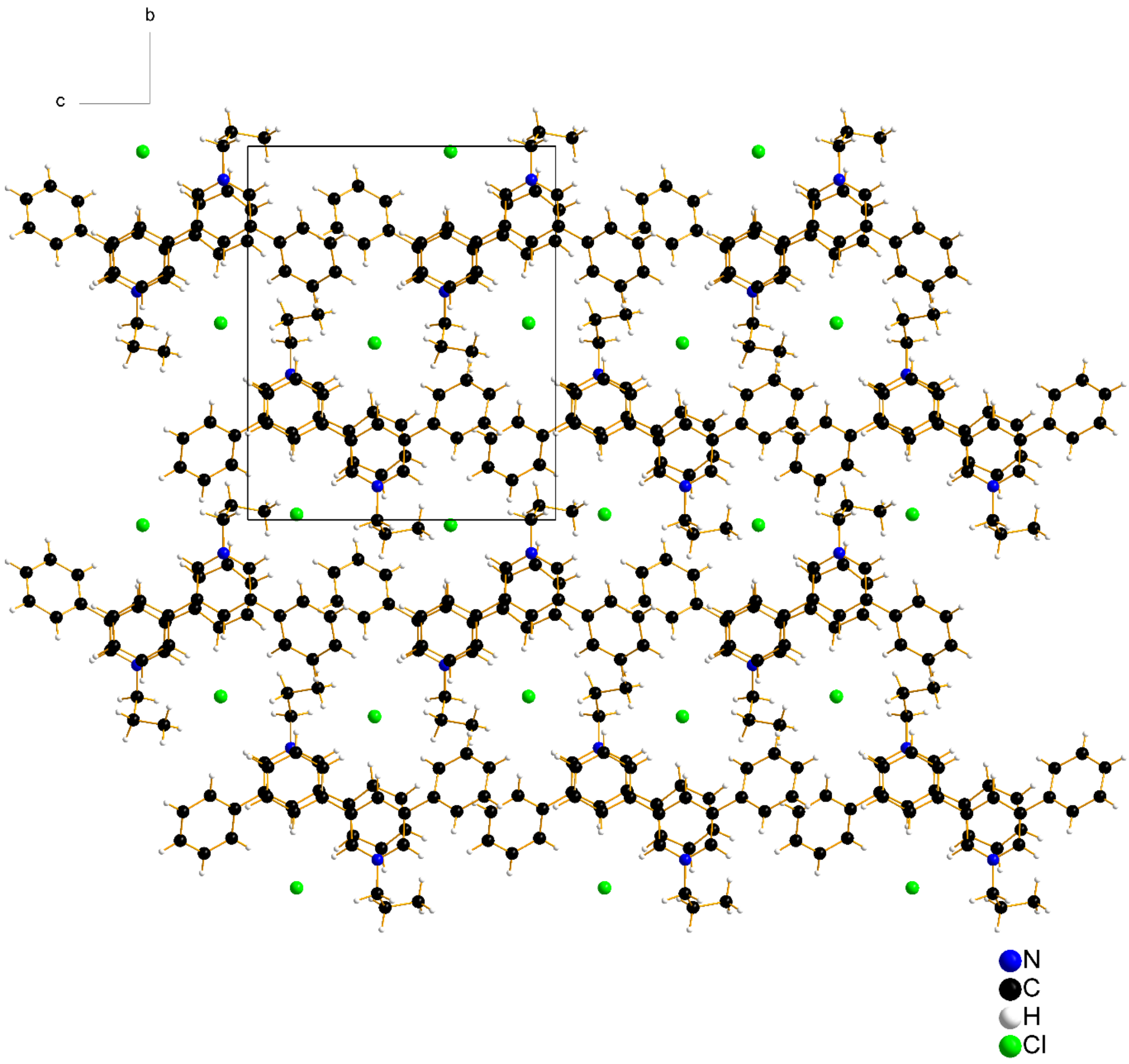

Figure S 24. The crystal packing and the unit cell of **7b** crystal structure viewed along the a-crystallographic axis. Only the major components of disordered atoms are shown for clarity.

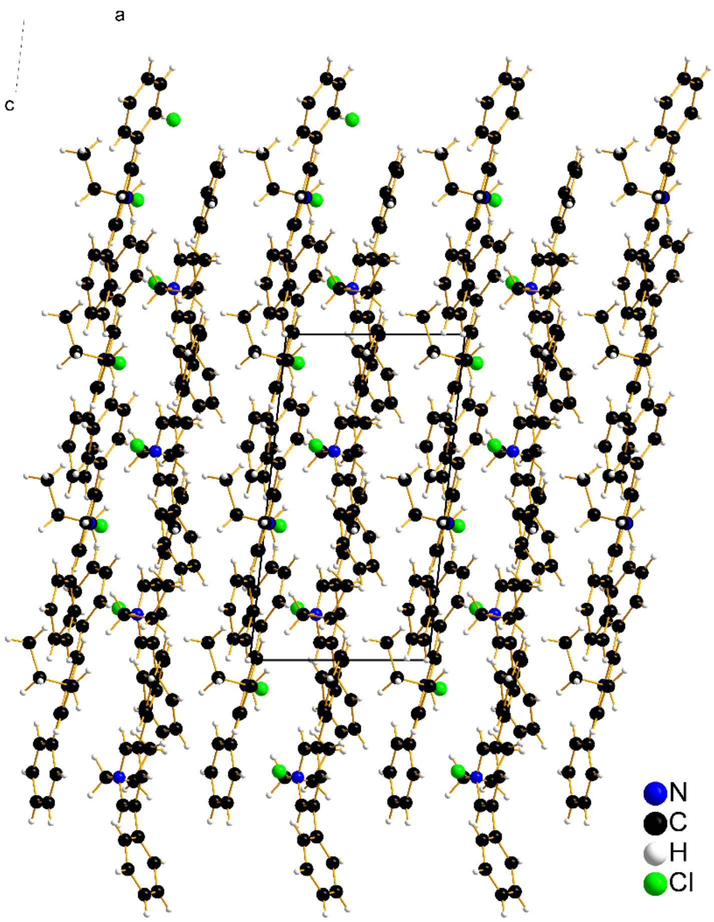

Figure S 25. The crystal packing and the unit cell of **7b** crystal structure viewed along the b-crystallographic axis. Only the major components of disordered atoms are shown for clarity.

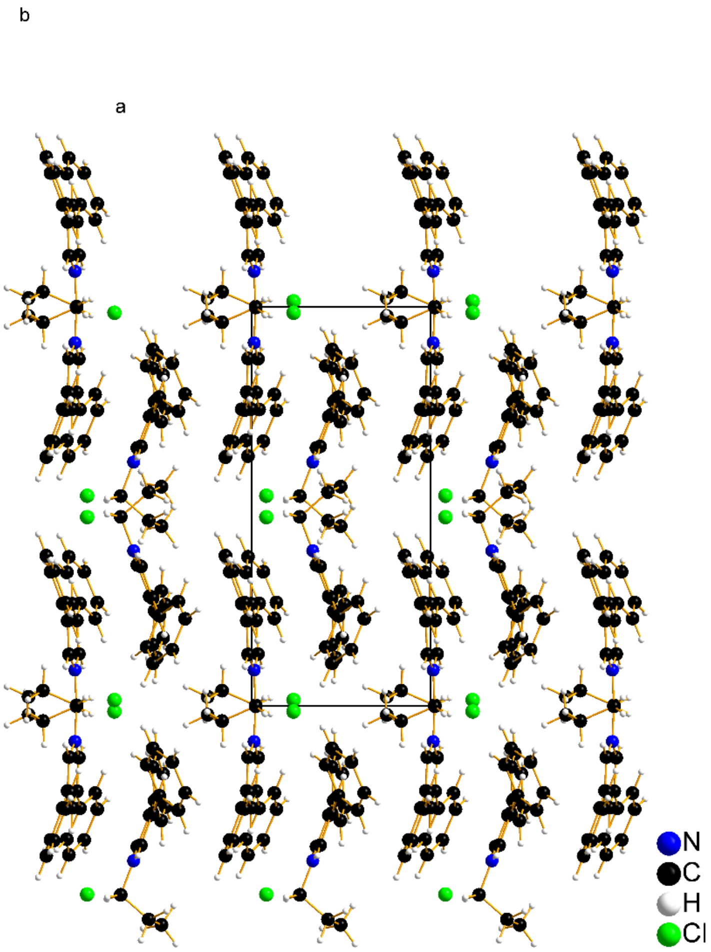

Figure S 26. The crystal packing and the unit cell of **7b** crystal structure viewed along the c-crystallographic axis. Only the major components of disordered atoms are shown for clarity.

Summary of the crystal data and structure refinements

Table S 7. Summary of the crystal data and structure refinements.

Compound	7b
Formula	$C_{20}H_{20}N^{+}$ Cl^{-}
F_w	309.82
T [K]	100.0(1)
Crystal system	monoclinic
Space group	*Pc*
a [Å]	7.41154(3)
b [Å]	16.38991(6)
c [Å]	13.60358(5)
α [°]	90
β [°]	96.3857(3)
γ [°]	90
V [Å^3]	1642.235(11)
Z	4
ρ_{calc} [g cm^{-3}]	1.253
Crystal size [mm]	0.16 × 0.13 × 0.09
Radiation type	Cu Kα
λ [Å]	1.54184
μ [mm−1]	2.003
F(000)	656
ϑ_{max} [°]	76.147
Index ranges	−9 ≤ h ≤ 9<br>−20 ≤ k ≤ 20<br>−17 ≤ l ≤ 17
Reflections collected	61375
Independent reflections	6857
Reflections with [I>2σ(I)]	6822
R_{int}	0.0286
Rsigma	0.0127
Data/restraints/parameters	6852/329/452
S	1.036
R_1, wR_2 [I>2σ(I)]	0.0330, 0.0944
R_1, wR_2 [all data]	0.0330, 0.0945
$\Delta\rho_{min}$, $\Delta\rho_{max}$ [eÅ^{-3}]	−0.209, 0.600
Flack x	BASF = 0.177(12)[a]
CCDC Deposition number[b]	2513694

[a] Refined as an inversion twin. [b] The supplementary crystallographic data for this paper can be obtained free of charge from The Cambridge Crystallographic Data Centre (CCDC) via www.ccdc.cam.ac.uk/structures.

Table S 8. Selected bond distances and angles in the crystal structure of **7b**.

Bond	Value [Å]
N1–C5	1.349(3)
N1–C18	1.490(3)
N1–C1	1.343(3)
N2–C21	1.353(3)
N2–C25	1.344(3)
N2–C37	1.496(3)

Angle	Value [°]
C5–N1–C18	118.77(19)
C1–N1–C5	122.04(19)
C1–N1–C18	119.18(18)
C21–N2–C37	118.7(2)
C25–N2–C21	121.7(2)
C25–N2–C37	119.6(2)

Table S 9. Weak H···Cl contacts of the non-disordered chloride anion in the crystal structure of **7b**.

D–H···A	D–H [Å]	H···A [Å]	D···A [Å]	D–H···A [°]
C23–H23···Cl1	0.95	3.38	3.979(2)	123.0
C31–H31···Cl1	0.95	2.93	3.843(2)	161.4
C5–H5···Cl1	0.95	2.82	3.460(2)	125.6
C20–H20A···Cl1	0.98	3.20	3.978(3)	137.2
C20–H20B···Cl1	0.98	3.14	3.967(3)	142.5
C18–H18A···Cl1	0.99	2.86	3.762(2)	151.9
C18–H18B···Cl1	0.99	2.84	3.731(2)	150.2
C1–H1···Cl1	0.95	2.70	3.576(2)	153.6
C33–H33···Cl1	0.95	2.82	3.732(2)	160.8
C17–H17···Cl1	0.95	2.87	3.808(2)	168.3
C7–H7···Cl1	0.95	2.98	3.909(2)	165.0
C34–H34···Cl1	0.95	3.20	3.845(3)	126.7

Computational studies (Table S10)

Geometry optimizations and harmonic frequency calculations were carried out using the ωB97X-D exchange–correlation functional,[7] and the def2-TZVP basis set,[8] as implemented in Gaussian16 (Revision C.01) program package.[3] Frequency calculations were performed at the corresponding level to confirm that the optimized structure is a genuine minimum (no imaginary frequencies). Population analysis (Hirshfeld fragment charges) was calculated at the same level. Implicit solvation, where appropriate, was modelled using the SMD continuum solvation model. To visualize electrostatic potential surfaces/maps from Gaussian calculations, the GaussView software was used.[4] Quantitative analysis of molecular surface was performed using Multifunctional Wavefunction Analyzer (Multiwfn Version 3.8.).[9]

---

[3] Gaussian 16 (Revision C.01), M. J. Frisch, G. W. Trucks, H. B. Schlegel, G. E. Scuseria, M. A. Robb, J. R. Cheeseman, G. Scalmani, V. Barone, G. A. Petersson, H. Nakatsuji, X. Li, M. Caricato, A. V. Marenich, J. Bloino, B. G. Janesko, R. Gomperts, B. Mennucci, H. P. Hratchian, J. V. Ortiz, A. F. Izmaylov, J. L. Sonnenberg, D. Williams-Young, F. Ding, F. Lipparini, F. Egidi, J. Goings, B. Peng, A. Petrone, T. Henderson, D. Ranasinghe, V. G. Zakrzewski, J. Gao, N. Rega, G. Zheng, W. Liang, M. Hada, M. Ehara, K. Toyota, R. Fukuda, J. Hasegawa, M. Ishida, T. Nakajima, Y. Honda, O. Kitao, H. Nakai, T. Vreven, K. Throssell, J. A. Montgomery, Jr. J. E. Peralta, F. Ogliaro, M. J. Bearpark, J. J. Heyd, E. N. Brothers, K. N. Kudin, V. N. Staroverov, T. A. Keith, R. Kobayashi, J. Normand, K. Raghavachari, A. P. Rendell, J. C. Burant, S. S. Iyengar, J. Tomasi, M. Cossi, J. M. Millam, M. Klene, C. Adamo, R. Cammi, J. W. Ochterski, R. L. Martin, K. Morokuma, O. Farkas, J. B. Foresman, D. J. Fox, Gaussian, Inc. Wallingford CT, 2016)

[4] R. Dennington, T. A. Keith, J. M. Millam, Gausview (Version 6.0.16.), Semichem Inc. Shawnee Mission, KS, 2016.

Table S 10. Hirshfeld charges with H summed into heavy atoms. Fragment charges are sums over the atom-index sets. The "headgroup" is the formal cationic core (*P* for **7a**; pyridinium ring *N*1,*C*2–*C*6 for **7b**/**7c**/**7e**/**7f**; heterocycle *N*1,*C*2,*S*3,*C*4,*C*5 for **7d**). Delocalization is quantified as the fraction of +1 charge residing outside the headgroup.

Cation	*q*(headgroup)	*q*(Pr)	*q*(other)	delocalization %[f]
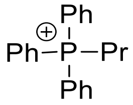 **7a**	0.388	-	0.612[a]	61.2
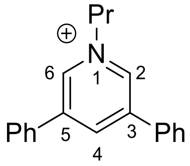 **7b**	0.483	0.248	0.269[b]	51.7
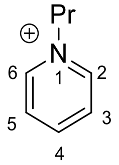 **7c**	0.735	0.265	-	26.5
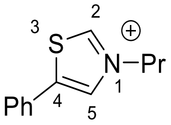 **7d**	0.575	0.260	0.165[c]	42.5
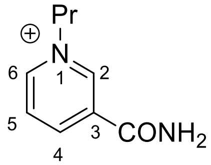 **7e**	0.644	0.265	0.091[d]	35.6
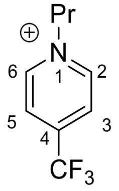 **7f**	0.662	0.275	0.063[e]	33.8

[a] (all non-*P* framework), [b] two phenyl groups, [c] phenyl group, [d] amide fragment net, [e] CF_3 fragment net, [f] 100x(1-*q*(headgroup)). Pr = propyl, Ph = phenyl.

Redundant internal coordinates for cations **7a** – **7f** optimized at the ωB97X-D/def2-TZVP level are given below.

7a

P,0,0.0096994701,0.0441477505,0.8257488446
C,0,-1.524786433,0.7158245394,0.1888631959
C,0,0.2452098814,-1.6311588413,0.234045629
C,0,1.3769987538,1.0650767955,0.2836497322
C,0,-0.0351952598,-0.0017507097,2.6341888848
C,0,-2.4472482004,-0.1047492739,-0.4550811434
C,0,-0.4487668197,-2.6880747684,0.8257234149
C,0,1.2814644765,1.8000230625,-0.8964755662
C,0,-1.8037222144,2.0731150067,0.3679520473
C,0,1.1008940991,-1.8751200326,-0.8378092609
C,0,2.5690457223,1.0617744019,1.00794106
C,0,-3.6451690469,0.4273385357,-0.9051773653
C,0,-0.2834900729,-3.9750595908,0.3463806147
C,0,2.3701314959,2.5310518838,-1.3421931004
C,0,-3.0010974094,2.5950205453,-0.084437632
C,0,1.2602350222,-3.1667970297,-1.3116939968
C,0,3.6505778804,1.7974442404,0.55808608
C,0,-3.9226168879,1.7716738954,-0.7178865173
C,0,0.5703899487,-4.2139697382,-0.7213411523
C,0,3.5507964288,2.531454955,-0.6155324288
H,0,0.8769620319,-0.5136298037,2.953302458
H,0,-0.8674002662,-0.6564857629,2.9041156664
H,0,-2.2336575207,-1.1532722996,-0.6153112892
H,0,-1.1231100995,-2.5168032322,1.6552507682
H,0,0.361310196,1.8056105505,-1.4664493185
H,0,-1.0824227568,2.7254259244,0.8440942355
H,0,1.6496473618,-1.0637656558,-1.2981475924
H,0,2.6628600986,0.4882796724,1.9214628666
H,0,-4.3594275566,-0.2111496355,-1.4074707678
H,0,-0.8202282893,-4.7930128999,0.8079995068
H,0,2.2939465637,3.1024470167,-2.257556651
H,0,-3.214813385,3.6465459652,0.0529921665
H,0,1.9281321625,-3.354380217,-2.1416726599
H,0,4.5726307675,1.7980724563,1.1237155933
H,0,-4.8582877953,2.183848272,-1.0724712293
H,0,0.6995735846,-5.2223582566,-1.0921714574
H,0,4.3984412776,3.106737986,-0.9642936692
C,0,-0.180460305,1.3530798195,3.3289089739
H,0,0.5931551584,2.0378499941,2.9732238264
H,0,-1.1432381149,1.7942550789,3.0651643148
C,0,-0.0833163402,1.2075625762,4.8423065407
H,0,0.8847599873,0.7995006678,5.1383831048
H,0,-0.200987971,2.1754848642,5.3282727856
H,0,-0.8605140844,0.5439802918,5.2253243071

7b

N,0,-0.3738779356,0.4173146724,0.4518168908
C,0,1.1394417818,-1.6054153587,-0.5475919188
C,0,-0.9756549537,-0.6956298588,0.0124140354
C,0,0.9574903179,0.5568101154,0.4045676189
C,0,-0.2434401677,-1.7545357715,-0.4942462856
C,0,1.7659345983,-0.4444039624,-0.1035568578
C,0,-1.2014698978,1.5462557502,0.9310858519
H,0,-0.6354628818,2.0551320659,1.7105992952
H,0,-2.0931347382,1.1229541335,1.3919267293
H,0,1.3581285729,1.4797277373,0.7987493804
C,0,-1.5615885185,2.4957714653,-0.2020860652
H,0,-0.6427544849,2.8735223488,-0.6581211776
H,0,-2.0967585196,1.9413360953,-0.9772498889
C,0,-2.4134196246,3.6530054648,0.3012327213
H,0,-2.6642911966,4.3234977827,-0.5191702142
H,0,-3.3479339723,3.2972906705,0.7383461036
H,0,-1.88531216,4.2351612705,1.0582879827
H,0,1.7427287995,-2.4130488804,-0.9428561634
C,0,3.2314533536,-0.2731052631,-0.1464323138
C,0,4.0704965637,-1.3325883644,0.1918041352
C,0,3.7892864136,0.9458767225,-0.5264033053
C,0,5.4447412451,-1.1719183073,0.154534179
H,0,3.6512089609,-2.2786799586,0.5123657112
C,0,5.1641334072,1.1013392714,-0.5679245097
H,0,3.1506539321,1.7692685602,-0.8241997846
C,0,5.9931261295,0.0437273342,-0.2258530778
H,0,6.0891542591,-1.9964851059,0.428904002
H,0,5.5890390534,2.0474565747,-0.875829574
H,0,7.0676500939,0.1665024731,-0.2571401309
C,0,-0.9240769829,-2.9762151163,-0.9668559691
C,0,-0.5006258001,-3.6048484855,-2.1358106175
C,0,-1.9924820475,-3.5122824403,-0.2509659782
C,0,-1.1399442691,-4.7482756301,-2.5825696381
H,0,0.3133459971,-3.186113902,-2.7152983486
C,0,-2.6259421646,-4.6591656963,-0.6978309464
H,0,-2.3120208938,-3.0544186945,0.6778398291
C,0,-2.2020160111,-5.2771142919,-1.8645074385
H,0,-0.8115572906,-5.2244107288,-3.4968524916
H,0,-3.446756176,-5.0758920029,-0.1293965819
H,0,-2.6987413989,-6.1726826734,-2.2136527389
H,0,-2.0549814739,-0.7102883555,0.0626642404

7c

N,0,1.0681445538,1.3698861603,-0.4434482847
C,0,-1.5131962787,0.4645686401,-0.3276680786
C,0,0.8317931453,0.062017704,-0.2549613296
C,0,0.0538543662,2.2400368523,-0.5692960371
C,0,-0.4538804675,-0.4202463675,-0.1945776924
C,0,-1.2516166273,1.8130642614,-0.5168971029
H,0,-0.6152585148,-1.4783644594,-0.0476048126
C,0,2.4637243039,1.8683435872,-0.4429498889
H,0,2.4998231203,2.727704208,-1.1115774008
H,0,3.0861294751,1.0859031417,-0.8757049032
H,0,0.3258553511,3.2760516943,-0.7141228617
C,0,2.9253801679,2.2347966447,0.9600722584
H,0,2.2571716888,2.9963971125,1.3705467627
H,0,2.8430183617,1.3567579196,1.606055173
C,0,4.3599297796,2.7454944404,0.9470630587
H,0,4.6772048575,3.0033554866,1.9560737052
H,0,5.047442666,1.9895386362,0.5643072413
H,0,4.4581751451,3.6387354191,0.3281102396
H,0,-2.0484976123,2.5342905649,-0.6267306763
H,0,1.7008692883,-0.5733671845,-0.1586033713
H,0,-2.5332271799,0.1061984481,-0.2859572889

7d

C,0,1.2358800525,0.1117432031,0.706402938
H,0,1.3817803165,-0.6719956112,1.4501072838
H,0,1.5283875468,1.0587091556,1.158741328
C,0,2.0332775919,-0.1657623811,-0.5601974233
H,0,1.6908244345,-1.1022537284,-1.0072009575
H,0,1.8346905153,0.6259825634,-1.2867521027
C,0,3.5239038443,-0.2448979018,-0.2590171652

H,0,4.0836144477,-0.4410026113,-1.1720324502
H,0,3.8940974187,0.6903114218,0.1642543689
H,0,3.7447660248,-1.0476573159,0.4463926975
N,0,-0.2120508934,0.1860453142,0.4375692511
C,0,-0.9807676565,-0.9295638206,0.2174369835
C,0,-0.8776810043,1.3146118867,0.3143068629
C,0,-2.2758041341,-0.6458705804,-0.0737101755
H,0,-0.5262677289,-1.9060285652,0.2691046674
H,0,-0.4192829336,2.2825767841,0.4445062039
S,0,-2.4929850032,1.0664956107,-0.0588767039
C,0,-3.3760324305,-1.5720505041,-0.34570419
C,0,-3.4315365394,-2.7924639646,0.3267468396
C,0,-4.3640442619,-1.2500268299,-1.2747875314
C,0,-4.4588749915,-3.6803812765,0.063646576
H,0,-2.687498406,-3.0371558518,1.0746263505
C,0,-5.3916235347,-2.1405997795,-1.5293870627
H,0,-4.3216565162,-0.3144175945,-1.8205108027
C,0,-5.4389682641,-3.3559751154,-0.862974923
H,0,-4.5008679217,-4.6236931378,0.5914721135
H,0,-6.1531032397,-1.8879630124,-2.2548875368
H,0,-6.2434339335,-4.0509608374,-1.0639625396

7e

N,0,-0.0812316757,-0.0400029705,0.3973054223
C,0,-2.7253811365,-0.0907705969,-0.2997990649
C,0,-0.7642260364,1.11023517,0.306760235
C,0,-0.6833952792,-1.2187548044,0.1641431794
C,0,-2.0964326556,1.1128323484,-0.0369189047
C,0,-2.0135045333,-1.2804181554,-0.1805097856
H,0,-2.6240551759,2.0531262174,-0.1057812472
C,0,1.3747839633,-0.0075851266,0.6722428504
H,0,1.6191604383,-0.9113788804,1.229515798
H,0,1.5609870663,0.8442919999,1.3252865582
H,0,-0.0535550723,-2.0952568064,0.2345242423
C,0,2.1829151451,0.0889673329,-0.613710091
H,0,1.9415442872,-0.7630837474,-1.2546953612
H,0,1.8859863215,0.9899070979,-1.1568867296
C,0,3.6766571664,0.1200162383,-0.3195691266
H,0,4.2416291665,0.1901488462,-1.2475358265
H,0,3.9424127294,0.9793094861,0.2982001799
H,0,3.9988625144,-0.7842951186,0.1992201576
H,0,-0.2089577686,2.0153061713,0.5090379395
H,0,-3.762464623,-0.1323093104,-0.6084368082
C,0,-2.71406359,-2.5732582399,-0.5422585169
O,0,-3.5754365061,-2.5468806965,-1.3842034689
N,0,-2.2826128996,-3.690157561,0.0873438561
H,0,-2.7700743372,-4.5460304702,-0.1249641049
H,0,-1.7877066289,-3.6511804739,0.9600102275

7f

N,0,-0.1369644951,-0.0006181076,0.3697519638
C,0,-2.7775425125,-0.0006595566,-0.3405585231
C,0,-0.77634084,1.1656541101,0.1946464725
C,0,-0.776215771,-1.1666305794,0.1942806423
C,0,-2.1034402008,1.1971057271,-0.1602282054
C,0,-2.1035844449,-1.1981982265,-0.1606174953
H,0,-2.5968602798,2.1490533179,-0.2953877928
C,0,1.3112968637,-0.0005260109,0.689879647
H,0,1.505744985,-0.8810672635,1.3010643466
H,0,1.5052343389,0.8789256462,1.3028015577
H,0,-0.1934096855,-2.0638760001,0.3472905915
C,0,2.1598213546,0.0009524007,-0.5737349439
H,0,1.9105342902,-0.8772563937,-1.1751357047
H,0,1.9100688639,0.8801859466,-1.1734437389
C,0,3.6440782089,0.0010346564,-0.2328625786
H,0,4.2388521365,0.0021047508,-1.1446762904
H,0,3.9190521644,0.88427578,0.3457423457
H,0,3.919549499,-0.8831952275,0.343992259
H,0,-0.1935270045,2.0628249149,0.3480065576
H,0,-2.5969102104,-2.1500673822,-0.2960260757
C,0,-4.2602030204,0.0017410959,-0.6909345351
F,0,-4.5685055555,1.0660952228,-1.4220433181
F,0,-4.9756122437,0.0351495146,0.4304583491
F,0,-4.5865178911,-1.0912117361,-1.369709640

Mitochondrial membrane potential (Figures S 27–S28)

JC-10 Assay

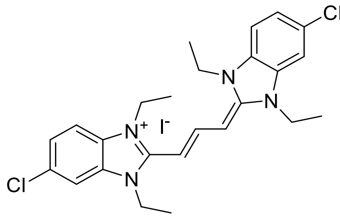

JC-10

The JC-10 mitochondrial membrane potential assay kit (Abcam Limited, catalog number: ab112134) was used according to the manufacturer's protocol to assess changes in mitochondrial membrane potential following the addition of test compounds **6a-f**, **8a-b and 9a-b**. Briefly, COLO-357 cells were seeded in a Costar 96-well clear-bottom black-sided plate (Corning Costar Corporation, New York, NY, USA) at a density of 20,000 cells per well. After 24 hours, 1 µM and 10 µM test compounds were added to the cells, followed by the addition of JC-10 reagent for 30 minutes at 37 °C. After a total of 45 minutes' exposure to the test compounds, fluorescence was measured at 490/525 nm and 540/590 nm (excitation/emission) using a microplate reader (Synergy MX, BioTek, Winooski, VT, USA). Mitochondrial membrane potential was calculated as follows: (ratio of 590 nm/525 nm in treated cells) × 100% / (ratio of 590 nm/525 nm in control cells). Vehicle control (0.15 % DMSO) treated cells were considered as control cells.

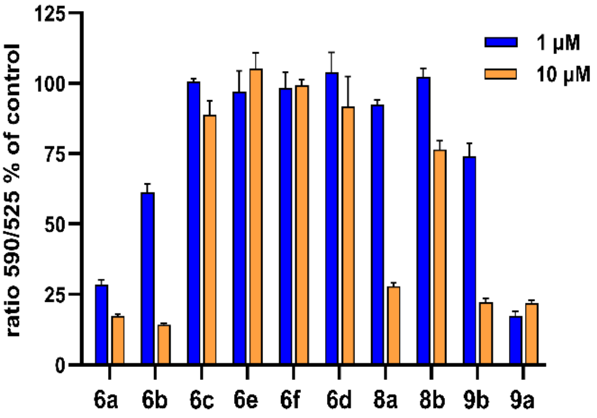

Figure S 27. Effect of bioactive and inert cargo conjugates on ΔΨm. Mitochondrial membrane potential was measured using the JC-10 assay kit after 45 min exposure of COLO-357 cells to 1 and 10 µM concentrations of test compounds. The ratios of fluorescence emitted at 590 nm and 525 nm were normalised to those of control (untreated) cells. The assay was conducted in a single biological replicate, with four technical replicates for each test condition. The error bar represents the standard deviation.

TMRM Fluorescence Measurements Using Automated Microscopy.

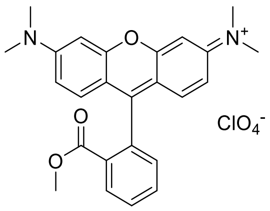

TMRM

Measurements of mitochondrial inner membrane depolarization upon exposure to test compounds were performed as described by Vianello et al.,[14] with minor modifications. COLO-357 cells were seeded at a density of 20,000 cells per well in a Costar 96-well clear-bottom black-sided plate (Corning Costar Corporation, New York, NY, USA) and allowed to attach for 24 hours. The cells were then treated simultaneously with 20 nM TMRM (tetramethylrhodamine methyl ester; Thermo Fisher Scientific, Waltham, MA, USA), 4 µM CSH (Cyclosporin H; Sigma-Aldrich, St. Louis, MO, USA), and 2 µM Hoechst 33342 (Thermo Fisher Scientific, Waltham, MA, USA), all dissolved in HBSS (Hank's Buffered Salt Solution; Thermo Fisher Scientific, Waltham, MA, USA), 100 µl per well, and incubated at 37°C for 40 minutes. Ten minutes before and at the end of incubation, basal fluorescence was acquired using 10x magnification objectives for Hoechst 33342 (Ex: 377/50, Em: 447/60 nm) and TMRM (Ex: 531/40, Em: 593/40 nm) with a Cytation 5 imaging reader and Gen5 software (BioTek, Winooski, VT, USA). After acquisition of basal fluorescence intensity, cells were treated with 1, 10, and 25 µM **6a, 6b, 8a, 8b 9a, and 9b**, **VC** (vehicle control; 0.25% DMSO), and 25 µM **FCCP** (carbonyl cyanide *p*-(trifluoromethoxy)phenylhydrazone; Thermo Fisher Scientific, Waltham, MA, USA), and images were acquired sequentially for both channels every 10 minutes for one hour. Experiments were repeated independently three times. For analysis, image segmentation was performed by detecting regions of interest (ROI; each ROI corresponding to a single cell) on the Hoechst33342 channel. Background-corrected TMRM fluorescence intensity was then measured for each ROI and averaged.

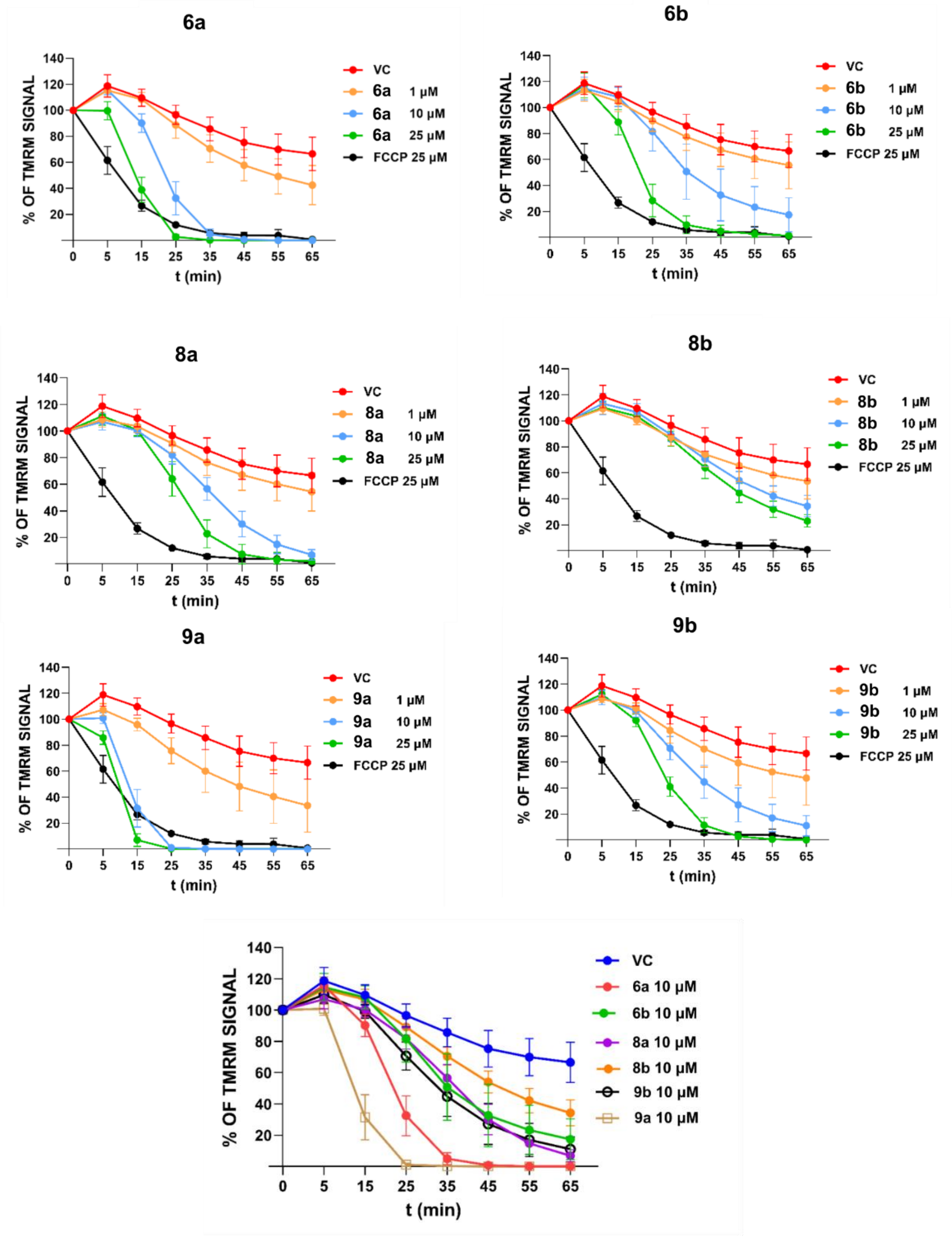

Figure S 28. Effects of tested compounds on mitochondrial function. Measurements of the mitochondrial inner membrane depolarization upon addition of test compounds were performed using the TMRM assay. FCCP was used as a positive control. The assay was conducted in three biological replicates, with three technical replicates for each test condition. The error bar represents the standard deviation.

Mitochondrial permeability transition pore opening (Figure S29)

Direct assessment of mitochondrial permeability transition pore (mPTP) opening in COLO357 cells was conducted using a protocol optimized for this cell line. Cells were incubated with calcein-AM (Sigma-Aldrich) for 40 min at 37 °C, then transferred into PSS supplemented with 2 mM Ca^{2+} and 100 µM $CoCl_2$. Under these conditions, cobalt selectively quenches cytosolic calcein, enabling specific monitoring of mitochondrial calcein fluorescence.

Mitochondrial calcium overload was induced by the addition of ionomycin (10 µM). Upon mPTP opening, cobalt penetrates the mitochondria and quenches the calcein signal, resulting in a characteristic fluorescence decline. mPTP activation was quantified by calculating the fluorescence decay slope between 100 and 200 seconds. Fluorescence emission was recorded at 515 nm using the FlexStation-3 system.

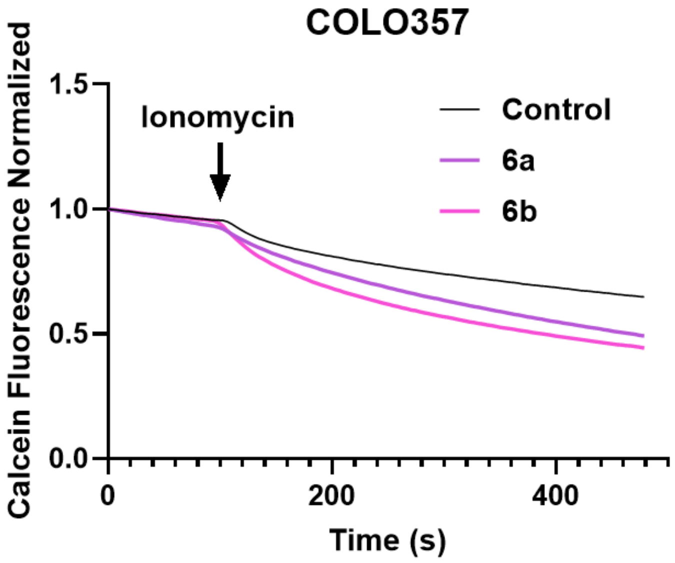

Figure S 29. Graph showing PTP opening following stimulation with 10 µM ionomycin in COLO357 cells pre-treated or not with conjugates **6a** and **6b**, or with DMSO.

Determination of Caspase 3/7 activity,apoptosis assay (Figure S30)

Apoptosis induction was evaluated through caspase-3/7 activity in live cell imaging using a cell-permeable reporter, Incucyte caspase-3/7 Green Dye (Sartorius; Green 4440). The probe incorporates a caspase-3/7 recognition motif (DEVD) that, when cleaved, releases a DNA intercalating dye that exhibits fluorescence upon binding to DNA. The presence of activated caspase-3/7 in the cytosol (initiation of apoptosis) results in a fluorescent nuclear signal.

The apoptosis after treatments was monitored by adding caspase-3/7 reagent, which was diluted at a ratio of 1:1000 (v:v) in the final volume of the culture medium (100 µL for monolayer culture and 150 µL for spheroid culture). The activation of caspase-3/7 was monitored by capturing fluorescence images over time using an Incucyte system under standard culture conditions (37°C, 5% CO2). The images were analyzed using the Incucyte Zoom software, which generated a mask for confluence and fluorescence signals through machine learning. The algorithm was trained using a set of 6–12 representative images where the sensitivity to cells vs. background, hole filling, minimal and maximal area were set for phase contrast analysis, and threshold, eccentricity and background subtraction in fluorescent signals were adjusted to optimize detection. Once trained, the detection algorithm was applied to each image throughout. The green object count (number of apoptotic cells) was normalized to culture confluence using Prism software. All treatments were performed in technical quadruplicates and biological triplicates.

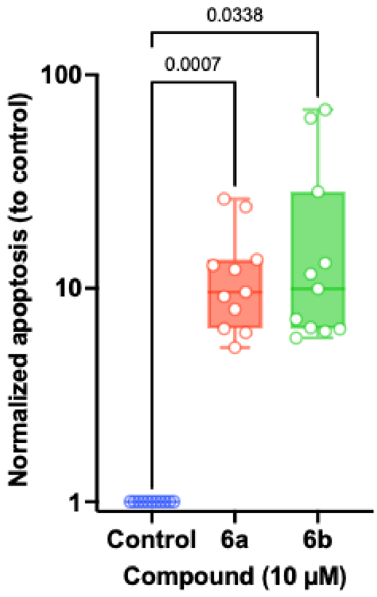

Figure S 30. Apoptosis induction by compounds **6a** and **6b** (10 µM) in COLO-357 cells after 48 h of addition of the compounds. The values are presented as normalized to the control (N=3, n=4)—one-way ANOVA with Dunnett's test.

Seahorse Extracellular flux real-time metabolic analysis (Figures S31–S35)

COLO-357 cells were plated in complete Advanced RPMI medium on Seahorse 24-well cell culture plates at 30,000 cells per well. C2C12 cells were plated in complete DMEM medium on Seahorse 24-well cell culture plates at 15,000 cells per well. After overnight incubation, the medium was removed and replaced with 500 µL of corresponding RPMI-based (COLO-357) or DMEM-based (C2C12) Seahorse medium (Seahorse RPMI/Seahorse DMEM, 11,1 mM glucose, 2 mM glutamine, 1 mM pyruvate). The Seahorse medium was replaced again and the cells incubated in a hydrated cell culture incubator without CO_2 for 45 min to 60 min prior to measurement.

Following this, a modified Seahorse Mito Stress assay was performed using the Seahorse XFe24 analyzer to measure the oxygen consumption rate (OCR) in real time. First, 5 baseline measurements were obtained. For the acute OCR response assay, the investigated compounds (**6a, 6b, 8a, 8b, 9a, 9b**) were then injected at 1 µM or 5 µM and the acute OCR response followed for 60 min. Following this, 0.5 µM oligomycin, 1 µM (COLO-357) or 2 µM (C2C12) carbonyl cyanide-p-trifluoromethoxyphenylhydrazone (FCCP) and 0.5 µM rotenone + 0.5 µM antimycin A (AA) were injected sequentially with three measurements obtained after each of these three injections. The lowest OCR value before oligomycin injection was used to assess maximum acute electron transfer chain inhibition at 60 min. Two independent experiments were performed for each cell line.

For the uncoupling assay, the ATP-synthase dependent OCR was first blocked by injecting 0.5 µM oligomycin, followed by injection of 1 µM or 5 µM of investigated compounds (**6a, 6b, 8a, 8b, 9a, 9b**). The OCR response was then followed for 60 min, after which 1 µM (COLO-357) or 2 µM (C2C12) FCCP and 0.5 µM rotenone + 0.5 µM AA were injected sequentially and, again, three measurements obtained after each injection. The maximum uncoupling OCR was defined as the highest OCR before FCCP injection. Two independent experiments were performed for each cell line.

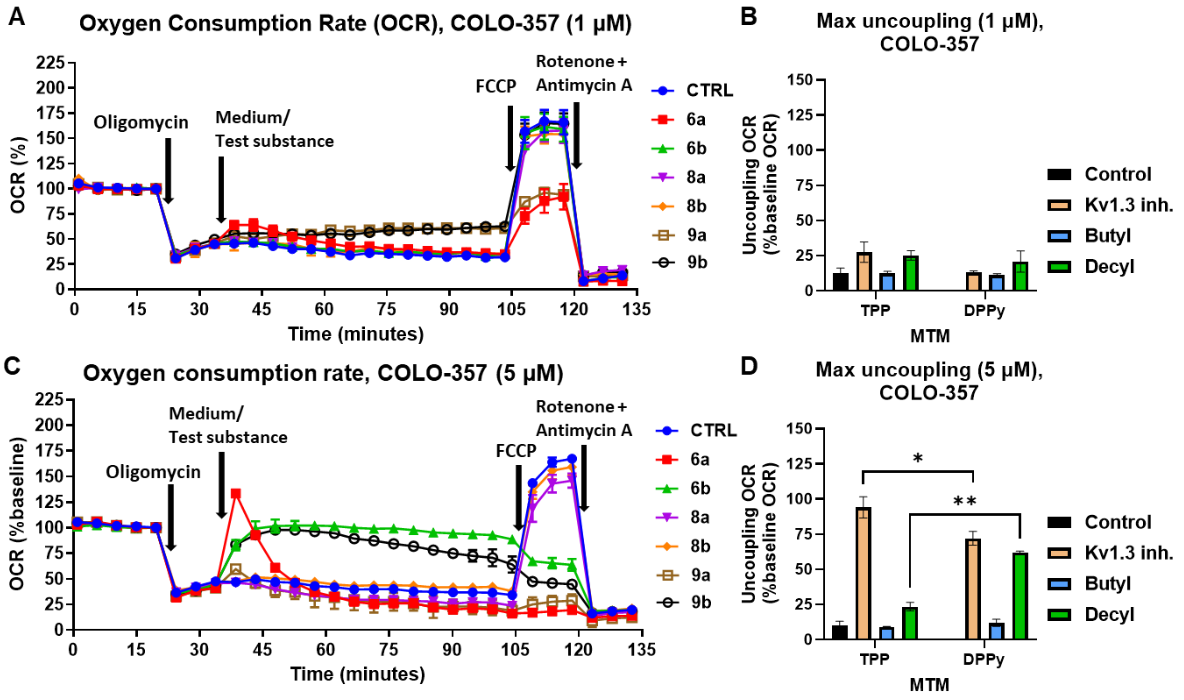

Figure S 31. Seahorse uncoupling assay in COLO-357 cells. The ATP-dependent oxygen consumption was first blocked with oligomycin, so any increase in oxygen consumption rate (OCR) after compound injection denotes uncoupling activity of the injected compounds. Both Kv1.3 inhibitors **6a** and **6b** induced uncoupling in COLO-357 cells, but the effect of **6a** was faster, more transient and occurred at lower concentration than **6b**. The effect of decyl conjugate **9b** was very similar to Kv1.3 inhibitor **6b**. While no major increase in OCR was observed for the TPP^+ decyl conjugate **9a**, this might be due to concurrent inhibition of the electron transfer chain as evidenced by the substantially reduced OCR following FCCP injection (i.e. no increase in OCR after the addition of a known uncoupler). The butyl conjugates **8a** and **8b** did not induce uncoupling at 5 µM. A representative time-course of OCR normalized to baseline is shown in (**A**) and (**C**) for 1 µM and 5 µM compounds, respectively. The maximum uncoupling effect (maximum OCR after compound injection) of 1 µM and 5 µM compounds is shown in (**B**) and (**D**), respectively, as the mean ± SEM of two independent experiments. *p<0.05, **p<0.01 as determined by two-way ANOVA with Šidak's post-hoc test.

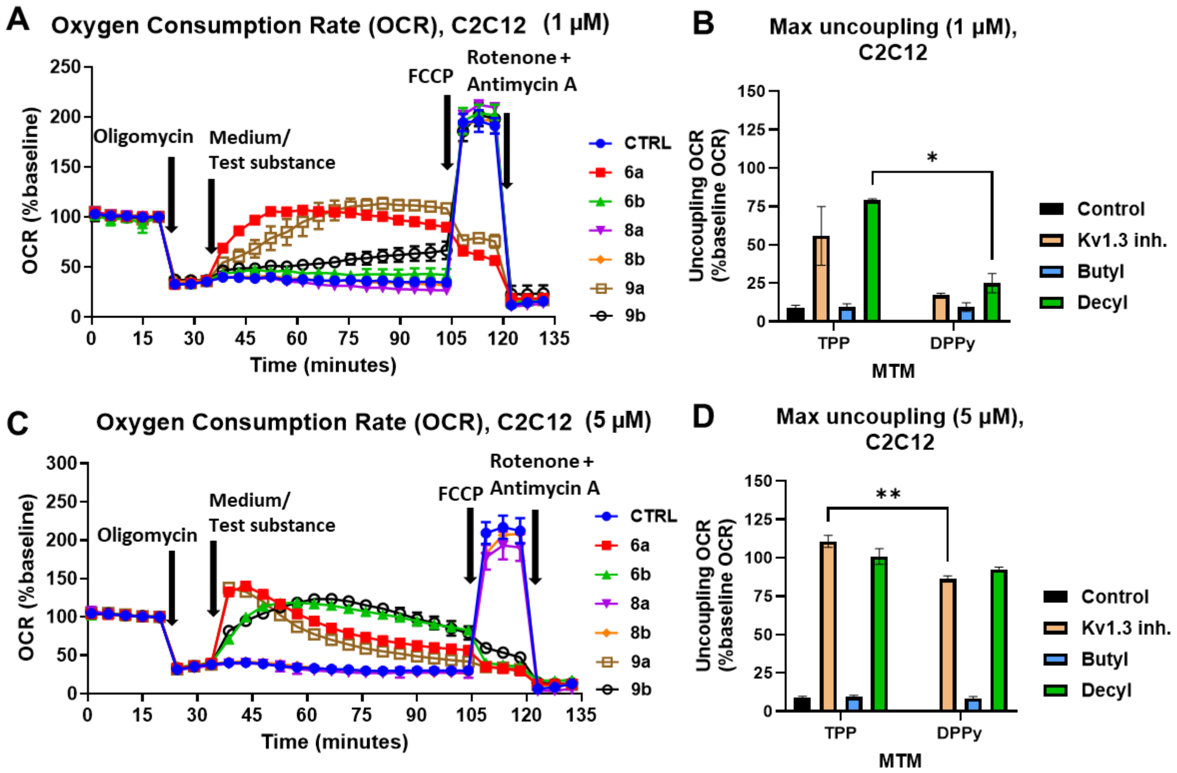

Figure S 32. Seahorse uncoupling assay in C2C12 cells. The ATP-dependent oxygen consumption was first blocked with oligomycin, so any increase in oxygen consumption rate (OCR) after compound injection denotes uncoupling activity of the injected compounds. Both Kv1.3 inhibitors **6a** and **6b** induced uncoupling in C2C12 cells, but the effect of **6a** was faster, more transient and occurred at lower concentration than **6b**. The effect of decyl conjugates **9a** and **9b** was very similar to their Kv1.3 inhibitor analogues with the same MTM (**9a** and **9b**, respectively). The butyl conjugates **8a** and **8b** did not induce uncoupling at 5 µM. A representative time-course of OCR normalized to baseline is shown in (**A**) and (**C**) for 1 µM and 5 µM compounds, respectively. The maximum uncoupling effect (maximum OCR after compound injection) of 1 µM and 5 µM compounds is shown in (**B**) and (**D**), respectively, as the mean ± SEM of two independent experiments. *p<0.05, **p<0.01 as determined by two-way ANOVA with Šidak’s post-hoc test.

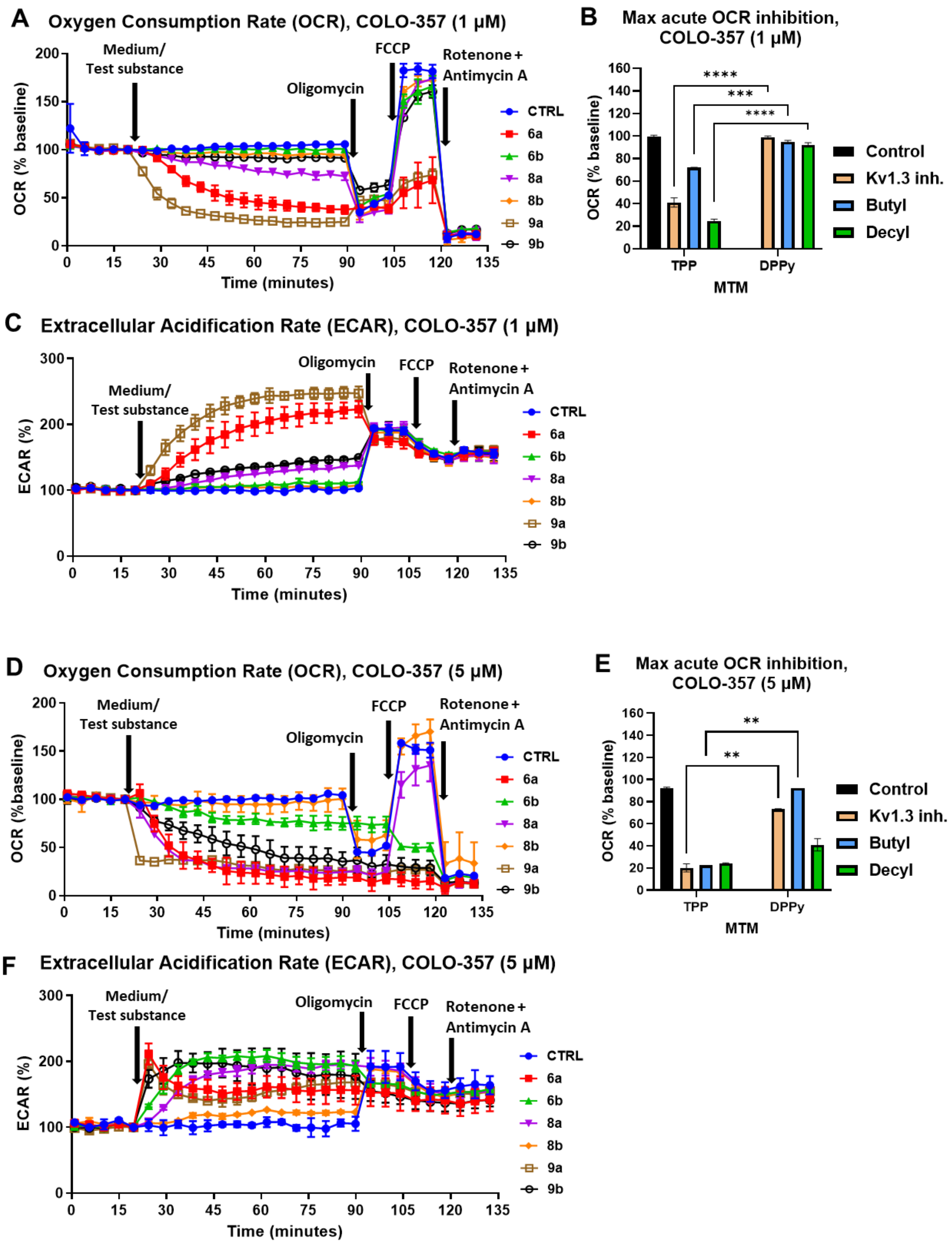

Figure S 33. Seahorse acute OCR inhibition assay in COLO-357 cells. The indicated substances were injected after 5 baseline measurements and the OCR response followed for 1 h. Only Kv1.3 inhibitor **6b** exhibited a noticeable transient increase in OCR, but all the TPP⁺ conjugates (**6a, 8a, 9a**) exhibited rapid acute inhibition of OCR, apparent already at 1 µM, including the supposedly most inert butyl conjugate **8a**. The OCR inhibition was particularly rapid for decyl conjugate **9a**. In contrast, DPPy⁺ conjugates did not notably inhibit OCR within 1 h of treatment at 1 µM. Even at 5 µM, the OCR inhibition was more gradual and weaker for Kv1.3 inhibitor **6b** and decyl conjugate **9b** compared to their TPP⁺ counterparts. Promisingly, the DPPy⁺ butyl conjugate **8b** did not show any OCR inhibition even at 5 µM. To confirm the effects of the investigated compounds on maximal respiration, oligomycin, FCCP and rotenone + antimycin A were injected sequentially as indicated. For all compounds, ECAR remained elevated compared to baseline, indicating specific disruption of mitochondria as opposed to non-specific cytotoxicity. A representative time-course of OCR (**A**, **D**) and ECAR (**C**, **F**) normalized to baseline is shown for 1 µM (**A**, **C**) and 5 µM (**D**, **F**) compounds. The maximum OCR inhibition effect (minimum OCR after compound injection prior to oligomycin injection) of 1 µM and 5 µM compounds is shown in (**B**) and (**E**), respectively, as the mean ± SEM of two independent experiments. *p<0.05, **p<0.01, ***p<0.001, ****p<0.0001 as determined by two-way ANOVA with Šidak's post-hoc test.

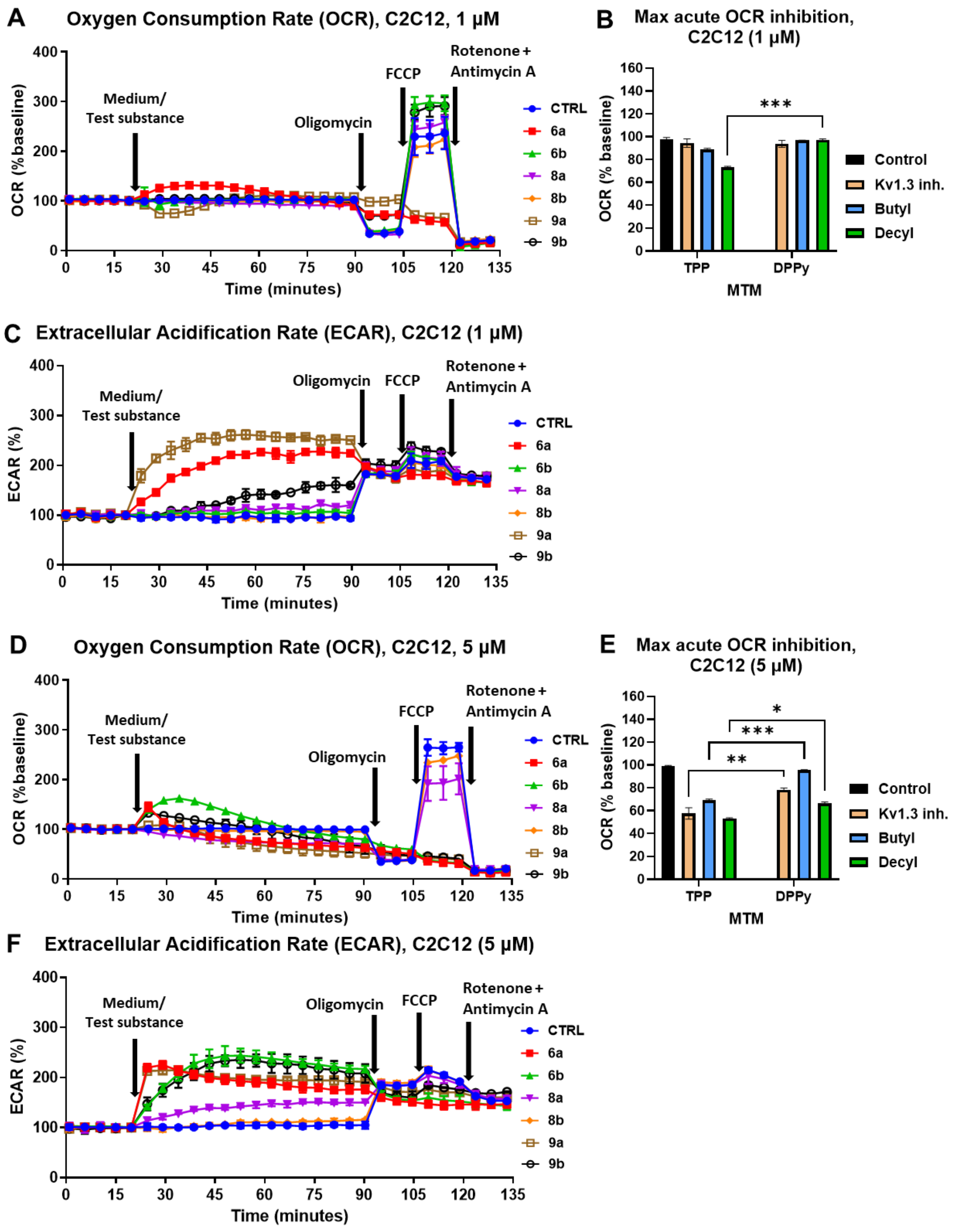

Figure S 34. Seahorse acute OCR inhibition assay in C2C12 mouse myoblast cells. The indicated substances were injected after 5 baseline measurements and the OCR response followed for 1 h. The Kv1.3 inhibitor **6a** exhibited a noticeable transient increase in OCR at 1 µM, while the Kv1.3 inhibitor **6b** required 5 µM for this effect, but showed a more sustained OCR increase. A similar effect was observed for decyl conjugate **9b**. As in COLO-357 cells, the TPP⁺ conjugates were stronger and/or faster and more potent inhibitors of respiration than their DPPy⁺ counterparts. Again, substantial acute OCR inhibition was observed even for supposedly inert TPP⁺ butyl conjugate **8a**, while its DPP⁺ analog **8b** did not show any inhibition of respiration. To confirm the effects of the investigated compounds on maximal respiration, oligomycin, FCCP and rotenone + antimycin A were injected sequentially as indicated. For all compounds, ECAR remained elevated compared to baseline, indicating specific disruption of mitochondria as opposed to non-specific cytotoxicity. A representative time-course of OCR (**A**, **D**) and ECAR (**C**, **F**) normalized to baseline is shown for 1 µM (**A**, **C**) and 5 µM (**D**, **F**) compounds. The maximum OCR inhibition effect (minimum OCR after compound injection prior to oligomycin injection) of 1 µM and 5 µM compounds is shown in (**B**) and (**E**), respectively, as the mean ± SEM of two independent experiments. *$p<0.05$, **$p<0.01$, ***$p<0.001$ as determined by two-way ANOVA with Šidak's post-hoc test.

In light of the differential effects of butyl-substituted TPP+ and DPPy+ derivatives **8a** and **8b** on acute mitochondrial respiration in COLO-357 at 5 µM (Figure S33), we sought to determine whether inhibition of Complex I contributes to this behavior. TPP+ alkyl derivatives have been described to inhibit the function of individual respiratory complexes, with Complex I (as opposed to Complex II) the most sensistive to the TPP+-alkyl conjugates with shorter alkyl chains.[10] Additionally, longer duration treatment with TPP+ derivatives was shown to reduce Complex I protein levels.[11] We therefore investigated the effect of butyl derivatives of TPP+ and DPPy+ on Complex I function as previously described.[12] COLO-357 cells were pre-treated with 5 µM **8a** or **8b** in Seahorse assay medium for 30 min, after which the medium was replaced with MAS-BSA buffer (MAS: 220 mM mannitol, 70 mM sucrose, 10 mM KH2PO4, 5 mM MgCl2, 2 mM HEPES, 1 mM EGTA, 4 mg/mL BSA, pH = 7.20) with the same compound concentration for 10 min. OCR was then measured with Seahorse analyzer. After 5 cycles, the cells were selectively permeabilized with 40 µg/mL saponin, and Complex I specific substrates (10 mM pyruvate, 5 mM malate and 1 mM ADP) were added at the same time. Three OCR measurements were obtained, after which 1.5 µM oligomycin and 1 µM rotenone were injected sequentially, with three measurements after each. Complex I-dependent respiration was calculated as the difference between the maximum OCR after addition of complex I substrates and the minimum OCR following rotenone injection. Two independent experiments were performed (Figure S35).

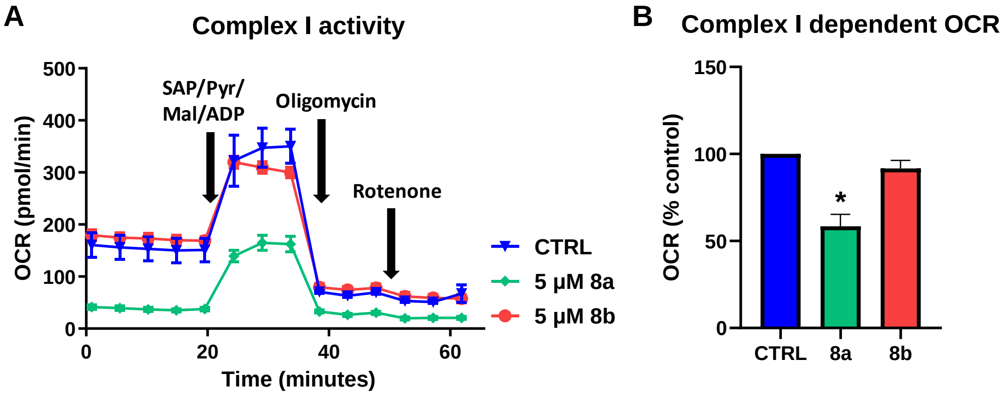

Figure S 35. Complex I activity in COLO-357 cells. The cells were pre-treated with **8a** and **8b** as described above. The TPP+-butyl conjugate **8a** reduced complex I dependent respiration by about 40%, while DPP+-butyl conjugate **8b** did not significantly reduce complex I dependent respiration at 5 µM. The effect of **8a** on respiration was already noticable prior to selective cell membrane permeabilization and addition of complex I substrates, which indicates the involvement of additional unidentified cellular targets. Notably, this effect was also not observed for DPP+-based **8b**. A representative time-course of OCR is shown in **A**. The complex I dependent OCR (maximum OCR after the first injection minus the minimum OCR after the last injection) is shown in (**B**) as the mean ± SEM of two independent experiments. *p<0.05 as determined by one-way ANOVA with Dunnett's post-hoc test.

Cancer selectivity in pancreatic 2D models (Figures S36–39)

Mitochondrial membrane potential and ROS production

15000 COLO357 or 20000 HPDE cells/well were seeded in glass-bottom 96-well cell imaging plates in standard culture medium and allowed to grow for 24 h. Cells were then incubated with either Tetramethylrhodamine (TMRM) (ThermoFisher) (5 nM for COLO357 and 10 nM for HPDE) or 1 µM mitoSOX Red (ThermoFisher) in HBSS for 20 min at 37 °C. The medium was then replaced with HBSS (supplemented with 2 nM TMRM for COLO357 or 3 nM for HPDE in the case of membrane potential determinations) and cells were imaged for 10 minutes using the Operetta system (Perkin Elmer). After 10 minutes, cells were treated with different compounds at the indicated concentrations and images were acquired every 5 min for 40 min. Five to seven fields/well were analyzed. Each condition was tested in triplicate. Analysis was performed using Harmony high-content analysis software.

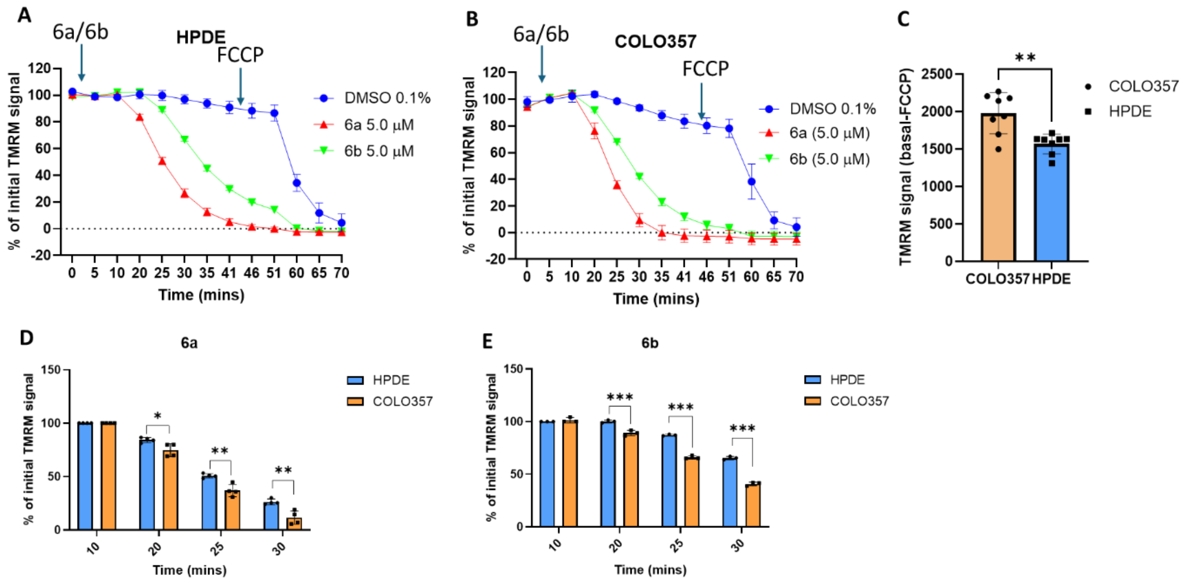

Figure S 36. (A, B) Mitochondrial membrane potential measurments as a function of time (n=3 biological replicates). Error bars represent SD. (C) TMRM signal under basal conditions (average of values measured for the first 3 points of the untreated samples) minus average signal after FCCP addition (last 2 time points). T-test, ** p<0.05. (D, E) Quantification of the traces shown in A and B at the indicated timepoints. ANOVA test was employed for statistics. *p<0.05; ***p<0.01.

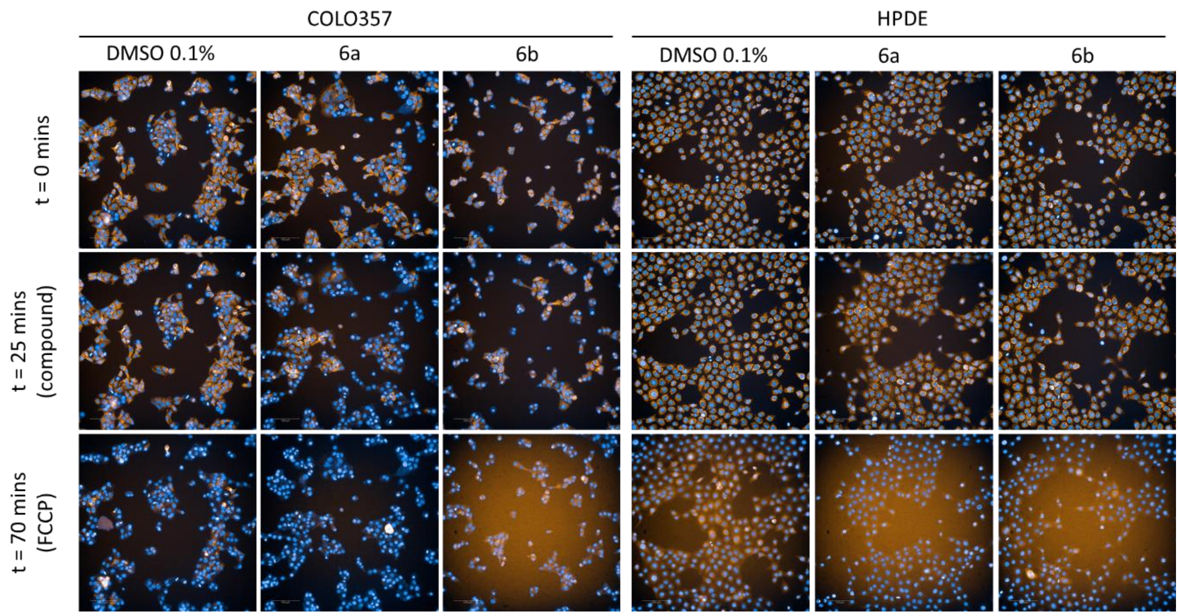

Figure S 37. Representative images acquired with Operetta. Red: TMRM, Blue: Hoechts to stain nuclues.

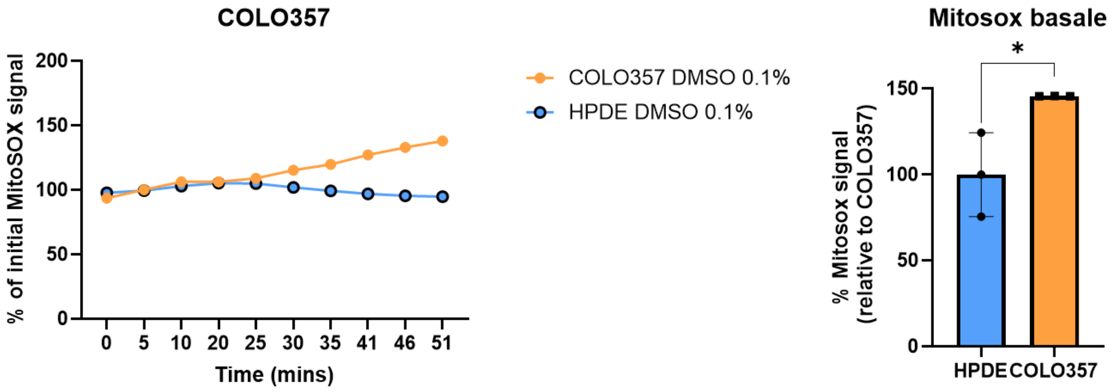

Figure S 38. Left: normalized MitoSOX values from a representative exp. in basal conditions (without treatment) (n=3 biological replicates). Right: The graph refers to the values at 51 minutes minus the value at 0 time point, so % is normalized to the initial values for both cell lines.

Cell death assay

For Sytox assays, 15000 COLO-357 or 20000 HPDE cells/well were seeded in black glass-bottom 96-well cell imaging plates in standard culture medium and allowed to grow for 24h. Cells were then treated with different compounds at the indicated concentrations. After 24h, cells were incubated for 10 minutes at 37°C with 150 µL of HBSS supplemented with Hoechst, 5 µM SYTOX green (Thermofisher, S7020). Cells treated with 0,1% Triton-X were used as a positive control for cell death. Fluorescence signals were measured using Biotek Synergy H1 plate reader.

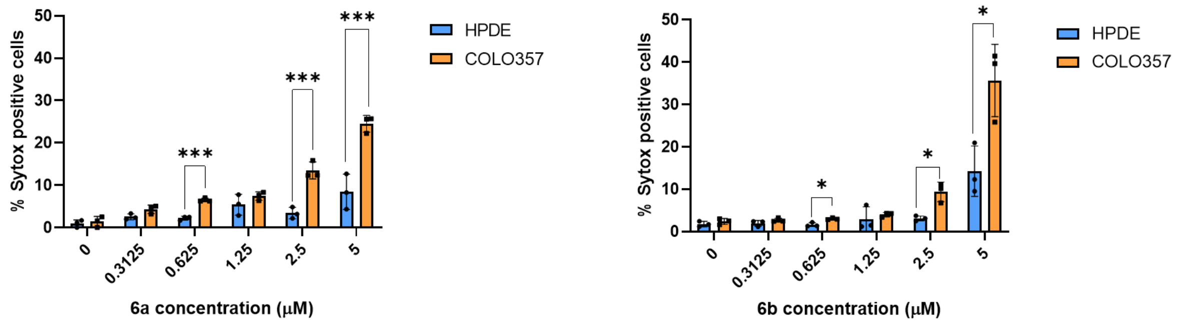

Figure S 39. Immortalized non-tumoral HPDE or tumoral COLO-357 cells were incubated for 24 hours with **6a** and **6b** at the indicated concentrations and cell death was measured using Sytox.

3-dimensional cell cultures (Figures S40–42)

Spheroids

The COLO-357 cells were cultured in RPMI-1640 (Gibco, Thermo Fischer Scientific, Waltham, MA, USA) supplemented with 10 % heat inactivated fetal bovine serum (Gibco, Thermo Fischer Scientific, Waltham, MA, USA), 100 U/mL penicillin and 100 U/mL streptomycin (Sigma-Aldrich, St. Louis, MO, USA), and 1 % GlutaMAX (Gibco, Thermo Fischer Scientific, Waltham, MA, USA). PANC-1 cells were cultured in DMEM/F-12 + GlutaMAX (Gibco, Thermo Fischer Scientific, Waltham, MA, USA) supplemented with 10 % heat inactivated fetal bovine serum (Gibco, Thermo Fischer Scientific, Waltham, MA, USA), 100 U/mL penicillin and 100 U/mL streptomycin (Sigma-Aldrich, St. Louis, MO, USA). Cells were harvested when they reached 80 - 90 % confluency, and their count was determined. Cells were seeded in round bottom Ultra-Low Attachment Microplate (Corning #7007), a total of 10,000 cells in 100 µL of medium supplemented with 2 % Matrigel were prepared. All steps involving Matrigel were performed on ice due to its rapid solidification at RT. Formation of spheroids was monitored in real-time using an Incucyte systems: Incucyte Zoom and Incucyte SX5. After approximately 24 hours, well-defined and compact spheroids were successfully formed. 100 µL of medium containing the compound tested and Incucyte Cytotox Dye (1:4000 (v:v) final dilution, Sartorius, Göttingen, Germany; Green 4633) were added to formed spheroids. Incucyte Cytotox Dye is fluorescent when bound to the DNA and can only enter cells that have compromised cell membranes, thereby reporting cytotoxicity. The induction of cytotoxicity was monitored by capturing fluorescence and phase images every two hours using two different Incucyte systems: Incucyte Zoom and Incucyte Zoom and Incucyte SX5 under standard culture conditions (37 °C in a 5% CO_2 atmosphere). The acquired images were analysed using the Incucyte software. Statistical analysis was later done in GraphPad Prism 10.3.1.

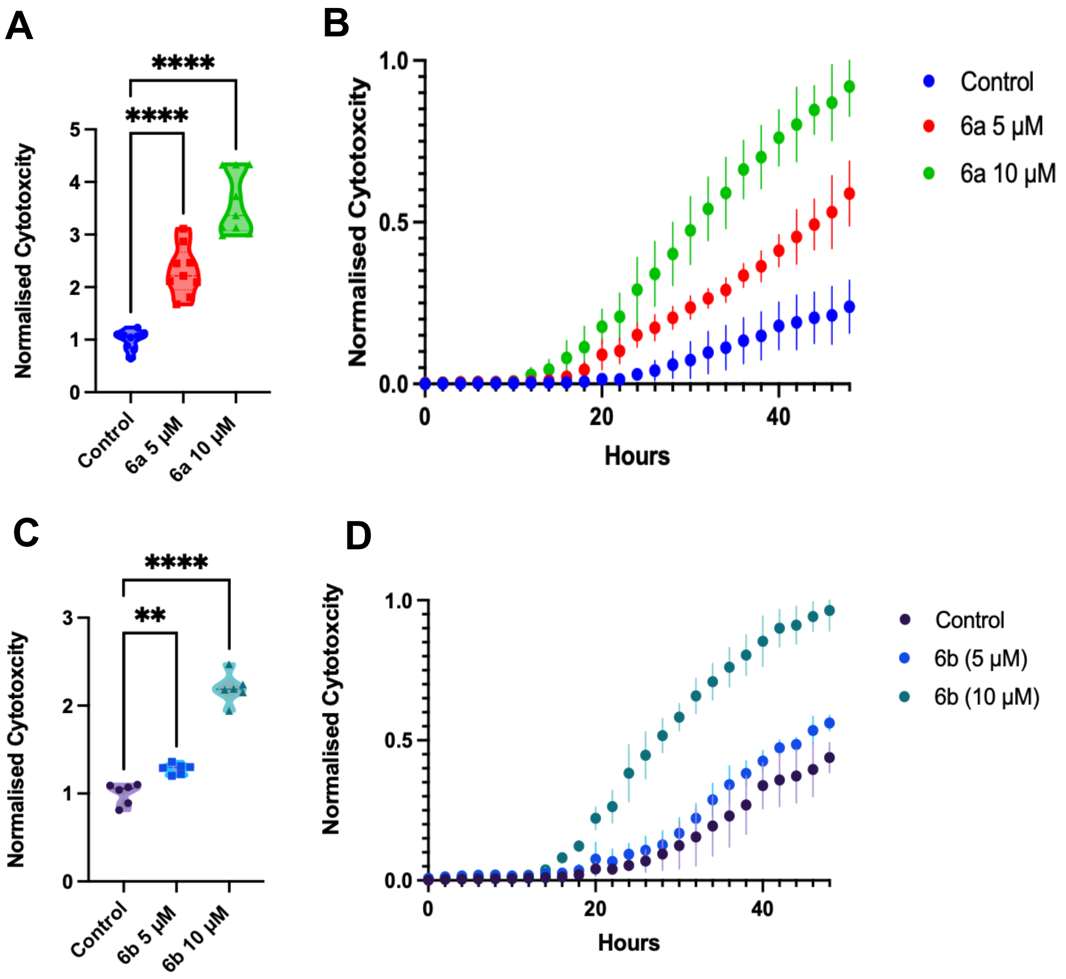

Figure S 40. Efficacies of cytotoxicity induction by compounds 6a and 6b on COLO-357 spheroids. Efficacy of compounds **6a** and **6b** was investigated on COLO-357 spheroids in the presence of green cytotoxicity regent. Compounds solvent DMSO (0.5 %) was added as a control. **A, C**: Representative data of the cytotoxicity of the compounds after 48 hours of treatment with compounds 6a and 6b in concentrations 5 µM and 10 µM. Cytotoxicity was quantified by total green object integrated intensity (GCU × µm²/image) and normalized to the mean value of the control group in each separate experiment. **B, D**: Representative data of the time course of the cytotoxicity induction by compounds **6a** and **6b** in concentrations 5 µM and 10 µM. Cytotoxicity was quantified as the total green object integrated intensity (GCU × µm²/image) and normalized to the highest value measured at 48 hours. Error bars represent mean ± SD of at least two biological replicates (N=2) made in triplicates (n=3). Statistical significance was determined using one-way ANOVA with Dunnett's multiple comparisons test, comparing each treatment to control at the respective time point (****$p < 0.0001$; **$p < 0.01$).

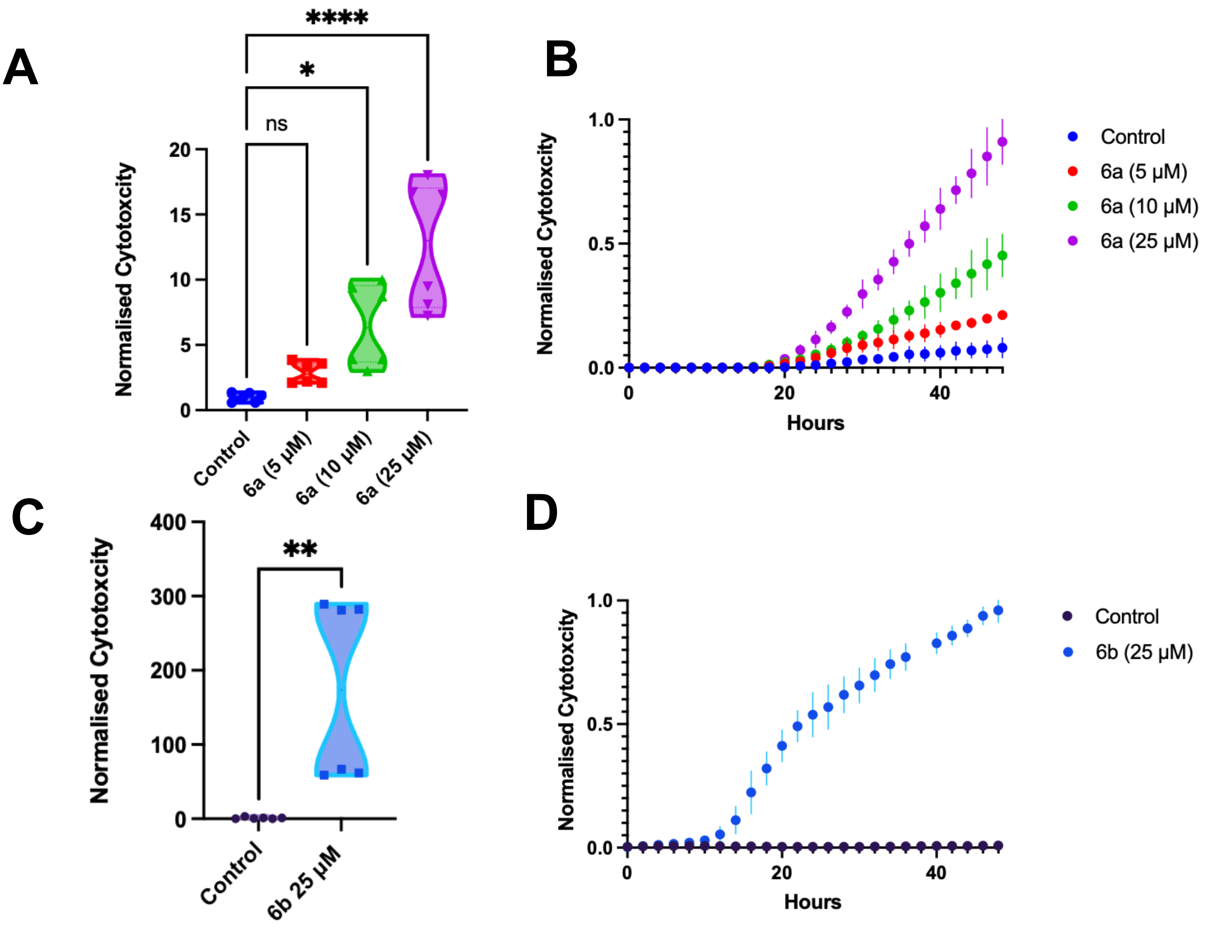

Figure S 41. Efficacies of cytotoxicity induction by compounds 6a and 6b on PANC-1 spheroids. Efficacy of compounds **6a** and **6b** was investigated on PANC-1 spheroids in the presence of green cytotoxicity regent. Compounds solvent DMSO (0,5 %) was added as a control. **A, C**: Representative data of the cytotoxicity of the compounds after 48 hours of treatment with compounds **6a** and **6b** in concentration 25 µM. Cytotoxicity was quantified by total green object integrated intensity (GCU × µm²/image) and normalized to the mean value of the control group in each separate experiment. The cytotoxicity was determined by the total integrated intensity (GCU x μm^2/Image). **B, D**: Representative data of the time course of the cytotoxicity induction by compounds **6a** and **6b** in concentration 25 µM. Error bars represent mean ± SD of at least two biological replicates (N=2) made in triplicates. Cytotoxicity was quantified as the total green object integrated intensity (GCU × µm²/image) and normalized to the highest value measured at 48 hours. Statistical significance was determined using one-way ANOVA with Dunnett's multiple comparisons test and Person's *t*-test, comparing each treatment to control at the respective time point (****p < 0.0001; **p < 0.01; *p < 0.05 and ns, p > 0.05).

Organoids

Murine pancreatic intraepithelial neoplasia (mP2) and normal pancreatic duct (mN11) organoid models were employed. Mouse organoids were cultured in Mouse complete feeding medium, which is made of 90 % Mouse splitting medium (Advanced DMEM/F-12 (Gibco, Thermo Fischer Scientific, Waltham, MA, USA), 10 mM HEPES (Gibco, Thermo Fischer Scientific, Waltham, MA, USA), 100 U/mL penicillin and 100 U/mL (Sigma-Aldrich, St. Louis, MO, USA) and 1 % GlutaMAX (Gibco, Thermo Fischer Scientific, Waltham, MA, USA), 0.5 µM A 83-01, 0.05 µg/mL mEGF, 0.1 µg/mL hFGF-10, 0.01 µM hGastrin I, 0.1 µg/mL mNoggin, 1.25 mM *N*-acetylcysteine, 10mM Nicotinamide, R-Spondin 1-Conditioned medium and B27 supplement. Organoids were prepared by collecting and fragmenting them according to the Tuveson lab`s protocol. The fragmented organoids were resuspended in cold Matrigel (Corning #356231) at an optimized density and plated in a 96-well plate. The formation of organoids was constantly monitored using the pre-installed “Organoid” program in Incucyte S3. After approximately 3–4 days, well-formed organoids with diameters ranging from 100 to 400 µm were ready for treatment. During the treatment phase, the compounds were applied to organoids in three different concentrations, including two controls, untreated one and one with 0,5 % of DMSO. All treatments were performed in four technical and three biological replications to ensure reliable and reproducible results.

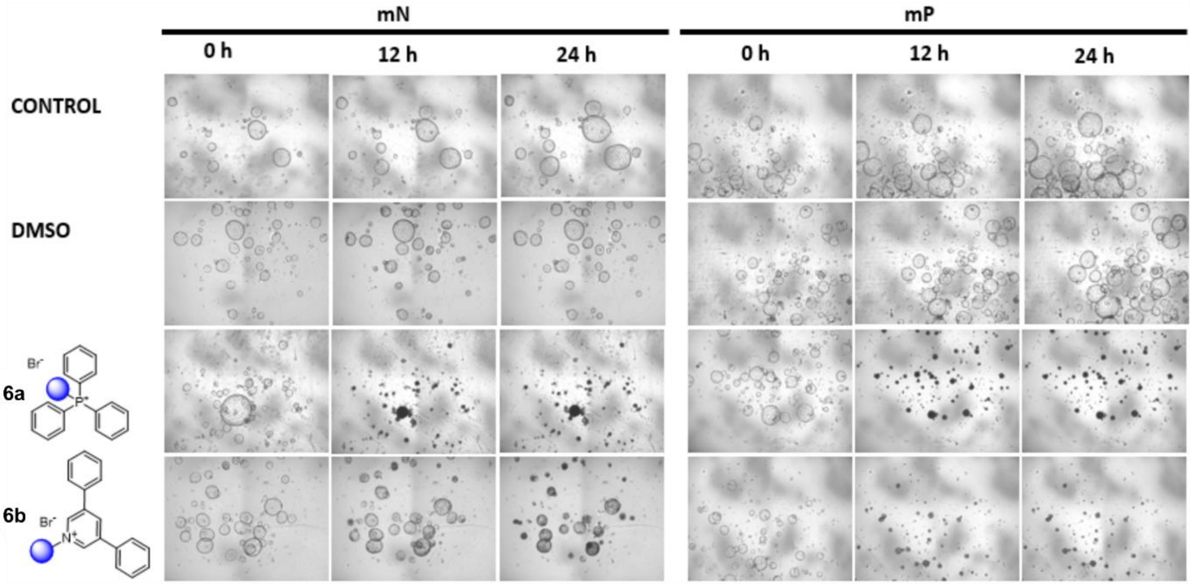

Figure S 42. Induction of PDAC organoid death by the compounds 6a and 6b. Representative pictures of the two types of organoids, untreated control, 0.1 % DMSO control and treated with 5 µM of compound **6a** and **6b** at 0 h, 12 h, and 24 h after treatment. For mN and mP: N = 3, n =4.

Stability studies of Compounds 6a and 6b (Tables S11–S16)

Analysis of Stability in Mouse and Human Plasma

Stability studies were performed by Bienta Enamine Biology Services, 78 Winston Churchill Street, 02094 Kyiv, Ukraine.

Reagents and consumables

DMSO Chromasolv Plus, HPLC grade, ≥99.7% (Sigma-Aldrich, USA; Cat #34869);
Acetonitrile Chromasolv, gradient grade, for HPLC, ≥99.9% (Sigma-Aldrich, USA; Cat #34851);
Formic acid for mass spectrometry, ~98% (Fluka) (Sigma-Aldrich, USA; Cat #94318);
DMSO stock solutions of the test compound at 20 mM;
Propantheline bromide ≥97% (TLC), powder (Sigma-Aldrich, USA; P8891);
Imipramine hydrochloride (Sigma-Aldrich, I7379);
Verapamil hydrochloride (Sigma Aldrich, USA; Cat #V4629);
Human plasma pooled, trisodium citrate as anticoagulant, from Kyiv city blood center;
Non-sterile mouse plasma with Li-heparin (Bienta/Enamine Ltd., batch #M031225);
Disposable pipette tips (Thermo Scientific, USA);
Agilent InfinityLab Poroshell 120 EC-C18, 2.1 x 50 mm, 4 µm (Cat #699770-902);;
0.75 ml microtubes in multiracks (ThermoScientific, USA; Cat # 4170)

Equipment

Gradient HPLC system Prominence UFLC XR (Shimadzu, Japan);
Triple quadrupole mass-detector API 3000 with TurboIonSpray Ion Source (AB Sciex, Canada);
IMT PN Ontouch nitrogen generator IMT-PN 1280 OG, nitrogen purity 99.99% (Inmatec, Germany);
Series II Water Jacketed CO2 incubator (Thermo Forma, USA);
Thermoshaker BioShake iQ (QInstruments, Germany);
Centrifuge 4-15C (Qiagen) (Sigma, Germany);
Water purification system Millipore Milli-Q Gradient A10 (Millipore, France);
Multichannel Electronic Pipettes 2-125 µL, 5-250 µL, 15-1250 µL, Matrix (Thermo Scientific, USA; Cat ## 2001, 2002, 2004)

Analytical System

All measurements were performed using Shimadzu HPLC system including vacuum degasser, gradient pumps, reverse phase HPLC column, column oven, and autosampler. Mass spectrometric analysis was performed using a Triple quadrupole mass-detector API 3000 with TurboIonSpray Ion Source (AB Sciex, Canada). The data acquisition and system control were performed using Analyst 1.6.3 software from (AB Sciex).

Methods

Incubations were carried out in 5 aliquots of 60 µL each (one for each time point), in duplicates. Test compounds (1 µM, final DMSO concentration 0.005%) were incubated with shaking (250 rpm) at 37 °C, 5% CO_2 and a saturating humidity (∼95%).[17] Five time points over 120 minutes have been analyzed. The reactions were stopped by adding 240 µL 90% acetonitrile containing internal standard with subsequent plasma proteins sedimentation by centrifuging at 6000 rpm for 5 minutes. Supernatants were analyzed by the HPLC system coupled with a tandem mass spectrometer. The percentage of the test compounds remaining after incubation in plasma and their half-lives ($T_{1/2}$) were calculated.

Table S 11. Mouse plasma stability of compounds **6a**, **6b**, and controls (verapamil and propantheline).

Compound ID	Time, min	Area Ratio		Mean Area Ratio	% Remain. Mean	$T_{1/2}$, min	Plot
		Inc. 1	Inc. 2				
Verapamil mouse	0	3.94E-01	3.99E-01	3.97E-01	100	1639*	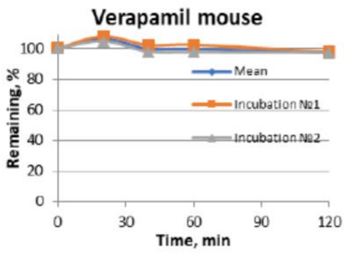
	20	4.26E-01	4.19E-01	4.22E-01	106		
	40	4.03E-01	3.91E-01	3.97E-01	100		
	60	4.04E-01	3.90E-01	3.97E-01	100		
	120	3.85E-01	3.88E-01	3.87E-01	97		
Propantheline mouse	0	2.92E+00	3.08E+00	3.00E+00	100	15	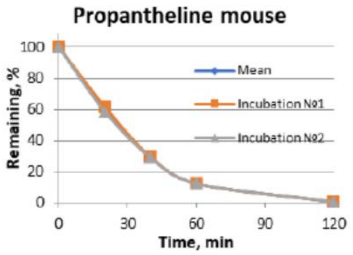
	20	1.81E+00	1.81E+00	1.81E+00	60		
	40	8.63E-01	8.94E-01	8.78E-01	29		
	60	3.59E-01	3.80E-01	3.69E-01	12		
	120	1.28E-02	1.68E-02	1.48E-02	0		
**6a** mouse	0	1.20E+00	1.21E+00	1.20E+00	100	877*	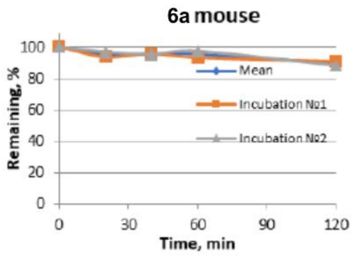
	20	1.13E+00	1.17E+00	1.15E+00	95		
	40	1.15E+00	1.15E+00	1.15E+00	96		
	60	1.12E+00	1.17E+00	1.15E+00	95		
	120	1.09E+00	1.06E+00	1.08E+00	90		
**6b**	0	7.61E-01	7.56E-01	7.58E-01	100	1125*	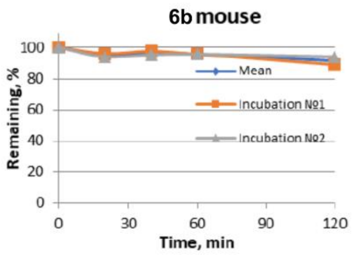
	20	7.31E-01	7.11E-01	7.21E-01	95		
	40	7.42E-01	7.21E-01	7.32E-01	96		
	60	7.28E-01	7.23E-01	7.26E-01	96		
	120	6.77E-01	7.11E-01	6.94E-01	91		

*Parameter should be considered as approximate due to high stability of this compound.

Table S 12. Human plasma stability of compounds **6a**, **6b**, and controls (verapamil and propantheline).

Compound ID	Time, min	Area Ratio		Mean Area Ratio	% Remain. Mean	$T_{1/2}$, min	Plot
		Inc. 1	Inc. 2				
Verapamil human	0	5.06E-01	4.84E-01	4.95E-01	100	1053*	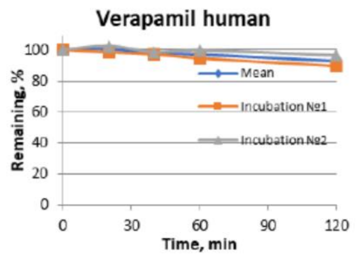
	20	4.97E-01	4.95E-01	4.96E-01	100		
	40	4.91E-01	4.77E-01	4.84E-01	98		
	60	4.78E-01	4.82E-01	4.80E-01	97		
	120	4.53E-01	4.66E-01	4.59E-01	93		
Propantheline human	0	2.33E+00	2.24E+00	2.28E+00	100	30	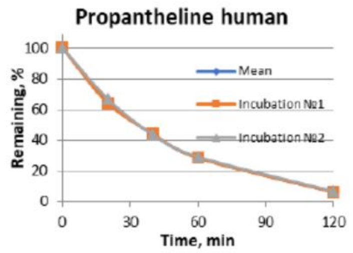
	20	1.49E+00	1.50E+00	1.49E+00	65		
	40	1.03E+00	9.72E-01	1.00E+00	44		
	60	6.58E-01	6.49E-01	6.54E-01	29		
	120	1.38E-01	1.47E-01	1.42E-01	6		
6a human	0	7.70E-01	8.43E-01	8.06E-01	100	778*	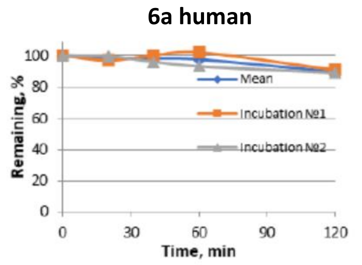
	20	7.46E-01	8.42E-01	7.94E-01	98		
	40	7.71E-01	8.11E-01	7.91E-01	98		
	60	7.87E-01	7.85E-01	7.86E-01	97		
	120	6.98E-01	7.48E-01	7.23E-01	90		
6b	0	9.05E-01	8.40E-01	8.73E-01	100	844*	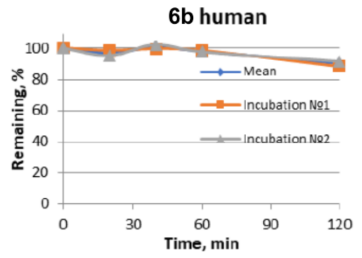
	20	8.91E-01	8.02E-01	8.47E-01	97		
	40	9.01E-01	8.59E-01	8.80E-01	101		
	60	8.91E-01	8.21E-01	8.56E-01	98		
	120	8.01E-01	7.71E-01	7.86E-01	90		

*Parameter should be considered as approximate due to high stability of this compound.

Assessment of Metabolic Stability in Human and Mouse Liver Microsomes for Compounds 6a and 6b

Reagents and consumables

DMSO Chromasolv Plus, HPLC grade, ≥99.7% (Sigma-Aldrich, USA; Cat# 34869)
Acetonitrile Chromasolv, gradient grade, for HPLC, ≥99.9% (Sigma-Aldrich, USA; Cat# 34851)
Methanol, HiPerSolv, HPLC-gradient grade, ≥99.9% (VWR Chemicals, USA, Cat# 20864.320)
Potassium phosphate monobasic (Bio-Basic, Canada; Lot #N9016010)
Potassium phosphate dibasic (Bio-Basic, Canada; Lot #MA7100050)
Magnesium chloride hexahydrate (Santa Cruz Biotechnology, Inc., USA; sc-203126A)
Mouse Liver Microsomes: pooled, male Balb/c mice (XenoTech, M3000/lot #1810163)
Human Liver Microsomes: pooled, mixed gender (XenoTech, H0630/lot N#2110263)
Glucose-6-phosphate dehydrogenase from baker's yeast, type XV (Sigma-Aldrich, USA; Cat #G6378)
D-Glucose-6-phosphate monosodium salt (EMD Milipore Corp., USA; Cat #346764-5GM)
NADPH tetrasodium salt (BLD Pharmatech Ltd., Cat #BD116582)
Formic acid (Sigma-Aldrich, 94318)
Verapamil hydrochloride (Sigma Aldrich, USA; Cat #V4629)
Niclosamide (Sigma-Aldrich, USA; Cat #N3510)
DMSO stock solutions of the tested compounds 20mM
(±)-Propranolol hydrochloride (Sigma-Aldrich, P0884)
Imipramine hydrochloride (Sigma-Aldrich, I7379)
Diclofenac sodium salt (Sigma-Aldrich, D6899)
InfinityLab Poroshell 120 EC-C18, 2.1 x 50 mm, 4 µm (Cat#699770-902)
Matrix™ 0.75 ml blank tubes (Cat #4170), pipettor tips (Thermo Scientific).

Equipment

Gradient HPLC system (Agilent Technologies)
Triple quadrupole mass-detector API 4000 with Turbo V Ion Source (AB Sciex, Canada)
Nitrogen generator N2-04-L1466, nitrogen purity 99%+ (Whatman)
Environmental Incubator Shaker G24; Digital Refrigerated Incubator/Shaker Innova 4330 (New Brunswick Scientific)
Water purification system Millipore Milli-Q Gradient A10 (Millipore, France)
Multichannel pipettors 1–30 µL, 2-125 µL, 30-850 µL (Thermo Scientific)

Analytical System

All measurements were performed using Agilent HPLC system including vacuum degasser, gradient pumps, reverse phase HPLC column, column oven, and autosampler. Mass spectrometric analysis was performed using an API 4000 mass spectrometer from AB Sciex with Turbo V ion source. The data acquisition and system control was performed using Analyst 1.6.3 software from AB Sciex.

Methods

Microsomal incubations were carried out in 96-well plates in 5 aliquots of 30 µL each (one for each time point). Liver microsomal incubation medium comprised of phosphate buffer (100 mM, pH 7.4), $MgCl_2$ (3.3 mM), NADPH (3 mM), glucose-6-phosphate (5.3 mM), glucose-6-phosphate dehydrogenase (0.67 units/mL) with 0.42 mg of liver microsomal protein per ml. In the control reactions, the NADPH-cofactor system was substituted with phosphate buffer. Test compounds (2 µM, final acetonitrile concentration 1.6 %) were incubated with microsomes at 37 °C, shaking at 100 rpm. Five time points over 40 minutes were analyzed. The reactions were stopped by adding 5 volumes of acetonitrile with internal standard to incubation aliquots, followed by protein sedimentation by centrifuging at 5500 rpm for 5 minutes. Each reaction was performed in duplicates. Supernatants were analyzed using the HPLC system coupled with a tandem mass spectrometer.

The elimination constant (k_{el}), half-life ($t_{1/2}$), and intrinsic clearance (C_{lint}) were determined in a plot of ln(AUC) versus time, using linear regression analysis. In order to indicate the quality of the linear regression analysis, the R^2 (determination coefficient) values are provided. In some cases, the last time point is excluded from the calculations to ensure acceptable logarithmic linearity of decay).

$k_{el} = -\text{slope}$
$t_{1/2} = 0.693/k_{el}$
$Cl_{int} = (0.693/t_{1/2})*(\mu L_{incubation}/mg_{microsomes})$

Table S 13. Human microsomal stability of componds **6a**, and controls (diclofenac and propranolol)

Compound ID	Time, min	Peak Area Ratio		Peak Area Ratio, Mean of 2	% Remaining, Mean of 2	R^2	k_{el}, min^{-1}	$t_{1/2}$, min	Cl_{int}, µl/min/mg	% Remaining without cofactor, Mean of 2
		Inc. 1	Inc. 2							
Diclofenac human	0	6.29E+00	5.32E+00	5.80E+00	100	0.997	0.084	8.3	202	100
	7	4.06E+00	3.06E+00	3.56E+00	61	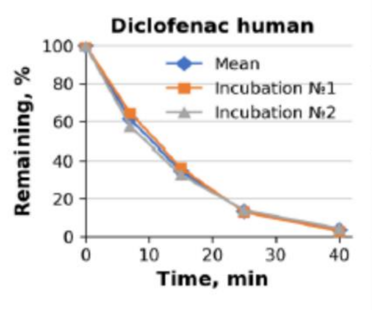				
	15	2.29E+00	1.74E+00	2.02E+00	35					
	25	8.21E-01	7.52E-01	7.86E-01	14					
	40	1.86E-01	2.31E-01	2.09E-01	4					93
Propranolol human	0	6.20E-01	5.61E-01	5.91E-01	100	0.973	0.007	100.5	17	100
	7	5.73E-01	5.18E-01	5.45E-01	92	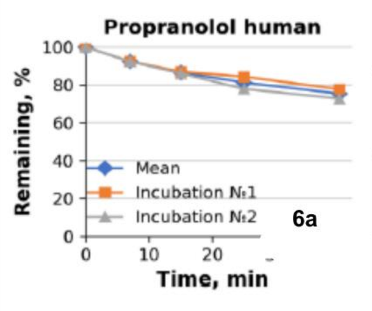				
	15	5.39E-01	4.83E-01	5.11E-01	87					
	25	5.23E-01	4.37E-01	4.80E-01	81					
	40	4.82E-01	4.08E-01	4.45E-01	75					88
**6a** human	0	1.58E+00	1.39E+00	1.48E+00	100	0.789	0.009	78.8	21	100
	7	1.27E+00	1.10E+00	1.19E+00	80	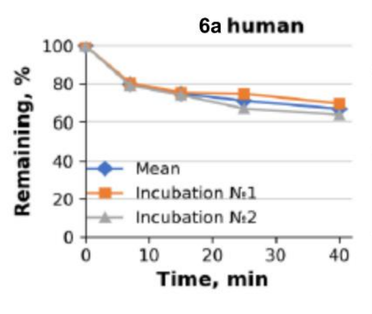				
	15	1.19E+00	1.03E+00	1.11E+00	75					
	25	1.18E+00	9.30E-01	1.05E+00	71					
	40	1.10E+00	8.84E-01	9.92E-01	67					92

Table S 14. Human microsomal stability of compond **6b**, and controls (diclofenac and propranolol)

Compound ID	Time, min	Analyte Peak Area Inc. 1	Analyte Peak Area Inc. 2	Analyte Peak Area, Mean of 2	% Remaining, Mean of 2	R^2	k_{el}, min^{-1}	$t_{1/2}$, min	Cl_{int}, μl/min/mg	% Remaining without cofactor, Mean of 2
Diclofenac human	0	5.86E-01	5.53E-01	5.70E-01	100	0.993	0.085	8.2	205	100
	7	4.45E-01	3.97E-01	4.21E-01	74	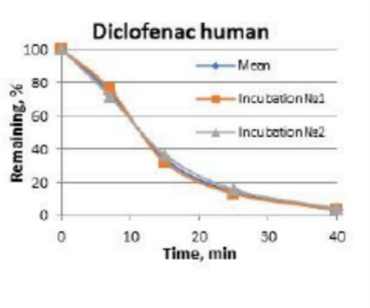				
	15	1.88E-01	2.02E-01	1.95E-01	34					
	25	7.61E-02	8.65E-02	8.13E-02	14					
	40	1.88E-02	2.32E-02	2.10E-02	4					103
Propranolol human	0	1.03E+00	9.75E-01	1.00E+00	100	0.940	0.010	71.6	23	100
	7	1.01E+00	9.61E-01	9.86E-01	98	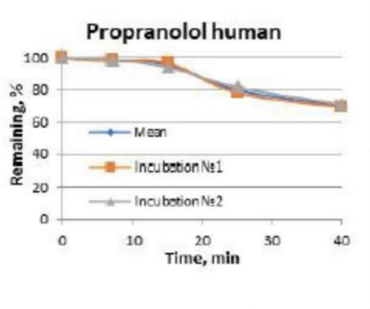				
	15	9.98E-01	9.16E-01	9.57E-01	95					
	25	8.05E-01	7.95E-01	8.00E-01	80					
	40	7.13E-01	6.87E-01	7.00E-01	70					95
**6b** human	0	9.70E-01	1.24E+00	1.10E+00	100	0.970	0.007	100.4	17	100
	7	9.07E-01	1.15E+00	1.03E+00	93	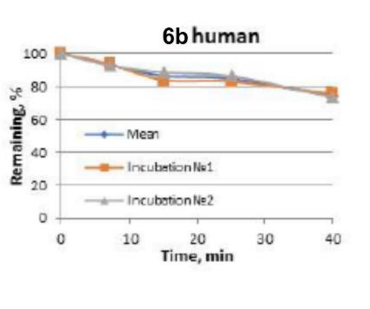				
	15	8.12E-01	1.10E+00	9.56E-01	87					
	25	8.02E-01	1.07E+00	9.36E-01	85					
	40	7.32E-01	9.16E-01	8.24E-01	75					107

Table S 15. Mouse microsomal stability of compond **6a**, and controls (imipramine and propranolol)

Compound ID	Time, min	Peak Area Ratio		Peak Area Ratio, Mean of 2	% Remaining, Mean of 2	R^2	k_{el}, min^{-1}	$t_{1/2}$, min	Cl_{int}, µl/min/mg	% Remaining without cofactor, Mean of 2
		Inc. 1	Inc. 2							
Imipramine mouse	0	1.65E+00	1.67E+00	1.66E+00	100	0.992	0.074	9.4	178	100
	7	1.15E+00	1.13E+00	1.14E+00	69	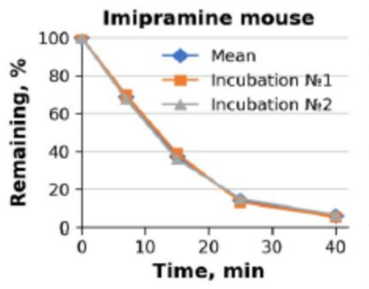				
	15	6.42E-01	6.01E-01	6.21E-01	37					
	25	2.20E-01	2.53E-01	2.36E-01	14					
	40	8.83E-02	1.07E-01	9.77E-02	6					102
Propranolol mouse	0	4.59E-01	4.65E-01	4.62E-01	100	0.831	0.025	27.8	60	100
	7	2.80E-01	3.15E-01	2.97E-01	64	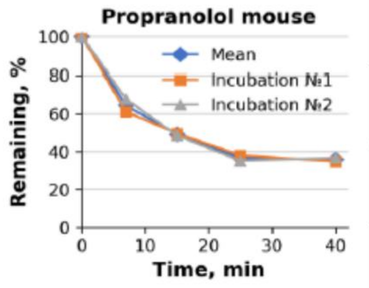				
	15	2.28E-01	2.26E-01	2.27E-01	49					
	25	1.75E-01	1.64E-01	1.69E-01	37					
	40	1.60E-01	1.71E-01	1.66E-01	36					100
**6a** mouse	0	1.85E+00	1.59E+00	1.72E+00	100	0.975	0.097	7.2	233	100
	7	1.07E+00	9.94E-01	1.03E+00	60	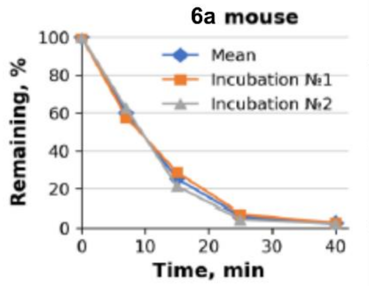				
	15	5.32E-01	3.40E-01	4.36E-01	25					
	25	1.34E-01	7.29E-02	1.03E-01	6					
	40	5.32E-02	3.67E-02	4.50E-02	3					96

Table S 16. Mouse microsomal stability of compond **6b**, and controls (imipramine and propranolol)

Compound ID	Time, min	Analyte Peak Area		Analyte Peak Area, Mean of 2	% Remaining, Mean of 2	R^2	k_{el}, min^{-1}	$t_{1/2}$, min	Cl_{int}, µl/min/mg	% Remaining without cofactor, Mean of 2
		Inc. 1	Inc. 2							
Propranolol mouse	0	1.02E+00	9.08E-01	9.64E-01	100	0.931	0.029	23.9	70	100
	7	6.85E-01	5.89E-01	6.37E-01	66	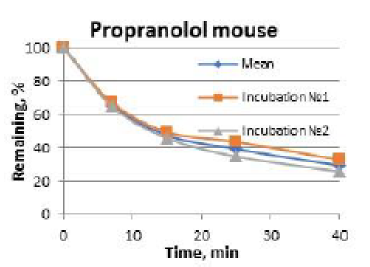				
	15	5.04E-01	4.14E-01	4.59E-01	48					
	25	4.45E-01	3.15E-01	3.80E-01	39					
	40	3.36E-01	2.32E-01	2.84E-01	29					100
Imipramine mouse	0	8.15E+00	8.19E+00	8.17E+00	100	0.995	0.071	9.8	170	100
	7	5.81E+00	5.81E+00	5.81E+00	71	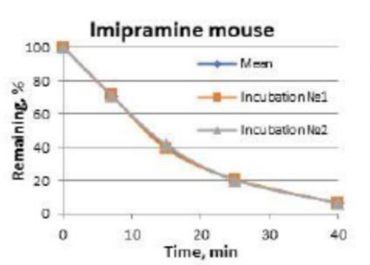				
	15	3.18E+00	3.38E+00	3.28E+00	40					
	25	1.67E+00	1.64E+00	1.65E+00	20					
	40	4.98E-01	4.98E-01	4.98E-01	6					97
**6b** mouse	0	9.31E-01	9.42E-01	9.36E-01	100	0.820	0.005	131.8	13	100
	7	8.16E-01	8.31E-01	8.23E-01	88	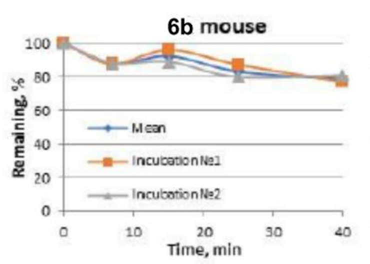				
	15	8.94E-01	8.38E-01	8.66E-01	92					
	25	8.12E-01	7.52E-01	7.82E-01	84					
	40	7.18E-01	7.61E-01	7.40E-01	79					100

Synthesis and characterization

Chemistry, General

Chemicals were obtained from BLDpharm (Reinbek, Germany), Enamine Ltd. (Kyiv, Ukraine), Sigma-Aldrich (St. Louis, MO), TCI (Tokyo, Japan), and Fluorochem Ltd. (Derbyshire, U.K.), and were used without further purification. For microwave-assisted reactions, we used Anton Paar Monowave 200 Microwave synthesis reactor. Analytical TLC was performed on silica gel Merck 60 F254 plates (0.25 mm), using visualization with UV light. Column chromatography was carried out on silica gel 60 (particle size 240–400 mesh). The automated normal-phase flash chromatography was performed on Biotage Selekt Enkel, using the Biotage Sfär Silica D 10 g column at flow rate 40 mL/min or Biotage Sfär Silica HC 5 g column at flow rate 18 mL/min, using en eluent mixture consisting of DCM/MeOH or hexane/EtOAc. Analytical reversed-phase UPLC analyses were performed on a modular system (Thermo Scientific Dionex UltiMate 3000 modular system; Thermo Fisher Scientific Inc., USA). A Waters Acquity UPLC® HSS C18 SB column (2.1 × 50 mm, 1.8 µm), t = 40 °C with a sample injection volume of 1-5 µL and a flow rate of 0.4 mL/min (method 1), and 0.3 mL/min (method 2), detector λ = 254 nm. The eluent consisted of 0.1% trifluoroacetic acid in ultrapure water (solvent A) and acetonitrile (solvent B). The gradient (defined for solvent B) in method 1 was 0–7.0 min, 5–95%; 7.0–8.0 min, 95%, and in method 2 was 0–10.0 min, 5–95%; 10.0–14.0 min, 95%; 14.0–14.5 min, 95–5%. ^{1}H NMR (400 MHz, internal Me_4Si), ^{13}C NMR (101 MHz, internal $CDCl_3$ DMSO-d_6 or acetone-d_6, δ = 77.16 ppm for $CDCl_3$, δ = 39.52 ppm for DMSO-d_6, δ = 29.84 ppm for acetone-d_6), ^{31}P NMR (162 MHz) and ^{19}F NMR (376 MHz) spectra were recorded on a Bruker AVANCE III 400 spectrometer (Bruker Corporation, Billerica, MA) in acetone-d_6, DMSO-d_6 or $CDCl_3$ solution. Chemical shifts δ are given in ppm. High-resolution mass spectra were obtained using an Exactive Plus Orbitrap mass spectrometer (Thermo Fisher Scientific, Waltham, MA). All final compounds were >95% pure by HPLC analysis unless otherwise specified.

S1. 3,5-Diphenylpyridine

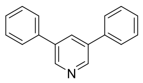

To a 30 mL microwave vial were added 3,5-diphenylpyridine (650 mg, 2.74 mmol, 1 eq), phenylboronic acid (834 mg, 6.84 mmol, 2.5 eq), tetrakis(triphenylphosphine)palladium (63 mg, 0.055 mmol, 0.02 eq), potassium carbonate (1.7 g, 12.35 mmol, 3.5 eq), flushed with argon, suspended in degassed dioxane/water 3:1 (20 mL), and stirred in a microwave reactor at 140 °C for 1 hour. The resulting mixture was filtered through Celite, washed with ethyl acetate (100 mL), and purified by automated silicagel chromatography (ethyl acetate:hexane 20–33 %), to get the title compound as white flakey crystals (602 mg, 95 % yield). **^{1}H NMR** (400 MHz, $CDCl_3$) δ 8.83 (d, J = 2.2 Hz, 2H), 8.05 (t, J = 2.2 Hz, 1H), 7.65 (dt, J = 8.2, 1.8 Hz, 4H), 7.60 – 7.48 (m, 4H), 7.48 – 7.35 (m, 2H). *Contains residual ethyl acetate (0.3 %). NMR data is in accordance with the literature data.[13]

S2. **5-Phenylthiazole**

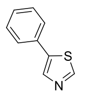

To a 30 mL microwave vial were added 5-bromothiazole (326 mg, 2 mmol, 1 eq), phenylboronic acid (243 mg, 2 mmol, 1 eq), tetrakis(triphenylphosphine)palladium (23 mg, 0.02 mmol, 0.01 eq), potassium carbonate (484 mg, 3.5 mmol, 1.75 eq), flushed with argon, suspended in degassed dioxane/water 3:1 (10 mL), and stirred in a microwave reactor at 140 °C for 15 minutes. The resulting mixture was concentrated, and dry-loaded onto a silicagel column (mobile phase ethyl acetate:hexane 9–33 %), to get the title compound as an off-white solid (258 mg, 80 % yield). **^{1}H NMR** (400 MHz, $CDCl_3$) δ 8.76 (s, 1H), 8.09 (s, 1H), 7.63 – 7.55 (m, 2H), 7.47 – 7.39 (m, 2H), 7.36 (dt, J = 9.6, 4.3 Hz, 1H). NMR data is in accordance with the literature data.[14]

S3. Methyl 2-(6-((6-chlorohexyl)oxy)-3-oxo-3*H*-xanthen-9-yl)benzoate

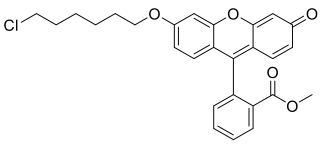

To a solution of fluorescein methyl ester (1 g, 2.9 mmol), prepared according to a literature-described procedure,[15] in DMF (40 mL) was added 1-bromo-6-chlorohexane (0.86 g, 640 µL, 4.34 mmol, 1.5 eq) and cesium carbonate (1.42 g, 4.34 mmol, 1.5 eq). The reaction mixture was stirred at 50 °C overnight. The reaction mixture was then concentrated in vacuo, 80 mL DCM and 100 mL H_2O were added, and the aqueous layer was washed additionally with DCM (2 × 80 mL). The organic phases were joined, washed with brine (100 mL), dried with Na_2SO_4(s), and filtered. The filtrate was concentrated in vacuo, and purified by automated flash chromatography on silica, eluent 0–7% MeOH in dichloromethane, to get the chloride intermediate as a dark orange oil, used without further purification (73 % yield, 0.989 g). **^{1}H NMR** (400 MHz, $CDCl_3$) δ 8.24 (dd, J = 7.8, 1.0 Hz, 1H), 7.74 (td, J = 7.5, 1.3 Hz, 1H), 7.67 (td, J = 7.7, 1.3 Hz, 1H), 7.31 (dd, J = 7.5, 0.9 Hz, 1H), 6.94 (d, J = 2.4 Hz, 1H), 6.86 (dd, J = 12.3, 9.3 Hz, 2H), 6.73 (dd, J = 8.9, 2.4 Hz, 1H), 6.54 (dd, J = 9.7, 1.9 Hz, 1H), 6.46 (d, J = 1.9 Hz, 1H), 4.07 (t, J = 6.4 Hz, 2H),

3.64 (s, 3H), 3.56 (t, J = 6.6 Hz, 2H), 1.84 (dt, J = 13.5, 6.7 Hz, 4H), 1.60 – 1.46 (m, 4H). *Contains residual DCM (7.9 %) and DMF (5.3 %).

S4. Methyl 2-(6-((6-iodohexyl)oxy)-3-oxo-3*H*-xanthen-9-yl)benzoate

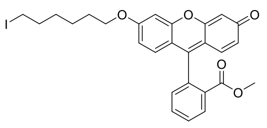

To a solution of the above alkyl chloride **S3** (0.989 g, 2.12 mmol) in acetone (16 mL) was added sodium iodide (2.5 g, 16.9 mmol, 8 eq), and the reaction mixture was stirred at 100 °C for 30 min in a microwave reactor. To the reaction mixture was added DCM (100 mL) to precipitate the inorganic salts, and the clear solution was decanted to a round-bottom flask, followed by additional washing of the inorganic precipitate with DCM (2 × 20 mL). The resulting solution was concentrated in vacuo, and purified by automatic flash chromatography on silica, eluent 0–7% MeOH in dichloromethane, to get the iodide **S4** as an orange-red solid (59 % yield, 0.690 g). **^{1}H NMR** (400 MHz, $CDCl_3$) δ 8.27 – 8.21 (m, 1H), 7.74 (td, J = 7.5, 1.4 Hz, 1H), 7.67 (dt, J = 7.6, 3.8 Hz, 1H), 7.31 (d, J = 7.5 Hz, 1H), 6.94 (d, J = 2.4 Hz, 1H), 6.86 (dd, J = 12.1, 9.3 Hz, 2H), 6.72 (dd, J = 8.9, 2.4 Hz, 1H), 6.54 (dd, J = 9.7, 1.9 Hz, 1H), 6.45 (d, J = 1.9 Hz, 1H), 4.07 (t, J = 6.4 Hz, 2H), 3.64 (s, 3H), 3.26 – 3.18 (m, 2H), 1.91 – 1.82 (m, 4H), 1.57 – 1.44 (m, 4H). *Contains residual DCM (1.3 %) and DMF (3.2 %).

1a. (6-((9-(2-(Methoxycarbonyl)phenyl)-3-oxo-3*H*-xanthen-6-yl)oxy)hexyl)triphenylphosphonium iodide

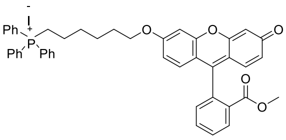

S4 (30 mg 0.054 mmol, 1 eq) of and triphenylphosphine (100 mg, 0.382 mmol, 7 eq) were added into a 10 mL microwave vial, flushed with argon, and dissolved in acetonitrile (2 mL). The reaction mixture was stirred in a microwave reactor at 135 °C for 1 h. The resulting mixture was concentrated to 0.5–1 mL, and precipitated with 25 mL diethyl ether, filtered and redissolved in dichloromethane, to be purified by automatic flash chromatography, upon which it was redissolved in DCM due to insufficient purity and the precipitation was repeated, to get the title compound as an orange powder (30.5 mg, 69% yield). **^{1}H NMR** (400 MHz, $CDCl_3$) δ 8.24 (dd, *J* = 8.1, 1.3 Hz, 1H), 7.78 – 7.89 (m, 9H), 7.66 – 7.74 (m, 8H), 7.28 – 7.33 (m, 1H), 6.81 – 6.91 (m, 3H), 6.75 (dd, *J* = 8.9, 2.4 Hz, 1H), 6.53 (dd, *J* = 9.7, 1.9 Hz, 1H), 6.44 (d, *J* = 1.9 Hz, 1H), 4.06 (t, *J* = 6.4 Hz, 2H), 3.81 (d, *J* = 3.4 Hz, 2H), 3.63 (s, 3H), 1.81 (t, *J* = 7.5 Hz, 4H), 1.68 (d, *J* = 8.2 Hz, 2H), 1.52 (s, 2H). *Contains residual acetonitrile (2.0 %) and DMF (1.8 %). **HRMS** (ESI+): calcd. For $C_{45}H_{40}O_5P^+$ $[M]^+$ 691.26079, found 691.25921 (− 2.28 ppm). **HPLC** purity, 90.14 % at 254 nm (t_R = 4.5 min).

1b. 1-(6-((9-(2-(methoxycarbonyl)phenyl)-3-oxo-3*H*-xanthen-6-yl)oxy)hexyl)-3,5-diphenylpyridin-1-ium iodide

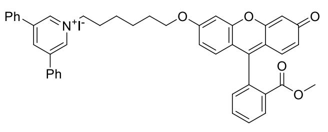

S4 (30 mg, 0.054 mmol, 1 eq) and **S1** (50 mg, 0.216 mmol, 4 eq) were added into a 10 mL microwave vial, flushed with argon, and dissolved in acetonitrile (2 mL). The reaction mixture was stirred in a microwave reactor at 135 °C for 1 h. The resulting mixture was concentrated to 0.5–1 mL, and precipitated with diethyl ether (25 mL), the precipitate collected and redissolved in DCM (0.5–1 mL), and precipitated with diethyl ether (25 mL), to get the title compound as a brown solid (35 mg, 82% yield). **^{1}H NMR** (400 MHz, $CDCl_3$) δ 9.58 (s, 2H), 8.57 (s, 1H), 8.24 (d, *J* = 7.7 Hz, 1H), 7.93 (d, *J* = 5.9 Hz, 4H), 7.74 (t, *J* = 7.2 Hz, 1H), 7.67 (t, *J* = 7.5 Hz, 1H), 7.52 (d, *J* = 7.0 Hz, 6H), 7.30 (s, 1H), 6.87 (d, *J* = 7.7 Hz, 2H), 6.84 (s, 1H), 6.70 (d, *J* = 8.7 Hz, 1H), 6.52 (d, *J* = 8.4 Hz, 1H), 6.42 (s, 1H), 5.32 (d, *J* = 16.2 Hz, 2H), 4.03 (s, 2H), 3.63 (d, *J* = 7.5 Hz, 3H), 2.15 (s, 2H), 1.94 (s, 2H), 1.80 (s, 2H), 1.57 (s, 2H – covered by H_2O protons). **^{13}C{^{1}H} NMR** (101 MHz, $CDCl_3$) δ 165.71, 163.94, 154.52, 141.61, 140.54, 139.77, 134.66, 132.88, 132.77, 131.24, 130.87, 130.67, 130.34, 129.97, 129.85, 129.06, 127.96, 117.45, 114.82, 114.16, 100.99, 77.48, 77.16, 76.84, 68.80, 65.96, 62.24, 52.55, 32.36, 28.60, 25.68, 25.53. **HRMS** (ESI+): calcd. For $C_{44}H_{38}NO_5^+$ $[M]^+$ 660.27445, found 660.27310 (− 2.04 ppm). **HPLC** purity, 90.14 % at 254 nm (t_R = 4.5 min).

S5. Methyl 2-(4-(mesylamino)phenyl)acetate[16]

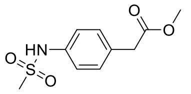

A solution of methyl 2-(4-aminophenyl)acetate (7.0 g, 42.4 mmol, 1.0 equiv) and triethylamine (8.81 mL, 63.6 mmol, 1.5 equiv) in dichloromethane (100 mL) at 0 °C was treated with mesyl chloride (3.61 mL, 46.6 mmol, 1.1 equiv) dropwise. The reaction mixture was stirred for 30 minutes in an ice bath, then it was transferred to a separatory funnel, and the organic phase was washed sequentially with 2 M HCl (2 × 30 mL), saturated aqueous $NaHCO_3$ solution (50 mL), and brine (30 mL). The organic phase was dried over anhydrous Na_2SO_4, filtered, and the volatiles were removed under reduced pressure. The crude contained 15 mol% of dimesylated product and was purified by flash chromatography on silica, eluent DCM/MeOH 20:1, to get the title compound as a white solid (7.53 g, 73% yield). **^{1}H NMR** (400 MHz, $CDCl_3$) δ 7.27 (d, *J* = 8.7 Hz, 2H), 7.18 (d, *J* = 8.5 Hz, 1H), 6.56 (s, 1H), 3.71 (s, 2H), 3.61 (s, 1H), 3.01 (s, 2H).

S6. 2-(4-(Mesylamino)phenyl)acetic acid

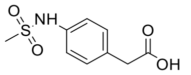

The above ester **S5** (2.65 g, 10.9 mmol, 1 equiv) was dissolved in methanol (30 mL), and 4 M NaOH (10.9 mL, 43.6 mmol, 4 equiv) was added with stirring. The reaction mixture was stirred at room temperature for 12 hours. The reaction mixture was concentrated under

reduced pressure (40 °C, 90 mbar) and the remaining aqueous solution was acidified with 2 M HCl to pH 2. The mixture was transferred to a separatory funnel and extracted with EtOAc (3 × 50 mL). The organic phase was washed with brine (50 mL), dried over anhydrous Na_2SO_4, filtered, and concentrated under reduced pressure. The crude product was triturated with DCM to get the title compound as a white solid (2.3 g, 92% yield). **^{1}H NMR** (400 MHz, DMSO-d_6) δ 12.32 (s, 1H), 9.68 (s, 1H), 7.21 (d, *J* = 8.5 Hz, 2H), 7.14 (d, *J* = 8.5 Hz, 2H), 3.52 (s, 2H), 2.96 (s, 3H). NMR data is in accordance with the literature data.[17]

S7. 5-Diethylamino-2-trifluoroacetylphenol

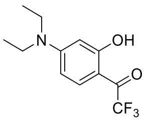

3-(Diethylamino)phenol (61.13 g, 367.0 mmol, 1.0 equiv) was dissolved in diethyl ether (100 mL) and cooled in an ice bath. Trifluoroacetic anhydride (154.4 mL, 1.11 mol, 3.0 equiv) was then added dropwise. The reaction mixture was stirred under reflux conditions for 24 hours. After cooling to room temperature, the reaction mixture was poured into a large beaker containing ice. $NaHCO_3$ was gradually added with stirring until the pH of the aqueous phase reached 7. The mixture was transferred to a separatory funnel, and the organic phase was washed with water (50 mL) and brine (50 mL). The organic layer was dried over anhydrous Na_2SO_4, filtered through a layer silica gel, eluent DCM, and the solvent was removed under reduced pressure, yielding the title compound as a brown viscous liquid (57 g, 59% yield). **^{1}H NMR** (400 MHz, $CDCl_3$) δ 11.83 (s, 1H), 7.57 (dq, *J* = 9.4, 2.2 Hz, 1H), 6.27 (dd, *J* = 9.5, 2.6 Hz, 1H), 6.11 (d, *J* = 2.6 Hz, 1H), 3.44 (q, *J* = 7.1 Hz, 4H), 1.23 (t, *J* = 7.2 Hz, 6H). **^{19}F NMR** (376 MHz, $CDCl_3$) δ −69.43 (d, *J* = 2.2 Hz) NMR data is in accordance with the literature data.[18]

2 (Spidye). *N*-(4-(7-(Diethylamino)-2-oxo-4-(trifluoromethyl)-2*H*-chromen-3-yl)phenyl)methanesulfonamide

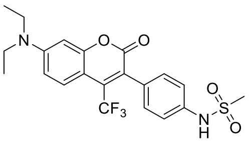

The above phenol **S7** (2.28 mg, 8.72 mmol, 1 equiv) and carboxylic acid **S6** (2.00 g, 8.72 mmol, 1 equiv) were dissolved in dichloromethane (DCM, 100 mL) along with triethylamine (6.1 mL, 44 mmol, 5 equiv) and *N*,*N*-dimethylaminopyridine (533 mg, 4.36 mmol, 0.5 equiv). While stirring, 1-ethyl-3-(3-dimethylaminopropyl)carbodiimide hydrochloride (3.34 g, 17.4 mmol, 2 eq) was added, and the reaction mixture was refluxed overnight. Reaction mixture was cooled to a room temperature and transferred to the separatory funnel. The organic layer was washed with 1 M HCl (150 mL), saturated $NaHCO_3$ solution (150 mL), brine (200 mL). The organic phase was dried over anhydrous Na_2SO_4, filtered, and the solvent was removed under reduced pressure. The crude product was purified by flash column chromatography (mobile phase DCM/MeOH 40:1) to afford the title compound (1.52 g, 38%) as a yellow solid. **^{1}H NMR** (400 MHz, $CDCl_3$) δ 7.60 (dq, *J* = 9.3, 2.4 Hz, 1H), 7.31 – 7.21 (m, 4H), 6.66 (dd, *J* = 9.4, 2.7 Hz, 1H), 6.61 (s, 1H),

6.56 (d, *J* = 2.7 Hz, 1H), 3.45 (q, *J* = 7.1 Hz, 4H), 3.06 (s, 3H), 1.24 (t, *J* = 7.1 Hz, 6H). *Contains residual DCM (2.9 %). **^{13}C NMR** (101 MHz, $CDCl_3$) δ 162.16, 156.24, 151.11, 137.84 (q, *J* = 29.9 Hz), 137.57, 131.37 (q, *J* = 1.8 Hz), 127.65 (q, *J* = 3.9 Hz), 125.74, 122.95 (q, *J* = 278.9 Hz), 121.76 (q, *J* = 2.6 Hz), 120.04, 109.99, 104.05, 97.96, 45.30, 39.87, 12.89. **^{19}F NMR** (376 MHz, $CDCl_3$) δ −54.92 (d, *J* = 2.1 Hz). **HRMS** (ESI): *m/z* calcd for $C_{21}H_{21}O_4N_2F_3NaS$ $[M + H]^+$ 477.1066; found 477.1061.

3. *N*-(6-Bromohexyl)-*N*-(4-(7-(diethylamino)-2-oxo-4-(trifluoromethyl)-2*H*-chromen-3-yl)phenyl)methanesulfonamide

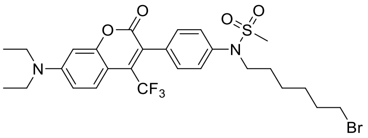

A suspension of the above mesylamine **2** (504 mg, 1.11 mmol, 1.0 equiv), K_2CO_3 (306 mg, 2.22 mmol, 2.0 equiv), and 1,6-dibromohexane (768 µL, 3.33 mmol, 3.0 equiv) in MeCN (10 mL) was stirred at 50 °C overnight, then concentrated under reduced pressure. The residue was partitioned between DCM (30 mL) and brine (30 mL). The organic layer was dried over Na_2SO_4, filtered and concentrated. The crude product thus obtained was purified by flash column chromatography (mobile phase DCM, then 1% MeOH in DCM), yielding the title compound as a yellow solid (630 mg, 92% yield). **^{1}H NMR** (400 MHz, $CDCl_3$) δ 7.60 (dq, *J* = 9.4, 2.2 Hz, 1H), 7.40 (d, *J* = 8.6 Hz, 2H), 7.32 (d, *J* = 8.6 Hz, 2H), 6.66 (dd, *J* = 9.3, 2.7 Hz, 1H), 6.57 (d, *J* = 2.7 Hz, 1H), 3.71 (t, *J* = 7.1 Hz, 2H), 3.45 (q, *J* = 7.1 Hz, 4H), 3.38 (t, *J* = 6.8 Hz, 2H), 2.89 (s, 3H), 1.83 (p, *J* = 6.9 Hz, 2H), 1.59 – 1.48 (m, 2H), 1.47 – 1.31 (m, 4H), 1.24 (t, *J* = 7.1 Hz, 6H). **^{13}C NMR** (101 MHz, $CDCl_3$) δ 161.35, 156.02, 150.86, 139.32, 137.62 (q, *J* = 29.7 Hz), 133.61, 130.91 (q, *J* = 1.9 Hz), 127.83, 127.37 (q, *J* = 3.8 Hz), 122.61 (q, *J* = 279.0 Hz), 121.28 (q, *J* = 2.3 Hz), 109.63, 103.64, 97.64, 50.56, 45.00, 37.21, 33.87, 32.70, 28.60, 27.72, 25.59, 12.58. **^{19}F NMR** (376 MHz, $CDCl_3$) δ −54.86 (d, *J* = 2.1 Hz). **HRMS** (ESI): m/z calcd for $C_{27}H_{33}O_4N_2F_3SBr$ $[M + H]^+$ 617.1291; found 617.1297.

4a. (6-(*N*-(4-(7-(Diethylamino)-2-oxo-4-(trifluoromethyl)-2*H*-chromen-3-yl)phenyl)methylsulfonamido)hexyl)triphenylphosphonium bromide

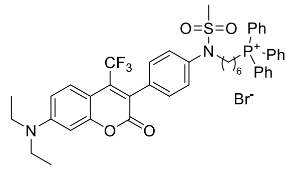

To a heat-dried microwave vial (10 mL loading) under argon atmosphere were added intermediate **3** (50 mg, 0.81 mmol), triphenylphosphine (171 mg, 0.71 mmol, 1.47 eq), acetonitrile (2 mL), and the reaction mixture was stirred in a microwave reactor at 120 °C for 1 hour. The reaction mixture was then concentrated in vacuo to approximately 0.5 - 1 mL. The mixture was precipitated with 50 mL diethyl ether, redisolved in 1 mL dichloromethane and precipitated using 50 mL diethyl ether, to get the title compound as a bright yellow solid (46 mg, 95 % yield). **^{1}H NMR** (400 MHz, $CDCl_3$) δ 7.88 – 7.66

(m, 15H), 7.64 – 7.56 (m, 1H), 7.40 (d, *J* = 8.4 Hz, 2H), 7.27 (d, *J* = 8.3 Hz, 2H), 6.66 (dd, *J* = 9.3, 2.7 Hz, 1H), 6.55 (d, *J* = 2.7 Hz, 1H), 3.80 – 3.64 (m, 4H), 3.46 (q, *J* = 7.1 Hz, 4H), 2.90 (s, 3H), 1.90 – 1.77 (m, 2H), 1.73 – 1.56 (m, 2H), 1.53 – 1.41 (m, 2H), 1.41 – 1.30 (m, 2H), 1.24 (t, *J* = 7.0 Hz, 6H). **$^{13}C\{^1H\}$ NMR** (101 MHz, $CDCl_3$) δ 161.36, 155.97, 150.83, 139.35, 135.08 (d, *J* = 3.0 Hz), 133.84 (d, *J* = 10 Hz), 133.42, 130.81, 130.61 (d, *J* = 13 Hz), 127.79, 127.38 (q, *J* = 3.7 Hz), 122.56 (q, *J* = 280 Hz), 121.28 (q, *J* = 4 Hz), 118.51 (d, *J* = 86 Hz), 109.64, 103.64, 97.58, 50.29, 44.99, 37.13, 29.47 (d, *J* = 16.0 Hz), 28.03, 25.69, 23.40 – 22.11 (m, 2C), 12.58. **^{19}F NMR** (376 MHz, $CDCl_3$) δ −54.83 (d, *J* = 2.5 Hz). **^{31}P NMR** (162 MHz, $CDCl_3$) δ 24.39. **HRMS** (ESI): *m/z* calcd for $C_{45}H_{47}O_4N_2F_3PS$ $[M]^+$ 799.2941; found 799.2923 (− 2.25 ppm). **HPLC** purity, 97.07 % at 254 nm (t_R = 5.8 min).

4b. 1-(6-(*N*-(4-(7-(Diethylamino)-2-oxo-4-(trifluoromethyl)-2*H*-chromen-3-yl)phenyl)methylsulfonamido)hexyl)-3,5-diphenylpyridin-1-ium bromide

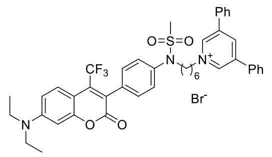

To a heat-dried microwave vial (10 mL loading) under argon atmosphere were added intermediate **3** (50 mg, 0.81 mmol), **S1** (70 mg, 0.71 mmol, 3.7 eq), acetonitrile (2 mL), and the reaction mixture was stirred in a microwave reactor at 140 °C for 1 hour. The reaction mixture was then concentrated in vacuo to approximately 0.5–1 mL. The mixture was precipitated with 50 mL diethyl ether, redisolved in 1 mL dichloromethane and precipitated using 50 mL diethyl ether, to get the title compound as a bright yellow solid (45 mg, 65 % yield). **^1H NMR** (400 MHz, $CDCl_3$) δ 9.60 (s, 2H), 8.60 (s, 1H), 7.94 (d, *J* = 7.2 Hz, 4H), 7.63 – 7.44 (m, 7H), 7.37 (d, *J* = 8.3 Hz, 2H), 7.22 (d, *J* = 8.3 Hz, 2H), 6.66 (dd, *J* = 9.3, 2.2 Hz, 1H), 6.53 (d, *J* = 2.2 Hz, 1H), 5.25 (t, *J* = 7.1 Hz, 2H), 3.69 (t, *J* = 6.5 Hz, 2H), 3.45 (q, *J* = 6.8 Hz, 4H), 2.84 (s, 3H), 2.03 (s, 2H), 1.48 (dd, *J* = 13.1, 6.7 Hz, 2H), 1.41 (s, 4H), 1.24 (t, *J* = 7.0 Hz, 6H). **$^{13}C\{^1H\}$ NMR** (101 MHz, $CDCl_3$) δ 12.57, 25.09, 25.44, 28.16, 32.12, 37.09, 44.98, 50.07, 62.21, 97.52, 103.59, 109.67, 121.15, 123.90, 127.29 – 127.49 (m), 127.85, 127.94, 129.94, 130.71, 130.76, 133.05, 133.47, 137.60 (q, *J* = 30.2 Hz), 139.38, 139.71, 140.89, 141.59, 150.85, 155.96, 161.42. **^{19}F NMR** (376 MHz, CDCl3) δ −54.88, −54.87. **HRMS** (ESI+): calcd. For $C_{44}H_{45}F_3N_3O_4S^+$ $[M]^+$ 768.30774, found 768.30652 (− 1.59 ppm). **HPLC** purity, 95.73 % at 254 nm (t_R = 5.8 min).

4c. 1-(6-(*N*-(4-(7-(Diethylamino)-2-oxo-4-(trifluoromethyl)-2*H*-chromen-3-yl)phenyl)methylsulfonamido)hexyl)pyridin-1-ium bromide

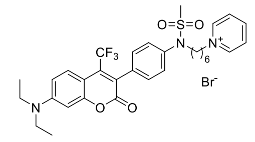

To a heat-dried microwave vial (10 mL loading) under argon atmosphere were added intermediate **3** (52 mg, 0.084 mmol), pyridine (202 mg, 2.55 mmol, 31 eq), acetonitrile (2 mL), and the reaction mixture was stirred in a microwave reactor at 100 °C for 1 hour. The reaction mixture was then concentrated in vacuo to approximately 0.5–1 mL. The mixture was precipitated with 35 mL diethyl ether, redisolved in 1 mL dichloromethane

and precipitated using 35 mL diethyl ether, followed by dissolution with dichloromethane, and precipitation with diethyl ether, to get the title compound as a yellow glassy oil (35 mg, 60 % yield). **^{1}H NMR** (400 MHz, $CDCl_3$) δ 1.24 (td, J = 7.2, 2.5 Hz, 6H), 1.39 (s, 2H), 1.48 (s, 2H), 1.87 (d, J = 21.4 Hz, 2H), 2.00 (s, 2H), 2.90 (d, J = 1.5 Hz, 3H), 3.45 (dt, J = 7.4, 3.7 Hz, 4H), 3.70 (q, J = 5.4, 4.4 Hz, 2H), 4.94 (d, J = 7.1 Hz, 2H), 6.55 (d, J = 3.0 Hz, 1H), 6.66 (dt, J = 9.4, 2.4 Hz, 1H), 7.31 (dd, J = 8.5, 2.4 Hz, 2H), 7.36 – 7.49 (m, 2H), 7.59 (d, J = 10.2 Hz, 1H), 8.10 (t, J = 6.8 Hz, 2H), 8.48 (d, J = 7.7 Hz, 1H), 9.47 (d, J = 5.8 Hz, 2H). **^{13}C{^{1}H} NMR** (101 MHz, $CDCl_3$) δ 12.54, 25.03, 25.32, 28.00, 31.69, 37.20, 44.96, 50.01, 61.76, 97.52, 103.53, 109.71, 118.37, 120.98 (d, J = 2.4 Hz), 122.53 (q, J = 279.1 Hz), 126.69, 127.21 – 127.94 (m), 128.54, 130.87, 133.66, 137.70 (q, J = 29.9 Hz), 139.18, 145.22, 145.24, 150.89, 155.97, 161.47. **^{19}F NMR** (376 MHz, $CDCl_3$) δ −54.81. **HRMS** calcd. For $C_{32}H_{37}O_5N_3F_3S^+$ $[M]^+$ 616.24514, found 616.24359 (− 2.51 ppm). **HPLC** purity, 97.77 % at 254 nm (t_R = 4.9 min).

4c-*d*$_5$. 1-(6-(*N*-(4-(7-(dDethylamino)-2-oxo-4-(trifluoromethyl)-2*H*-chromen-3-yl)phenyl)methylsulfonamido)hexyl)pyridin-1-ium-2,3,4,5,6-*d*$_5$

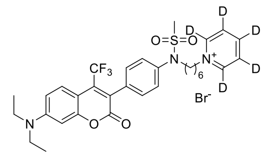

To a heat-dried microwave vial (10 mL loading) under argon atmosphere were added intermediate **3** (30 mg, 0.049 mmol), pyridine-*d*$_5$ (135 mg, 1.6 mmol, 33 eq), acetonitrile (2 mL), and the reaction mixture was stirred in a microwave reactor at 120 °C for 80 minutes. The reaction mixture was then concentrated in vacuo, and dissolved in 0.5 mL DCM. The mixture was precipitated with 35 mL diethyl ether, redisolved in 0.5 mL acetonitrile and precipitated using 35 mL diethyl ether, followed by dissolution with dichloromethane, and precipitation with diethyl ether. The residue was dissolved in 1 mL dichloromethane, and transferred to an automatic silicagel column (DCM:MeOH 0-22%), to get the title compound as a glassy yellow oil (22 mg, 64 % yield). **^{1}H NMR** (400 MHz, $CDCl_3$) δ 7.67 – 7.49 (m, 1H), 7.41 (d, *J* = 8.3 Hz, 2H), 7.37 – 7.19 (m, 2H), 6.66 (dd, *J* = 9.4, 2.4 Hz, 1H), 6.55 (d, *J* = 2.4 Hz, 1H), 4.94 (t, *J* = 7.3 Hz, 2H), 3.71 (t, *J* = 6.4 Hz, 2H), 3.46 (q, *J* = 6.9 Hz, 4H), 2.90 (s, 3H), 2.18 (residual acetone present in NMR tube), 2.02 (d, *J* = 6.2 Hz, 2H), 1.49 (s, 2H), 1.40 (s, 4H), 1.26 – 1.15 (m, 6H). **^{13}C NMR** (101 MHz, $CDCl_3$) δ 161.45, 155.96, 150.88, 144.81 (t), 139.19, 137.68 (q, *J* = 30.0 Hz), 133.65, 130.85, 128.74 – 127.91 (m), 127.86, 127.34 (d, *J* = 4.0 Hz), 122.52 (q, *J* = 279.0 Hz), 120.98 (d, *J* = 1.9 Hz), 109.71, 103.52, 97.51, 61.64, 50.03, 44.95, 37.23, 31.65, 29.77 (Residual acetone present in NMR tube), 28.00, 25.33, 25.03, 12.53. **^{19}F NMR** (376 MHz, $CDCl_3$) δ -54.81, -54.81. **HRMS** calcd. For $C_{32}H_{32}D_5F_3N_3O_4S^+$ $[M]^+$ 621,2765, found 621.2758 (− 1.12 ppm). **HPLC** purity, 98.19 % at 254 nm (t_R = 4.9 min).

4d. 3-(6-(*N*-(4-(7-(Diethylamino)-2-oxo-4-(trifluoromethyl)-2*H*-chromen-3-yl)phenyl)methylsulfonamido)hexyl)-5-phenylthiazol-3-ium bromide

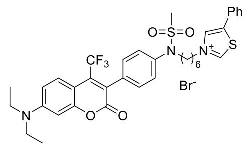

To a heat-dried microwave vial (10 mL loading) under argon atmosphere were added intermediate **3** (60 mg, 0.097 mmol), **S2** (106 mg, 0.66 mmol, 6.8 eq), acetonitrile (2 mL), and the reaction mixture was stirred in a microwave reactor at 160 °C for 1 hour. The reaction mixture was then concentrated in vacuo to 2 mL, followed by precipitation using 40 mL diethyl ether. The solid residue was disolved 1 mL dichloromethane and precipitated using 40 mL diethyl eher, to get the title compound as a yellow oil (43 mg, 57 % yield). **^{1}H NMR** (400 MHz, $CDCl_3$) δ 1.24 (td, J = 7.1, 1.7 Hz, 6H), 1.40 (s, 4H), 2.05 (q, J = 7.4 Hz, 2H), 2.11 (s, 2H), 2.83 – 2.99 (m, 3H), 3.45 (q, J = 7.1 Hz, 4H), 3.71 (t, J = 6.5 Hz, 2H), 4.84 (t, J = 7.8 Hz, 2H), 6.53 (t, J = 2.2 Hz, 1H), 6.66 (dt, J = 9.5, 2.0 Hz, 1H), 7.23 – 7.34 (m, 2H), 7.38 – 7.45 (m, 2H), 7.48 (q, J = 2.9 Hz, 3H), 7.59 (dt, J = 9.3, 2.3 Hz, 1H), 7.67 – 7.78 (m, 2H), 8.94 – 9.12 (m, 1H), 11.05 – 11.22 (m, 1H). *Contains residual DCM (2.5 %). **^{13}C{^{1}H} NMR** (101 MHz, $CDCl_3$) δ 12.46, 25.06, 25.22, 27.98, 30.39, 37.12, 44.87, 49.94, 56.06, 97.39, 103.44, 109.62, 120.89 (q, J = 2.5 Hz), 122.42 (q, J = 279.0 Hz), 127.07, 127.26 (d, J = 3.9 Hz), 127.80, 129.82, 130.75, 131.20, 132.06, 133.52, 137.57 (q, J = 30.0 Hz), 139.14, 143.91, 143.94, 150.78, 155.86, 157.88, 161.38. **^{19}F NMR** (376 MHz, $CDCl_3$) δ −54.83 (d, J = 3.0 Hz). **HRMS** calcd. For $C_{36}H_{39}O_4N_3F_3S_2^+$ $[M]^+$ 698.23286, found 698.23114 (− 2.46 ppm). **HPLC** purity, 95.32 % at 254 nm (t_R = 5.4 min).

4e. 3-Carbamoyl-1-(6-(*N*-(4-(7-(diethylamino)-2-oxo-4-(trifluoromethyl)-2*H*-chromen-3-yl)phenyl)methylsulfonamido)hexyl)pyridin-1-ium bromide

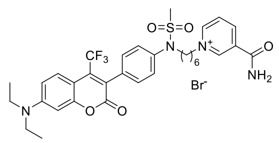

To a heat-dried microwave vial (10 mL loading) under argon atmosphere were added intermediate **3** (60 mg, 0.097 mmol), nicotinamide (153 mg, 1.25 mmol, 13 eq), acetonitrile (2 mL), and the reaction mixture was stirred in a microwave reactor at 140 °C for 1 hour. The reaction mixture was then concentrated in vacuo, and filtered using 15 mL acetonitrile. The filtrate was concentrated to a volume of 2 mL, followed by precipitation using 35 mL diethyl ether. The solid residue was disolved 1 mL dichloromethane and precipitated using 35 mL diethyl eher. The residue was dissolved in 1 mL dichloromethane, and transferred to an automatic silicagel column (DCM:MeOH 0-22%), to get the title compound as a glassy yellow oil (32 mg, 45 % yield). **^{1}H NMR** (400 MHz $CDCl_3$) δ 1.22 (t, J = 7.0 Hz, 6H), 1.38 (s, 4H), 2.05 (s, 2H), 2.88 (s, 3H), 3.35 (s, 2H), 3.44 (q, J = 7.1 Hz, 4H), 3.66 (t, J = 6.3 Hz, 2H), 4.81 (s, 2H), 6.53 (d, J = 2.5 Hz, 1H), 6.64 (dd, J = 9.5, 2.6 Hz, 1H), 7.30 (s, 1H), 7.39 (d, J = 8.1 Hz, 2H), 7.49 – 7.61 (m, 1H), 8.18 (t, J = 7.1 Hz, 1H), 8.94 (s, 1H), 9.13 (d, J = 6.0 Hz, 1H), 9.18 (d, J = 8.0 Hz, 1H), 10.10 (s, 1H). *Contains residual DCM (1.9 %). **^{13}C{^{1}H} NMR** (101 MHz, $CDCl_3$) δ 12.47, 25.08, 25.26, 27.92, 31.19, 37.17, 44.85, 50.02, 62.16, 97.42, 103.43, 109.60, 117.34 – 126.93 (m), 120.91 (d, J = 2.7 Hz), 127.23, 127.85, 128.42, 130.76, 133.55, 133.65, 137.53 (q, *J* = 30.1 Hz), 139.10, 145.08, 145.22, 146.13, 150.77, 155.84, 161.40,

163.42. ^{19}**F NMR** (376 MHz, $CDCl_3$) δ −54.78 (d, J = 2.2 Hz). **HRMS** calcd. For $C_{33}H_{38}O_5N_4F_3S^+$ [M]$^+$ 659.25095, found 659.24939 (− 2.37 ppm). **HPLC** purity, 97.59 % at 254 nm (t_R = 4.7 min).

4f. 1-(6-(*N*-(4-(7-(Diethylamino)-2-oxo-4-(trifluoromethyl)-2*H*-chromen-3-yl)phenyl)methylsulfonamido)hexyl)-4-(trifluoromethyl)pyridin-1-ium bromide

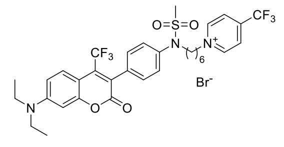

To a heat-dried microwave vial (10 mL loading) under argon atmosphere were added intermediate **3** (57 mg, 0.092 mmol), 4-(trifluoromethyl)pyridine (340 mg, 0.71 mmol, 25 eq), acetonitrile (2 mL), and the reaction mixture was stirred in a microwave reactor at 135 °C for 1 hour. The reaction mixture was then concentrated in vacuo. The solid residue was disolved 1 mL dichloromethane and transferred to an automatic silicagel column (DCM:MeOH 0-17%), to get the title compound as an orange oil (46 mg, 65 % yield).

^{1}**H NMR** (400 MHz, $CDCl_3$) δ 1.24 (t, J = 7.1 Hz, 6H), 1.42 (d, J = 4.8 Hz, 4H), 1.90 (s, 2H), 2.05 (t, J = 7.2 Hz, 2H), 2.88 (s, 3H), 3.46 (q, J = 7.1 Hz, 4H), 3.70 (t, J = 6.3 Hz, 2H), 5.09 (t, J = 7.4 Hz, 2H), 6.54 (d, J = 2.6 Hz, 1H), 6.66 (dd, J = 9.4, 2.7 Hz, 1H), 7.31 (d, J = 6.7 Hz, 2H), 7.35 – 7.47 (m, 2H), 7.59 (dd, J = 9.4, 2.4 Hz, 1H), 8.29 (d, J = 6.2 Hz, 2H), 9.94 (d, J = 6.2 Hz, 2H). ^{13}**C{^{1}H} NMR** (101 MHz, $CDCl_3$) δ 12.55, 24.75, 25.10, 27.67, 31.63, 37.13, 44.98, 49.74, 62.63, 97.52, 103.53, 109.75, 120.94 (d, *J* = 2.4 Hz), 121.00 (q, J = 275.4 Hz), 122.53 (q, J = 279.0 Hz), 124.96 (d, J = 3.5 Hz), 127.38 (d, J = 4.0 Hz), 127.89, 130.90, 133.74, 137.73 (q, J = 30.1 Hz), 139.04, 145.12 (q, J = 36.9 Hz), 147.60, 150.92, 155.99, 161.50. ^{19}**F NMR** (376 MHz, $CDCl_3$) δ −65.08, −54.86. **HRMS** calcd. For $C_{33}H_{36}O_4N_3F_6S^+$ [M]$^+$ 684.23252, found 684.23079 (− 2.53 ppm). **HPLC** purity, 97.88 % at 254 nm (t_R = 5.2 min).

5. 4-(4-(4-(3-Iodopropyl)phenoxy)butoxy)-7*H*-furo[3,2-*g*]chromen-7-one

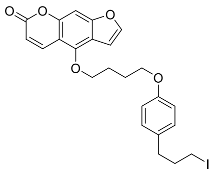

Prepared according to the literature procedure.[19] ^{1}**H NMR** (400 MHz, $CDCl_3$) δ 8.15 (dd, J = 9.8, 0.5 Hz, 1H), 7.58 (d, J = 2.4 Hz, 1H), 7.14 (s, 1H), 7.10 (t, J = 5.7 Hz, 2H), 6.96 (dd, J = 2.4, 1.0 Hz, 1H), 6.87 – 6.78 (m, 2H), 6.25 (d, J = 9.8 Hz, 1H), 4.55 (t, J = 6.0 Hz, 2H), 4.05 (t, J = 5.8 Hz, 2H), 3.16 (t, J = 6.8 Hz, 2H), 2.67 (t, J = 7.2 Hz, 2H), 2.10 (dd, J = 14.4, 6.8 Hz, 4H), 2.07 – 1.93 (m, 2H). NMR data is in accordance with the literature data.[19]

6a. (3-(4-(4-((7-Oxo-7*H*-furo[3,2-*g*]chromen-4-yl)oxy)butoxy)phenyl)propyl)triphenylphosphonium iodide

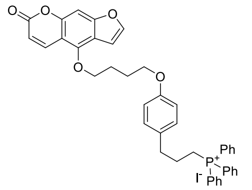

To a heat-dried microwave vial (10 mL loading) under argon atmosphere were added intermediate **5** (40 mg, 0.077 mmol), triphenylphosphine (160 mg, 0.62 mmol, 8 eq), acetonitrile (2 mL), and the reaction mixture was stirred in a microwave reactor at 140 °C for 1.5 hours. The reaction mixture was then

concentrated in vacuo until dryness. The impurities were separated from the product by flash chromatography, eluent DCM:MeOH to get the title compound as a crispy white foam (59 mg, 98% yield). **^{1}H NMR** (400 MHz, $CDCl_3$) δ 8.15 (dd, J = 9.8, 0.5 Hz, 1H), 7.82 – 7.72 (m, 9H), 7.71 – 7.63 (m, 6H), 7.59 (d, J = 2.4 Hz, 1H), 7.16 (s, 1H), 7.13 (d, J = 3.9 Hz, 2H), 6.96 (dd, J = 2.4, 1.0 Hz, 1H), 6.79 (t, J = 5.8 Hz, 2H), 6.23 (d, J = 9.8 Hz, 1H), 4.54 (t, J = 6.0 Hz, 2H), 4.04 (t, J = 5.8 Hz, 2H), 3.85 – 3.68 (m, 2H), 2.99 (t, J = 7.2 Hz, 2H), 2.13 – 1.99 (m, 4H), 1.93 (dd, J = 15.6, 7.7 Hz, 2H). NMR data is in accordance with the literature data.[19] **HRMS** calcd. For $C_{41}H_{36}NO_5^+$ $[M]^+$ 653.24514, found 653.24414 (− 1.53 ppm) **HPLC** purity, >99.99 % at 254 nm (t_R = 7.5 min).

6b. 1-(3-(4-(4-((7-Oxo-7*H*-furo[3,2-*g*]chromen-4-yl)oxy)butoxy)phenyl)propyl)-3,5-diphenylpyridin-1-ium iodide

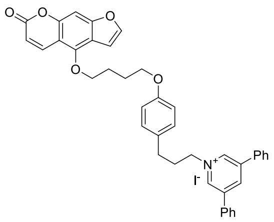

To a heat-dried microwave vial (30 mL loading) under argon atmosphere were added intermediate **5** (249 mg, 0.482 mmol), 3,5-diphenylpyridine (171 mg, 0.71 mmol, 1.47 eq), acetonitrile (8 mL), and the reaction mixture was stirred in a microwave reactor at 150 °C for 2 hours. The reaction mixture was then concentrated in vacuo to approximately 1 mL. The mixture had precipitated upon cooling, and was washed with 70 mL diethyl ether, redisolved in 2 mL acetonitrile and precipitated using diethyl ether, to get the title compound as a pale yellow-brownish solid (343 mg, 95 % yield). **^{1}H NMR** (400 MHz, $CDCl_3$) δ 9.44 (s, 2H), 8.50 (s, 1H), 8.09 (d, *J* = 9.8 Hz, 1H), 7.89 (d, *J* = 6.8 Hz, 5H), 7.59 – 7.56 (m, 1H), 7.52 (dt, *J* = 6.8, 4.6 Hz, 6H), 7.08 (s, 2H), 7.06 (s, 1H), 6.95 (d, *J* = 1.5 Hz, 1H), 6.65 (d, *J* = 8.5 Hz, 2H), 6.18 (d, *J* = 9.8 Hz, 1H), 5.33 (t, *J* = 7.2 Hz, 2H), 4.52 (t, *J* = 5.9 Hz, 2H), 3.92 (t, *J* = 5.7 Hz, 2H), 2.80 (t, *J* = 7.4 Hz, 2H), 2.60 – 2.28 (m, 2H), 2.13 – 1.80 (m, 4H). *Contains residual diethyl ether (5.0 %). **^{13}C{^{1}H} NMR** (101 MHz, $CDCl_3$) δ 161.37, 158.38, 157.32, 152.75, 148.99, 145.00, 141.53, 140.46, 139.82, 139.47, 132.82, 131.97, 130.82, 129.95, 129.53, 127.92, 114.57, 113.24, 112.50, 106.72, 105.28, 93.89, 72.60, 67.32, 62.04, 33.71, 31.32, 27.01, 26.00. **HRMS** calcd. For $C_{41}H_{36}NO_5^+$ $[M]^+$ 622.25880, found 622.25793 (− 1.40 ppm). **HPLC** purity, 97.65 % at 254 nm (t_R = 5.7 min).

6c. 1-(3-(4-(4-((7-Oxo-7*H*-furo[3,2-*g*]chromen-4-yl)oxy)butoxy)phenyl)propyl)pyridin-1-ium iodide

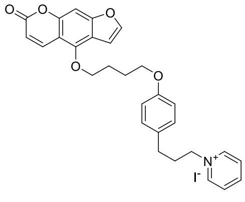

To a heat-dried pressure tube under argon atmosphere were added intermediate **5** (50 mg, 0.097 mmol), pyridine (78 mg, 0.97, 10 eq), anhydrous acetonitrile (2 mL), and the reaction mixture was stirred over night at 65 °C, to full conversion. The reaction mixture was concentrated in vacuo, followed by the removal of residual reagent under high vacuum using an oil pump. The flask containing product was left open over night in order to quantitatively remove the residual excess pyridine, to get the title

compound as a yellow oil (59 mg, quantitative yield). **^{1}H NMR** (400 MHz, Acetone-*d*$_6$) δ 9.43 (d, *J* = 5.5 Hz, 2H), 8.79 – 8.69 (m, 1H), 8.27 (t, *J* = 7.1 Hz, 2H), 8.23 (dd, *J* = 9.8, 0.5 Hz, 1H), 7.87 (d, *J* = 2.4 Hz, 1H), 7.29 (dd, *J* = 2.4, 0.9 Hz, 1H), 7.19 (s, 1H), 7.15 (dd, *J* = 9.2, 6.1 Hz, 2H), 6.85 – 6.79 (m, 2H), 6.21 (d, *J* = 9.8 Hz, 1H), 5.02 – 4.97 (m, 2H), 4.68 (t, *J* = 5.9 Hz, 2H), 4.09 (t, *J* = 5.9 Hz, 2H), 2.80 – 2.74 (m, 2H), 2.50 – 2.40 (m, 2H), 2.10 (ddd, *J* = 11.0, 8.7, 3.5 Hz, 4H). **^{13}C{^{1}H} NMR** (101 MHz, Acetone-*d*$_6$) δ 160.86, 159.16, 158.44, 150.11, 146.62, 146.29, 145.96, 140.05, 133.15, 130.24, 129.26, 115.35, 114.02, 113.23, 107.26, 106.42, 93.90, 73.53, 68.15, 62.12, 34.09, 31.86, 27.50, 26.63. **HRMS** (ESI+): calcd. For $C_{29}H_{28}NO_5^+$ $[M]^+$ 470.19620, found 470.19599 (− 0.45 ppm). **HPLC** purity, 97.26 % at 254 nm (t_R = 4.3 min).

6d. 3-(3-(4-(4-((7-Oxo-7*H*-furo[3,2-*g*]chromen-4-yl)oxy)butoxy)phenyl)propyl)-5-phenylthiazol-3-ium iodide

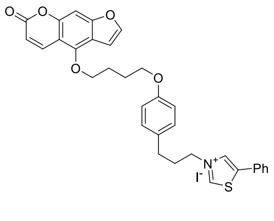

To a heat-dried microwave vial under argon atmosphere were added intermediate **5** (51.8 mg, 0.1 mmol), **S2** (62 mg, 0.39 mmol, 3.9 eq), anhydrous acetonitrile (2 mL), and the reaction mixture was stirred in the microwave reactor for 45 min at 130 °C, then 1 h at 150 °C until 70% conversion was reached according to NMR. The reaction mixture was concentrated in vacuo. The impurities were separated from the product by flash chromatography, eluent DCM:MeOH to get the title compound as an off-white solid (44 mg, 65% yield). **^{1}H NMR** (400 MHz, DMSO-*d*$_6$) δ 10.18 (s, 1H), 9.03 (d, *J* = 1.2 Hz, 1H), 8.19 (d, *J* = 9.8 Hz, 1H), 8.04 (d, *J* = 1.6 Hz, 1H), 7.81 (d, *J* = 1.8 Hz, 1H), 7.79 (d, *J* = 1.4 Hz, 1H), 7.63 – 7.51 (m, 3H), 7.35 (s, 1H), 7.35 – 7.32 (m, 1H), 7.11 (d, *J* = 8.6 Hz, 2H), 6.81 (d, *J* = 8.6 Hz, 2H), 6.30 (d, *J* = 9.8 Hz, 1H), 4.58 (t, *J* = 7.3 Hz, 4H), 3.98 (t, *J* = 5.8 Hz, 2H), 2.62 (t, *J* = 7.6 Hz, 2H), 2.33 – 2.23 (m, 2H), 2.01 – 1.86 (m, 4H). **^{13}C{^{1}H} NMR** (101 MHz, DMSO-*d*$_6$) δ 160.49, 157.66, 157.60, 156.78, 156.54, 151.91, 148.37, 144.49, 143.25, 138.97, 131.87, 130.93, 130.69, 129.22, 128.71, 126.36, 113.96, 112.51, 111.59, 105.86, 104.71, 92.86, 71.87, 66.65, 55.27, 39.93, 39.73, 39.52, 39.32, 39.11, 31.12, 30.83, 26.21, 25.24. **HRMS** (ESI+): calcd. For $C_{33}H_{30}NO_5S^+$ $[M]^+$ 552.18392, found 552.18364 (− 0.51 ppm). **HPLC** purity, 97.99 % at 254 nm (t_R = 5.3 min).

6e. 3-Carbamoyl-1-(3-(4-(4-((7-oxo-7*H*-furo[3,2-*g*]chromen-4-yl)oxy)butoxy)phenyl)propyl)pyridin-1-ium iodide

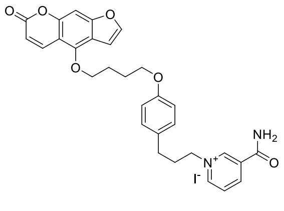

To a heat-dried flask under argon atmosphere were added intermediate **5** (50 mg, 0.097 mmol), nicotinamide (18 mg, 0.15 mmol, 1.5 eq), anhydrous acetonitrile (1.3 mL), and the reaction mixture was sealed, and stirred for two hours at 80 °C, followed by replenishing with acetonitrile (2.4 mL), resealing, and stirring over night at 80 °C. The solvent had evaporated and the resulting solid was

partially dissolved and washed with 2 × 2 mL acetonitrile to get the title compound as an off-white solid (32.3 mg, 52 % yield). **^{1}H NMR** (400 MHz, DMSO-d_6) δ 9.44 (s, 1H), 9.18 (d, *J* = 6.1 Hz, 1H), 8.90 (d, *J* = 8.2 Hz, 1H), 8.52 (s, 1H), 8.27 – 8.22 (m, 1H), 8.19 (d, *J* = 9.9 Hz, 1H), 8.17 (s, 1H), 8.05 (d, *J* = 2.4 Hz, 1H), 7.37 (s, 1H), 7.35 (d, *J* = 2.3 Hz, 1H), 7.10 (d, *J* = 8.6 Hz, 2H), 6.81 (d, *J* = 8.6 Hz, 2H), 6.29 (d, *J* = 9.8 Hz, 1H), 4.67 (t, *J* = 7.4 Hz, 2H), 4.58 (d, *J* = 5.8 Hz, 2H), 4.03 (d, *J* = 6.0 Hz, 2H), 2.63 – 2.56 (m, 2H), 2.28 – 2.15 (m, 2H), 1.95 (s, 4H). *Contains residual acetonitrile (6.6 %). **^{13}C{^{1}H} NMR** (101 MHz, DMSO-d_6) δ 162.78, 160.12, 157.66, 156.92, 152.12, 148.72, 146.39, 145.95, 144.84, 143.26, 139.48, 133.78, 132.02, 129.16, 127.83, 115.15, 114.37, 112.90, 112.29, 105.96, 105.68, 93.21, 72.29, 67.04, 61.06, 32.18, 30.74, 26.20, 25.31. **HRMS** calcd. For $C_{30}H_{29}N_2O_6^+$ [M]$^+$ 513,20201 , found 513.20161 (− 0.79 ppm). **HPLC** purity, 98.03 % at 254 nm (t_R = 4.4 min).

6f. 1-(3-(4-(4-((7-Oxo-7*H*-furo[3,2-*g*]chromen-4-yl)oxy)butoxy)phenyl)propyl)-4-(trifluoromethyl)pyridin-1-ium iodide

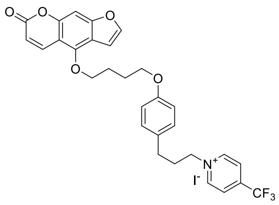

To a heat-dried pressure tube under argon atmosphere were added intermediate **5** (50 mg, 0.097 mmol), 4-(trifluoromethyl)pyridine (234 mg, 1.59 mmol, 16 eq), anhydrous acetonitrile (4 mL), and the reaction mixture was stirred at 100 °C for 72 h. The reaction mixture was concentrated in vacuo, followed by the removal of residual reagent under high vacuum using an oil pump, to get the title compound as an orange oil (64 mg, 98 %). **^{1}H NMR** (400 MHz, Acetone-d_6) δ 9.85 (d, *J* = 6.6 Hz, 2H), 8.65 (d, *J* = 6.2 Hz, 2H), 8.23 (dd, *J* = 9.8, 0.6 Hz, 1H), 7.88 (d, *J* = 2.4 Hz, 1H), 7.29 (dd, *J* = 2.4, 1.0 Hz, 1H), 7.18 (d, *J* = 8.7 Hz, 2H), 7.16 (d, *J* = 0.7 Hz, 1H), 6.81 (d, *J* = 8.7 Hz, 2H), 6.21 (d, *J* = 9.8 Hz, 1H), 5.24 – 5.03 (m, 2H), 4.68 (t, *J* = 6.0 Hz, 2H), 4.08 (dd, *J* = 8.4, 3.5 Hz, 2H), 2.84 – 2.78 (m, 2H), 2.57 – 2.47 (m, 2H), 2.09 (dd, *J* = 7.5, 3.5 Hz, 4H). *Contains residual DCM (1.9 %). **^{19}F NMR** (376 MHz, Acetone-d_6) δ −65.81. **^{13}C{^{1}H} NMR** (101 MHz, Acetone) δ13C NMR (101 MHz, Acetone) δ 161.61, 159.93, 159.21, 154.53, 150.87, 149.12, 147.05, 140.80, 133.82, 131.02, 126.67 (q, J = 3.5 Hz), 116.08, 114.77, 113.99, 108.02, 107.17, 94.67, 74.26, 68.87, 63.78, 61.29, 34.72, 32.60, 28.26, 27.38, 21.58. **HRMS** (ESI+): calcd. For $C_{30}H_{27}F_3NO_5^+$ [M]$^+$ 538.18358, found 538.18303 (− 1.03 ppm). **HPLC** purity, 97.82 % at 254 nm (t_R = 4.5 min).

7b. 3,5-Diphenyl-1-propylpyridin-1-ium chloride

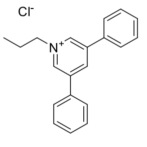

To a solution of propylbromide (220 mg, 1.8 mmol, 5.8 eq) in acetonitrile (2 mL) was added **S1** (71 mg, 0.31 mmol) and the reaction mixture was stirred at 130 °C for 30 min in a microwave reactor. The resulting solution was concentrated in vacuo, and the white solid residue was redissolved in 0.5 mL DCM, and precipitated with diethyl ether (30 mL), then washed with diethyl ether (30 mL) to get the pure bromide. For anion exchange, the above bromide was

dissolved in 20 mL DCM, and the organic layer was washed with a 2.5 M aqueous NaCl (5 x 20 mL), concentrated in vacuo, followed by redissolution in DCM, filtration through a HPLC filter, and slow evaporation of solvent to get the title compound as white crystals, suitable for single-crystal X-ray diffraction analysis (38 mg, 40% yield). ^{1}**H NMR** (400 MHz, $CDCl_3$) δ 9.74 (s, 2H), 8.53 (s, 1H), 7.95 – 7.87 (m, 4H), 7.59 – 7.42 (m, 6H), 5.34 (t, *J* = 7.3 Hz, 2H), 2.20 – 2.06 (m, 2H), 1.04 (t, *J* = 7.3 Hz, 3H). ^{13}**C NMR** (101 MHz, $CDCl_3$) δ 141.26, 141.25, 139.16, 132.99, 130.67, 129.93, 127.78, 63.48, 25.78, 10.71. **HRMS** (ESI+): calcd. For $C_{20}H_{20}N^+$ $[M]^+$ 274,15903, found 274.15864 (− 1.42 ppm).

8a. Butyltriphenylphosphonium hexafluorophosphate(V)

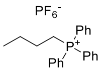

To a solution of butylbromide (138 mg, 1 mmol, 1 eq) in acetonitrile (1.5 mL) was added triphenylphosphine (262 mg, 1 mmol) and the reaction mixture was stirred at 120 °C for 1 h in a microwave reactor. The resulting solution was concentrated in vacuo, and the white solid residue was redisolved in 0.5 mL DCM, precipitated with diethyl ether (15 mL), and the solid washed with diethyl ether (2 x 15 mL). The resulting residue was dissolved in 10 mL DCM, and the organic layer was washed with a 0.2 M solution of KPF_6 (4 x 5 mL), concentrated in vacuo, followed by redissolution in DCM, filtration through a HPLC filter, and the solvent was left to evaporate to get the title compound as a white crystalline solid (187.1 mg, 40% yield). ^{1}**H NMR** (400 MHz, $CDCl_3$) δ 0.82 – 1.02 (m, 3H), 1.59 (q, *J* = 3.7 Hz, 4H), 2.93 – 3.51 (m, 2H), 7.58 – 7.78 (m, 12H), 7.78 – 8.01 (m, 3H). *Contains residual DCM (0.4 %). ^{13}**C{^{1}H} NMR** (101 MHz, $CDCl_3$) δ 13.57, 22.07 (d, *J* = 51.7 Hz), 23.70 (d, *J* = 16.5 Hz), 24.57 (d, *J* = 4.4 Hz), 118.07 (d, *J* = 86.2 Hz), 130.75 (d, *J* = 12.6 Hz), 133.45 (d, *J* = 9.9 Hz), 135.39 (d, *J* = 3.0 Hz). ^{19}**F NMR** (376 MHz, $CDCl_3$) δ −72.92 (d, *J* = 713.2 Hz). ^{31}**P NMR** (162 MHz, $CDCl_3$) δ −144.32 (hept, *J* = 712.65 Hz), 23.57. **HRMS** (ESI+): calcd. For $C_{22}H_{24}P^+$ $[M]^+$ 319.16101, found 319.16081 (− 0.64 ppm). **HPLC** purity, 92.99 % at 254 nm (t_R = 5.7 min).

8b. 1-Butyl-3,5-diphenylpyridin-1-ium hexafluorophosphate(V)

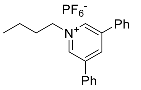

To a solution of butylbromide (105 mg, 0.766 mmol, 2.5 eq) in acetonitrile (1.5 mL) was added 3,5-diphenylpyridine (100 mg , 0.303 mmol) and the reaction mixture was stirred at 120 °C for 50 min in a microwave reactor. The resulting solution was concentrated to 0.7 mL *in vacuo,* and was washed with diethyl ether (3 x 15 mL). The resulting residue was dissolved in 5-10 mL DCM, and the organic layer was washed with a 0.2 M solution of KPF_6 (4 x 5 mL), concentrated *in vacuo*, followed by redislution in DCM, filtration through a HPLC filter, and was left to evaporate to get the title compound as white needle-like crystals (86.5 mg, 66% yield). ^{1}**H NMR** (400 MHz, DMSO-*d*$_6$) δ 9.46 (d, J = 1.6 Hz, 2H), 9.14 (t, J = 1.6 Hz, 1H), 8.16 – 7.93 (m, 4H), 7.73 – 7.54 (m, 6H), 4.71 (t, J = 7.6 Hz, 2H), 2.18 – 1.98 (m, 2H), 1.53 – 1.29 (m, 2H), 0.96 (t, J = 7.4 Hz, 3H). *Contains residual DCM (0.4 %). ^{13}**C{^{1}H} NMR** (101 MHz, DMSO-*d*$_6$) δ 141.08, 139.87, 139.80, 133.28, 130.21, 129.39, 127.86,

61.21, 32.74, 19.01, 13.41. **^{19}F NMR** (376 MHz, DMSO-d_6) δ −70.16 (d, J = 711 Hz). **^{31}P NMR** (162 MHz, DMSO-d_6) δ −144.2 (hept, J = 711 Hz. **HRMS** (ESI+): calcd. For $C_{21}H_{22}N^+$ [M]$^+$ 288.17468, found 288.17441 (− 0.92 ppm). **HPLC** purity, > 99.99 % at 254 nm (t_R = 5.7 min).

9a. 1-Decyl-triphenylphosphonium bromide

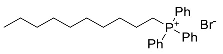

To a solution of decylbromide (336 mg, 1.52 mmol, 3.6 eq) in acetonitrile (2 mL) was added triphenylphosphine (110 mg, 0.42 mmol) and the reaction mixture was stirred at 130 °C for 1 h in a microwave reactor. The resulting solution was reduced in vacuo to 1 mL*,* and precipitated with diethyl ether (40 mL). The resulting residue was dissolved in 0.7 mL DCM, precipitated with diethyl ether (40 mL), the title compound as a thick transparent oil (188 mg, 93% yield). **^{1}H NMR** (400 MHz, $CDCl_3$) δ 0.83 (p, J = 6.9 Hz, 3H), 1.09 – 1.36 (m, 12H), 1.61 (q, J = 7.0, 6.0 Hz, 4H), 3.68 (dt, J = 18.1, 9.7 Hz, 2H), 7.63 – 7.96 (m, 15H). **^{13}C{^{1}H} NMR** (101 MHz, $CDCl_3$) δ 13.95, 22.43, 22.46, 22.92, 29.02, 29.04, 29.28, 30.30 (d, J = 15.5 Hz), 31.64, 53.46, 118.09 (d, J = 85.8 Hz), 130.43 (d, J = 12.5 Hz), 133.47 (d, J = 10.0 Hz), 134.98 (d, J = 3.1 Hz). **^{31}P NMR** (162 MHz, $CDCl_3$) δ 24.05. **HRMS** (ESI+): calcd. For $C_{28}H_{36}P^+$ [M]$^+$ 403.25491, found 403.25452 (− 0.98 ppm)**. HPLC** purity, 99.46 % at 254 nm (t_R = 8.2 min).

9b. 1-decyl-3,5-diphenylpyridin-1-ium bromide

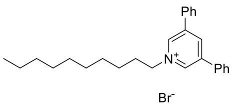

To a solution of decylbromide (429 mg, 1.94 mmol, 5.9 eq) in acetonitrile (2 mL) was added 3,5-diphenylpyridine (77 mg, 0.33 mmol) and the reaction mixture was stirred at 130 °C for 1 h in a microwave reactor. The resulting solution was concentrated to 0.7 mL in vacuo, and was triturated with diethyl ether (3 x 20 mL) to get the title compound as a pale-yellow solid (90 mg, 60 % yield). **^{1}H NMR** (400 MHz, $CDCl_3$) δ 0.84 (t, J = 6.8 Hz, 3H), 1.08 – 1.34 (m, 11H), 1.38 (q, J = 7.7 Hz, 2H), 2.03 (q, J = 7.6 Hz, 2H), 5.39 (t, J = 7.4 Hz, 2H), 7.51 (dq, J = 14.0, 7.1 Hz, 6H), 7.87 – 8.08 (m, 4H), 8.51 (s, 1H), 9.64 (s, 2H). **^{13}C{^{1}H} NMR** (101 MHz, $CDCl_3$) δ 14.06, 22.59, 26.05, 29.16, 29.19, 29.31, 29.44, 31.76, 32.45, 62.10, 127.75, 129.75, 130.53, 132.78, 139.09, 140.86, 140.98. **HRMS** (ESI+): calcd. For $C_{27}H_{34}N^+$ [M]$^+$ 372.26858, found 372.26823 (− 0.93 ppm)**. HPLC** purity, 95.95 % at 254 nm (t_R = 8.2 min).

NMR spectra (Figures S43–S104)

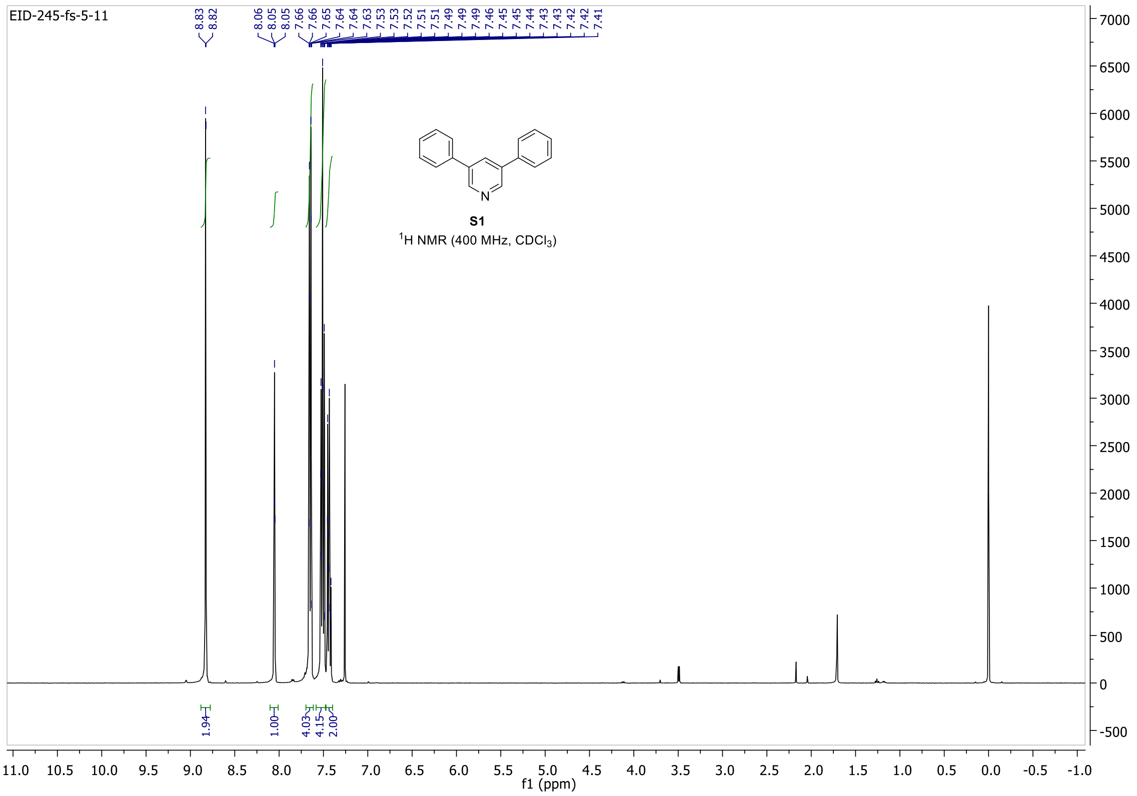

Figure S 43. ^{1}H NMR spectrum of compound **S1**.

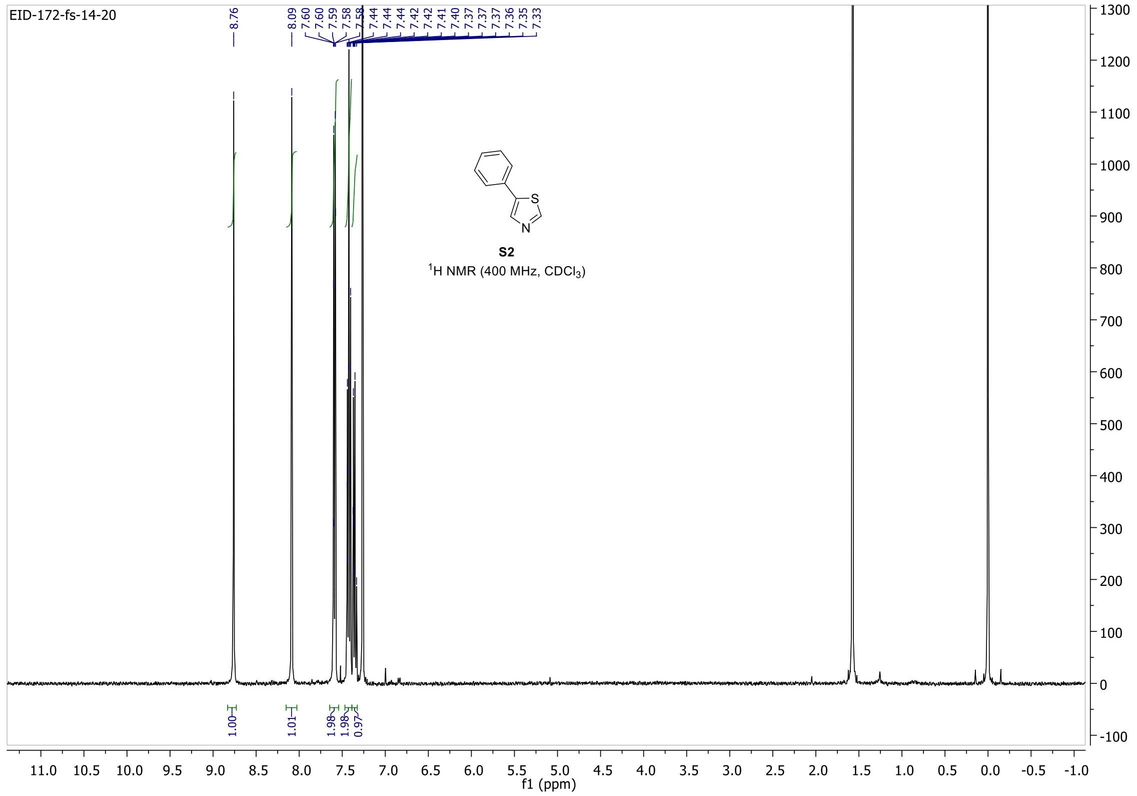

Figure S 44. ^{1}H NMR spectrum of compound **S2**.

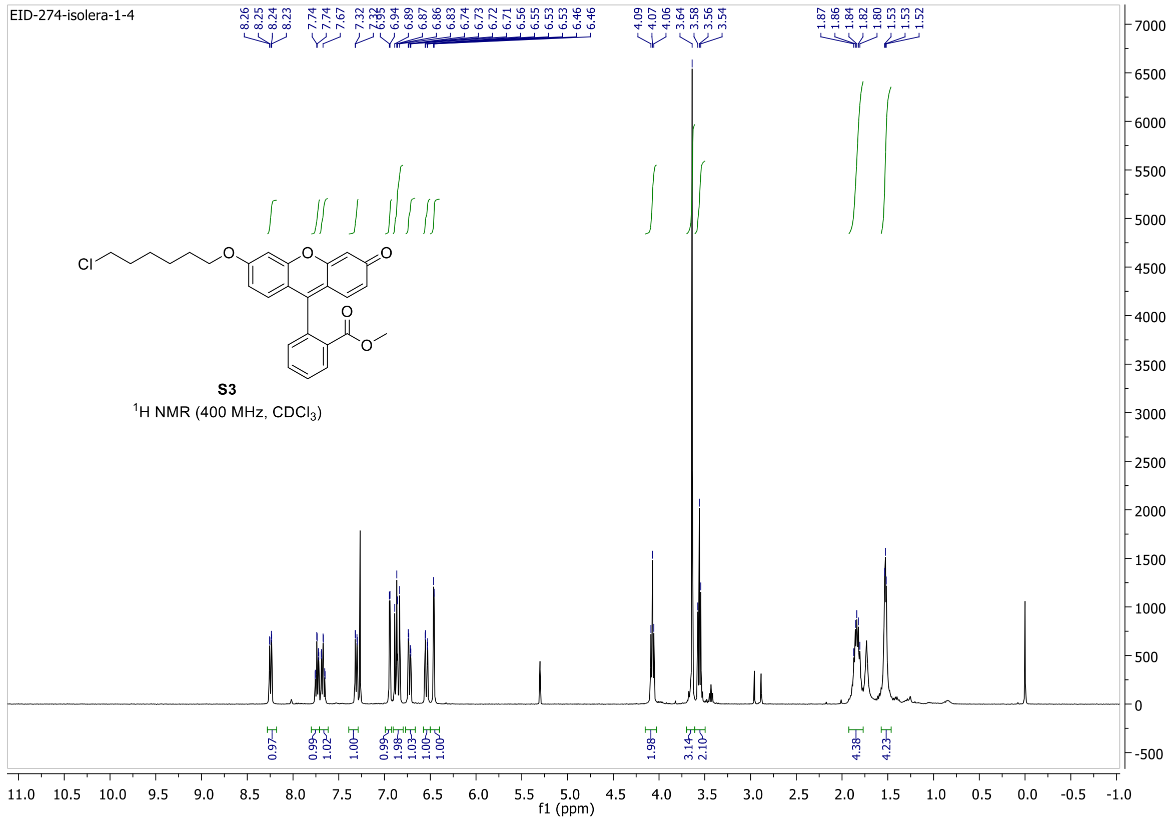

Figure S 45. ^{1}H NMR spectrum of compound **S3**.

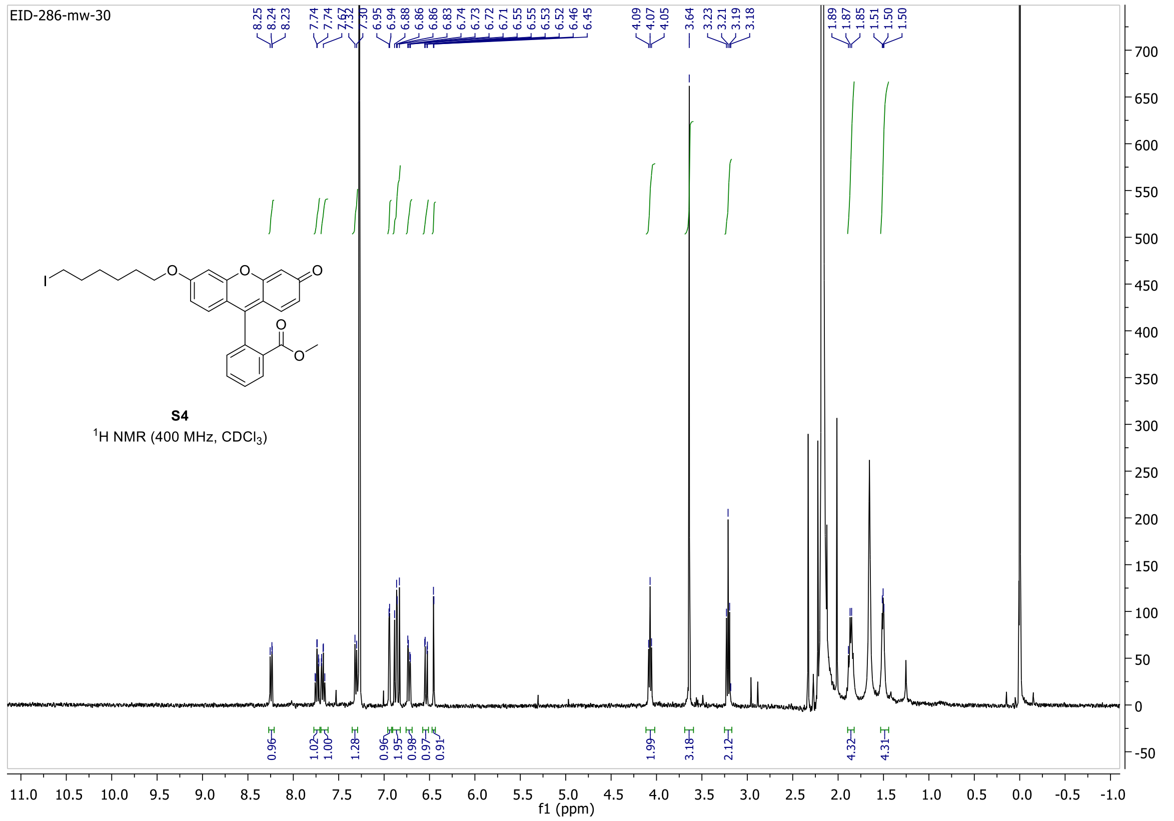

Figure S 46. ^{1}H NMR spectrum of compound **S4**.

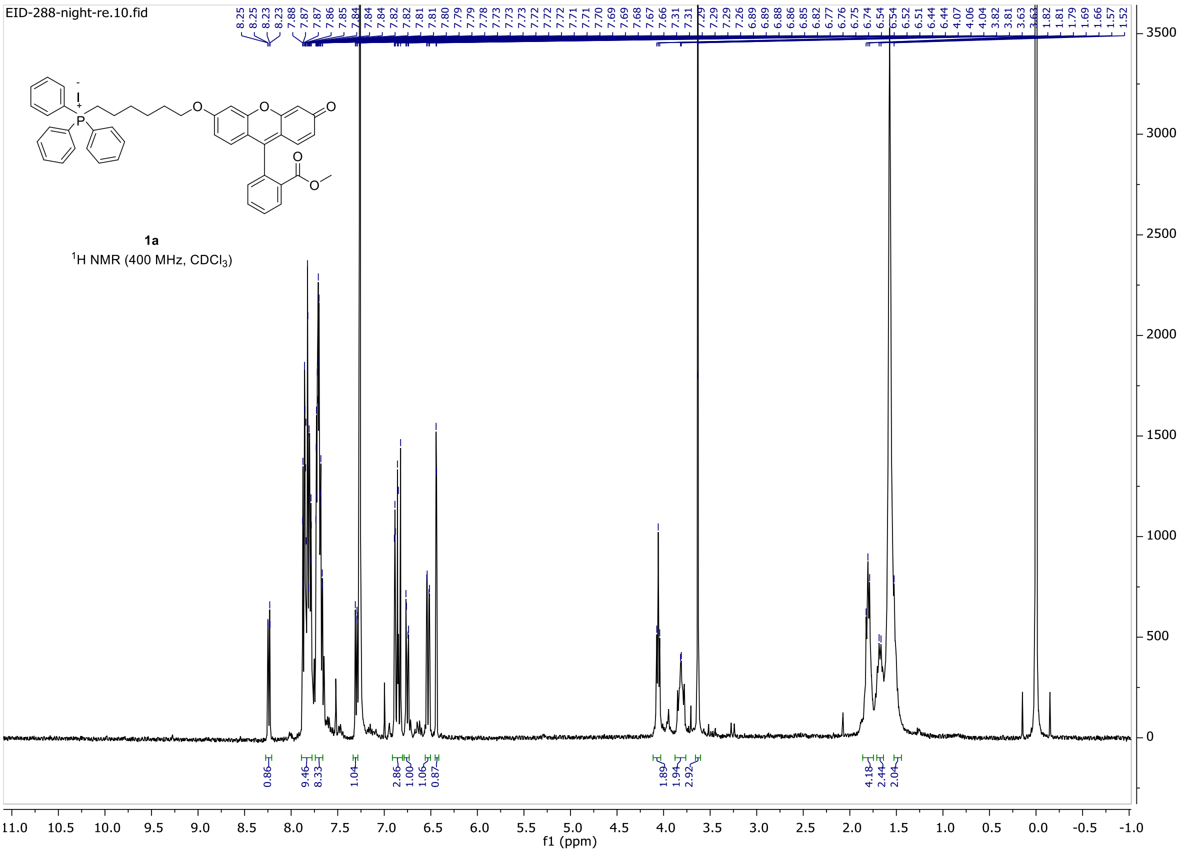

Figure S 47. ^{1}H NMR spectrum of compound **1a**.

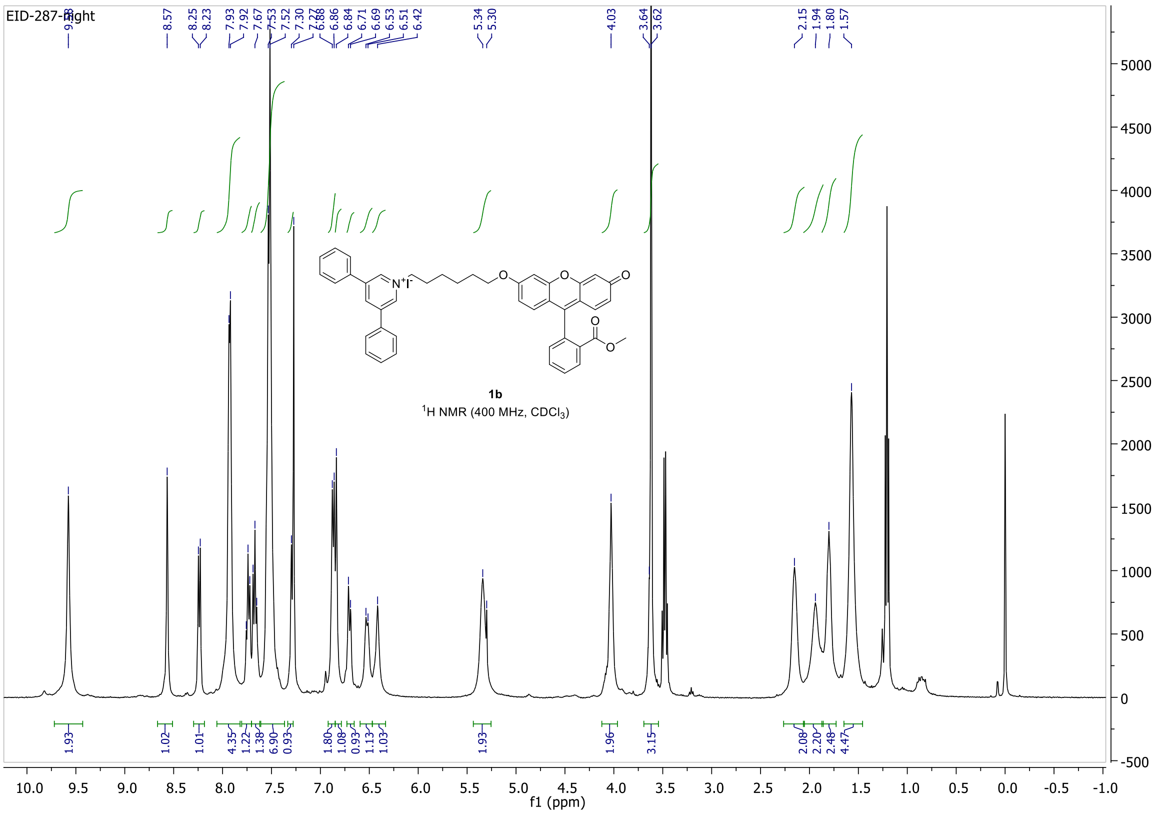

Figure S 48. ^{1}H NMR spectrum of compound **1b**.

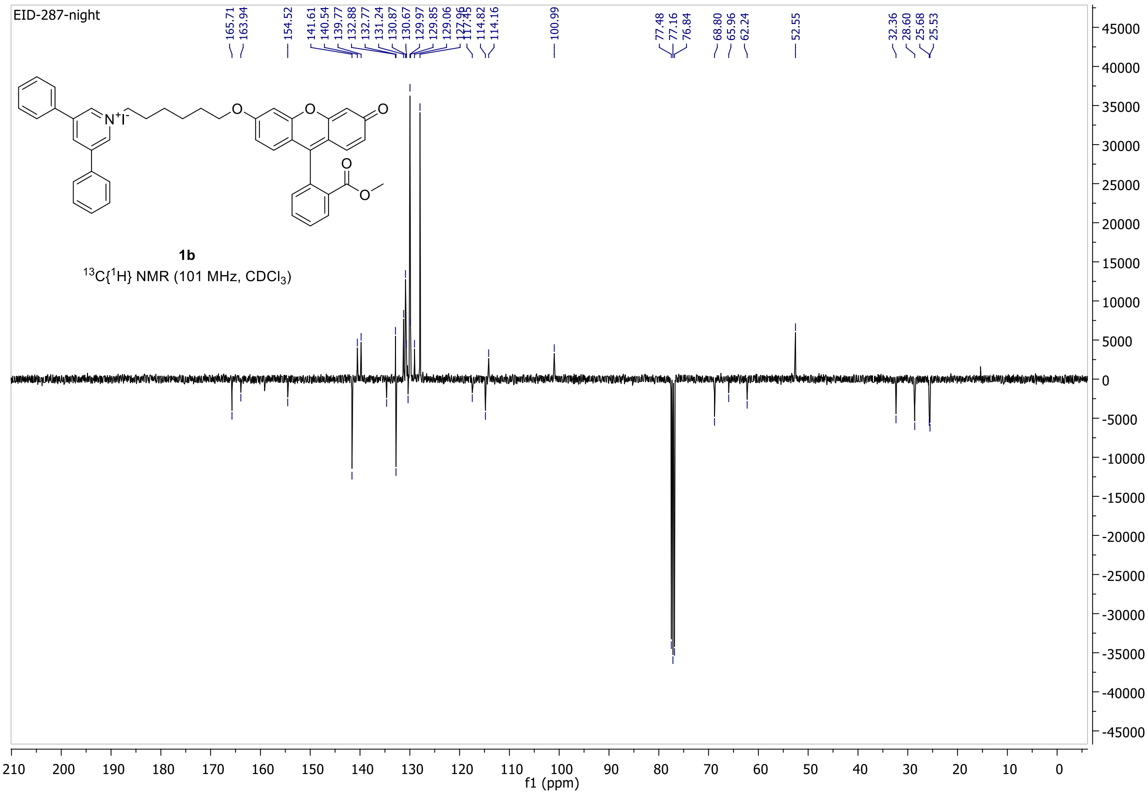

Figure S 49. ^{13}C Attached Proton Test (APT) NMR spectrum of compound **1b**.

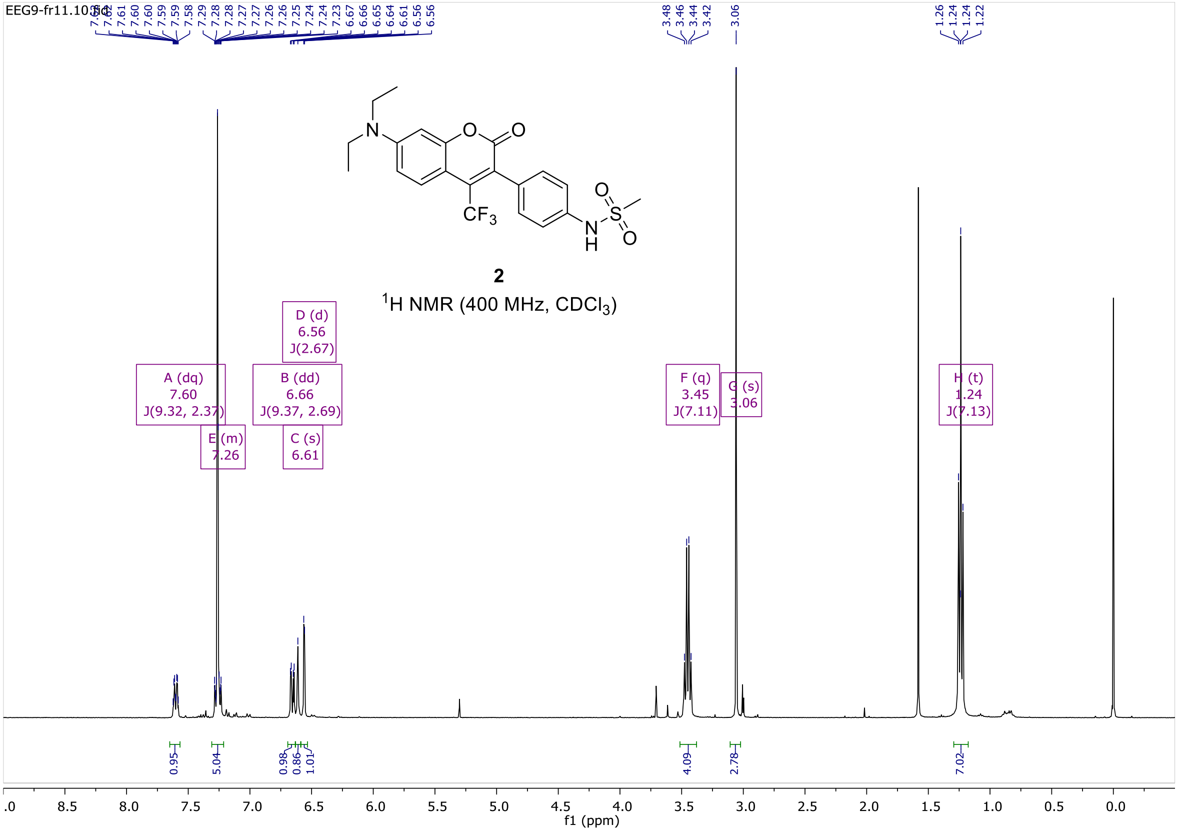

Figure S 50. ^{1}H NMR spectrum of compound **2**.

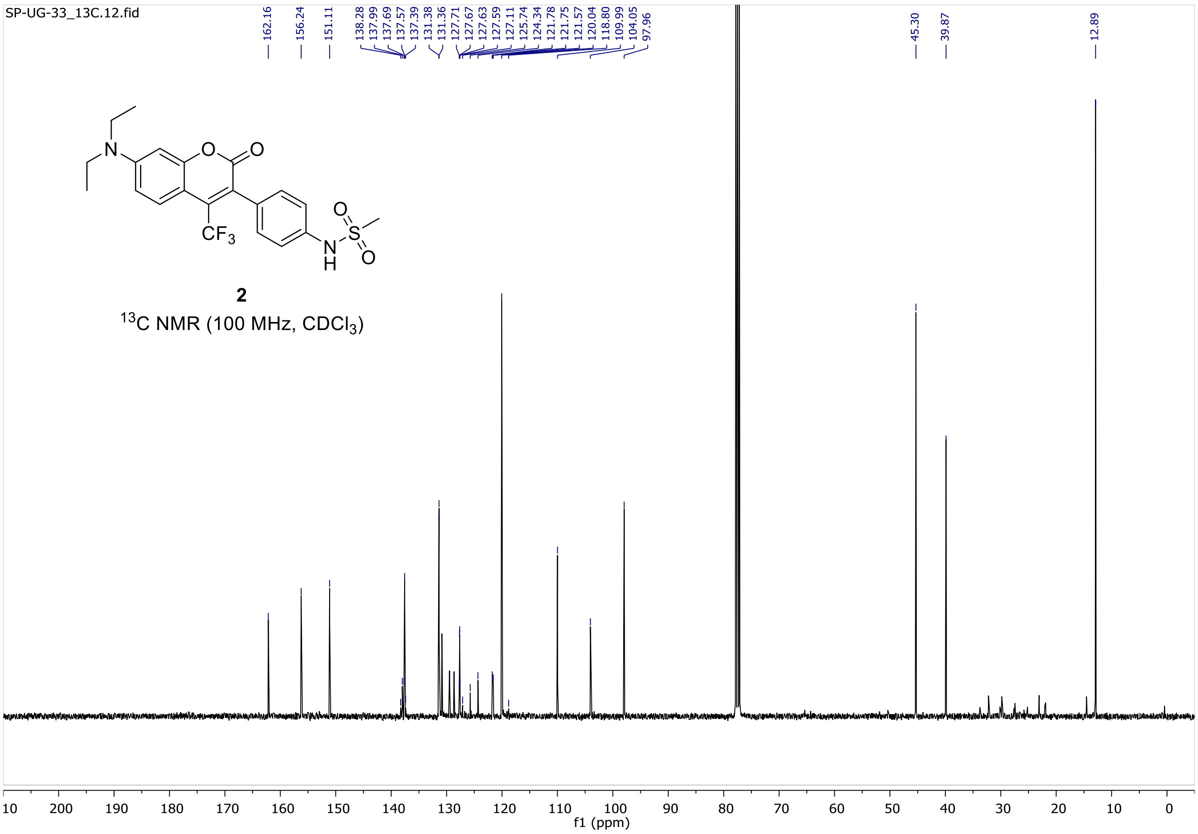

Figure S 51. ^{13}C NMR spectrum of compound **2**.

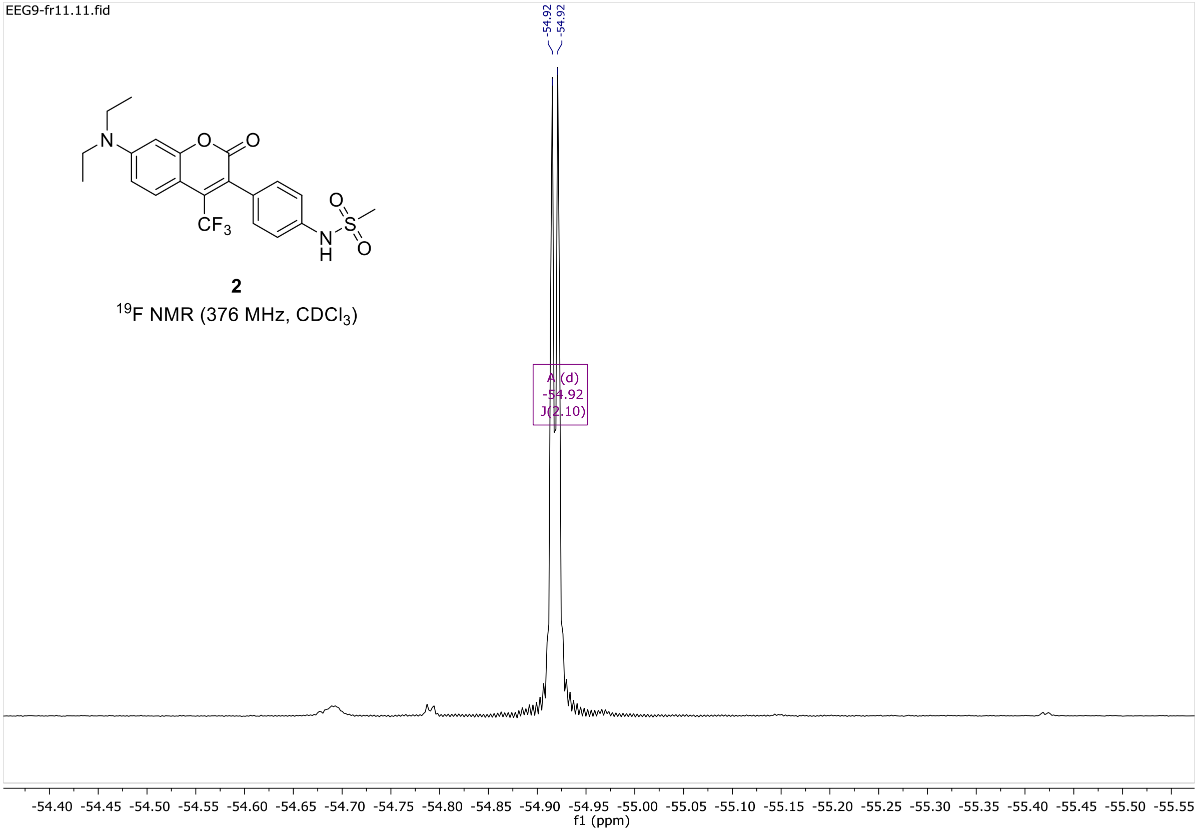

Figure S 52. ^{19}F NMR spectrum of compound **2**.

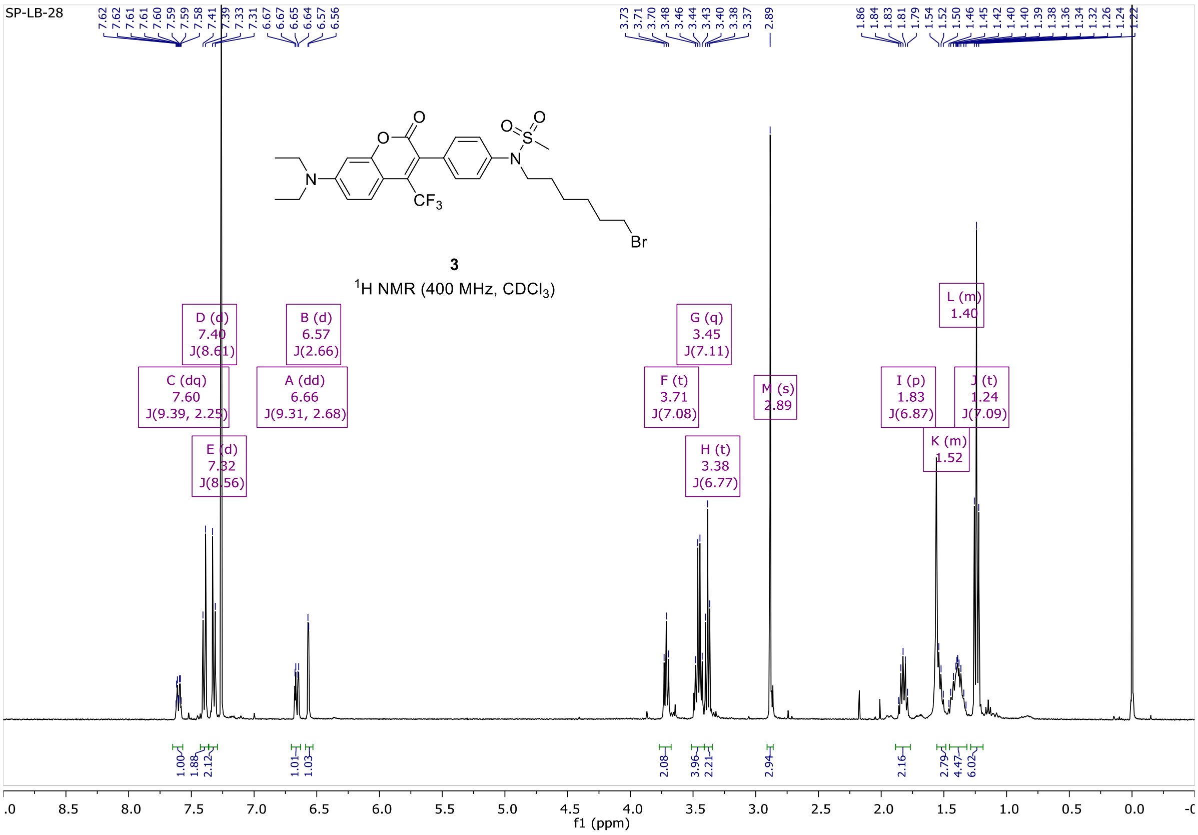

Figure S 53. ^{1}H NMR spectrum of compound **3**.

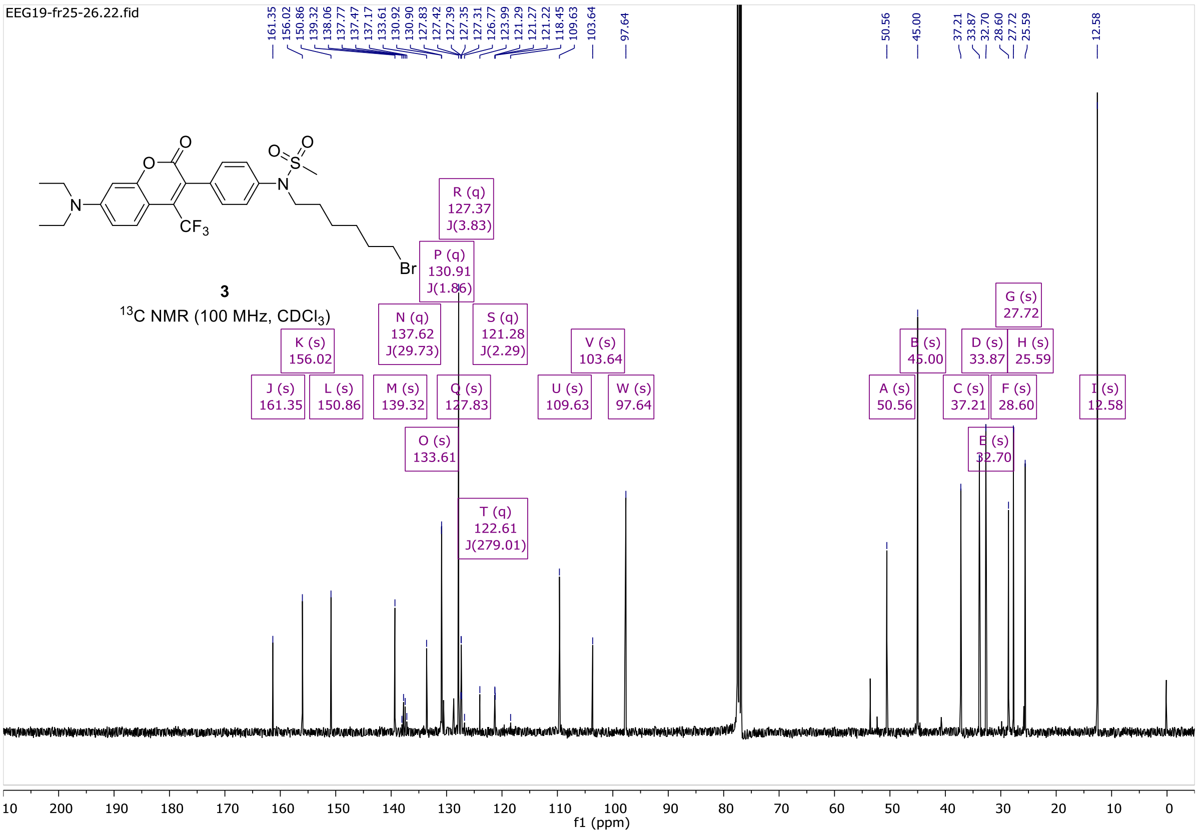

Figure S 54. ^{13}C NMR spectrum of compound **3**.

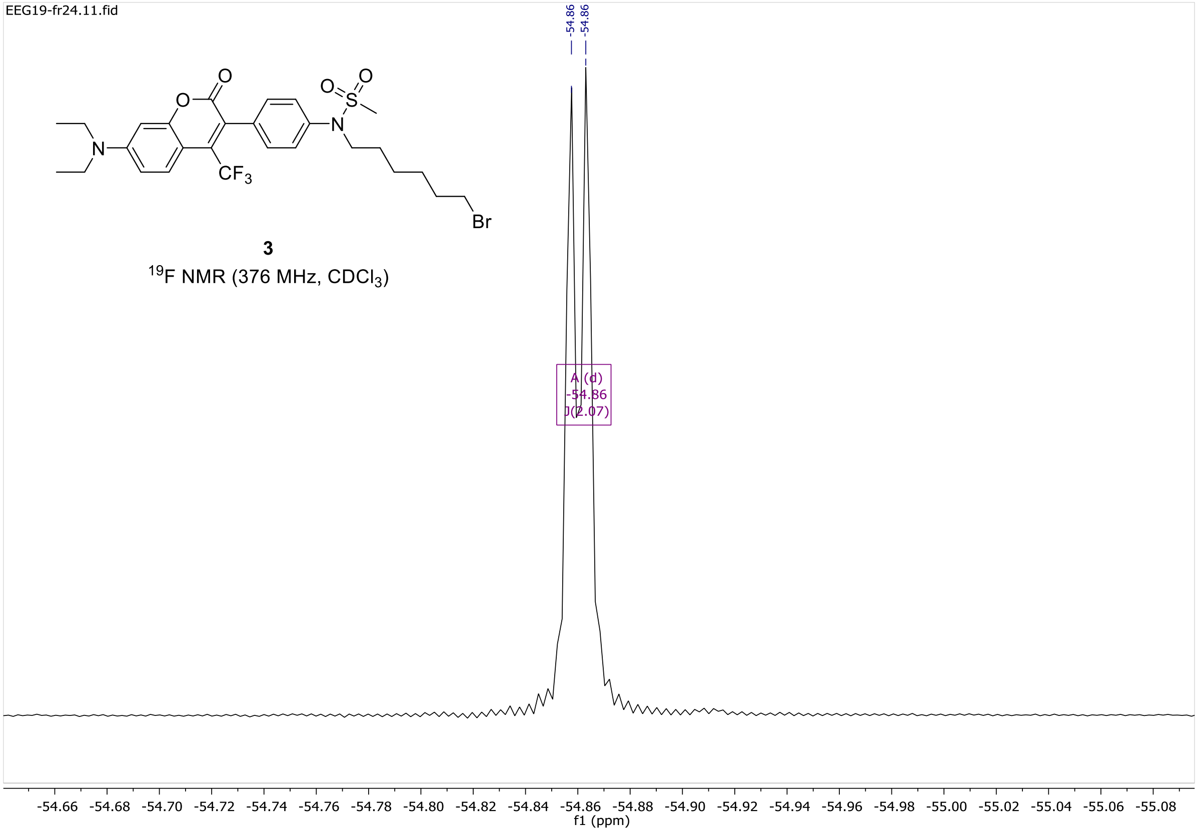

Figure S 55. ^{19}F NMR spectrum of compound **3**.

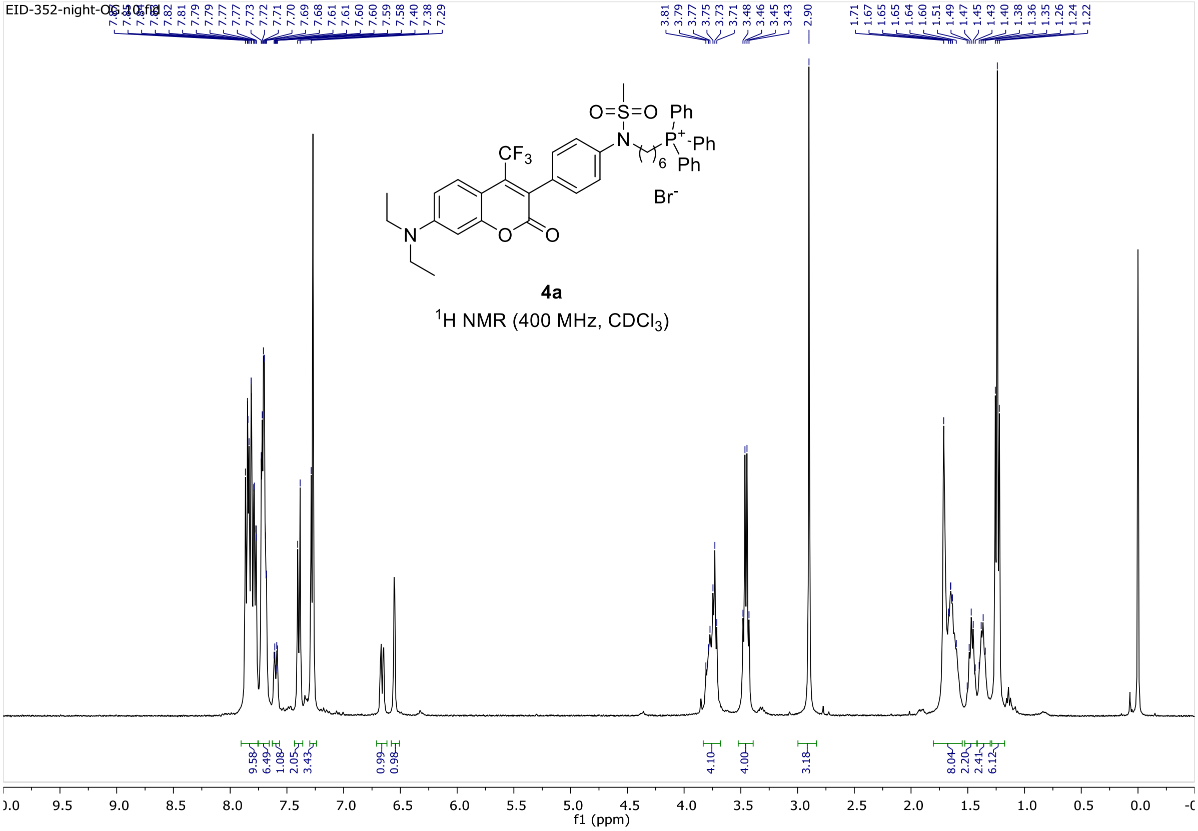

Figure S 56. ^{1}H NMR spectrum of compound **4a**.

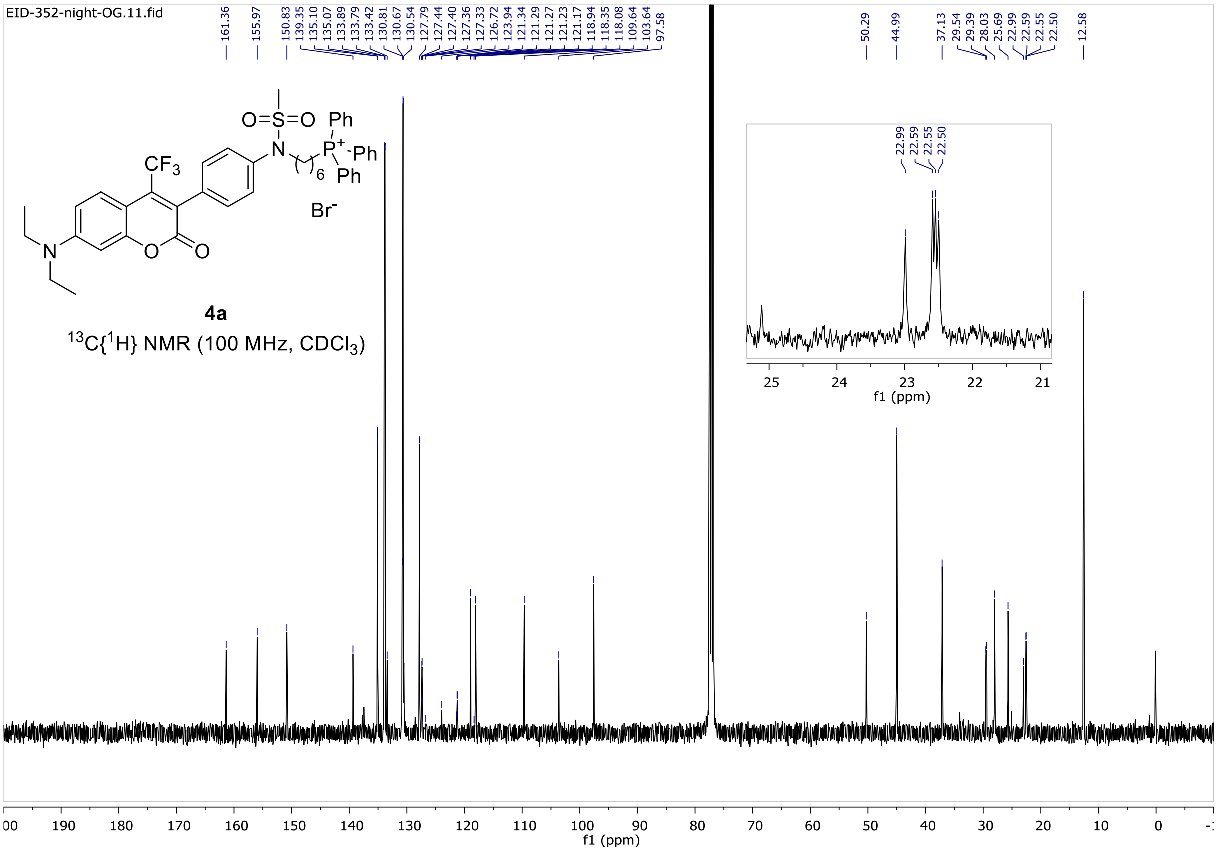

Figure S 57. ^{13}C NMR spectrum of compound **4a**.

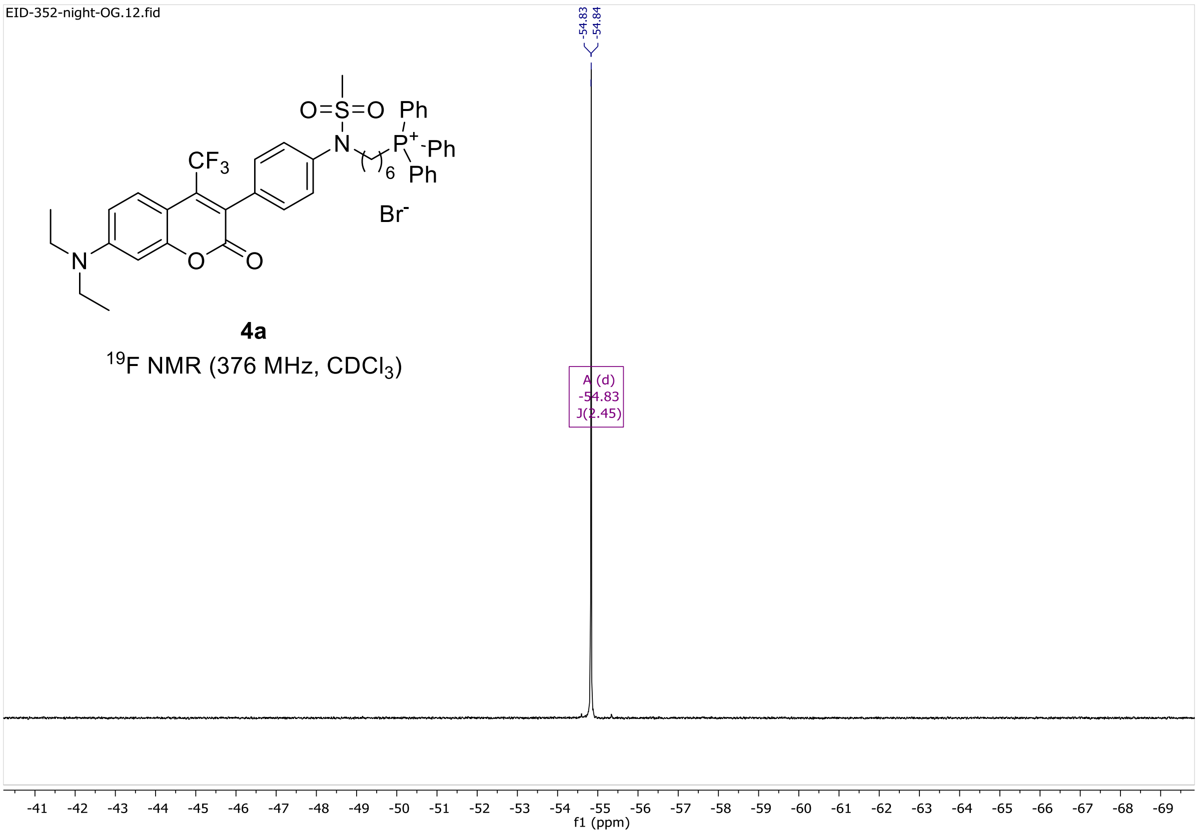

Figure S 58. ^{19}F NMR spectrum of compound **4a**.

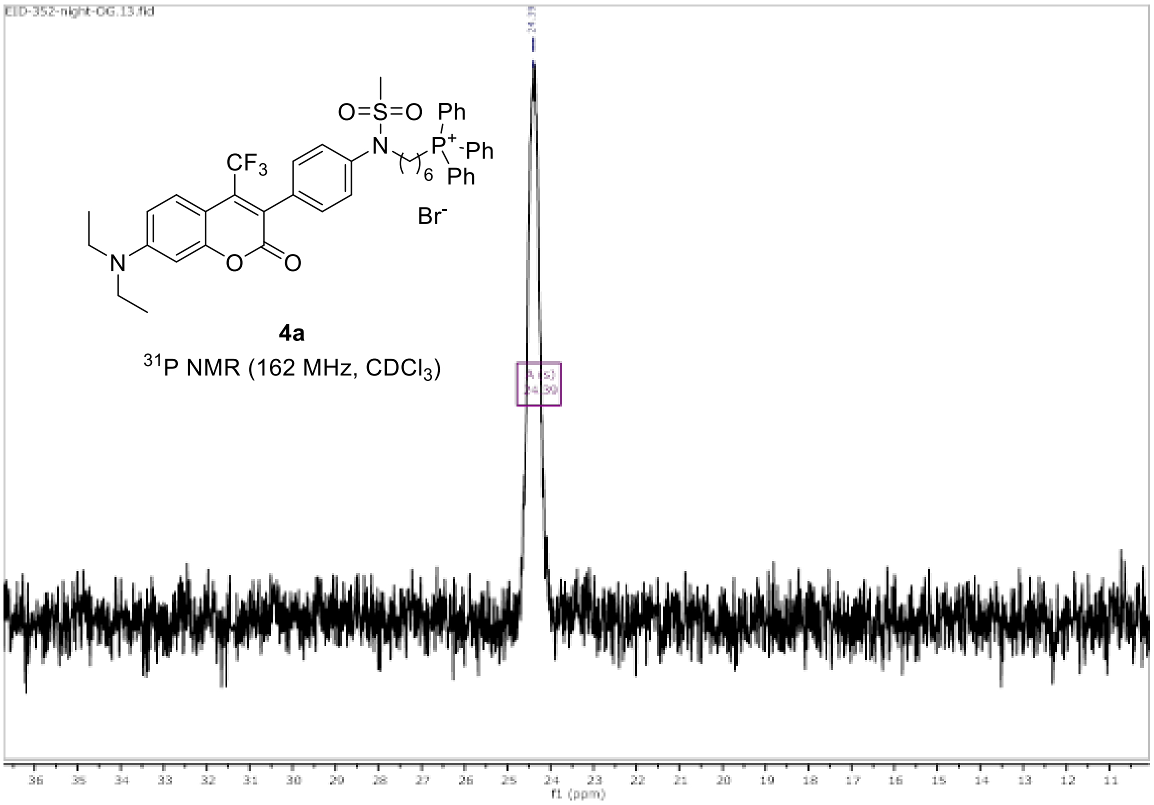

Figure S 59. ^{31}P NMR spectrum of compound **4a**.

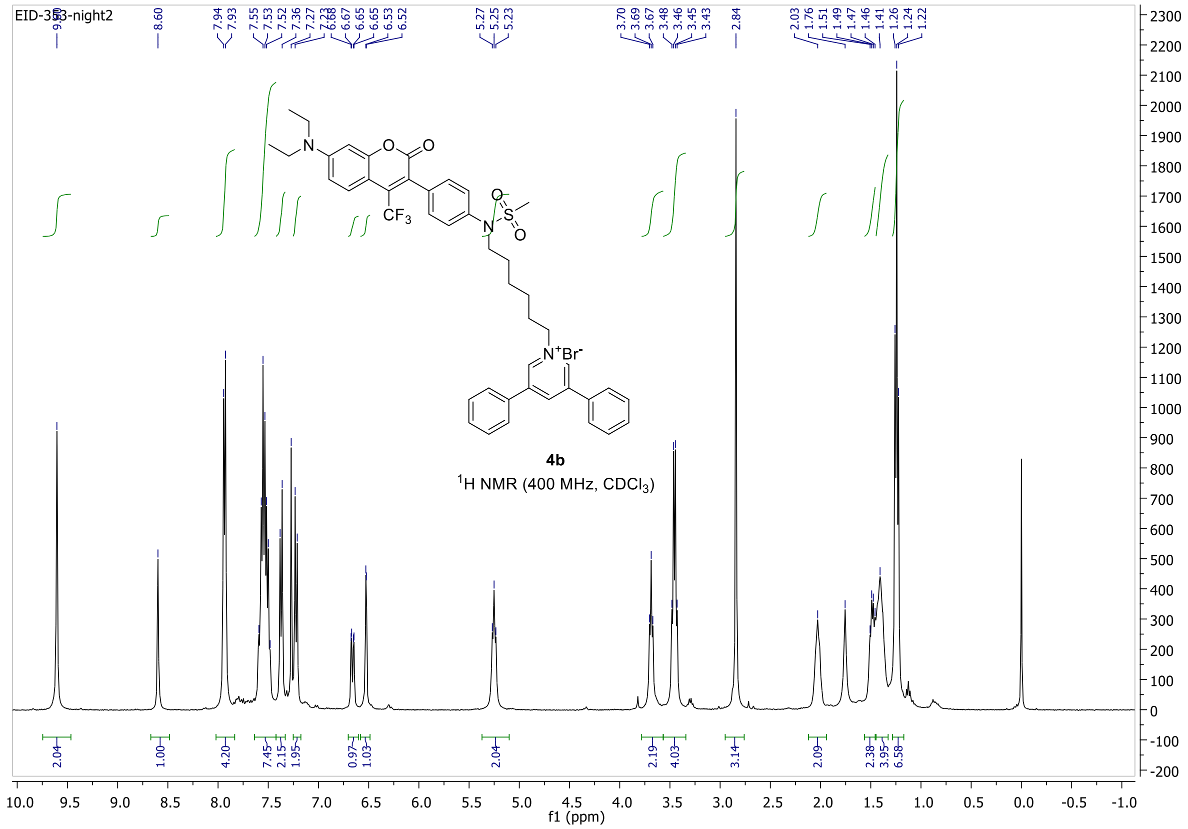

Figure S 60. ^{1}H NMR spectrum of compound **4b**.

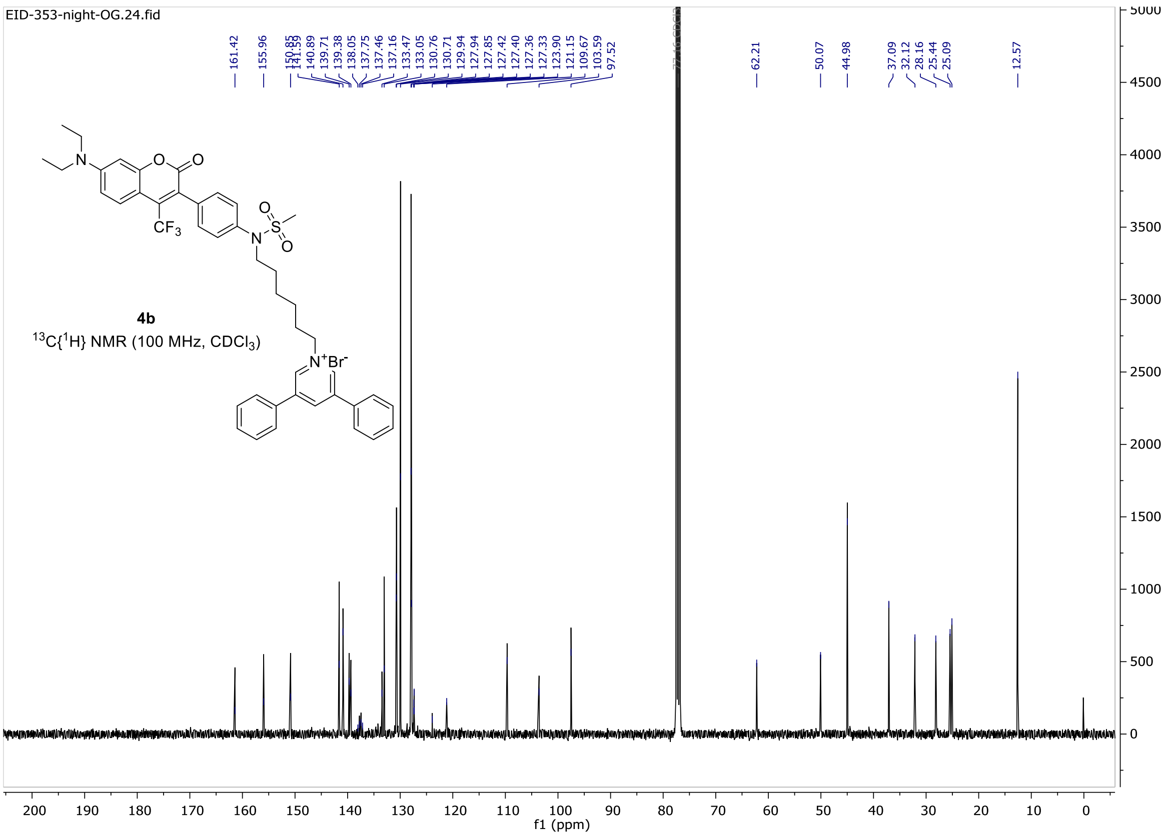

Figure S 61. ^{13}C NMR spectrum of compound **4b**.

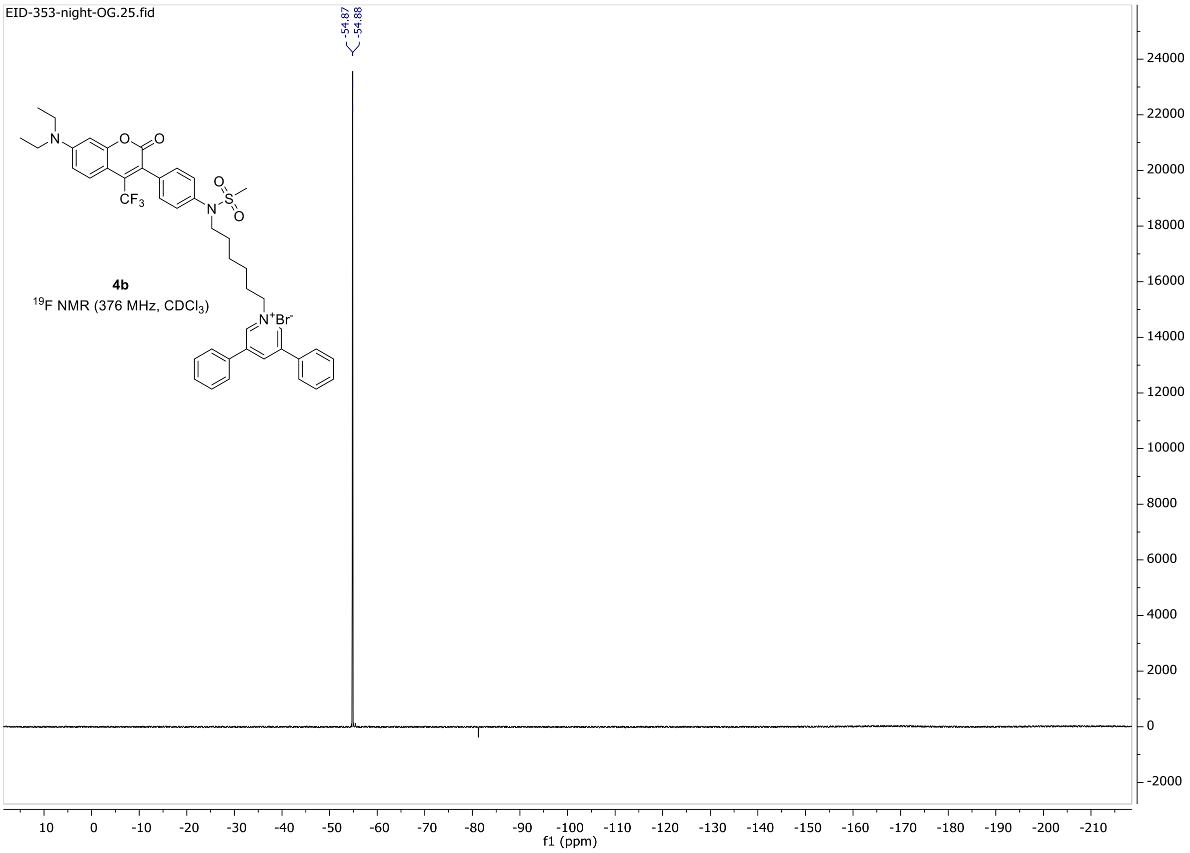

Figure S 62. ^{19}F NMR spectrum of compound **4b**.

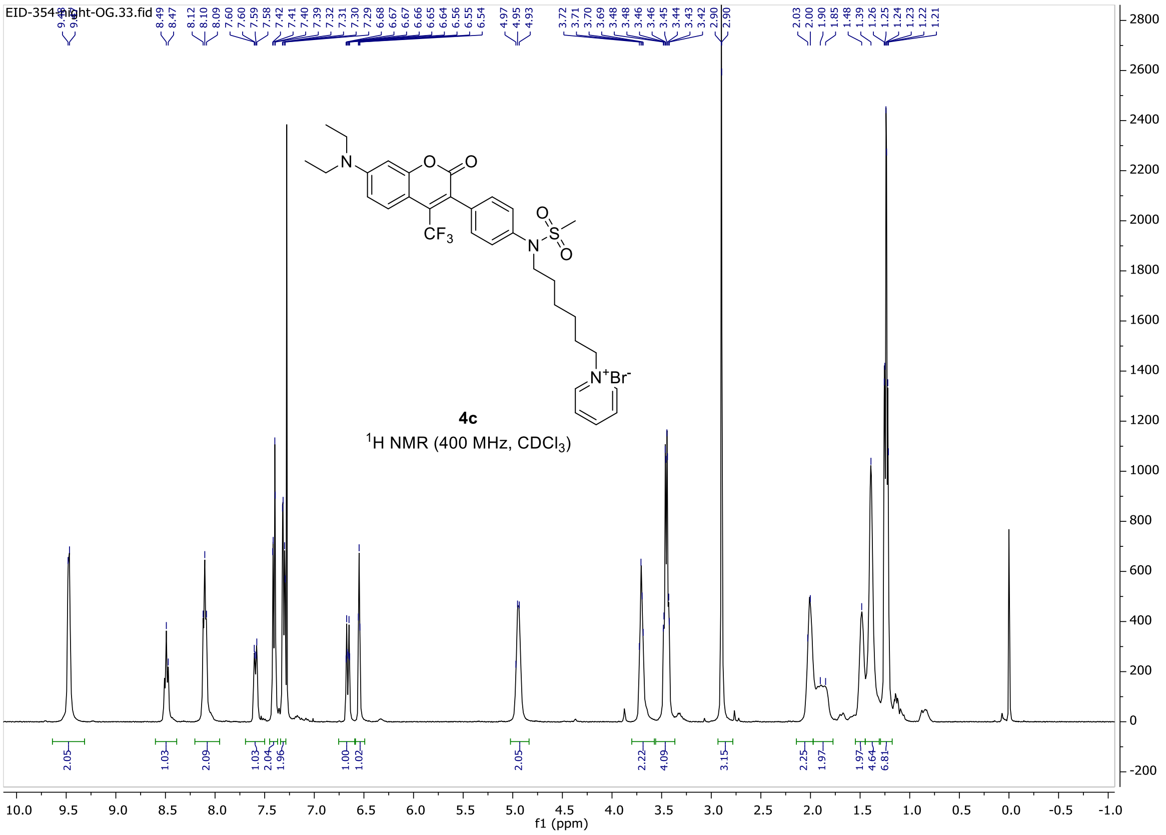

Figure S 63. ^{1}H NMR spectrum of compound **4c**.

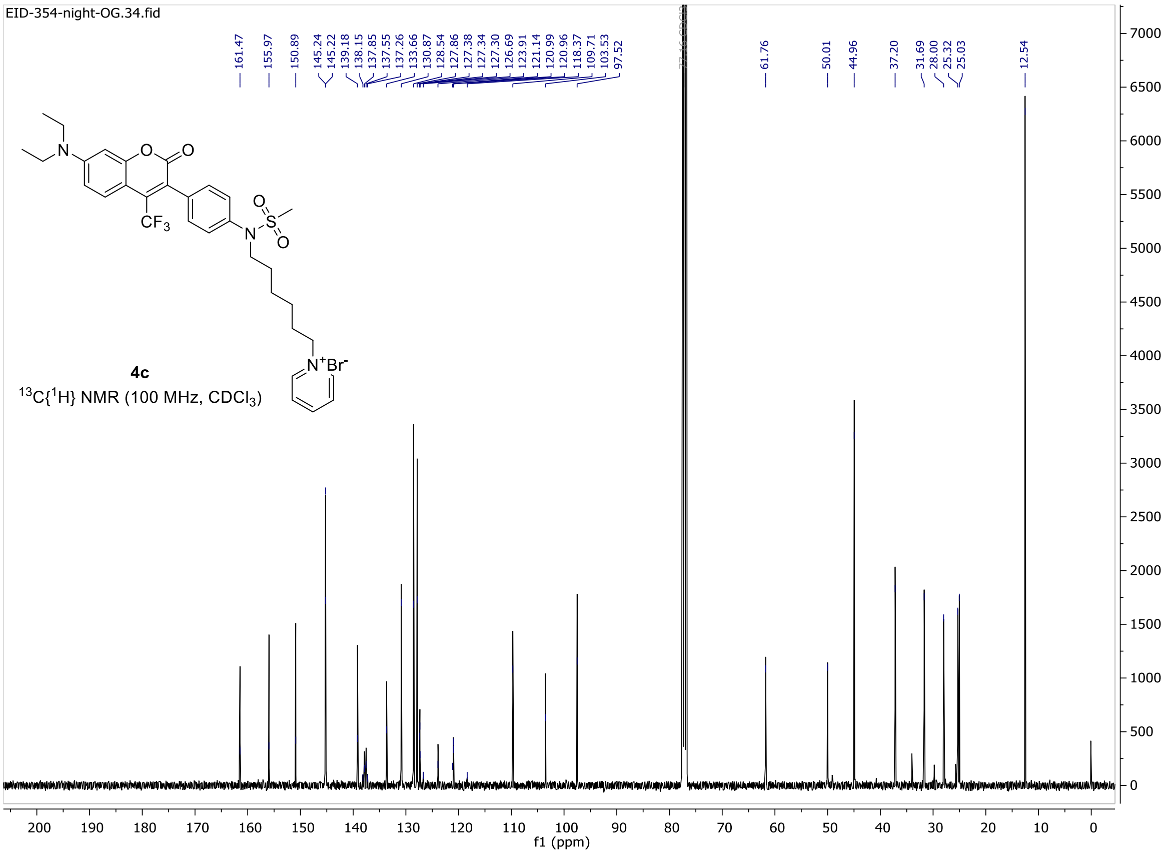

Figure S 64. ^{13}C NMR spectrum of compound **4c**.

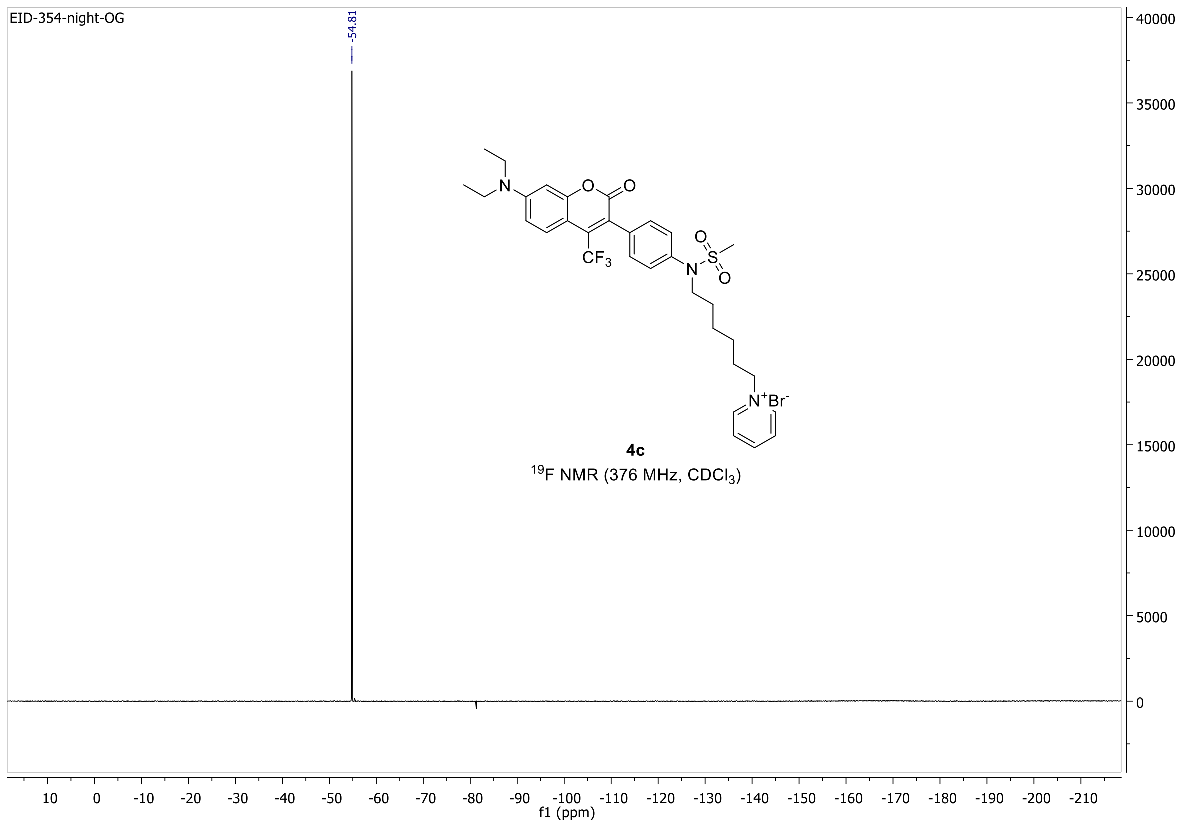

Figure S 65. ^{19}F NMR spectrum of compound **4c**.

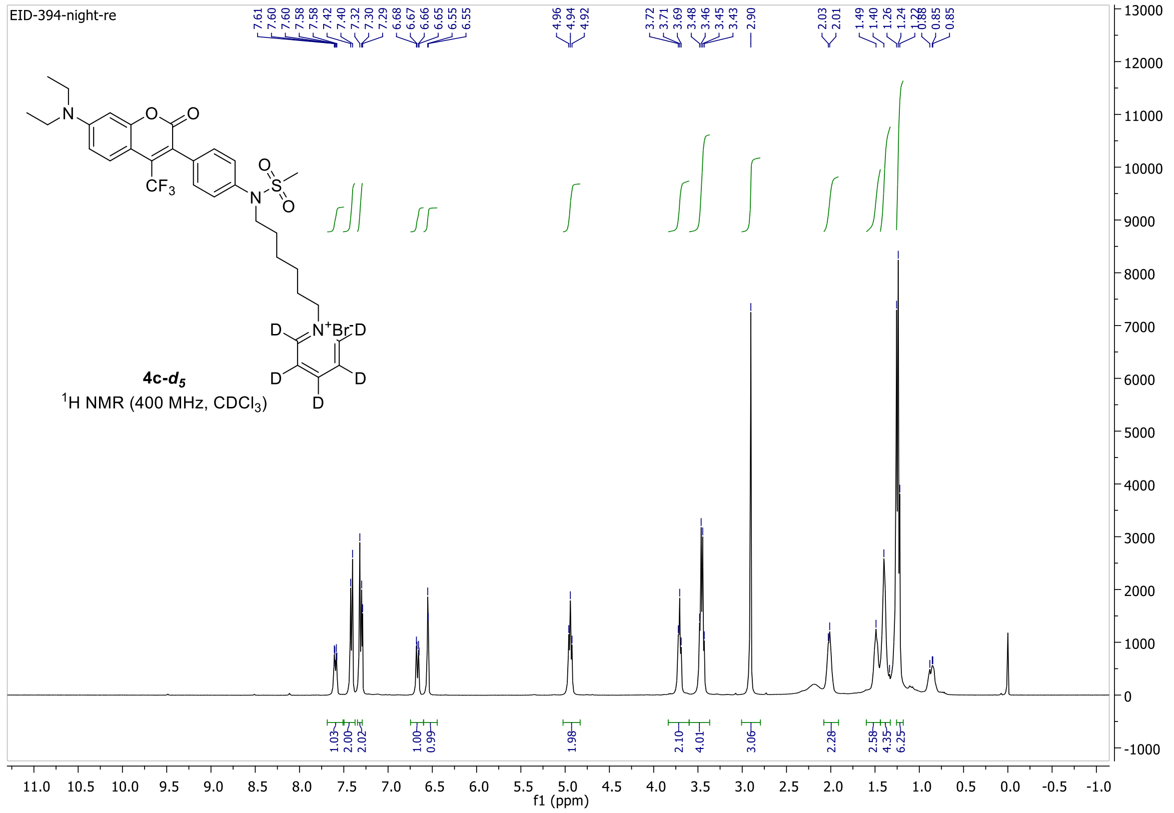

Figure S 66. ^{1}H NMR spectrum of compound **4c-*d*$_5$**.

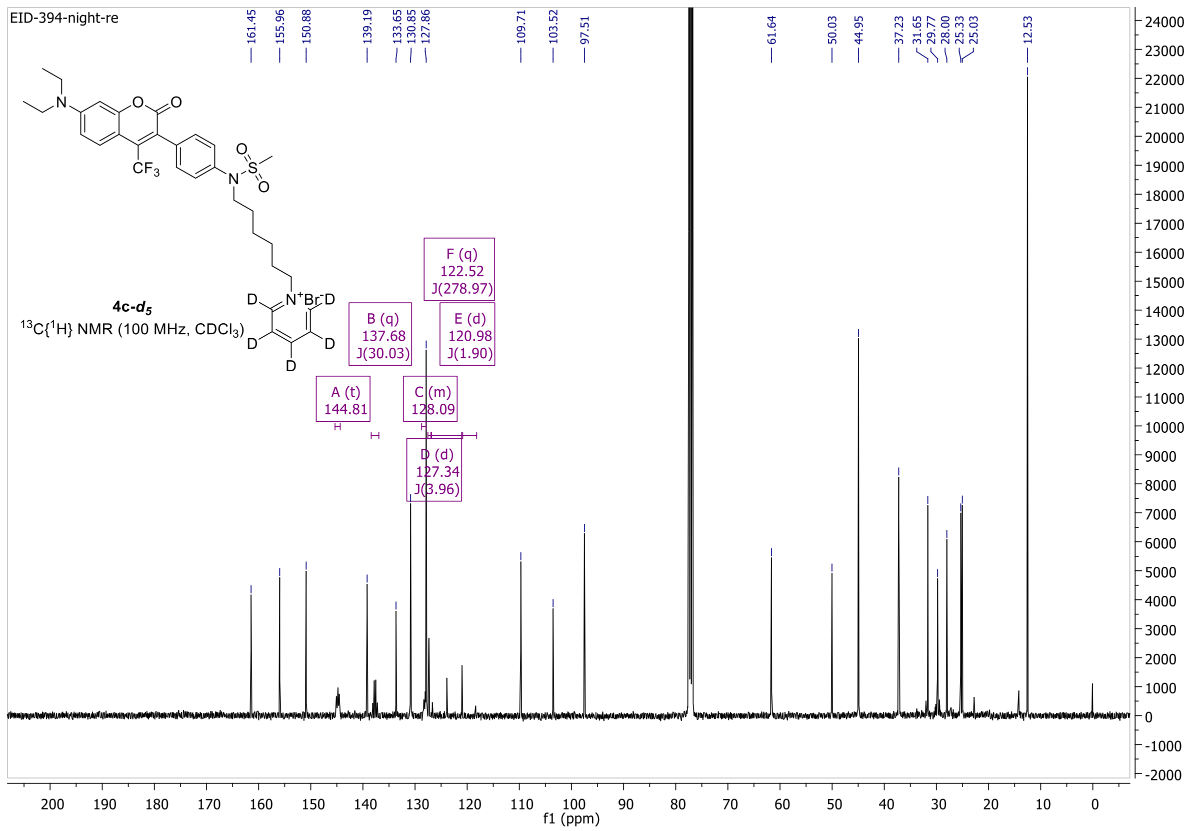

Figure S 67. ^{13}C NMR spectrum of compound **4c-*d*$_5$**.

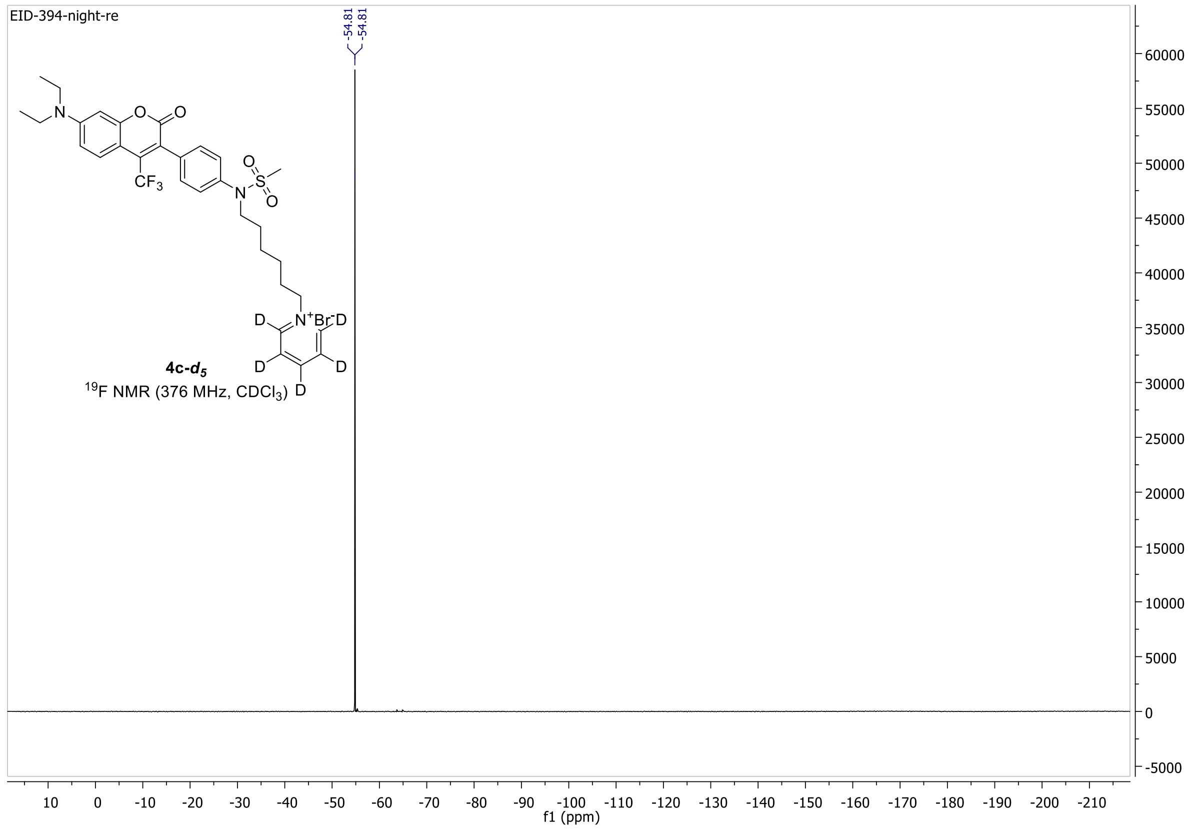

Figure S 68. ^{19}F NMR spectrum of compound **4c-*d*$_5$**.

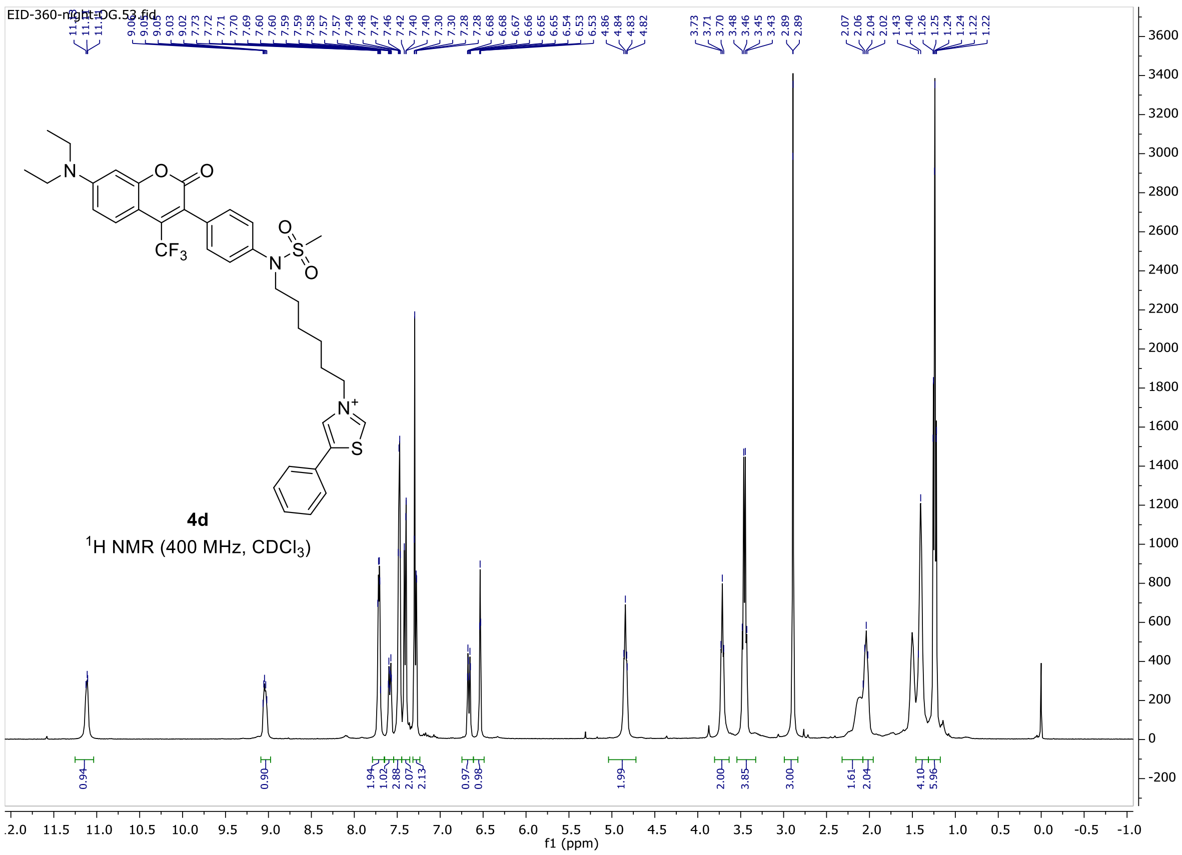

Figure S 69. ^{1}H NMR spectrum of compound **4d**.

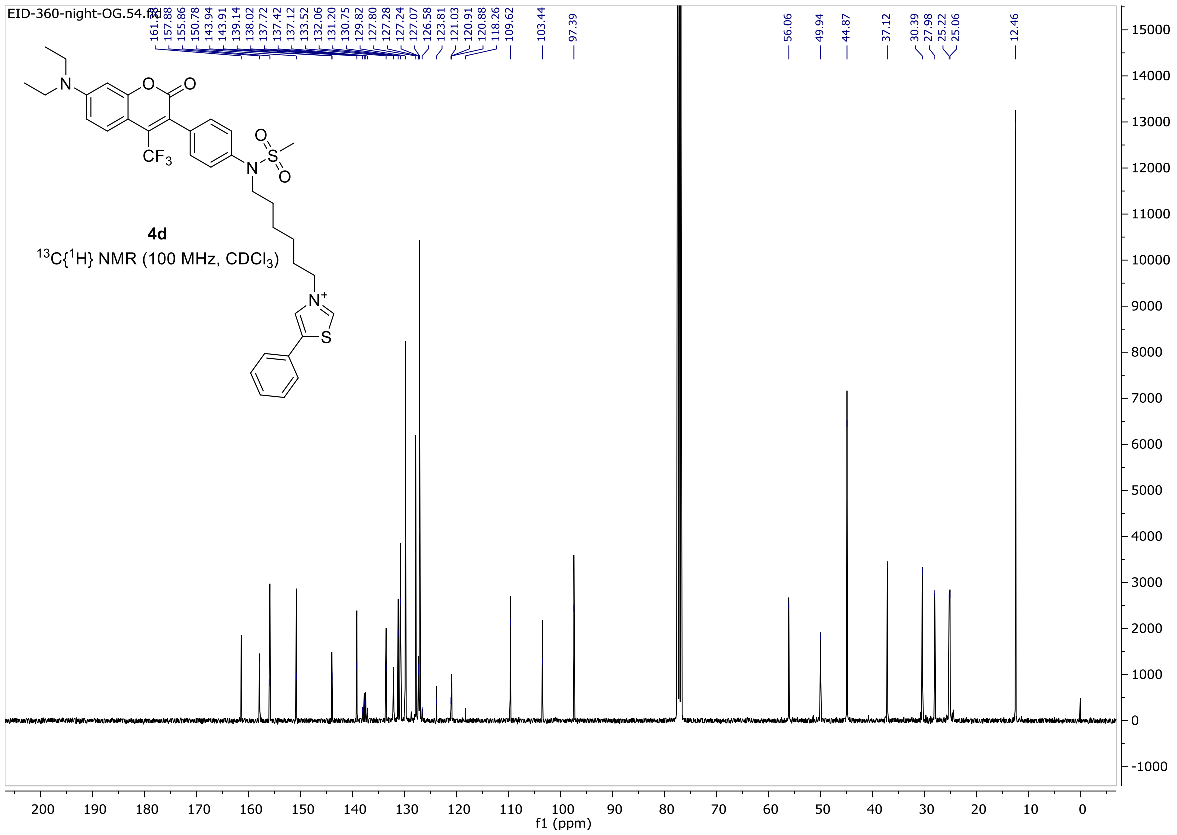

Figure S 70. ^{13}C NMR spectrum of compound **4d**.

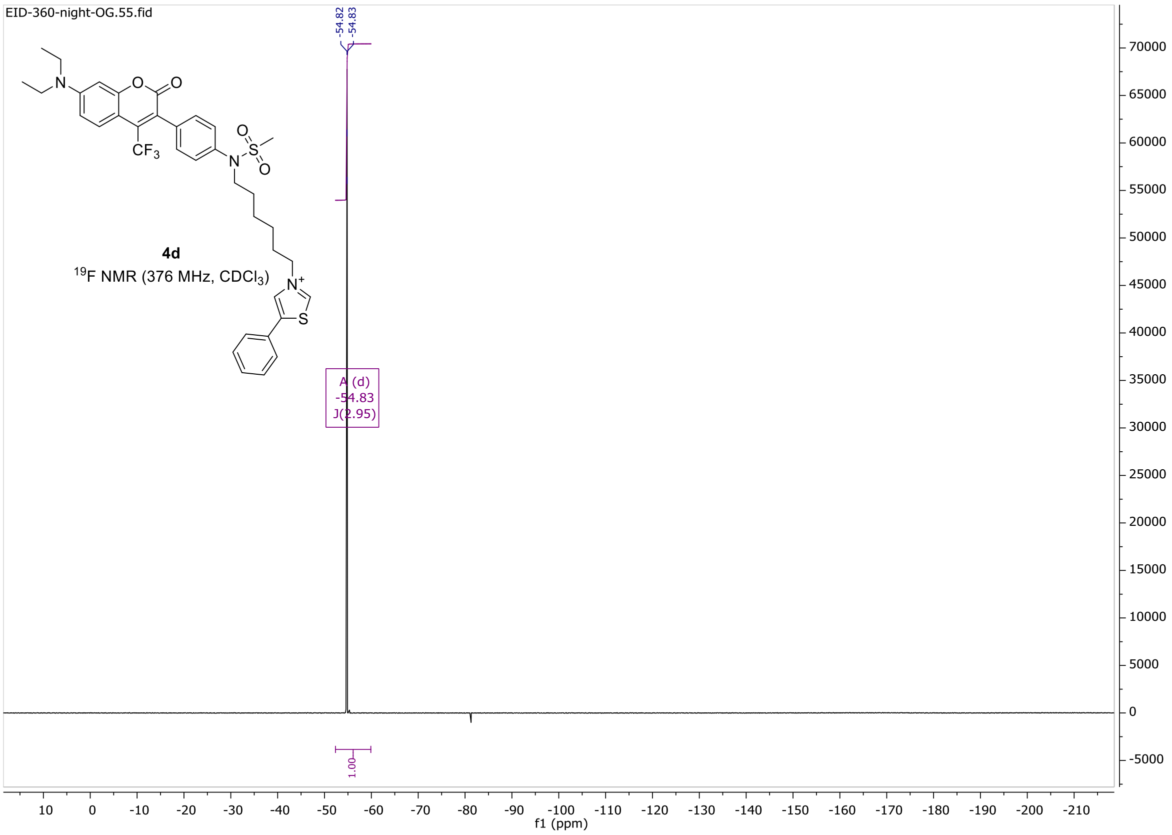

Figure S 71. ^{19}F NMR spectrum of compound **4d**.

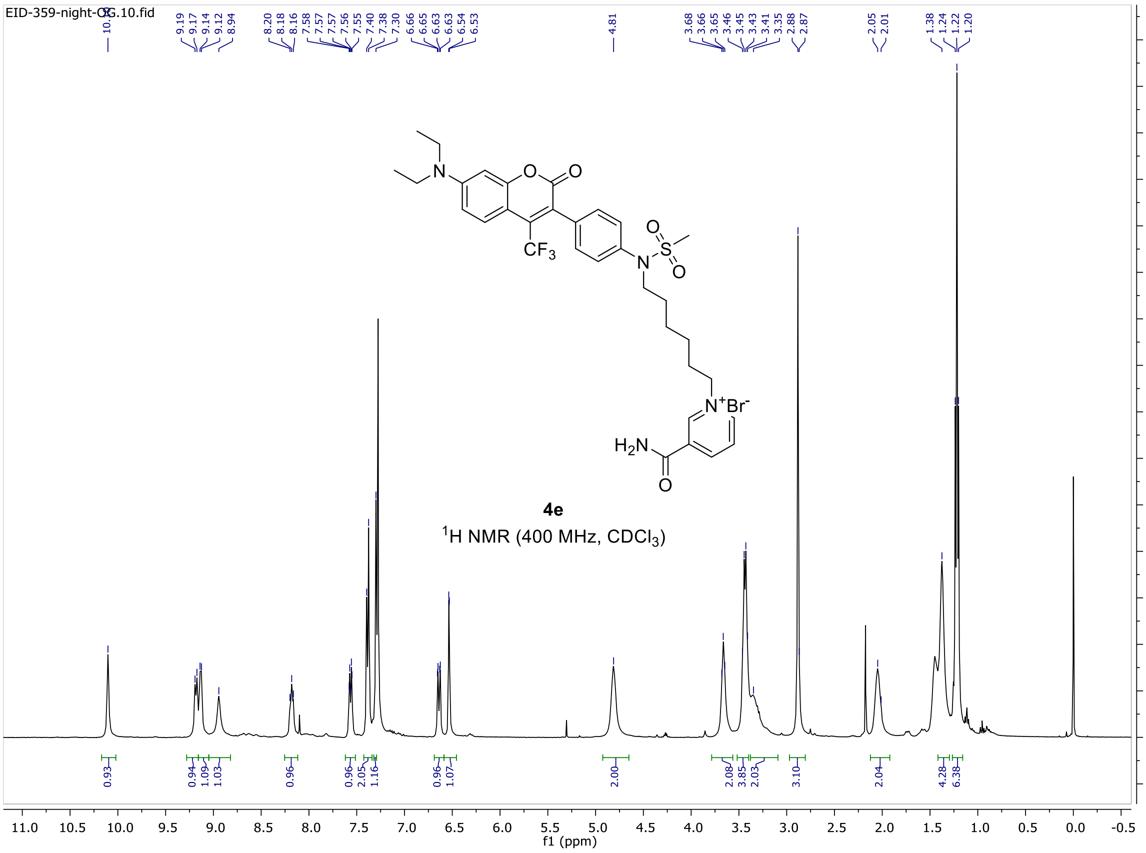

Figure S 72. ^{1}H NMR spectrum of compound **4e**.

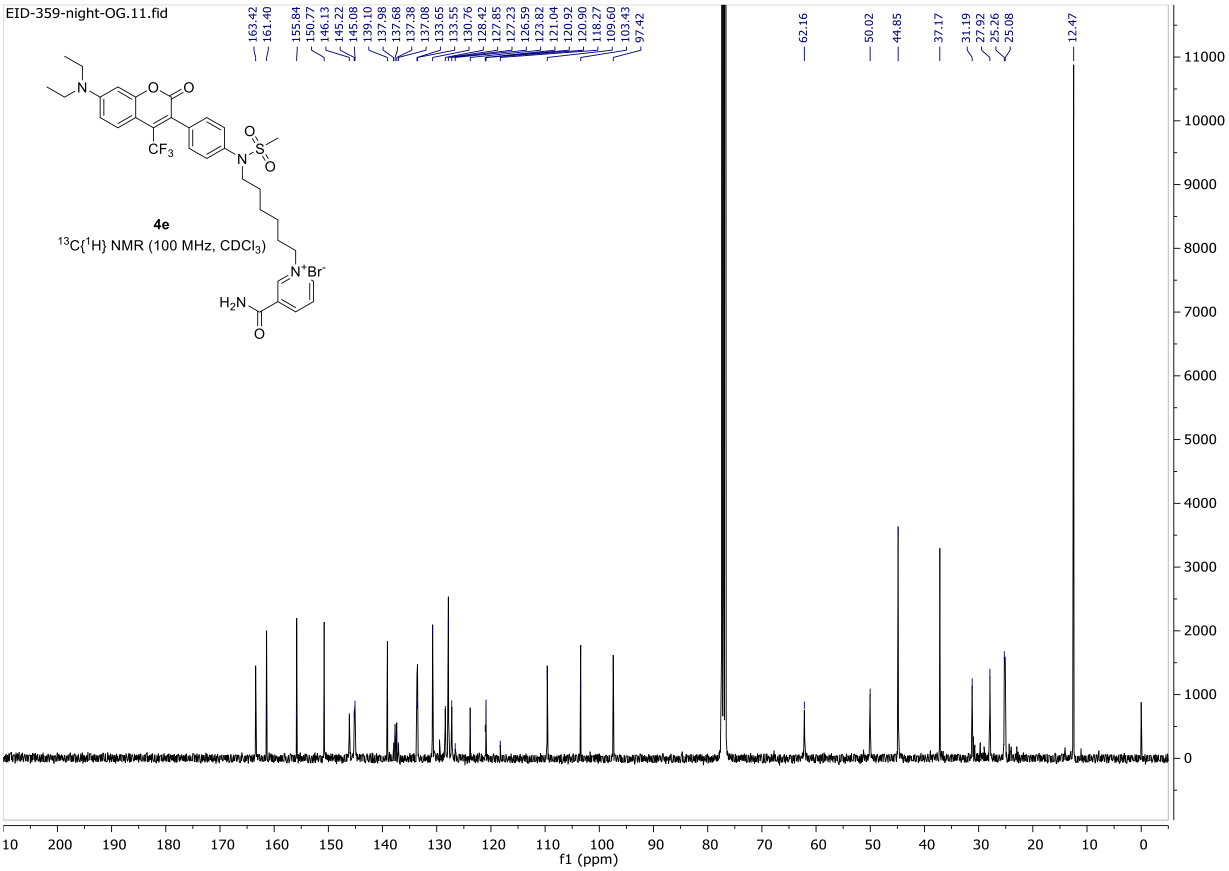

Figure S 73. ^{13}C NMR spectrum of compound **4e**.

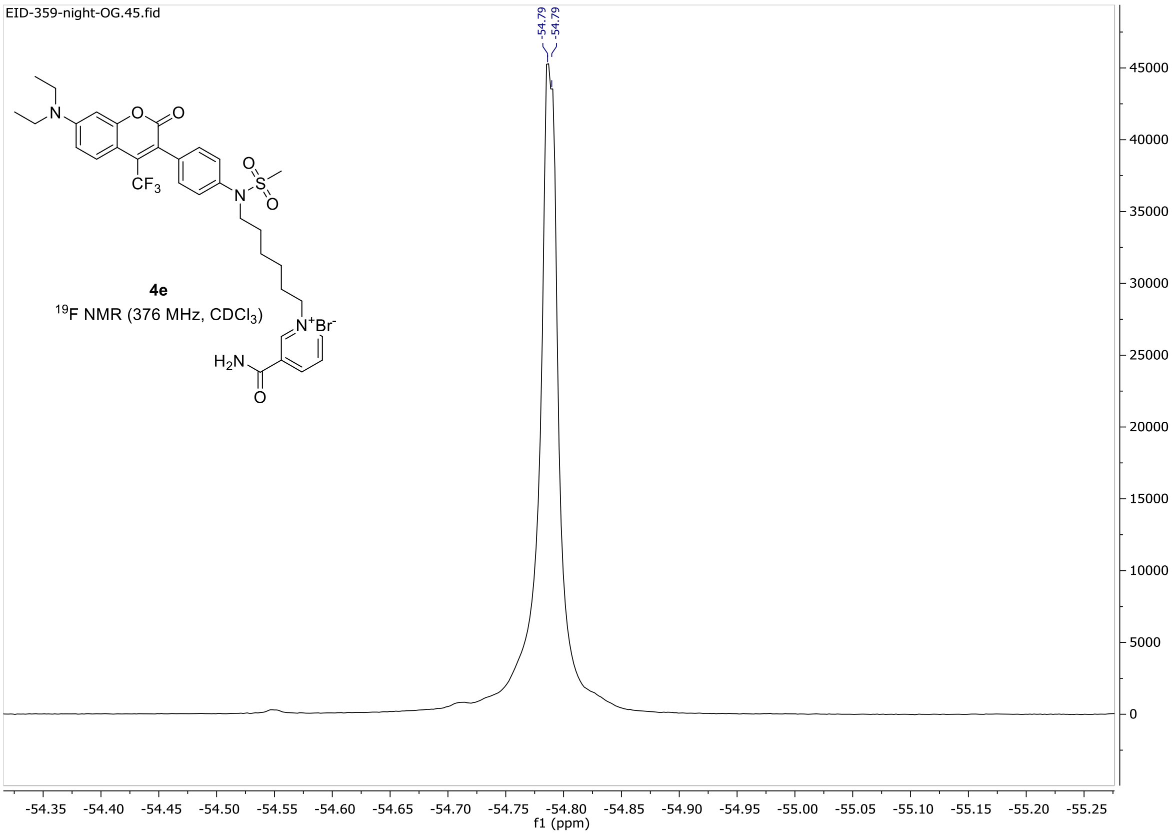

Figure S 74. ^{19}F NMR spectrum of compound **4e**.

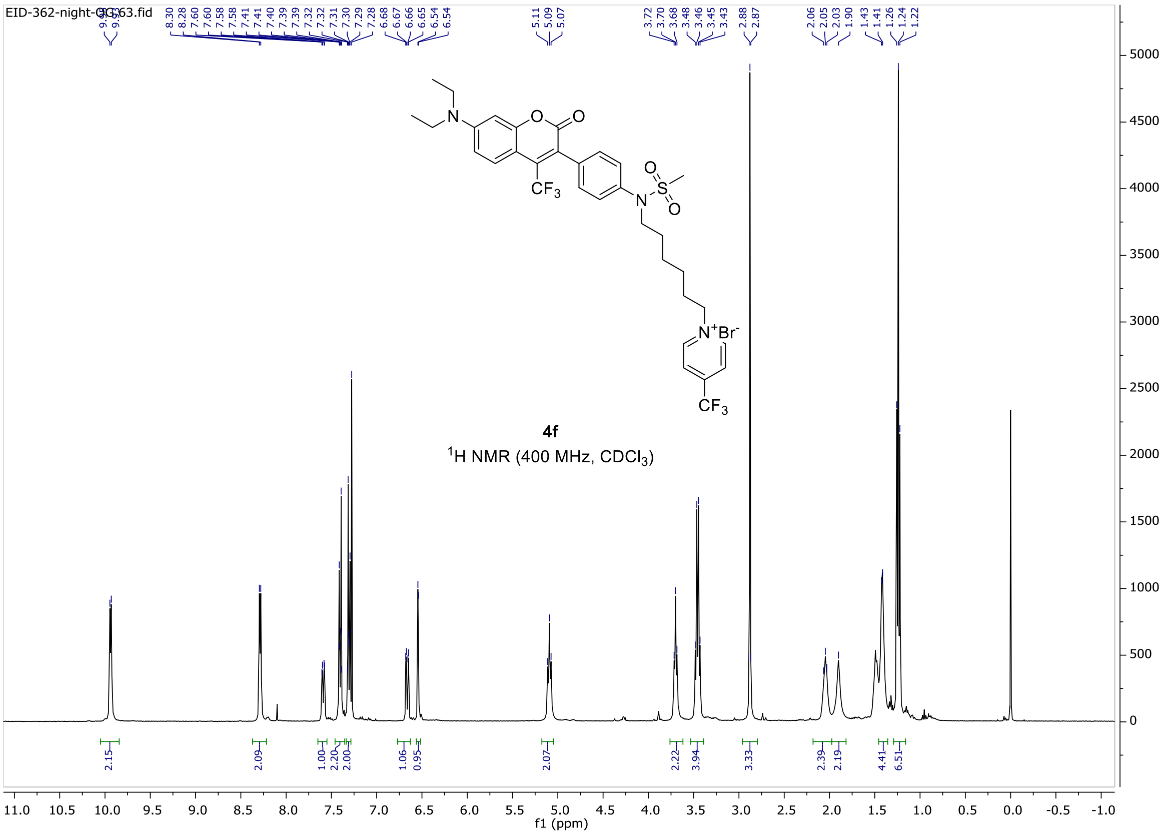

Figure S 75. ^{1}H NMR spectrum of compound **4f**.

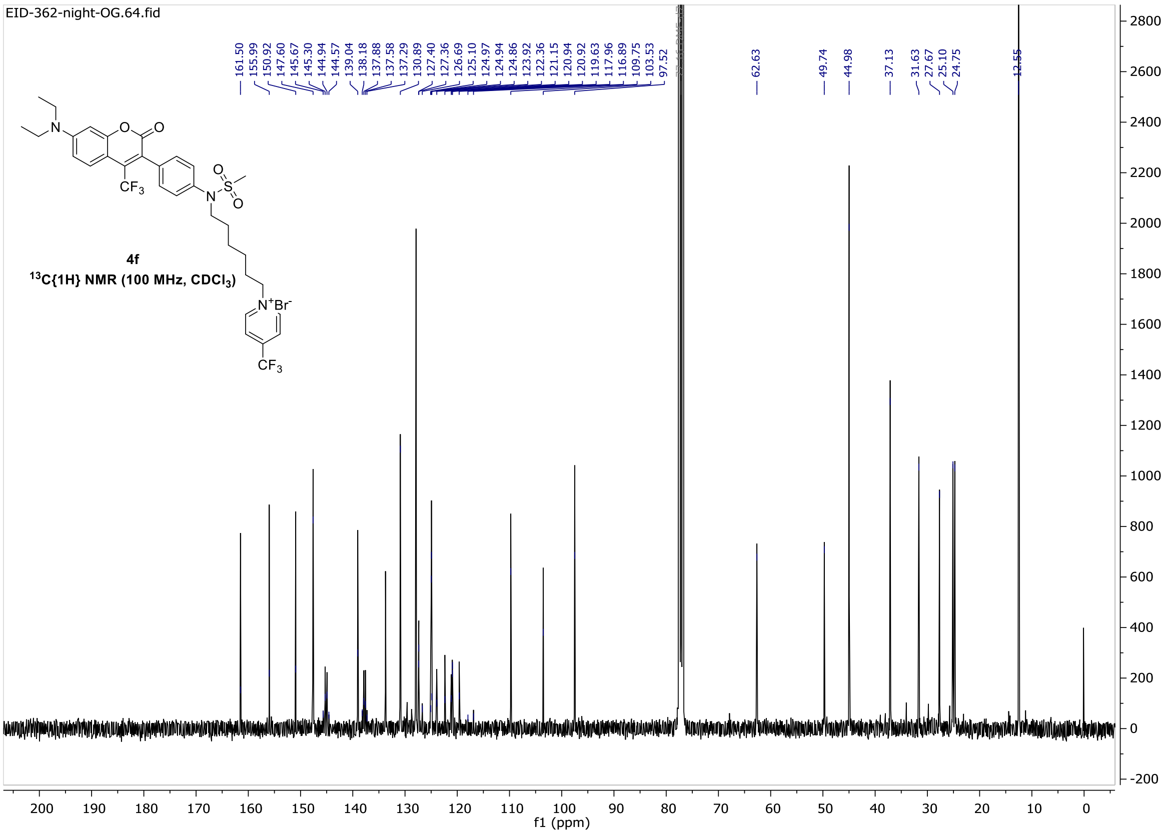

Figure S 76. ^{13}C NMR spectrum of compound **4f**.

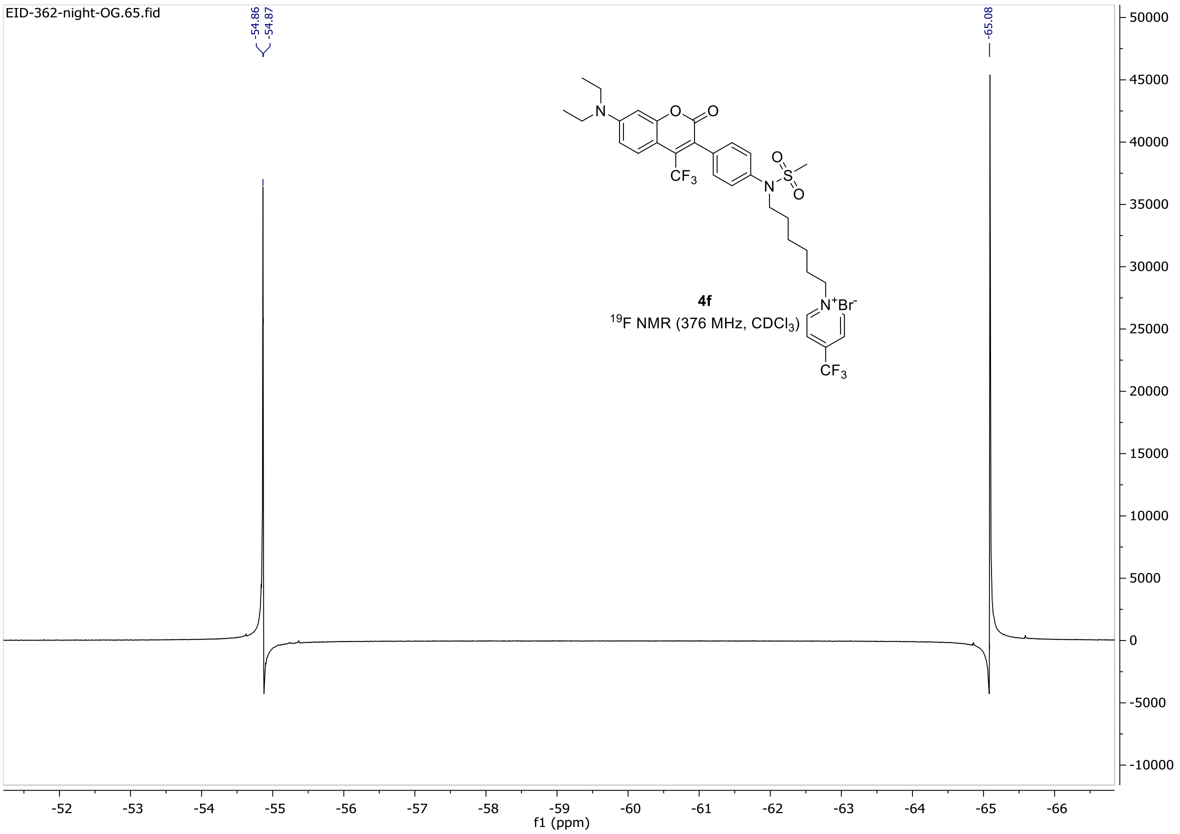

Figure S 77. ^{19}F NMR spectrum of compound **4f**.

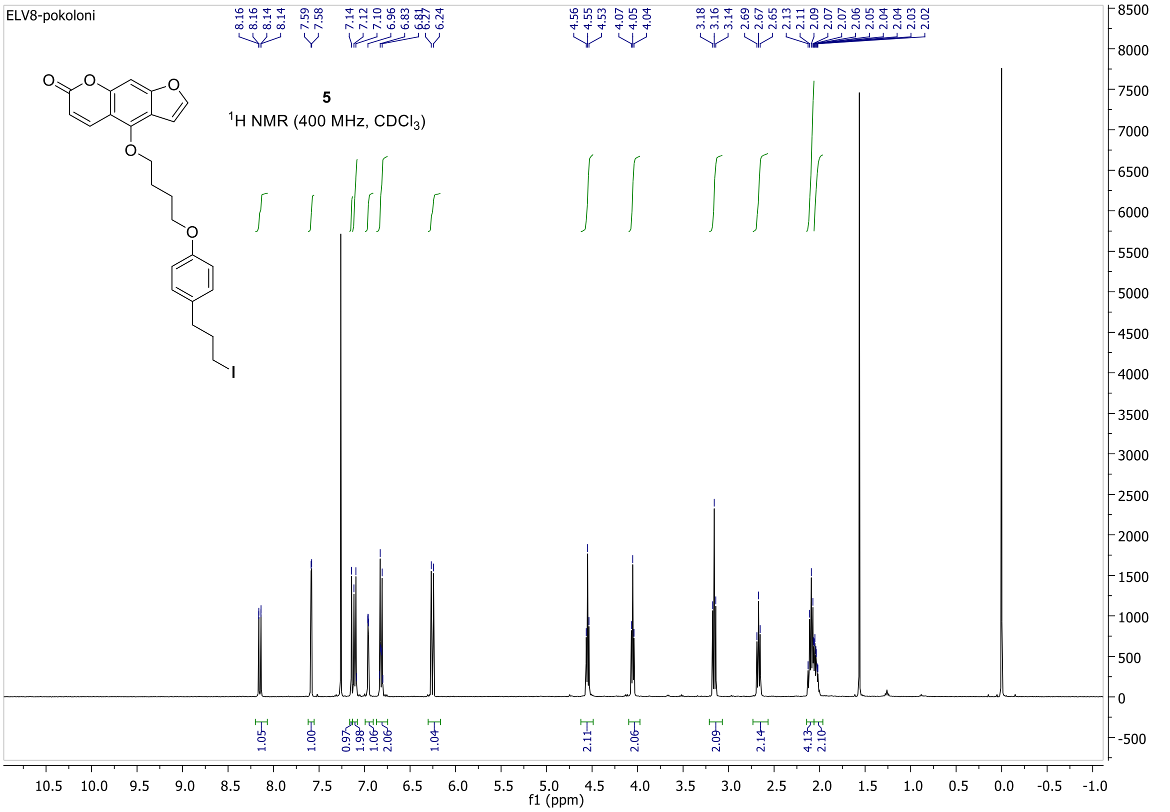

Figure S 78. ^{1}H NMR spectrum of compound **5**.

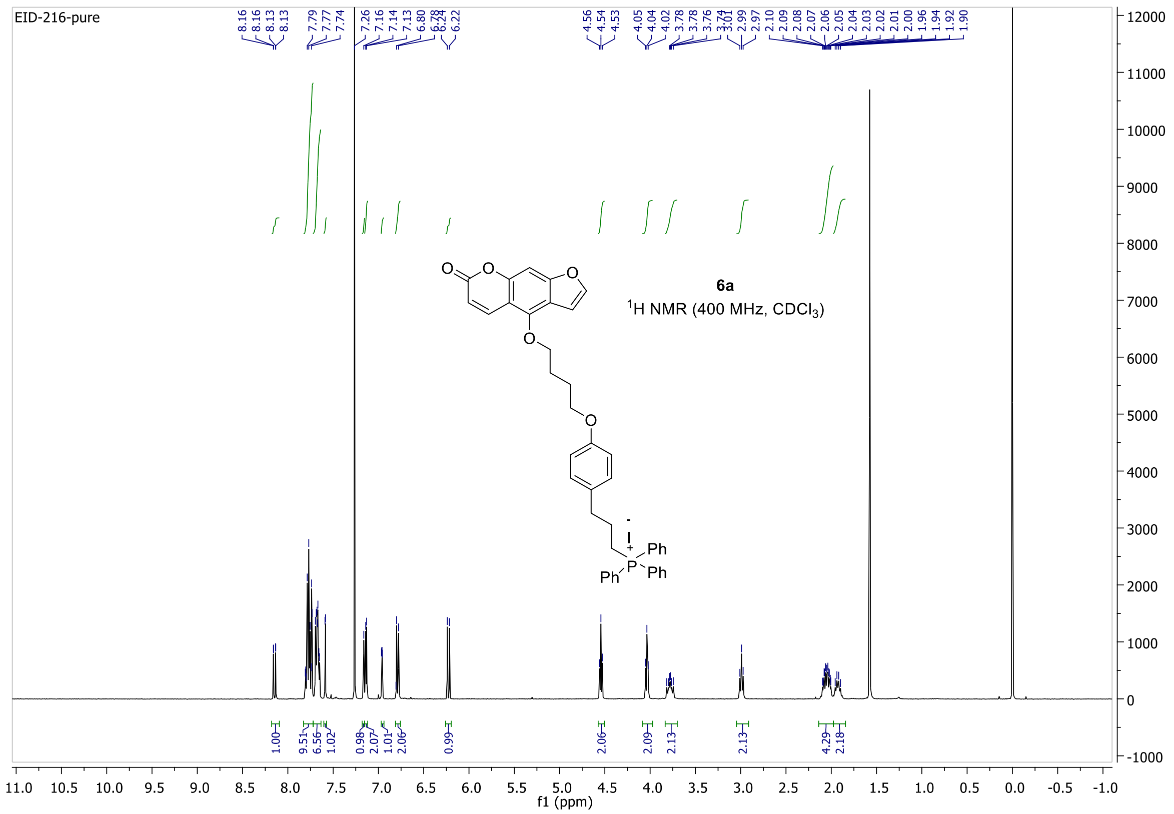

Figure S 79.^{1}H NMR spectrum of compound **6a**.

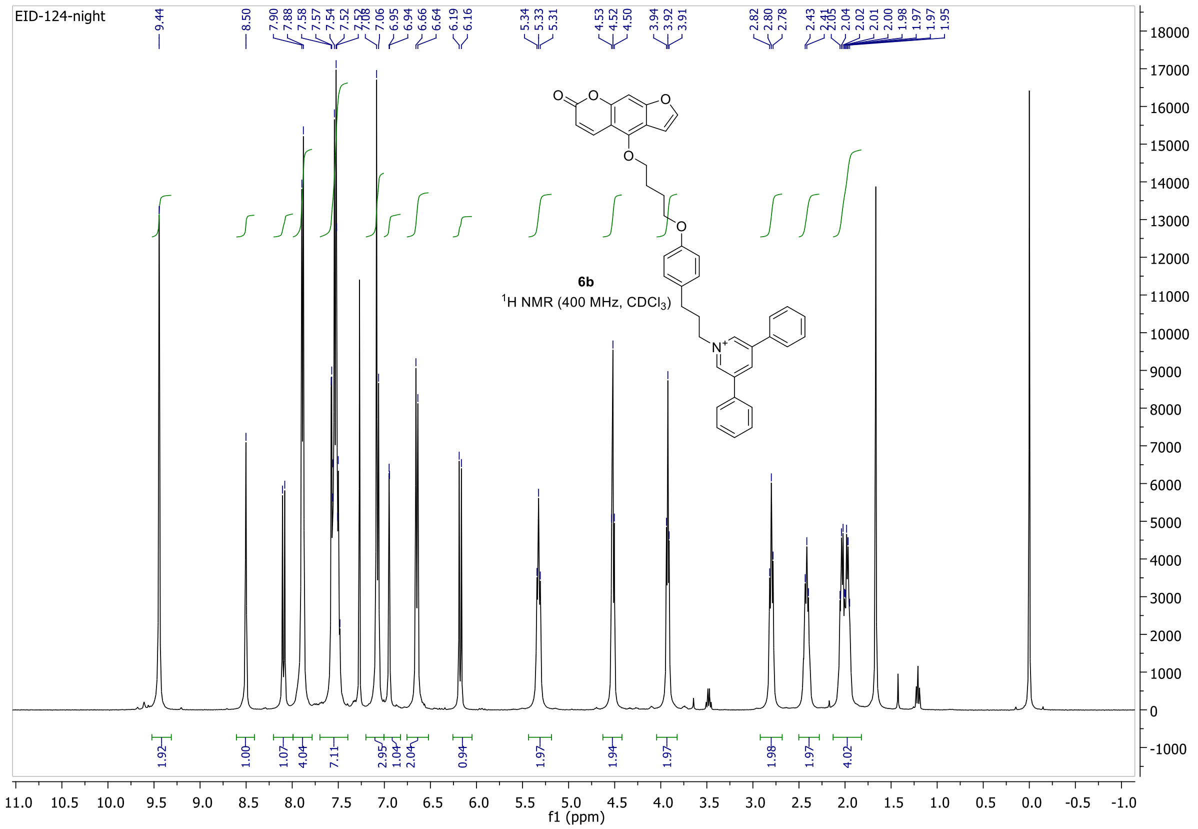

Figure S 80. ^{1}H NMR spectrum of compound **6b**.

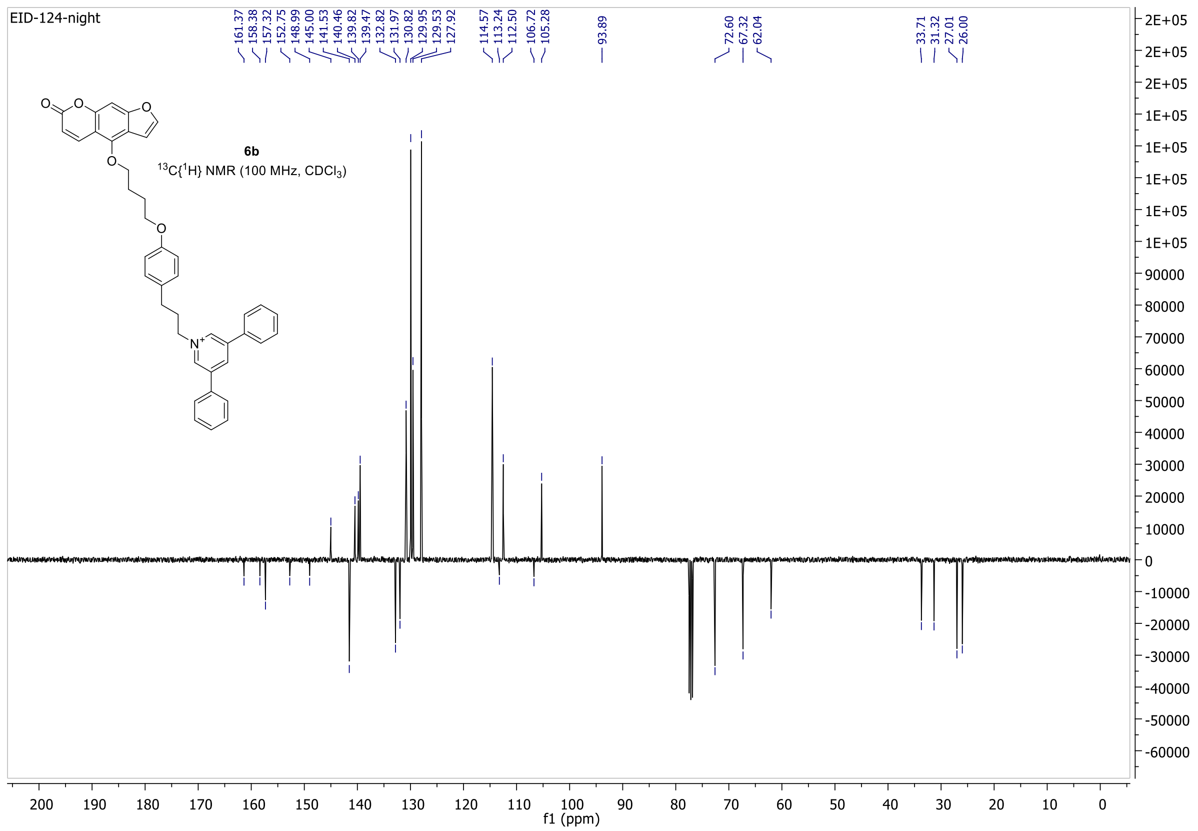

Figure S 81. ^{13}C Attached Proton Test (APT) NMR spectrum of compound **6b**.

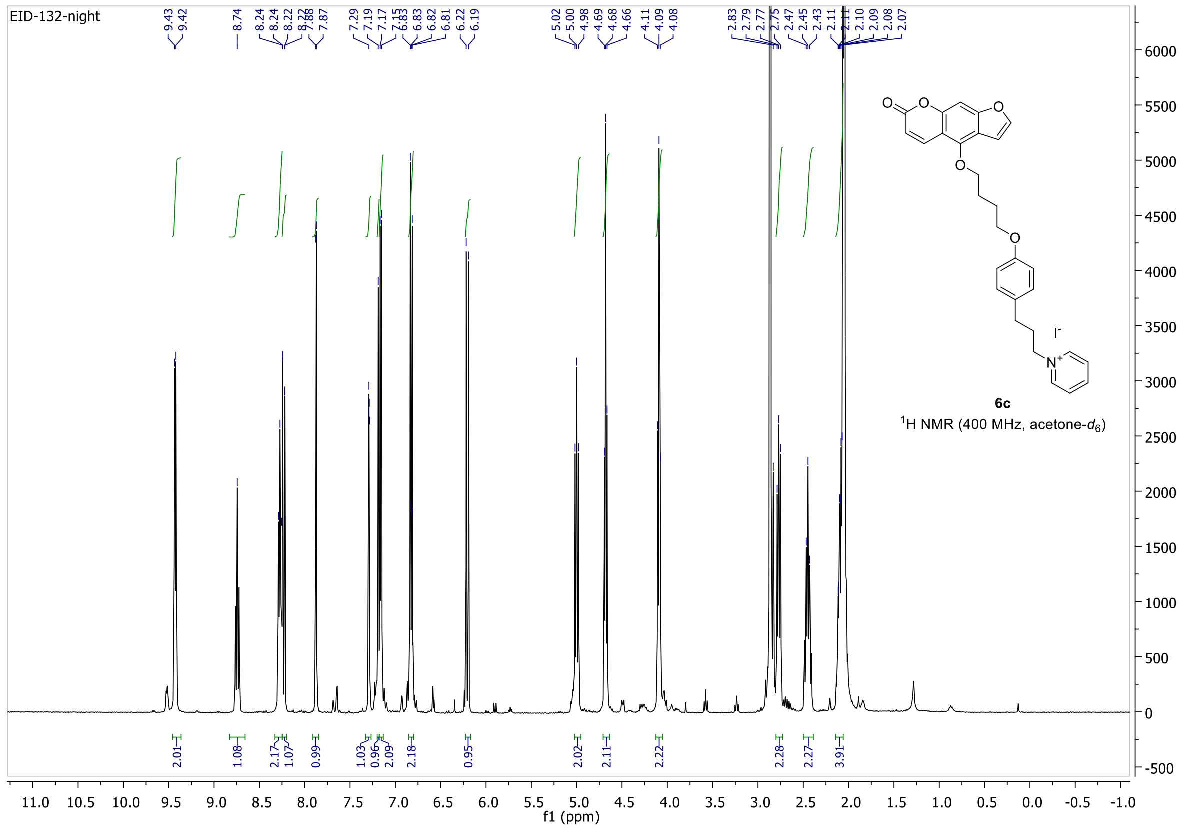

Figure S 82. ^{1}H NMR spectrum of compound **6c**.

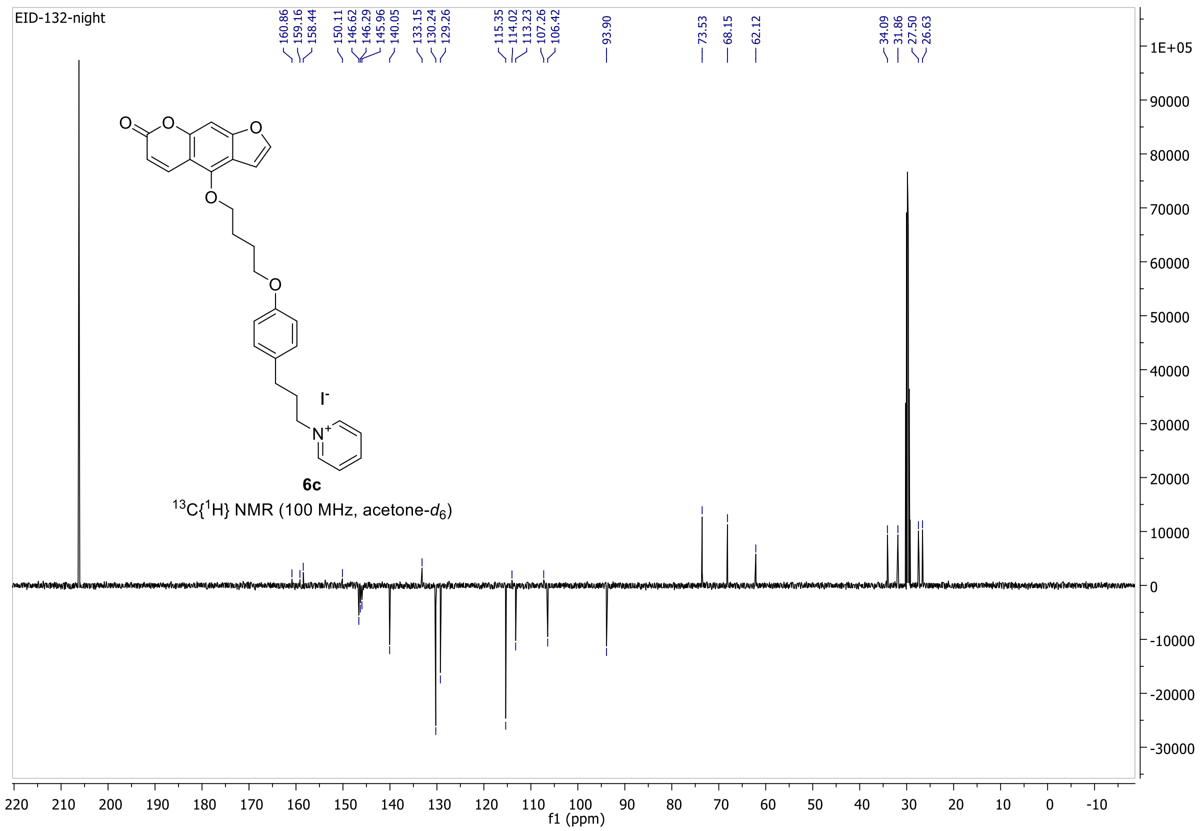

Figure S 83. ^{13}C Attached Proton Test (APT) NMR spectrum of compound **6c**.

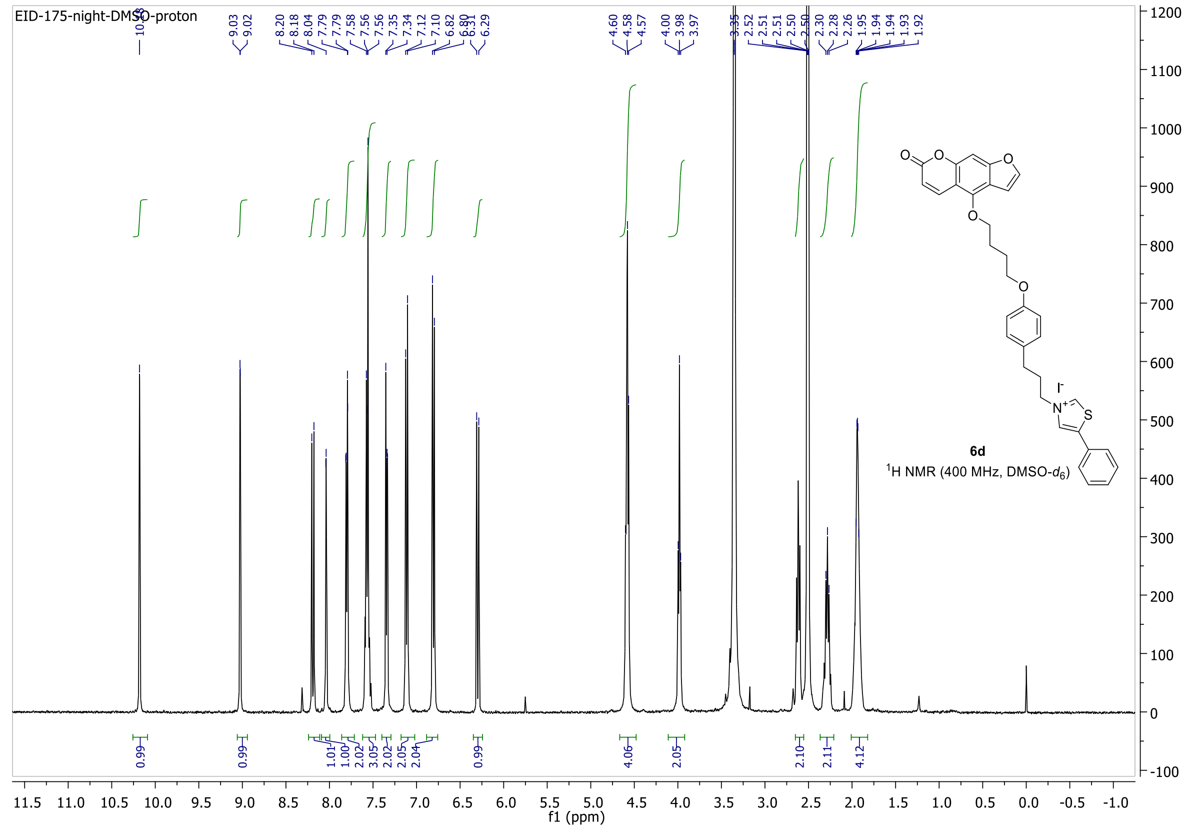

Figure S 84. ^{1}H NMR spectrum of compound **6d**.

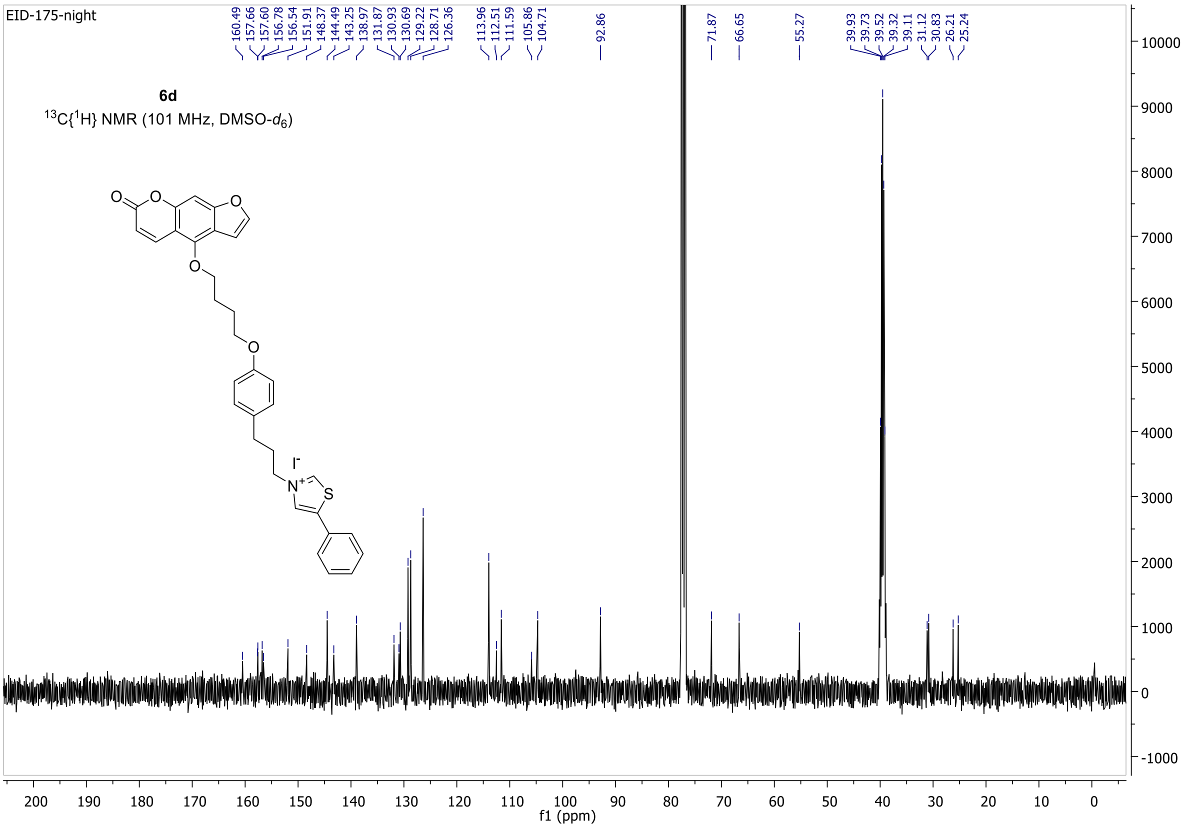

Figure S 85. ^{13}C NMR spectrum of compound **6d**.

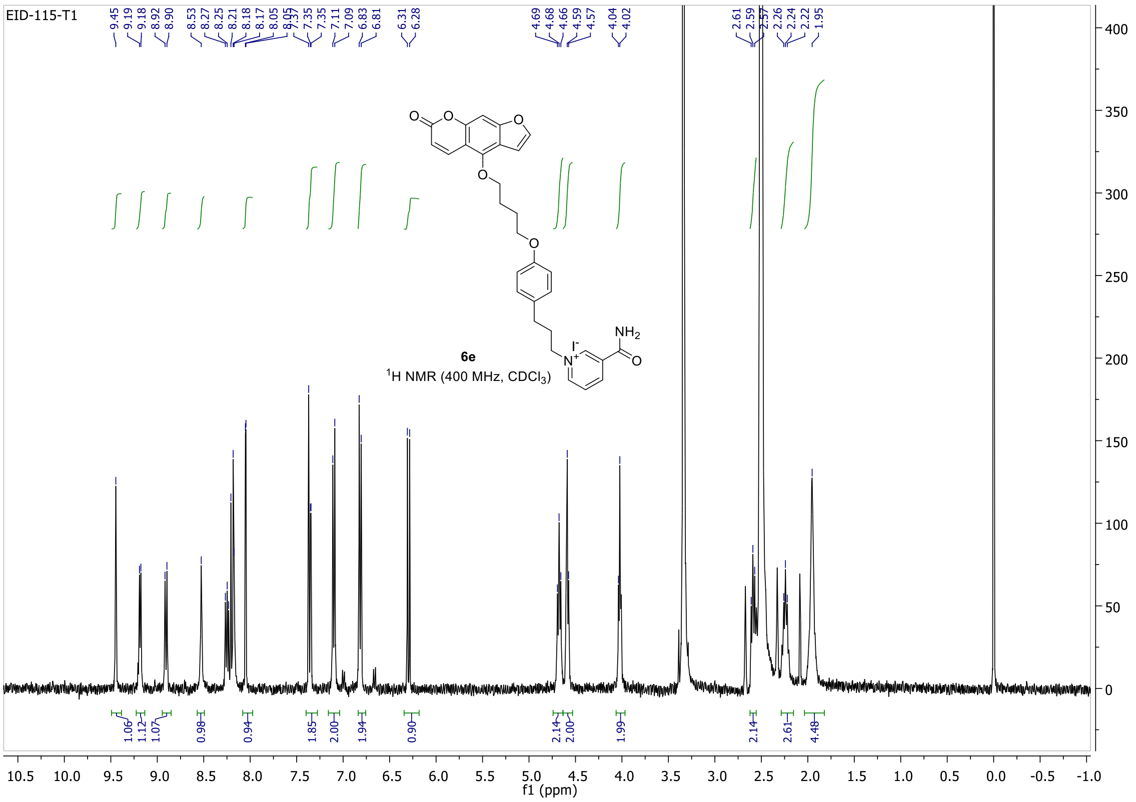

Figure S 86. ^{1}H NMR spectrum of compound **6e**.

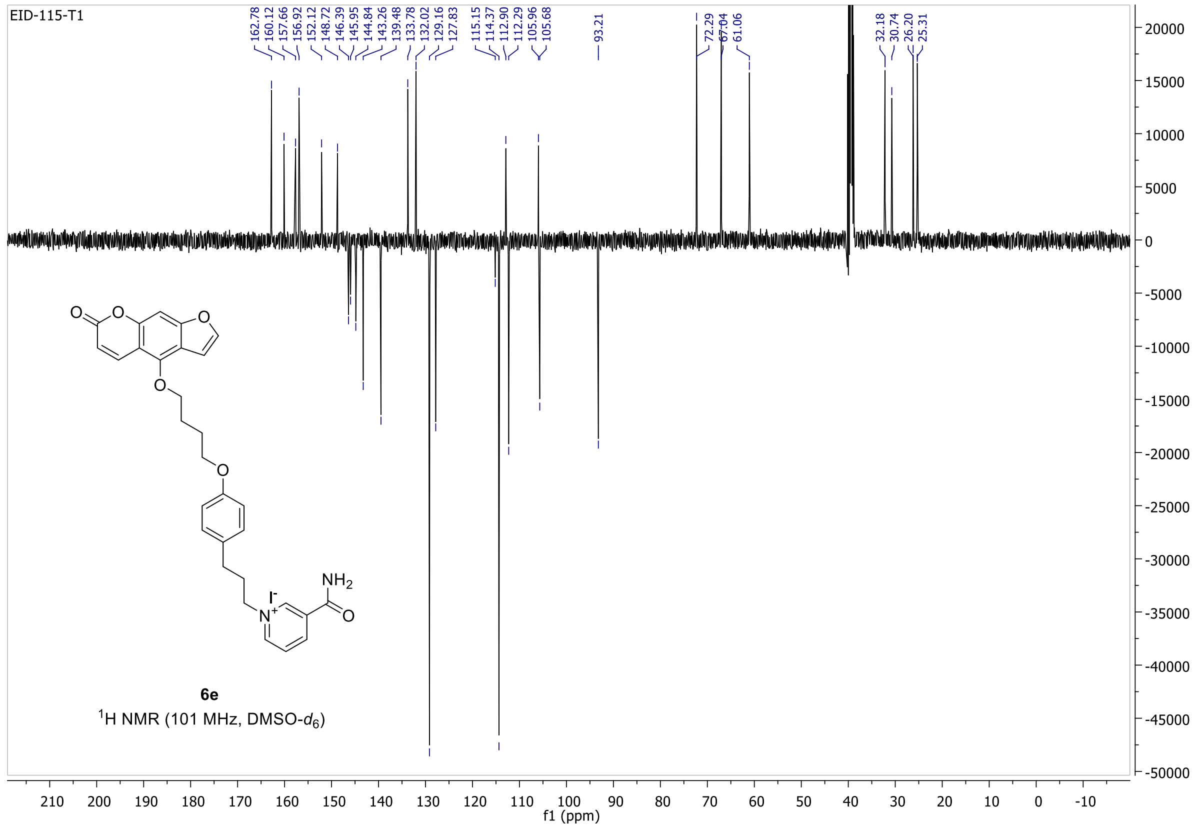

Figure S 87. ^{13}C Attached Proton Test (APT) NMR spectrum of compound **6e**.

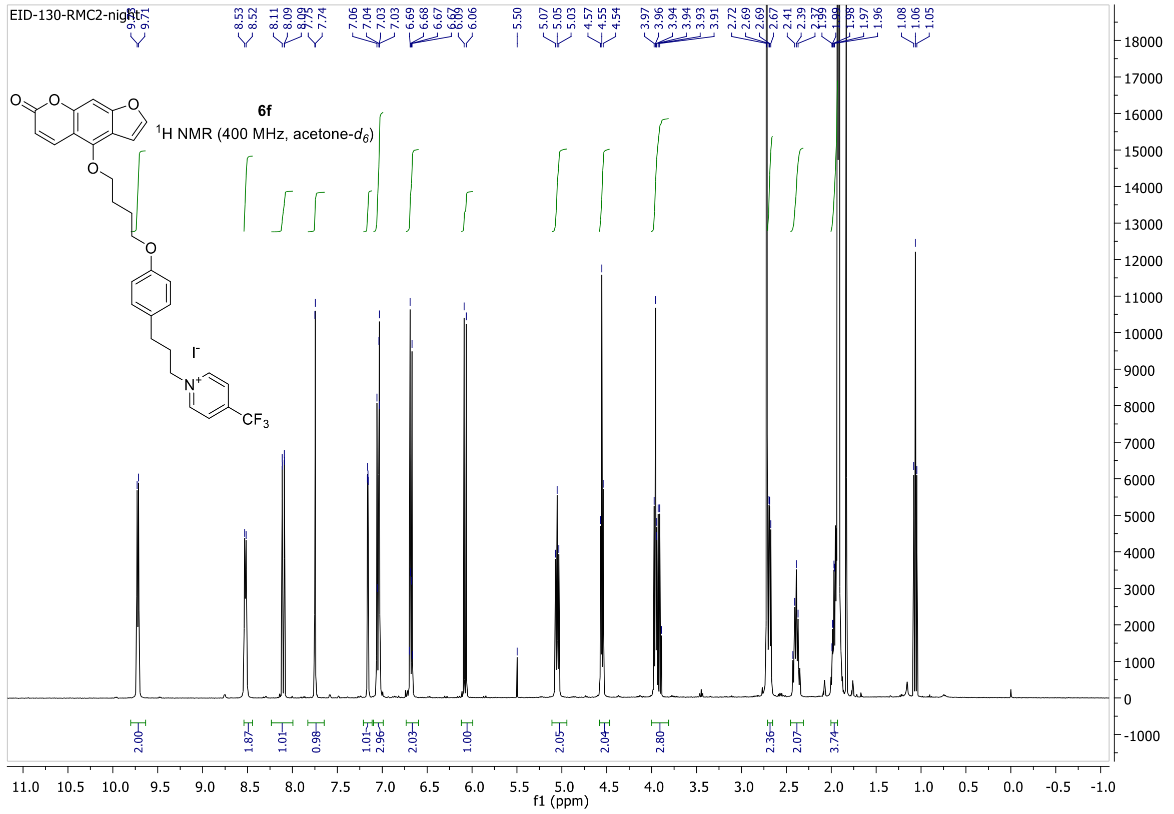

Figure S 88. ^{1}H NMR spectrum of compound **6f**.

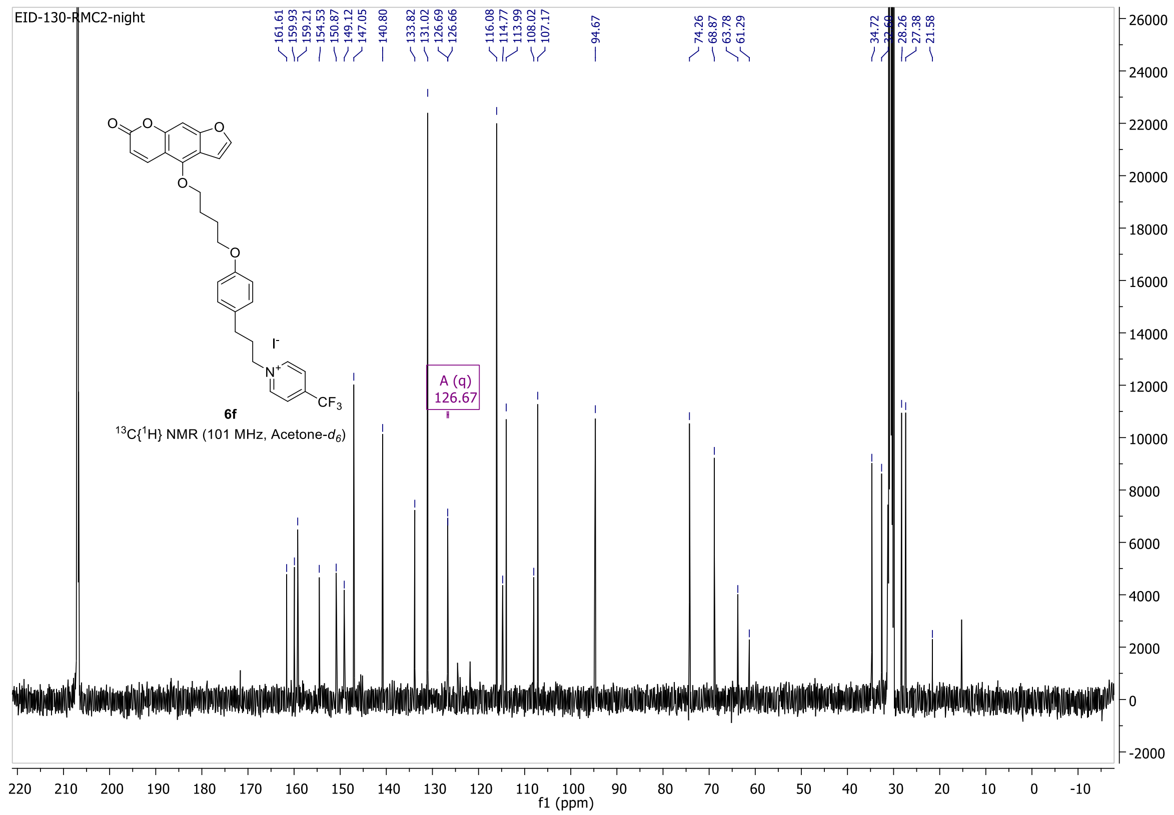

Figure S 89. ^{13}C NMR spectrum of compound **6f**.

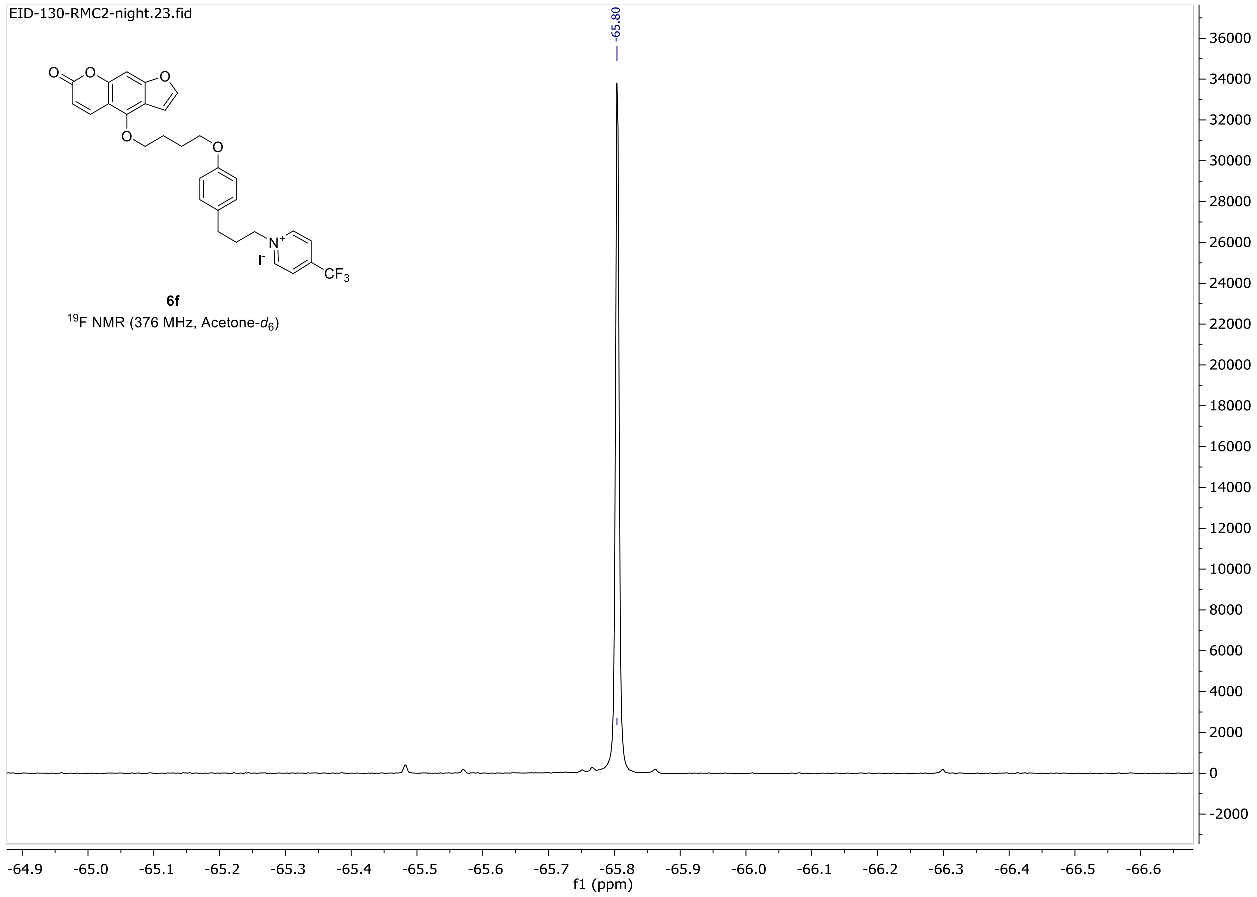

Figure S 90. ^{19}F NMR spectrum of compound **6f**.

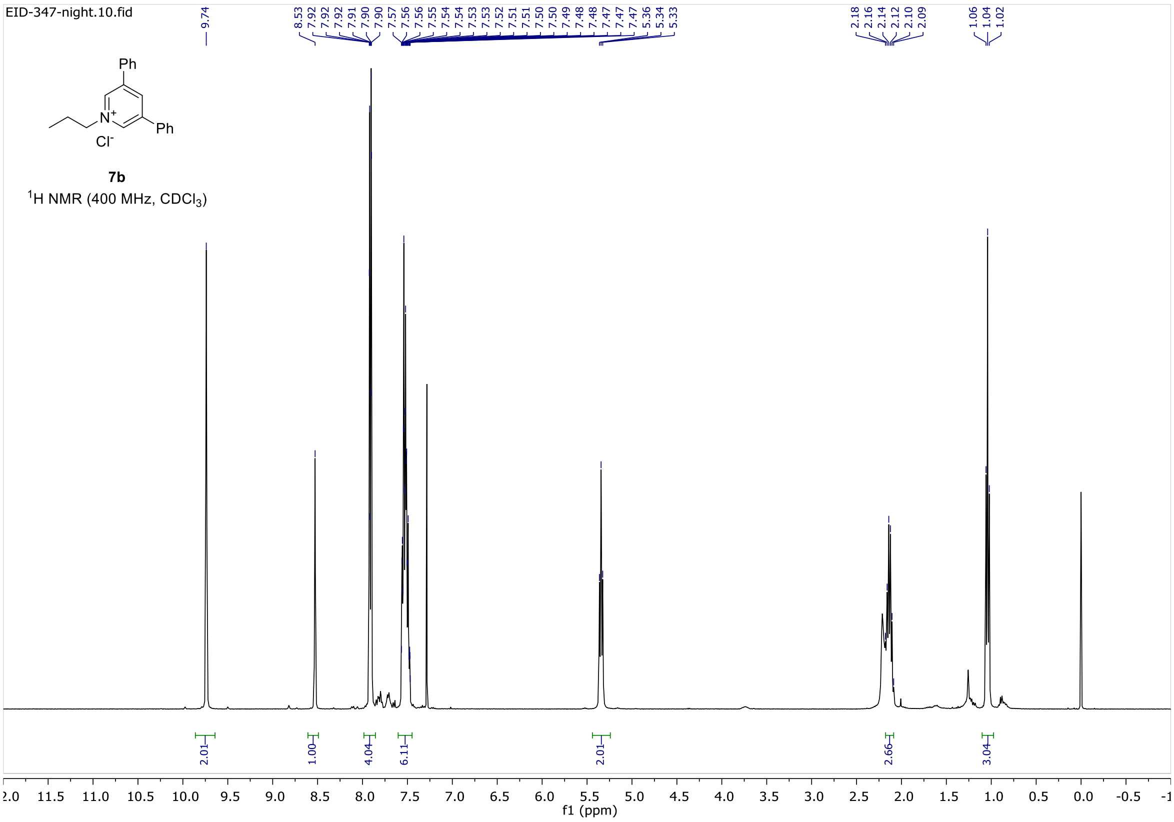

Figure S 91. ^{1}H NMR spectrum of compound **7b**.

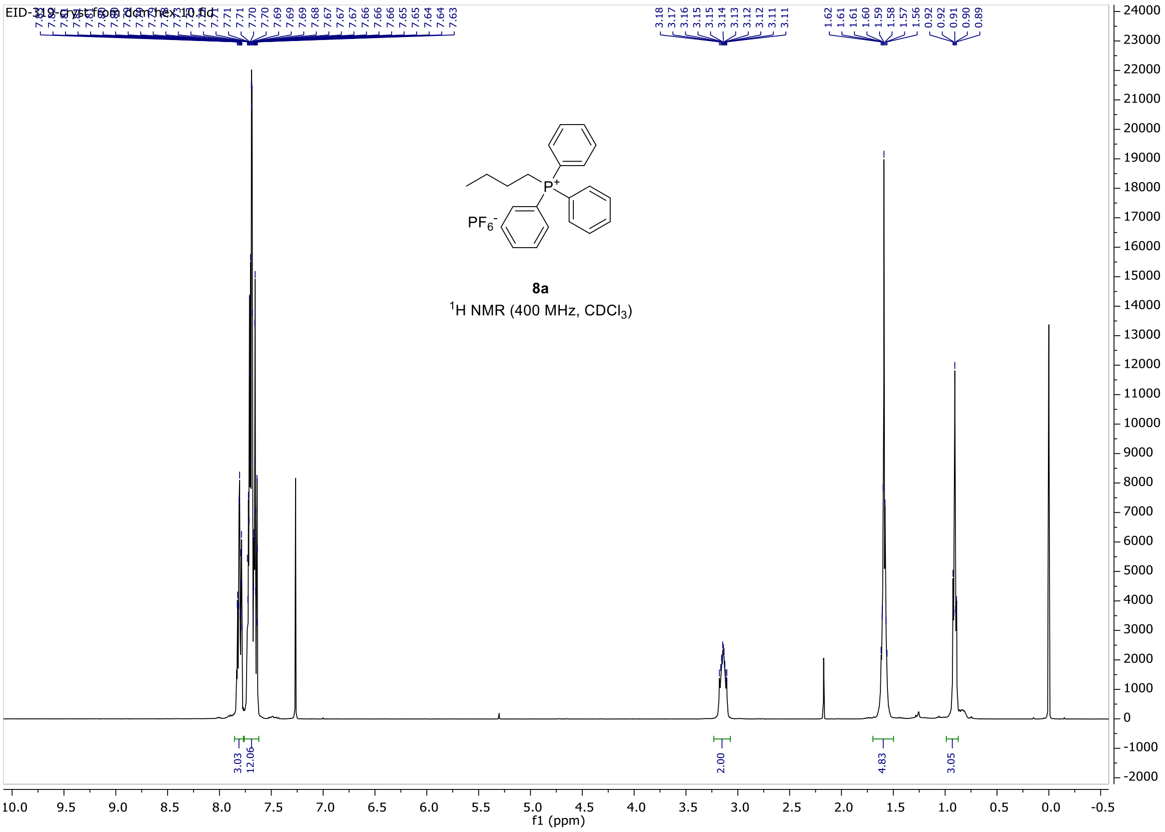

Figure S 92. ^{1}H NMR spectrum of compound **8a**.

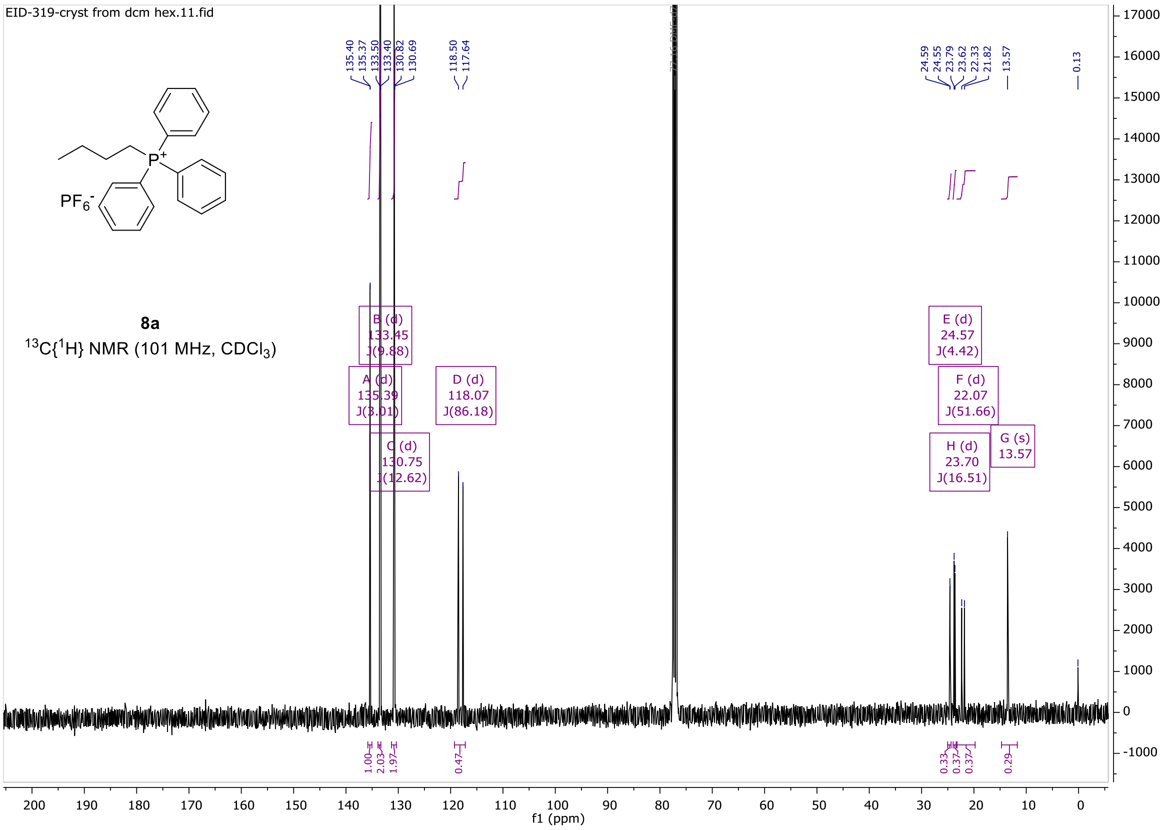

Figure S 93. ^{13}C NMR spectrum of compound **8a**.

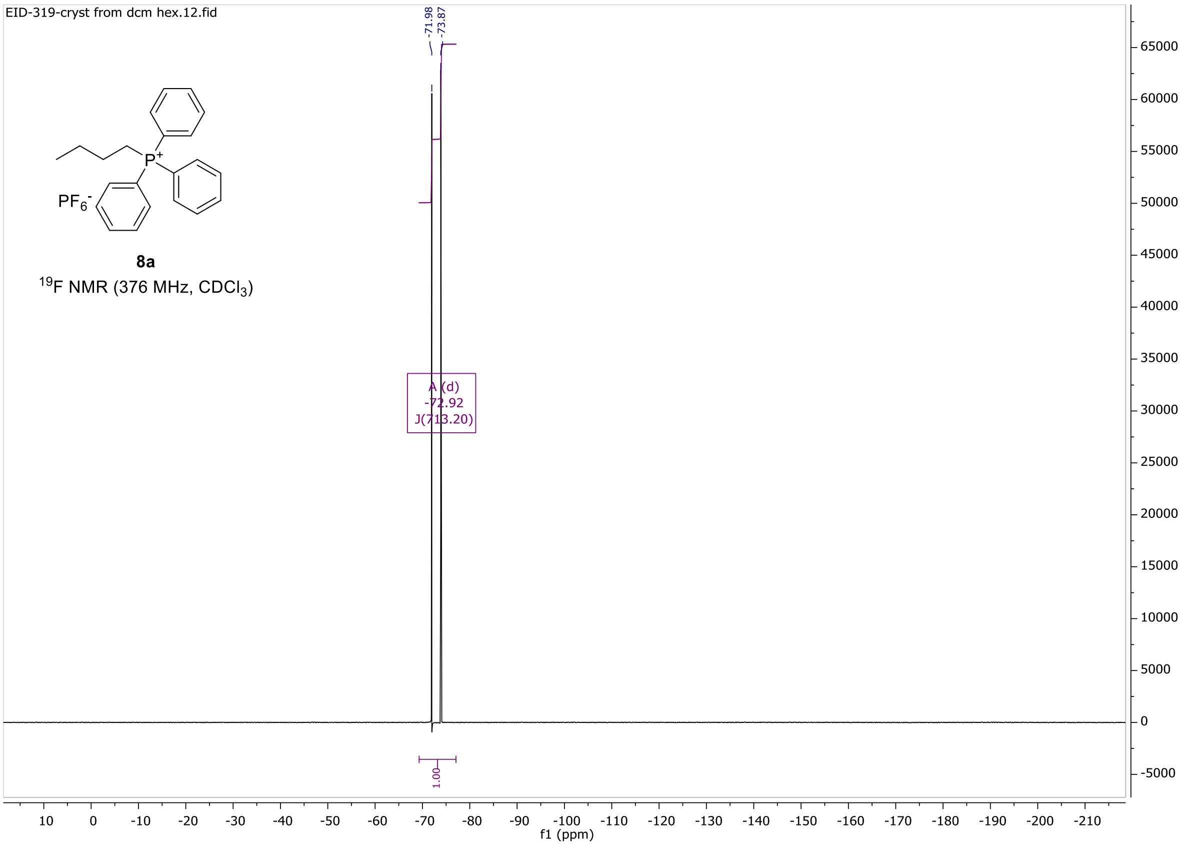

Figure S 94. ^{19}F NMR spectrum of compound **8a**.

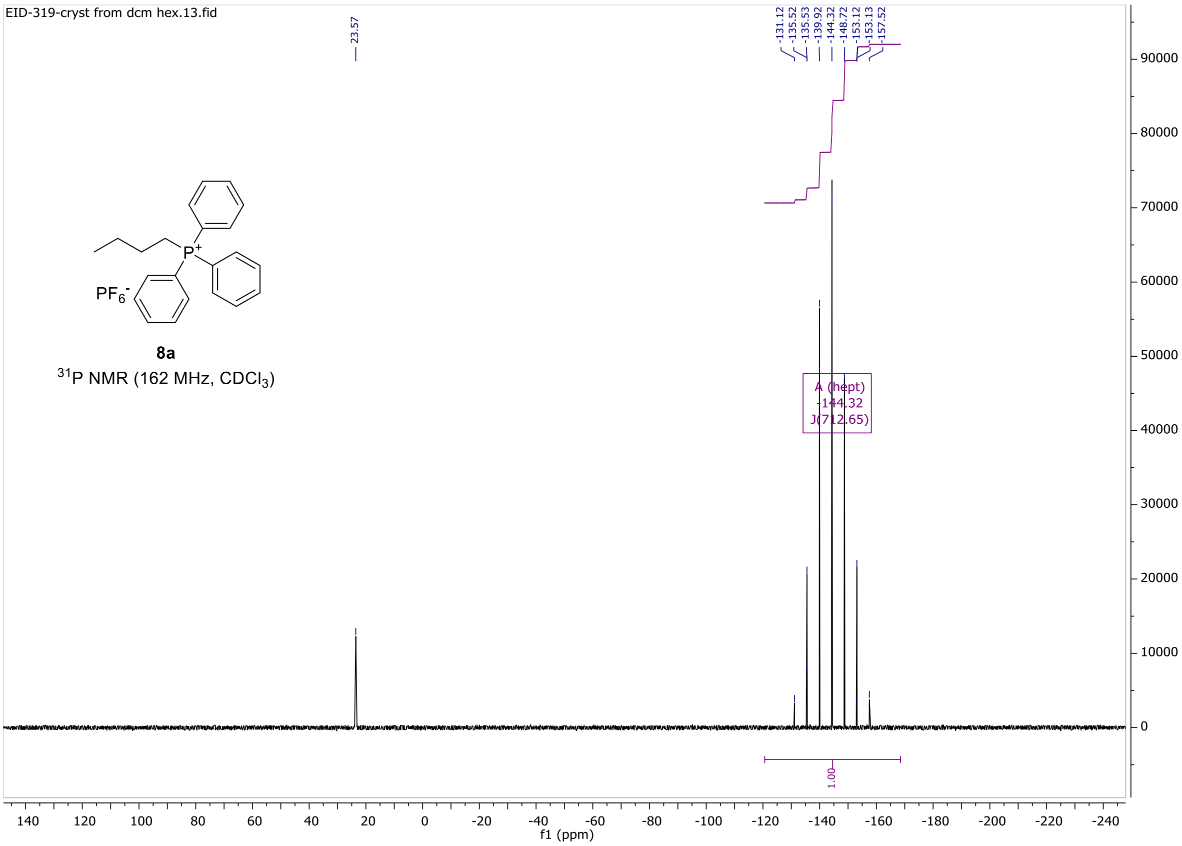

Figure S 95. ^{31}P NMR spectrum of compound **8a**.

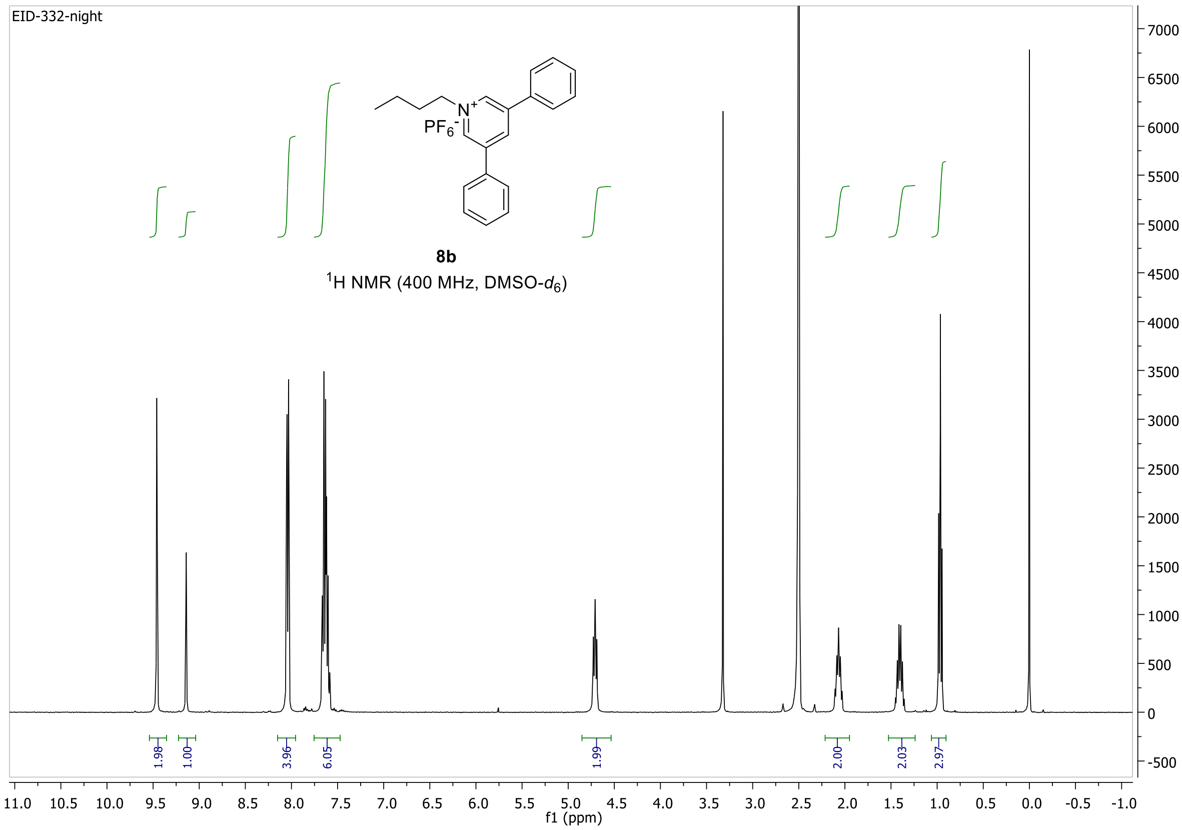

Figure S 96. ^{1}H NMR spectrum of compound **8b**.

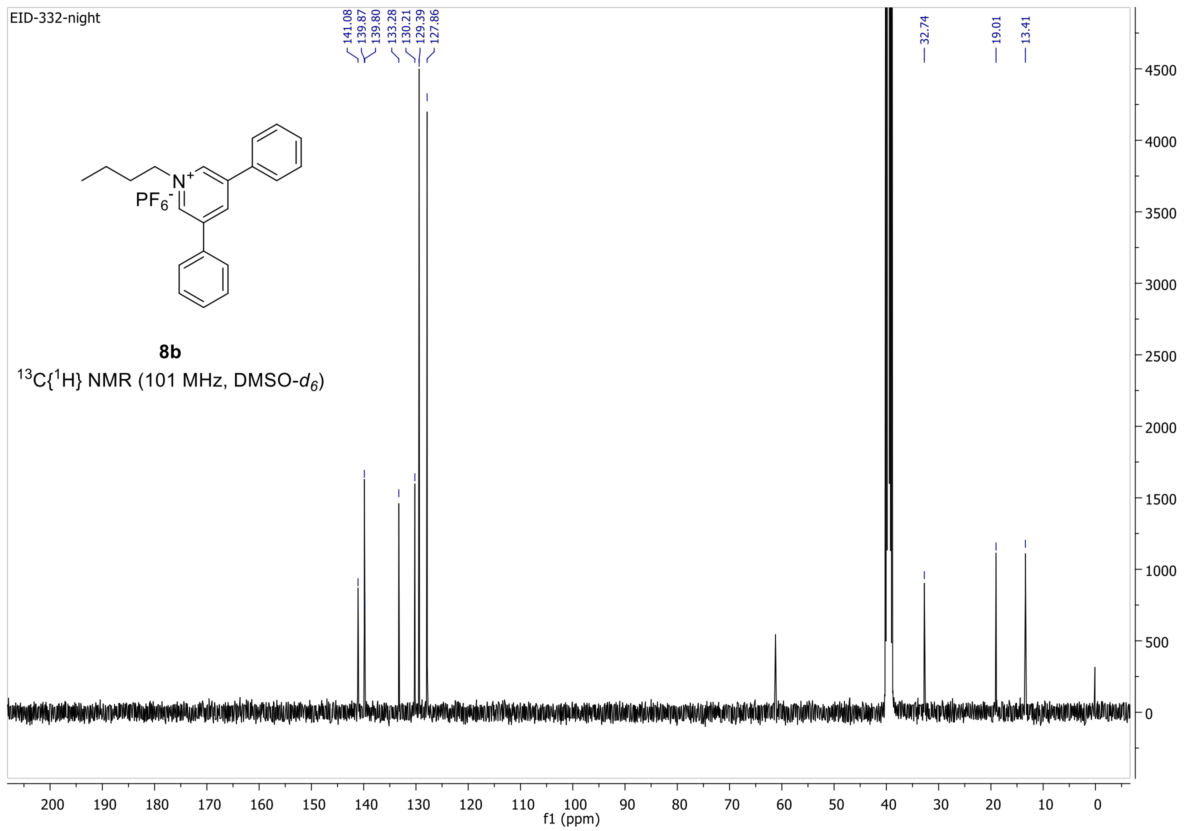

Figure S 97. ^{13}C NMR spectrum of compound **8b**.

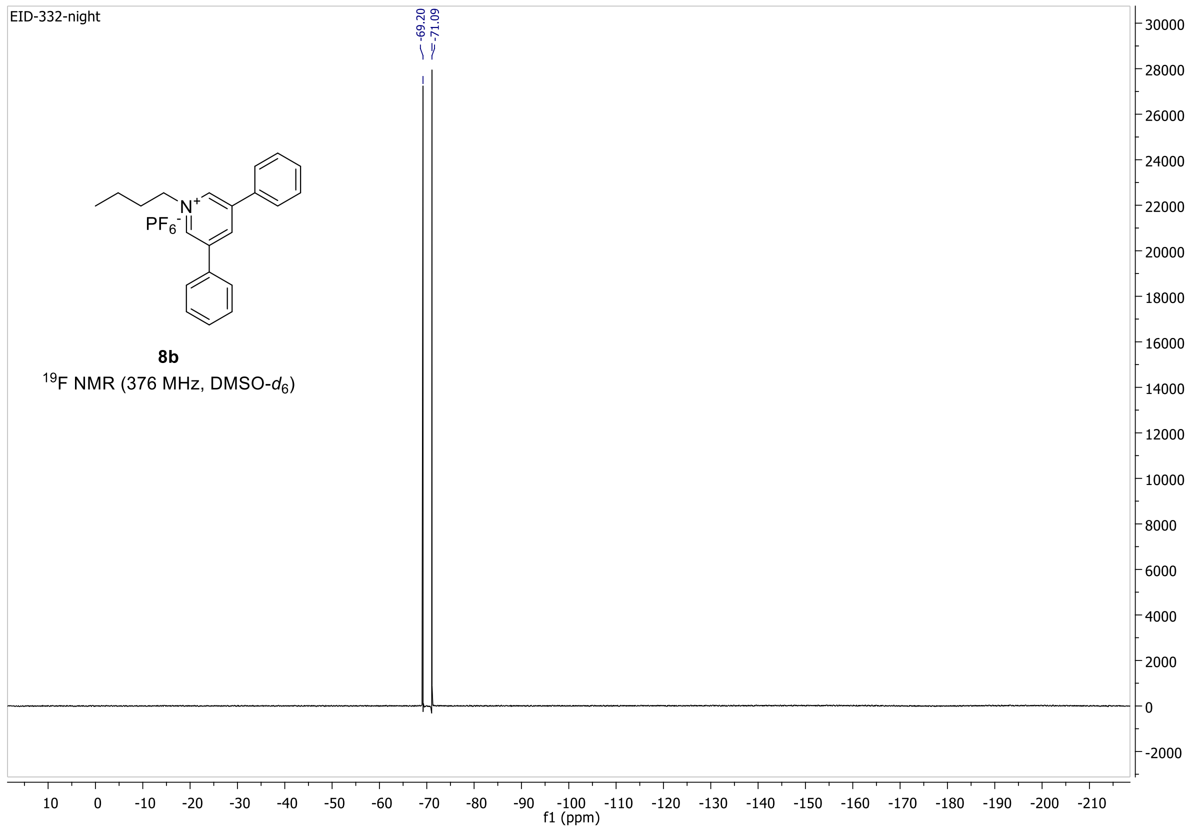

Figure S 98. ^{19}F NMR spectrum of compound **8b**.

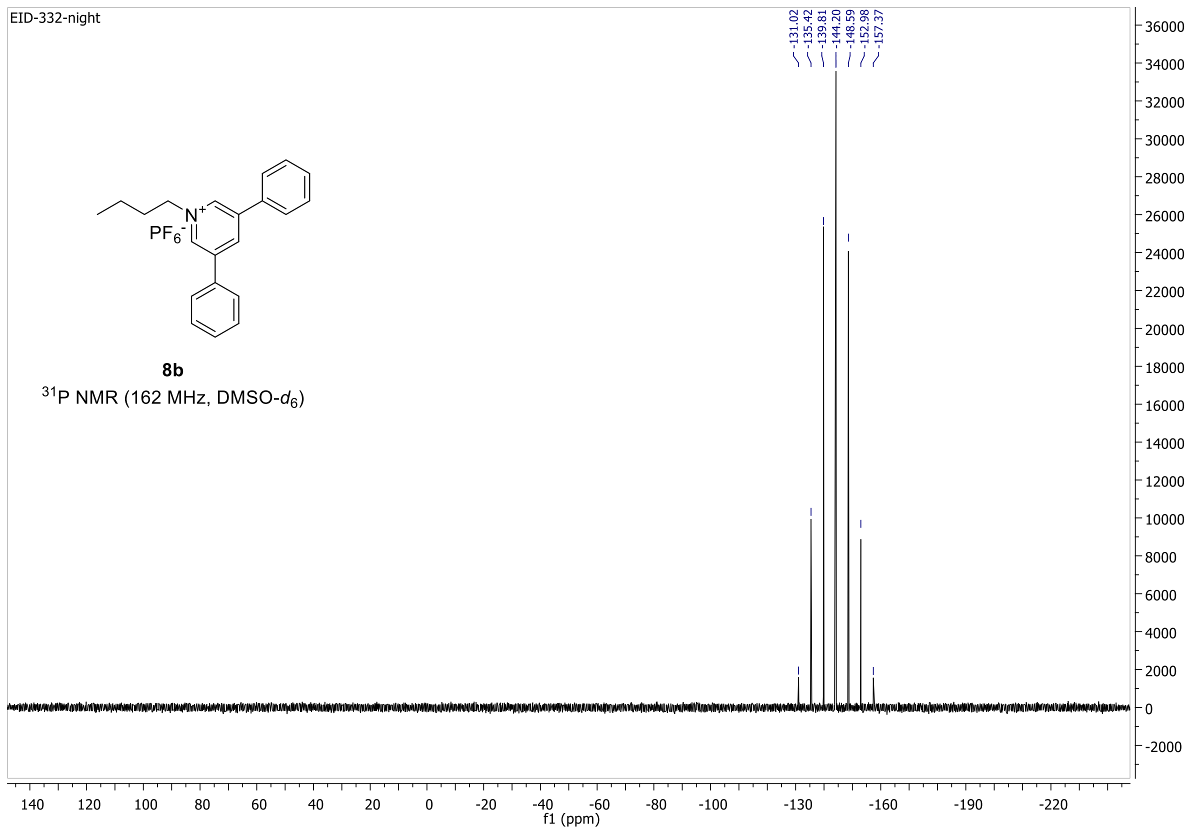

Figure S 99. ^{31}P NMR spectrum of compound **8b**.

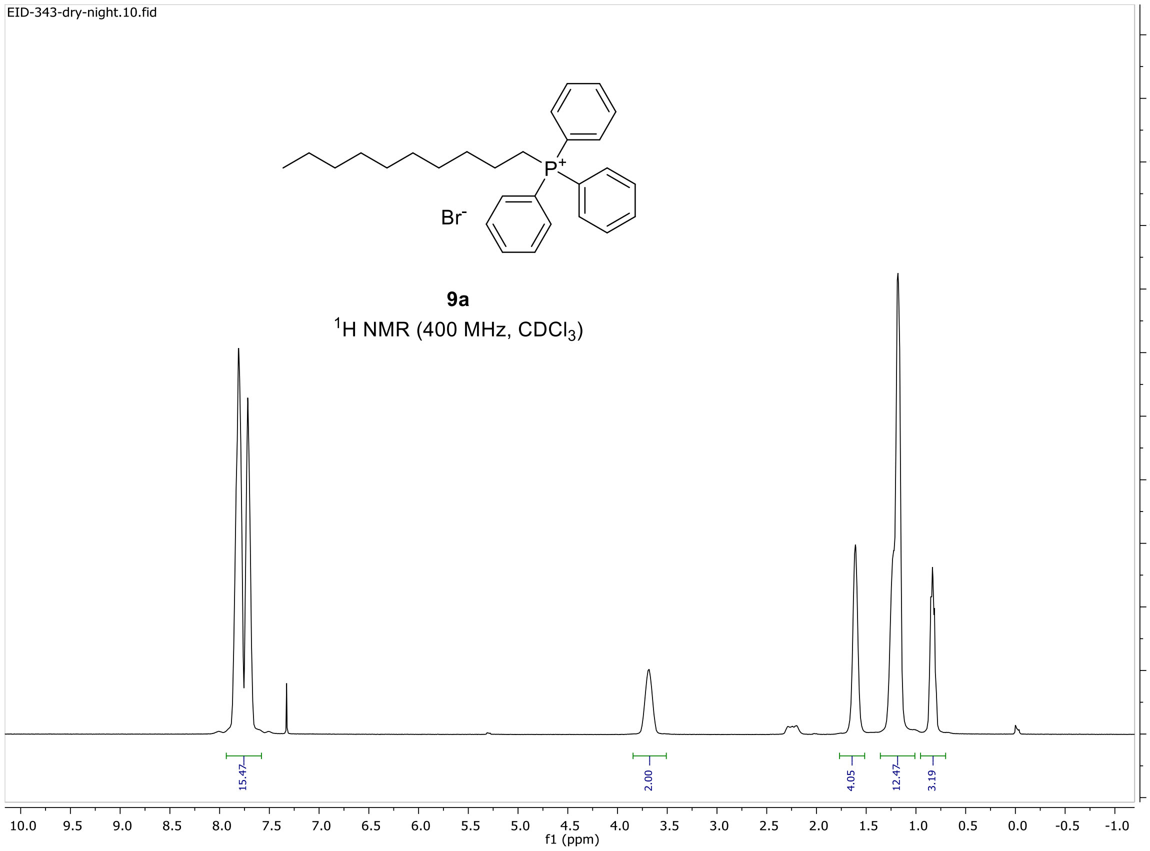

Figure S 100. 1H NMR spectrum of compound 9a.

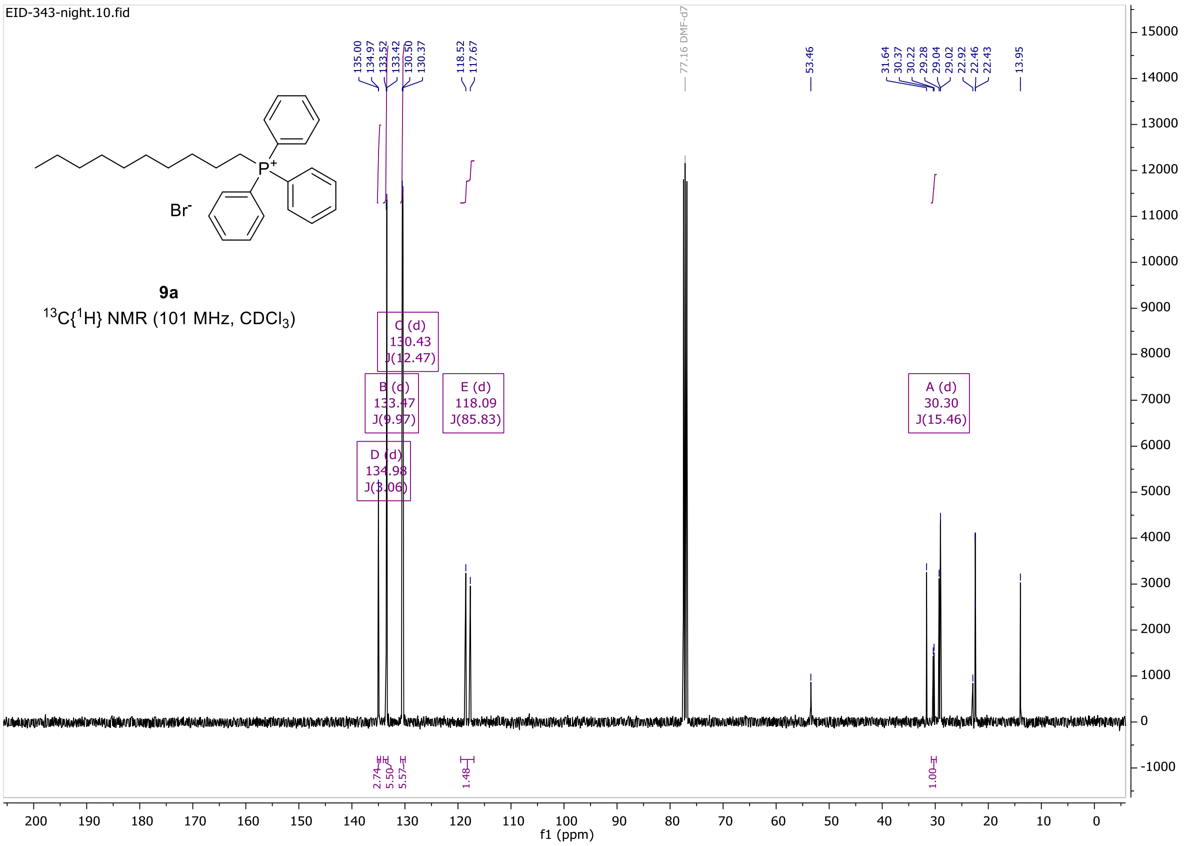

Figure S 101. ^{13}C NMR spectrum of compound **9a**.

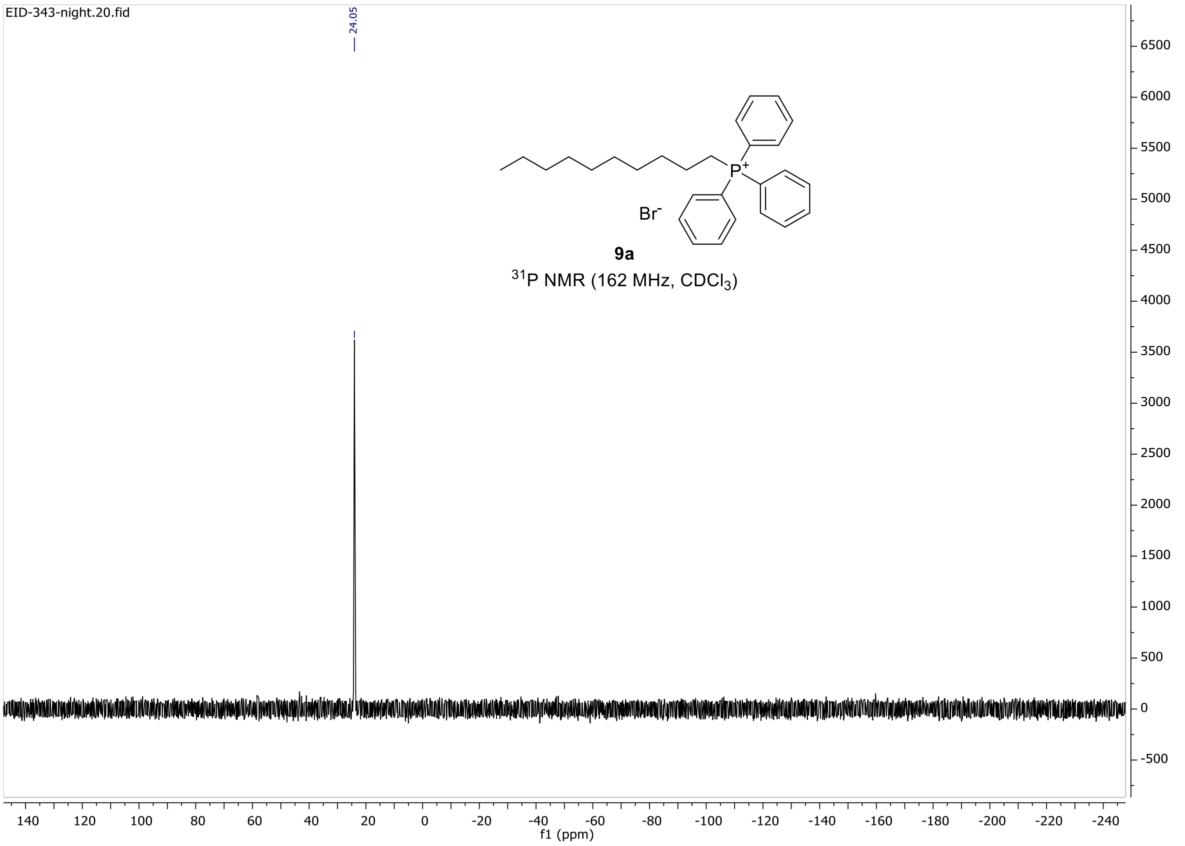

Figure S 102. ^{31}P NMR spectrum of compound **9a**.

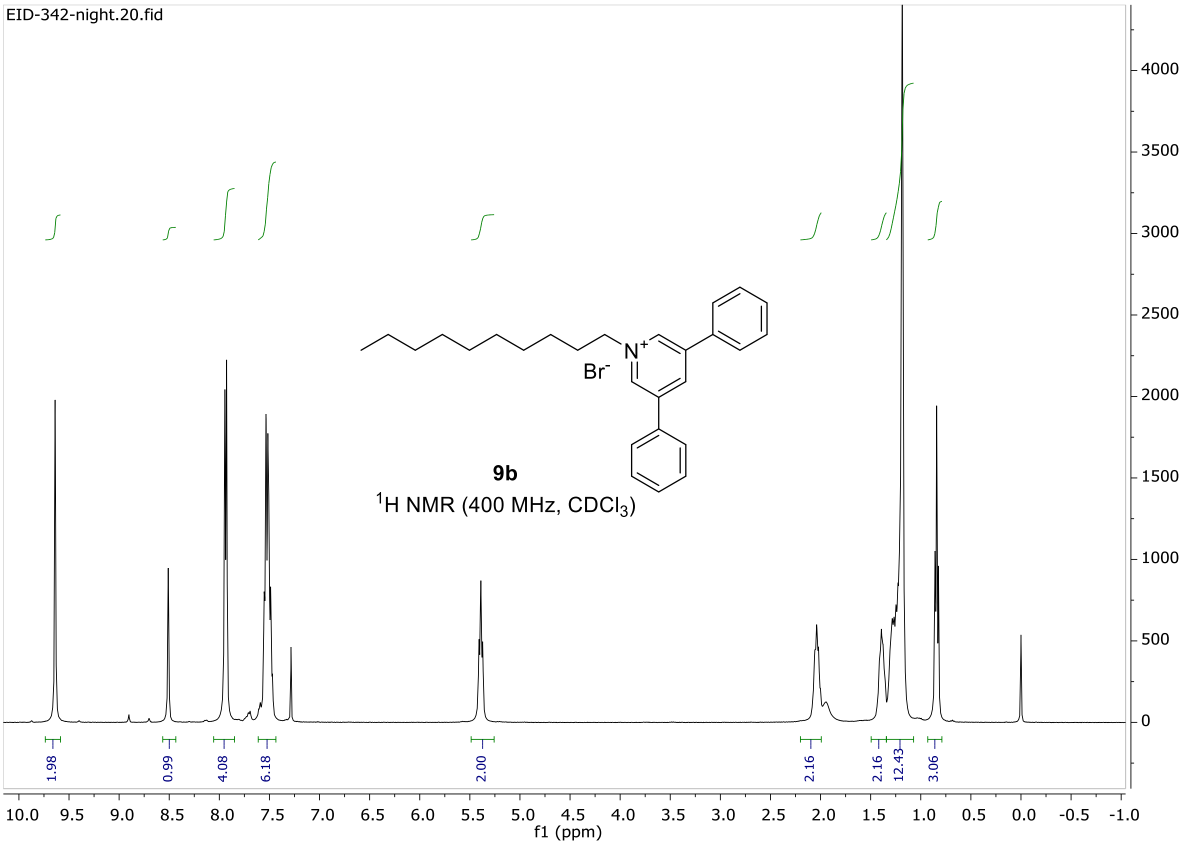

Figure S 103. ^{1}H NMR spectrum of compound **9b**.

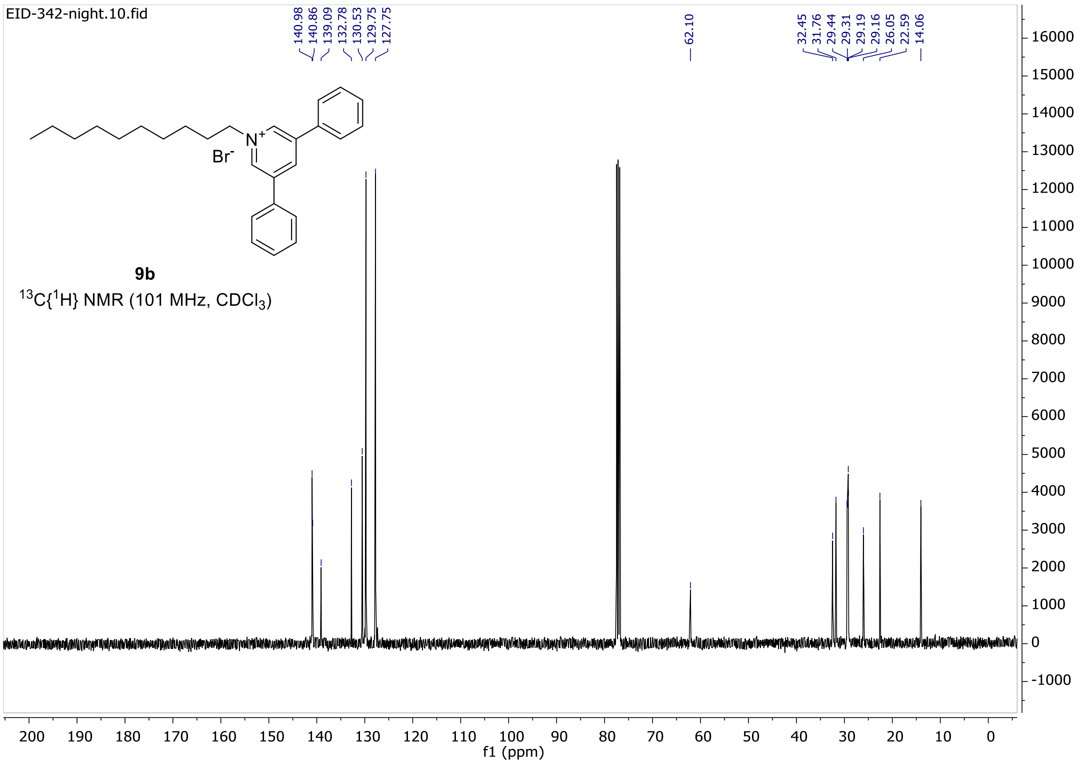

Figure S 104. ^{13}C NMR spectrum of compound **9b**.

HPLC traces (Figures S105–S123)

1a

Chromatogram and Results

Injection Details

Injection Name:	**EID-288**	*Run Time (min):*	**8,00**
Vial Number:	**RD5**	*Injection Volume:*	**2,00**
Injection Type:	**Unknown**	*Channel:*	**UV_VIS_3**
Calibration Level:		*Wavelength:*	**254**
Instrument Method:	**Purity_short**	*Bandwidth:*	**16**
Processing Method:	**Quantitative**	*Dilution Factor:*	**1,0000**
Injection Date/Time:	**03.jun..24 15:47**	*Sample Weight:*	**1,0000**

Chromatogram

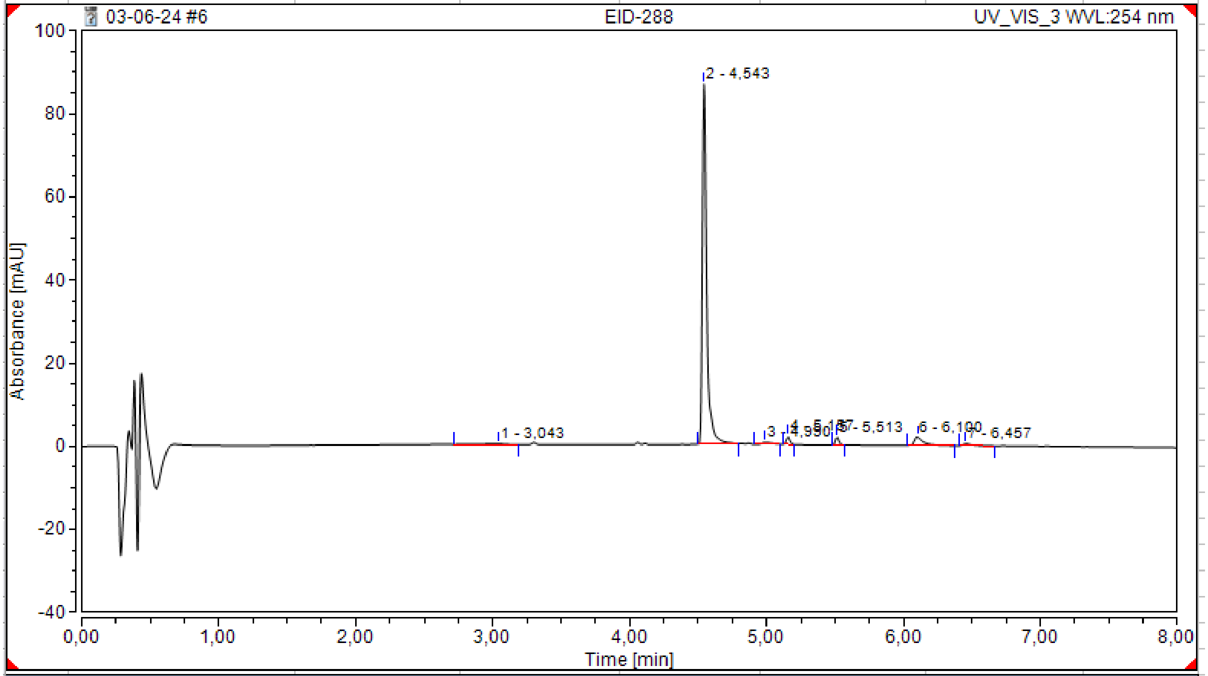

Integration Results

No.	Peak Name	Retention Time min	Area mAU*min	Height mAU	Relative Area %	Relative Height %	Amount n.a.
1		3,043	0,032	0,170	0,89	0,18	n.a.
2		4,543	3,266	86,597	90,14	92,72	n.a.
3		4,990	0,031	0,399	0,85	0,43	n.a.
4		5,157	0,054	1,840	1,50	1,97	n.a.
5		5,513	0,056	1,943	1,55	2,08	n.a.
6		6,100	0,146	1,983	4,02	2,12	n.a.
7		6,457	0,038	0,468	1,05	0,50	n.a.
**Total:**			**3,624**	**93,400**	**100,00**	**100,00**	

Figure S 105. Chromatogram of compound **1a**.

1b

Chromatogram and Results

Injection Details

Injection Name:	**EID-287**	*Run Time (min):*	**8,00**
Vial Number:	**RD4**	*Injection Volume:*	**2,00**
Injection Type:	**Unknown**	*Channel:*	**UV_VIS_3**
Calibration Level:		*Wavelength:*	**254**
Instrument Method:	**Purity_short**	*Bandwidth:*	**16**
Processing Method:	**Quantitative**	*Dilution Factor:*	**1,0000**
Injection Date/Time:	**03.jun..24 15:35**	*Sample Weight:*	**1,0000**

Chromatogram

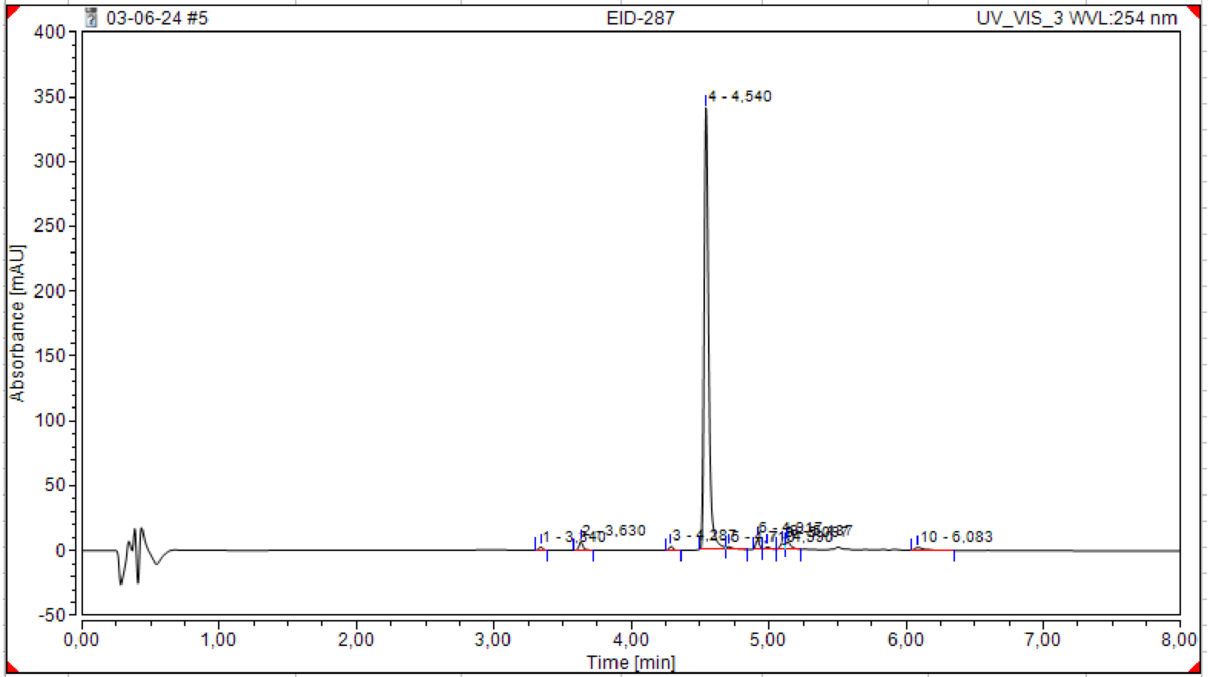

Integration Results

No.	Peak Name	Retention Time min	Area mAU*min	Height mAU	Relative Area %	Relative Height %	Amount n.a.
1		3,340	0,079	2,591	0,54	0,68	n.a.
2		3,630	0,220	6,769	1,51	1,79	n.a.
3		4,287	0,094	3,239	0,65	0,85	n.a.
4		4,540	13,111	340,833	90,14	89,95	n.a.
5		4,710	0,113	2,029	0,77	0,54	n.a.
6		4,917	0,246	8,231	1,69	2,17	n.a.
7		4,990	0,078	2,008	0,54	0,53	n.a.
8		5,097	0,169	4,904	1,16	1,29	n.a.
9		5,137	0,258	5,991	1,77	1,58	n.a.
10		6,083	0,178	2,300	1,22	0,61	n.a.
**Total:**			**14,545**	**378,894**	**100,00**	**100,00**	

Figure S 106. Chromatogram of compound **1b**.

4a

Chromatogram and Results

Injection Details

Injection Name:	**EID-352**	*Run Time (min):*	**8,00**
Vial Number:	**GB5**	*Injection Volume:*	**1,00**
Injection Type:	**Unknown**	*Channel:*	**UV_VIS_3**
Calibration Level:		*Wavelength:*	**254**
Instrument Method:	**Purity_PROTAC_short**	*Bandwidth:*	**16**
Processing Method:	**Quantitative**	*Dilution Factor:*	**1,0000**
Injection Date/Time:	**14.nov..24 12:45**	*Sample Weight:*	**1,0000**

Chromatogram

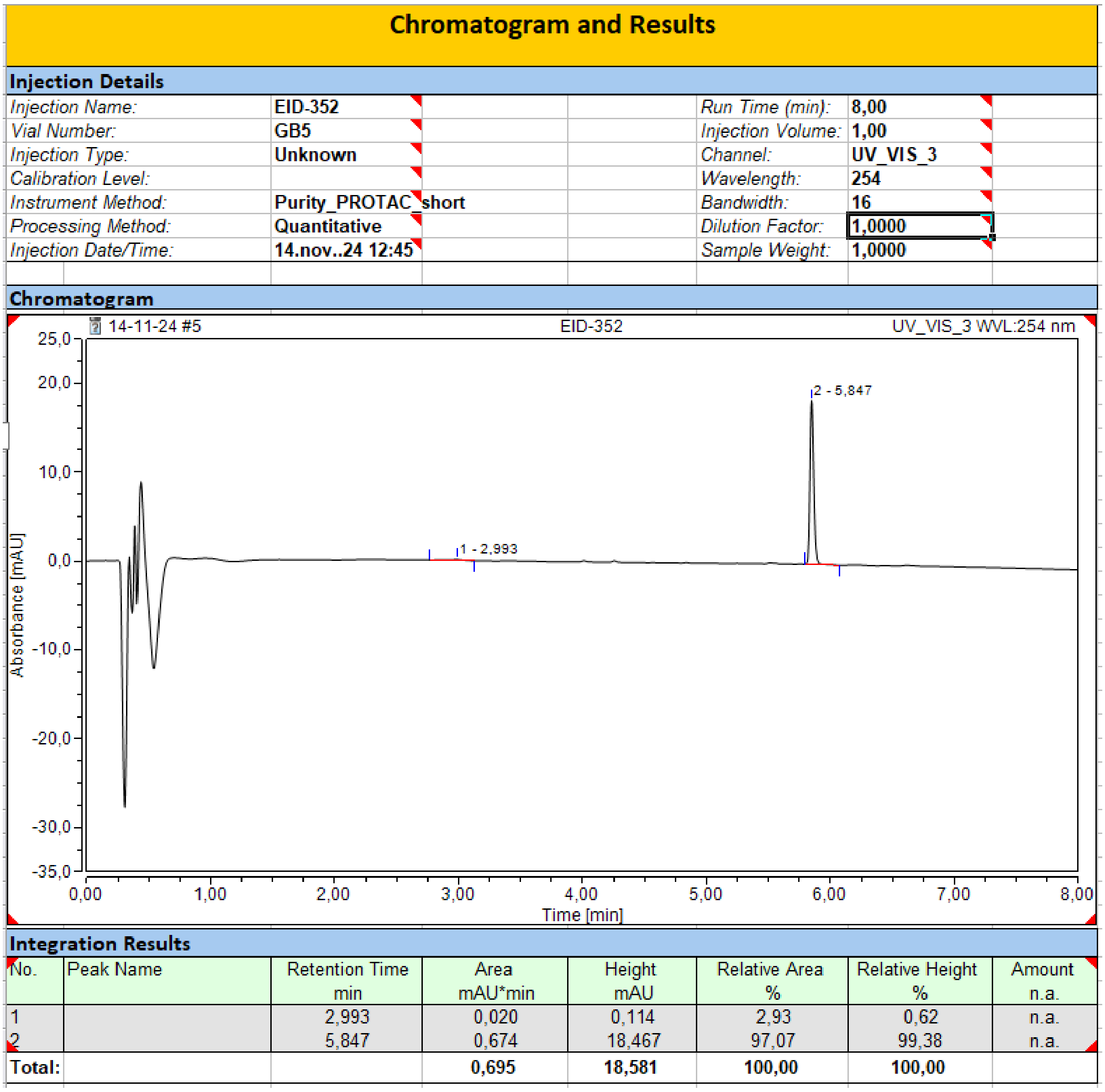

Integration Results

No.	Peak Name	Retention Time<br>min	Area<br>mAU*min	Height<br>mAU	Relative Area<br>%	Relative Height<br>%	Amount<br>n.a.
1		2,993	0,020	0,114	2,93	0,62	n.a.
2		5,847	0,674	18,467	97,07	99,38	n.a.
**Total:**			**0,695**	**18,581**	**100,00**	**100,00**	

Figure S 107. Chromatogram of compound **4a**.

4b

Chromatogram and Results

Injection Details

Injection Name:	**EID-353**	*Run Time (min):*	**8,00**
Vial Number:	**GB6**	*Injection Volume:*	**1,00**
Injection Type:	**Unknown**	*Channel:*	**UV_VIS_3**
Calibration Level:		*Wavelength:*	**254**
Instrument Method:	**Purity_PROTAC_short**	*Bandwidth:*	**16**
Processing Method:	**Quantitative**	*Dilution Factor:*	**1,0000**
Injection Date/Time:	**14.nov..24 12:57**	*Sample Weight:*	**1,0000**

Chromatogram

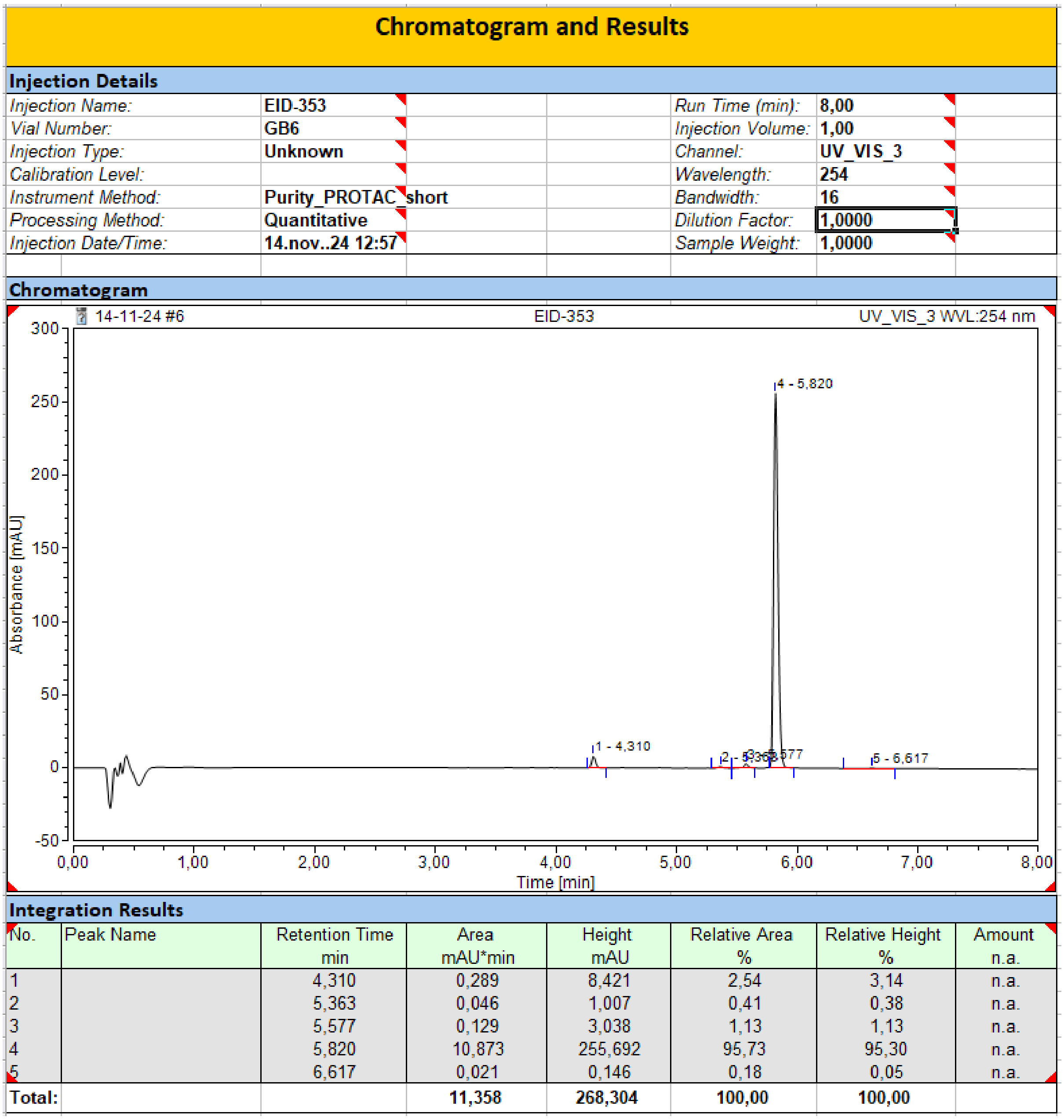

Integration Results

No.	Peak Name	Retention Time min	Area mAU*min	Height mAU	Relative Area %	Relative Height %	Amount n.a.
1		4,310	0,289	8,421	2,54	3,14	n.a.
2		5,363	0,046	1,007	0,41	0,38	n.a.
3		5,577	0,129	3,038	1,13	1,13	n.a.
4		5,820	10,873	255,692	95,73	95,30	n.a.
5		6,617	0,021	0,146	0,18	0,05	n.a.
**Total:**			**11,358**	**268,304**	**100,00**	**100,00**	

Figure S 108. Chromatogram of compound **4b**.

4c

Chromatogram and Results

Injection Details

Injection Name:	**EID-354**	*Run Time (min):*	**8,00**
Vial Number:	**GA4**	*Injection Volume:*	**2,50**
Injection Type:	**Unknown**	*Channel:*	**UV_VIS_3**
Calibration Level:		*Wavelength:*	**254**
Instrument Method:	**Purity_short**	*Bandwidth:*	**16**
Processing Method:	**Quantitative**	*Dilution Factor:*	**1,0000**
Injection Date/Time:	**03.dec..24 15:05**	*Sample Weight:*	**1,0000**

Chromatogram

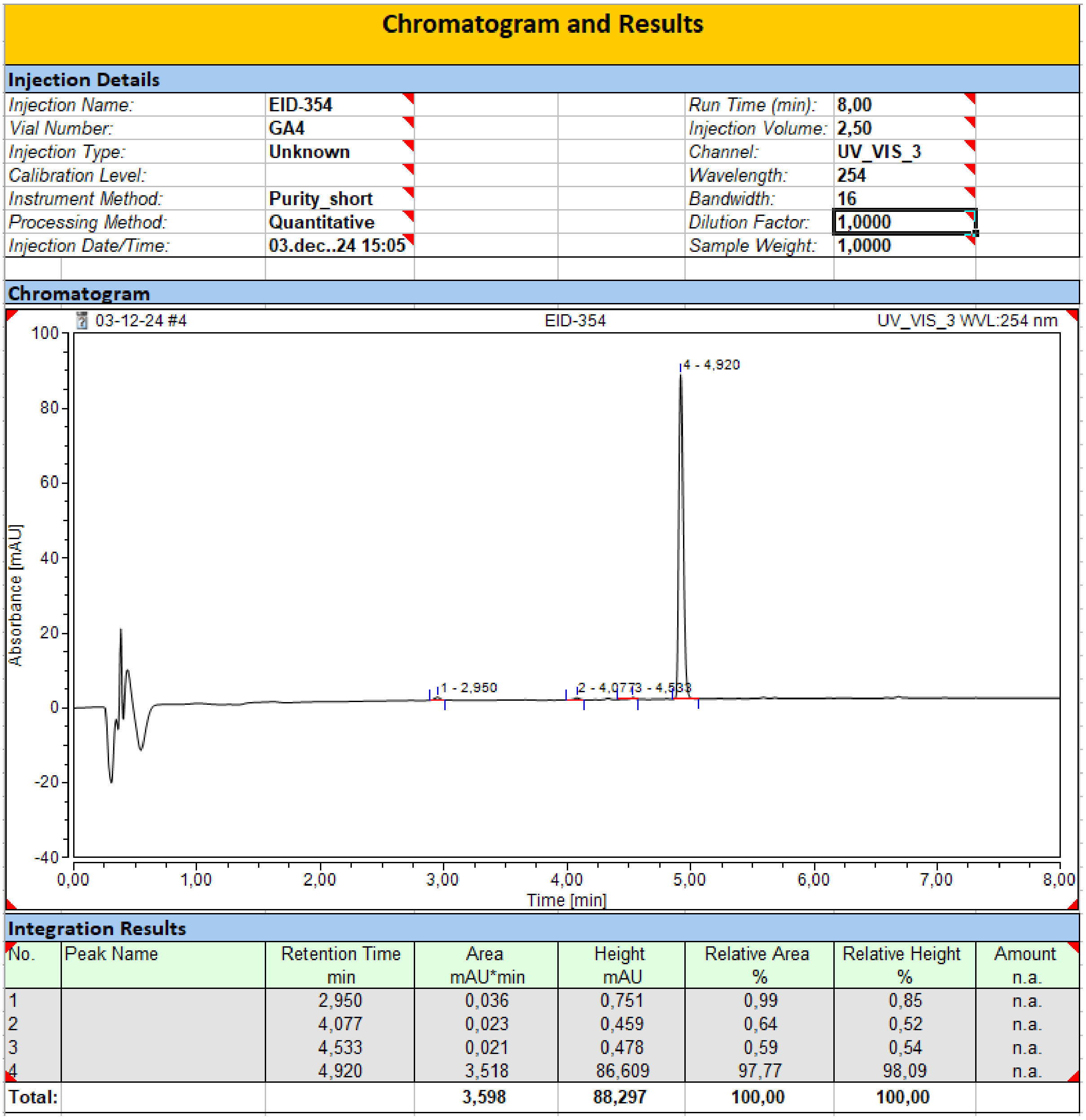

Integration Results

No.	Peak Name	Retention Time min	Area mAU*min	Height mAU	Relative Area %	Relative Height %	Amount n.a.
1		2,950	0,036	0,751	0,99	0,85	n.a.
2		4,077	0,023	0,459	0,64	0,52	n.a.
3		4,533	0,021	0,478	0,59	0,54	n.a.
4		4,920	3,518	86,609	97,77	98,09	n.a.
**Total:**			**3,598**	**88,297**	**100,00**	**100,00**	

Figure S 109. Chromatogram of compound **4c**.

4c-d_5

Chromatogram and Results

Injection Details

Injection Name:	**UL-EID-394**	*Run Time (min):*	**8,00**
Vial Number:	**GA3**	*Injection Volume:*	**0,50**
Injection Type:	**Unknown**	*Channel:*	**UV_VIS_3**
Calibration Level:		*Wavelength:*	**254**
Instrument Method:	**Purity_short**	*Bandwidth:*	**16**
Processing Method:	**Quantitative**	*Dilution Factor:*	**1,0000**
Injection Date/Time:	**23.mar..26 21:24**	*Sample Weight:*	**1,0000**

Chromatogram

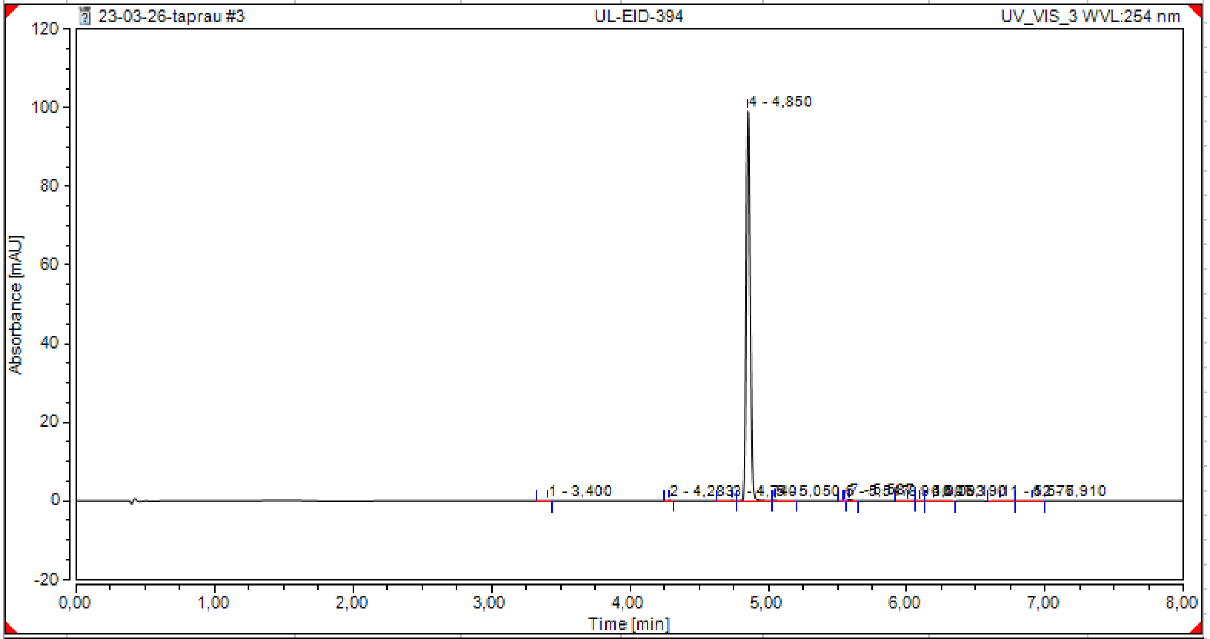

Integration Results

No.	Peak Name	Retention Time min	Area mAU*min	Height mAU	Relative Area %	Relative Height %	Amount n.a.
1		3,400	0,003	0,061	0,07	0,06	n.a.
2		4,283	0,003	0,109	0,10	0,11	n.a.
3		4,740	0,003	0,080	0,10	0,08	n.a.
4		4,850	3,429	99,296	98,19	98,89	n.a.
5		5,050	0,006	0,112	0,18	0,11	n.a.
6		5,547	0,000	0,012	0,00	0,01	n.a.
7		5,587	0,014	0,369	0,39	0,37	n.a.
8		6,007	0,003	0,053	0,10	0,05	n.a.
9		6,093	0,002	0,047	0,06	0,05	n.a.
10		6,190	0,008	0,100	0,24	0,10	n.a.
11		6,677	0,009	0,083	0,26	0,08	n.a.
12		6,910	0,011	0,090	0,31	0,09	n.a.
**Total:**			**3,492**	**100,412**	**100,00**	**100,00**	

Figure S 110. Chromatogram of compound **4c-d_5.**

4d

Chromatogram and Results

Injection Details

Injection Name:	**EID-360**	*Run Time (min):*	**8,00**
Vial Number:	**BC4**	*Injection Volume:*	**1,00**
Injection Type:	**Unknown**	*Channel:*	**UV_VIS_3**
Calibration Level:		*Wavelength:*	**254**
Instrument Method:	**Purity_PROTAC_short**	*Bandwidth:*	**16**
Processing Method:	**Quantitative**	*Dilution Factor:*	**1,0000**
Injection Date/Time:	**16.dec..24 15:42**	*Sample Weight:*	**1,0000**

Chromatogram

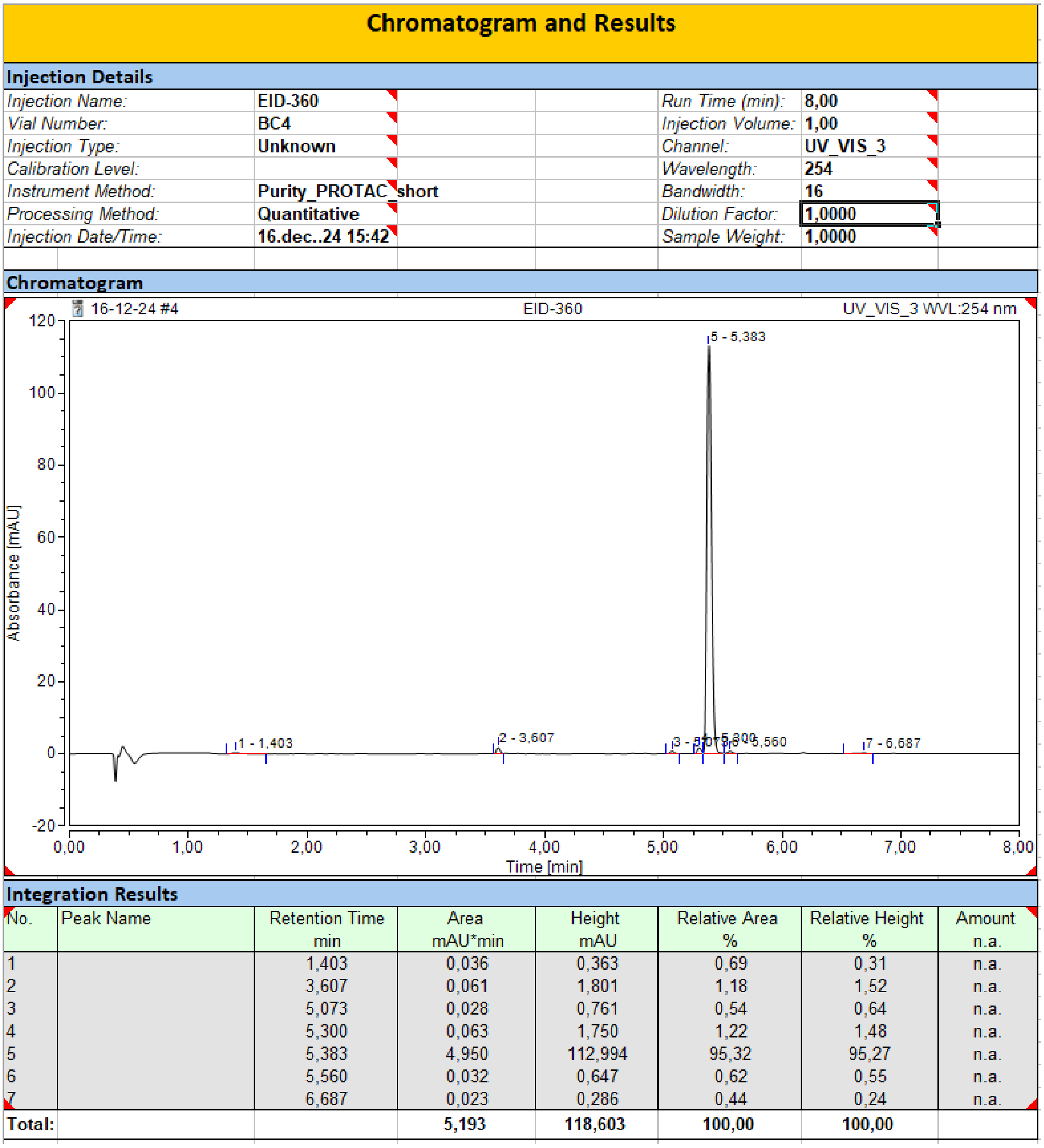

Integration Results

No.	Peak Name	Retention Time min	Area mAU*min	Height mAU	Relative Area %	Relative Height %	Amount n.a.
1		1,403	0,036	0,363	0,69	0,31	n.a.
2		3,607	0,061	1,801	1,18	1,52	n.a.
3		5,073	0,028	0,761	0,54	0,64	n.a.
4		5,300	0,063	1,750	1,22	1,48	n.a.
5		5,383	4,950	112,994	95,32	95,27	n.a.
6		5,560	0,032	0,647	0,62	0,55	n.a.
7		6,687	0,023	0,286	0,44	0,24	n.a.
**Total:**			**5,193**	**118,603**	**100,00**	**100,00**	

Figure S 111. Chromatogram of compound **4d**.

4e

Chromatogram and Results

Injection Details

Injection Name:	EID-359	*Run Time (min):*	8,00
Vial Number:	BC3	*Injection Volume:*	1,00
Injection Type:	Unknown	*Channel:*	UV_VIS_3
Calibration Level:		*Wavelength:*	254
Instrument Method:	Purity_PROTAC_short	*Bandwidth:*	16
Processing Method:	Quantitative	*Dilution Factor:*	1,0000
Injection Date/Time:	16.dec..24 15:30	*Sample Weight:*	1,0000

Chromatogram

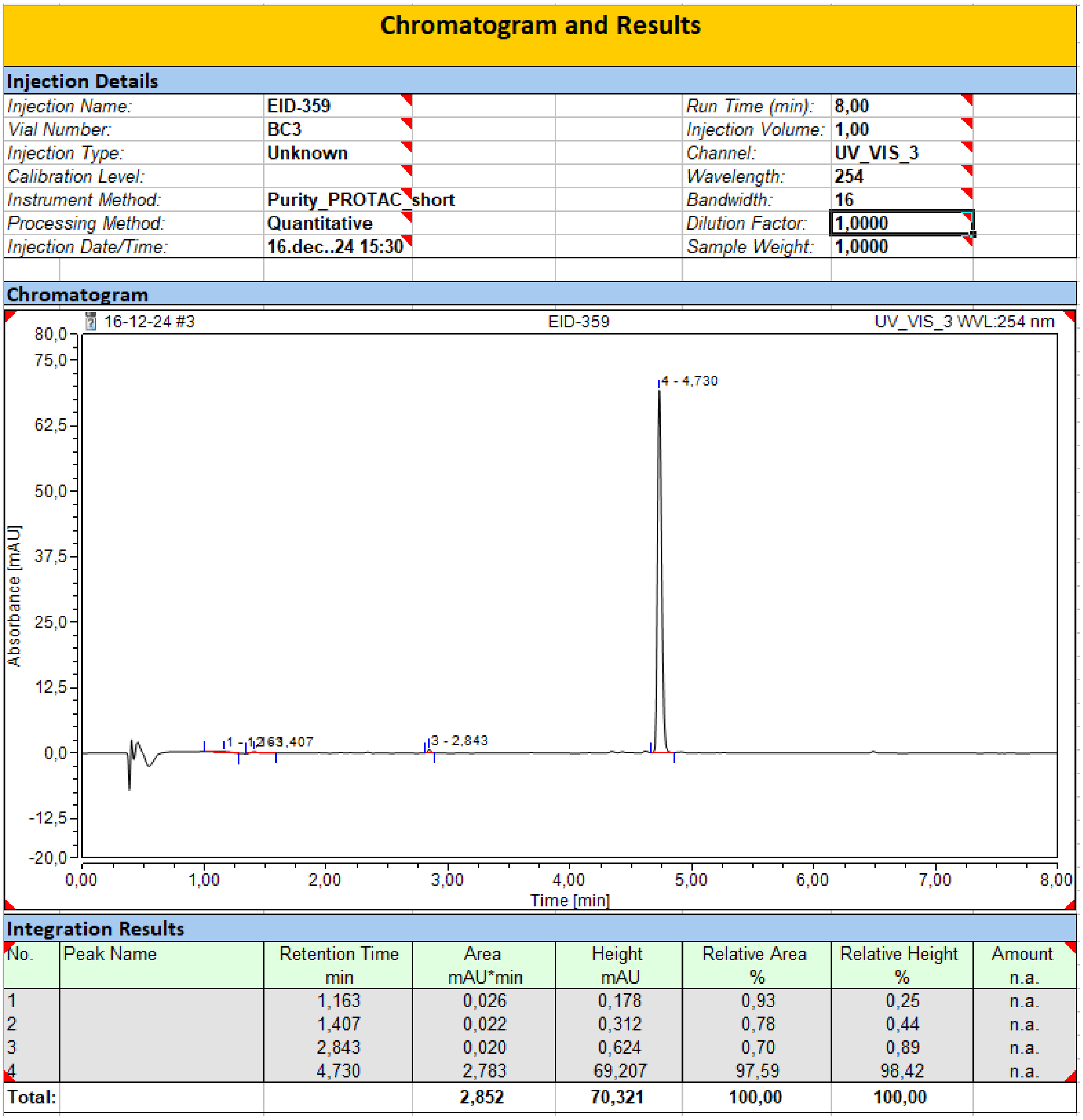

Integration Results

No.	Peak Name	Retention Time min	Area mAU*min	Height mAU	Relative Area %	Relative Height %	Amount n.a.
1		1,163	0,026	0,178	0,93	0,25	n.a.
2		1,407	0,022	0,312	0,78	0,44	n.a.
3		2,843	0,020	0,624	0,70	0,89	n.a.
4		4,730	2,783	69,207	97,59	98,42	n.a.
**Total:**			**2,852**	**70,321**	**100,00**	**100,00**	

Figure S 112. Chromatogram of compound **4e**.

4f

Chromatogram and Results

Injection Details

Injection Name:	**EID-362**	*Run Time (min):*	**8,00**
Vial Number:	**BC5**	*Injection Volume:*	**1,00**
Injection Type:	**Unknown**	*Channel:*	**UV_VIS_3**
Calibration Level:		*Wavelength:*	**254**
Instrument Method:	**Purity_PROTAC_short**	*Bandwidth:*	**16**
Processing Method:	**Quantitative**	*Dilution Factor:*	**1,0000**
Injection Date/Time:	**16.dec..24 15:54**	*Sample Weight:*	**1,0000**

Chromatogram

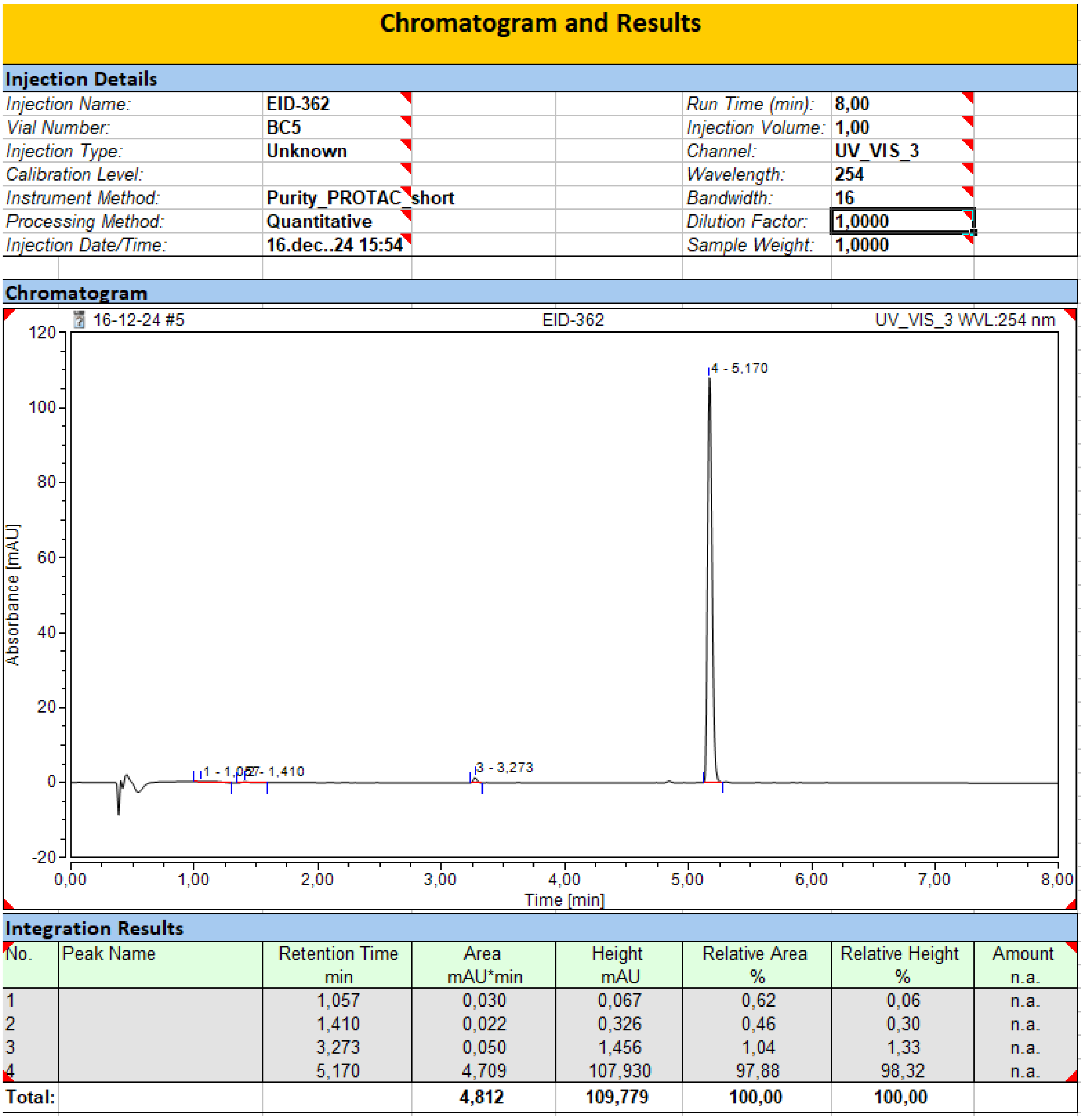

Integration Results

No.	Peak Name	Retention Time min	Area mAU*min	Height mAU	Relative Area %	Relative Height %	Amount n.a.
1		1,057	0,030	0,067	0,62	0,06	n.a.
2		1,410	0,022	0,326	0,46	0,30	n.a.
3		3,273	0,050	1,456	1,04	1,33	n.a.
4		5,170	4,709	107,930	97,88	98,32	n.a.
**Total:**			**4,812**	**109,779**	**100,00**	**100,00**	

Figure S 113. Chromatogram of compound **4f**.

6a

Chromatogram and Results

Injection Details

Injection Name:	**EID-216**	*Run Time (min):*	**14,50**
Vial Number:	**RC3**	*Injection Volume:*	**1,00**
Injection Type:	**Unknown**	*Channel:*	**UV_VIS_3**
Calibration Level:		*Wavelength:*	**254**
Instrument Method:	**Purity_PROTAC**	*Bandwidth:*	**16**
Processing Method:	**Quantitative**	*Dilution Factor:*	**1,0000**
Injection Date/Time:	**03.okt..24 13:19**	*Sample Weight:*	**1,0000**

Chromatogram

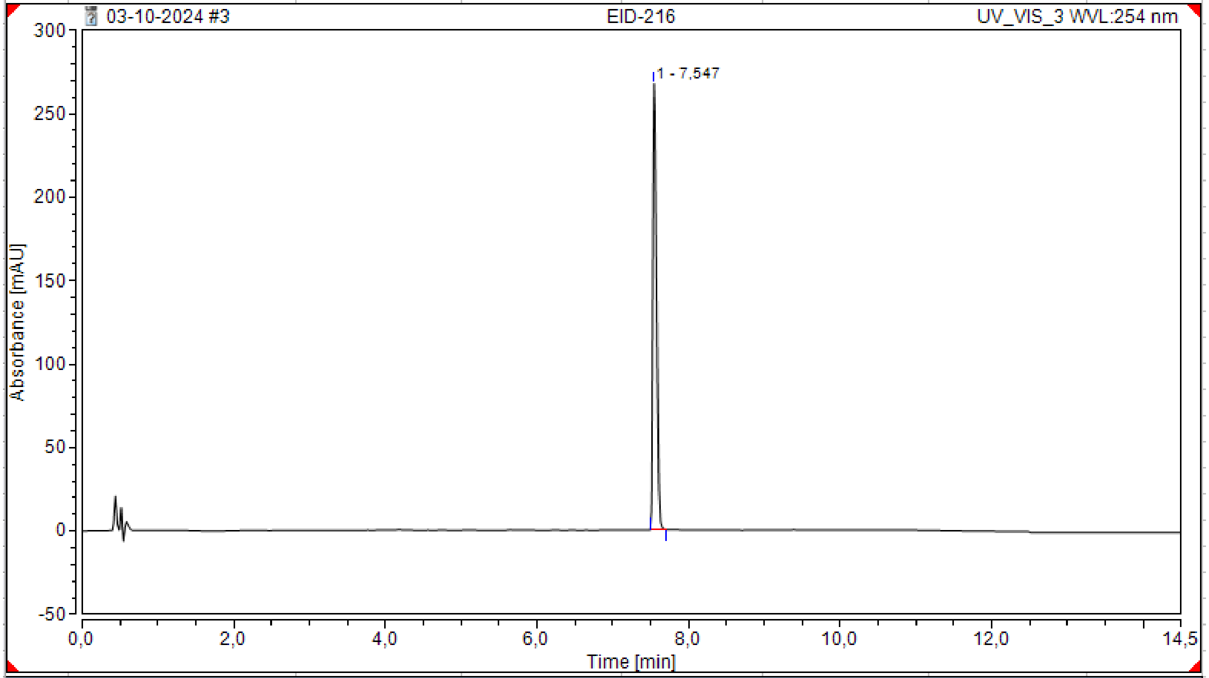

Integration Results

No.	Peak Name	Retention Time min	Area mAU*min	Height mAU	Relative Area %	Relative Height %	Amount n.a.
1		7,547	14,974	267,574	100,00	100,00	n.a.
**Total:**			**14,974**	**267,574**	**100,00**	**100,00**	

Figure S 114. Chromatogram of compound **6a**.

6b

Injection Details

Injection Name:	EID-124		*Run Time (min):*	8,00	
Vial Number:	GE4		*Injection Volume:*	5,00	
Injection Type:	Unknown		*Channel:*	UV_VIS_3	
Calibration Level:			*Wavelength:*	254	
Instrument Method:	Purity_PROTAC_short		*Bandwidth:*	16	
Processing Method:	Quantitative(1)		*Dilution Factor:*	1,0000	
Injection Date/Time:	08.jun..23 13:31		*Sample Weight:*	1,0000	

Chromatogram

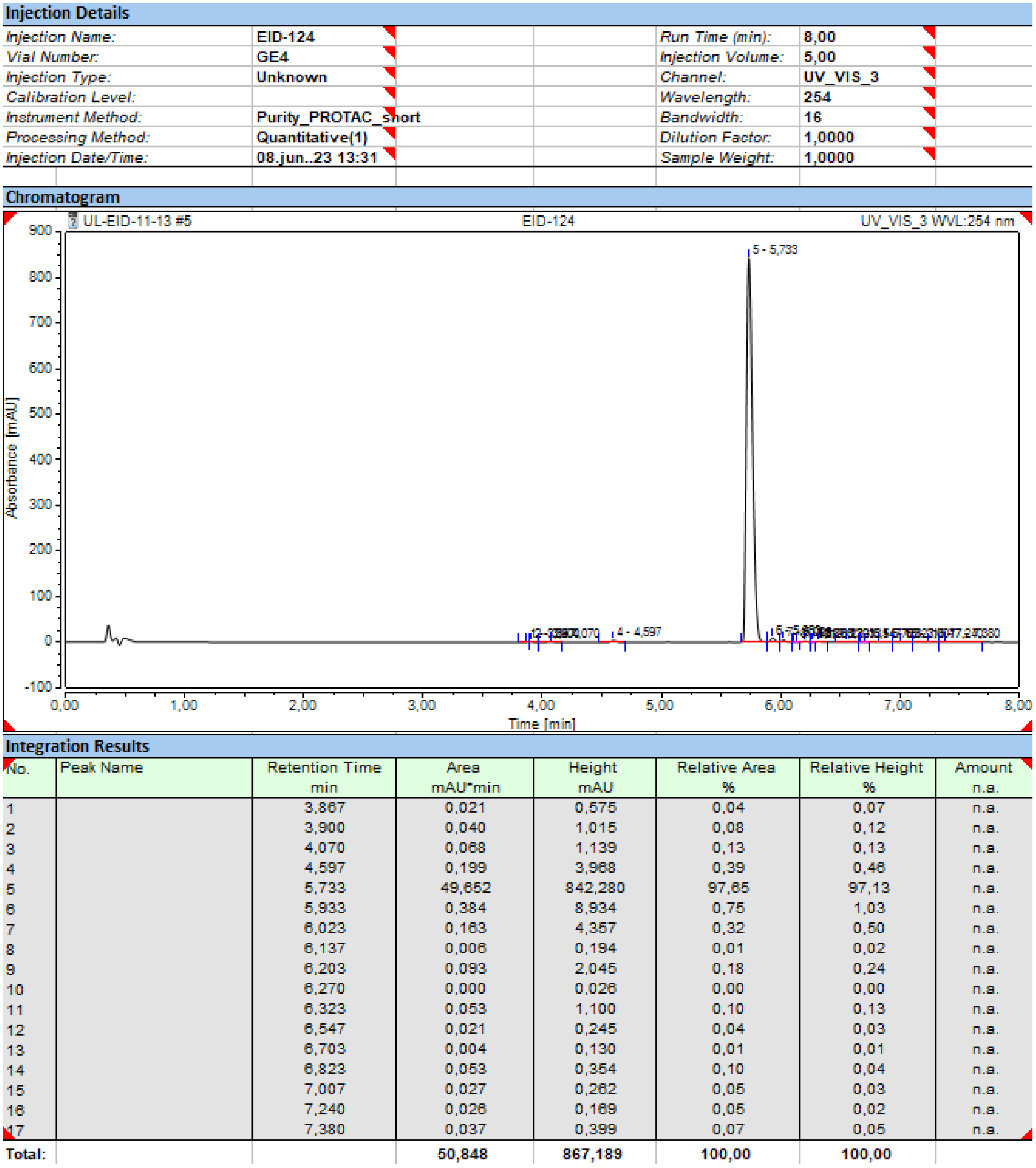

Integration Results

No.	Peak Name	Retention Time min	Area mAU*min	Height mAU	Relative Area %	Relative Height %	Amount n.a.
1		3,867	0,021	0,575	0,04	0,07	n.a.
2		3,900	0,040	1,015	0,08	0,12	n.a.
3		4,070	0,068	1,139	0,13	0,13	n.a.
4		4,597	0,199	3,968	0,39	0,46	n.a.
5		5,733	49,652	842,280	97,65	97,13	n.a.
6		5,933	0,384	8,934	0,75	1,03	n.a.
7		6,023	0,163	4,357	0,32	0,50	n.a.
8		6,137	0,006	0,194	0,01	0,02	n.a.
9		6,203	0,093	2,045	0,18	0,24	n.a.
10		6,270	0,000	0,026	0,00	0,00	n.a.
11		6,323	0,053	1,100	0,10	0,13	n.a.
12		6,547	0,021	0,245	0,04	0,03	n.a.
13		6,703	0,004	0,130	0,01	0,01	n.a.
14		6,823	0,053	0,354	0,10	0,04	n.a.
15		7,007	0,027	0,262	0,05	0,03	n.a.
16		7,240	0,026	0,169	0,05	0,02	n.a.
17		7,380	0,037	0,399	0,07	0,05	n.a.
Total:			50,848	867,189	100,00	100,00	

Figure S 115. Chromatogram of compound **6b**.

6c

Chromatogram and Results

Injection Details

Injection Name:	**EID8**	*Run Time (min):*	**8,00**
Vial Number:	**BA3**	*Injection Volume:*	**5,00**
Injection Type:	**Unknown**	*Channel:*	**UV_VIS_3**
Calibration Level:		*Wavelength:*	**254**
Instrument Method:	**Purity_PROTAC_short**	*Bandwidth:*	**16**
Processing Method:	**Quantitative**	*Dilution Factor:*	**1,0000**
Injection Date/Time:	**16.nov..23 19:57**	*Sample Weight:*	**1,0000**

Chromatogram

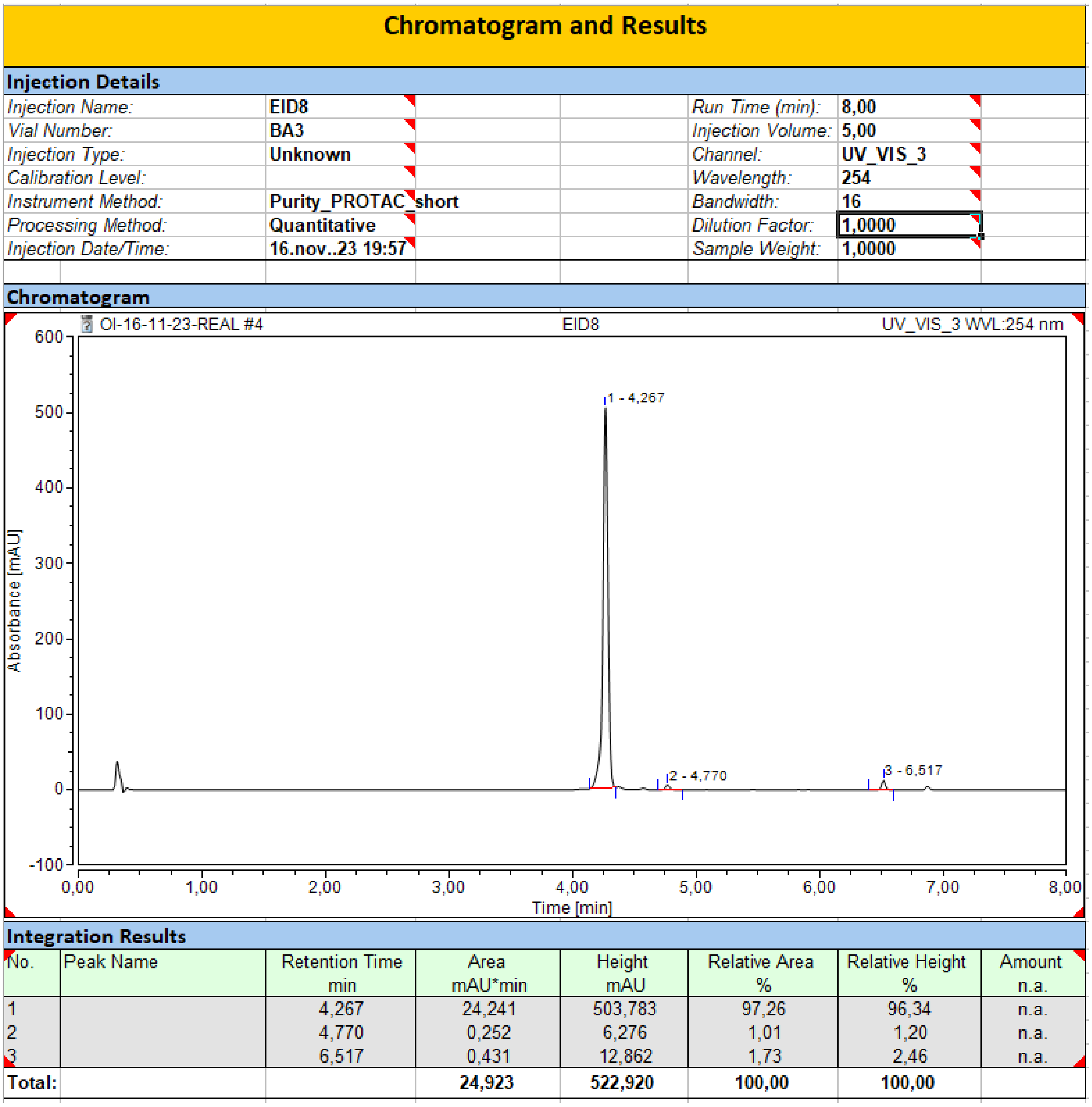

Integration Results

No.	Peak Name	Retention Time min	Area mAU*min	Height mAU	Relative Area %	Relative Height %	Amount n.a.
1		4,267	24,241	503,783	97,26	96,34	n.a.
2		4,770	0,252	6,276	1,01	1,20	n.a.
3		6,517	0,431	12,862	1,73	2,46	n.a.
**Total:**			**24,923**	**522,920**	**100,00**	**100,00**	

Figure S 116. Chromatogram of compound **6c**.

6d

Injection Name:	175		Run Time (min):	8,00
Vial Number:	RB8		Injection Volume:	5,00
Injection Type:	Unknown		Channel:	UV_VIS_3
Calibration Level:			Wavelength:	254
Instrument Method:	Purity_PROTAC_short		Bandwidth:	16
Processing Method:	Quantitative		Dilution Factor:	1,0000
Injection Date/Time:	22.avg..23 21:19		Sample Weight:	1,0000

Chromatogram

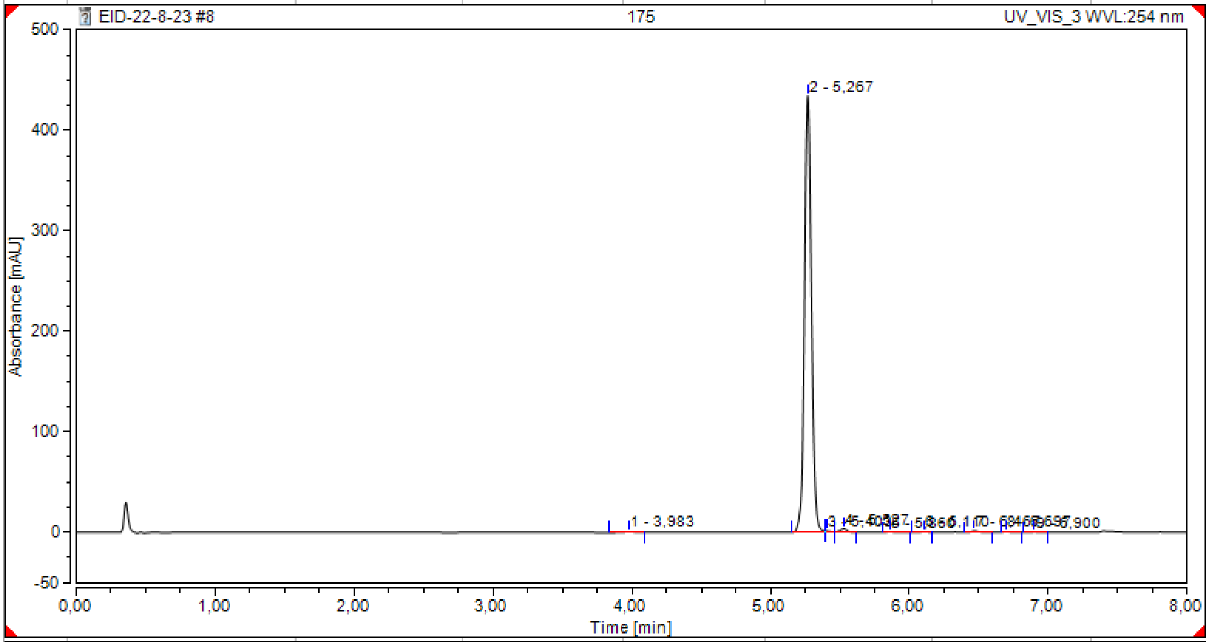

Integration Results

No.	Peak Name	Retention Time min	Area mAU*min	Height mAU	Relative Area %	Relative Height %	Amount n.a.
1		3,983	0,062	0,808	0,25	0,18	n.a.
2		5,267	24,527	434,450	97,99	98,01	n.a.
3		5,403	0,060	1,302	0,24	0,29	n.a.
4		5,527	0,209	3,581	0,84	0,81	n.a.
5		5,860	0,025	0,356	0,10	0,08	n.a.
6		6,110	0,030	0,567	0,12	0,13	n.a.
7		6,467	0,073	1,379	0,29	0,31	n.a.
8		6,697	0,022	0,492	0,09	0,11	n.a.
9		6,900	0,022	0,323	0,09	0,07	n.a.
**Total:**			**25,029**	**443,257**	**100,00**	**100,00**	

Figure S 117. Chromatogram of compound **6d**.

6e

Injection Details

Injection Name:	**EID - 115 orisit**	*Run Time (min):*	**8,00**
Vial Number:	**RB3**	*Injection Volume:*	**5,00**
Injection Type:	**Unknown**	*Channel:*	**UV_VIS_3**
Calibration Level:		*Wavelength:*	**254**
Instrument Method:	**Purity_PROTAC_short**	*Bandwidth:*	**16**
Processing Method:	**Quantitative**	*Dilution Factor:*	**1,0000**
Injection Date/Time:	**09.maj.23 12:15**	*Sample Weight:*	**1,0000**

Chromatogram

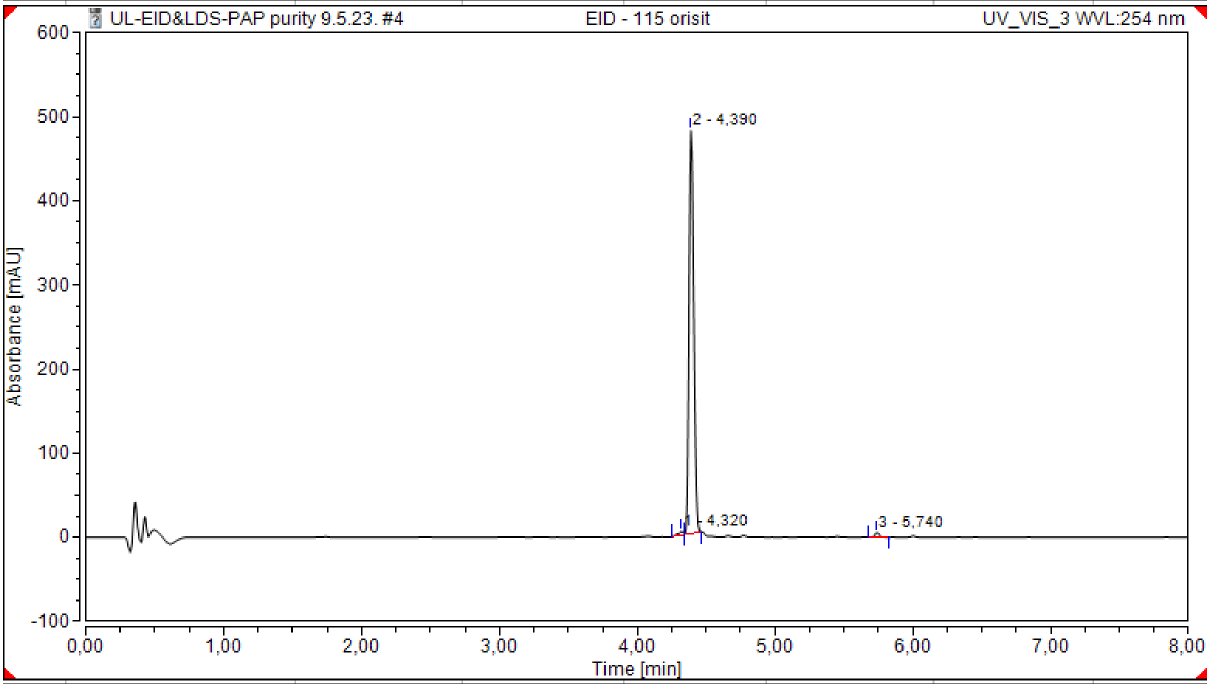

Integration Results

No.	Peak Name	Retention Time min	Area mAU*min	Height mAU	Relative Area %	Relative Height %	Amount n.a.
1		4,320	0,198	4,676	0,98	0,95	n.a.
2		4,390	19,721	479,430	98,03	97,88	n.a.
3		5,740	0,199	5,694	0,99	1,16	n.a.
**Total:**			**20,118**	**489,800**	**100,00**	**100,00**	

Figure S 118. Chromatogram of compound **6e**.

6f

Chromatogram and Results

Injection Details

Injection Name:	**EID10**	*Run Time (min):*	**8,00**
Vial Number:	**BA5**	*Injection Volume:*	**5,00**
Injection Type:	**Unknown**	*Channel:*	**UV_VIS_3**
Calibration Level:		*Wavelength:*	**254**
Instrument Method:	**Purity_PROTAC_short**	*Bandwidth:*	**16**
Processing Method:	**Quantitative**	*Dilution Factor:*	**1,0000**
Injection Date/Time:	**16.nov..23 20:21**	*Sample Weight:*	**1,0000**

Chromatogram

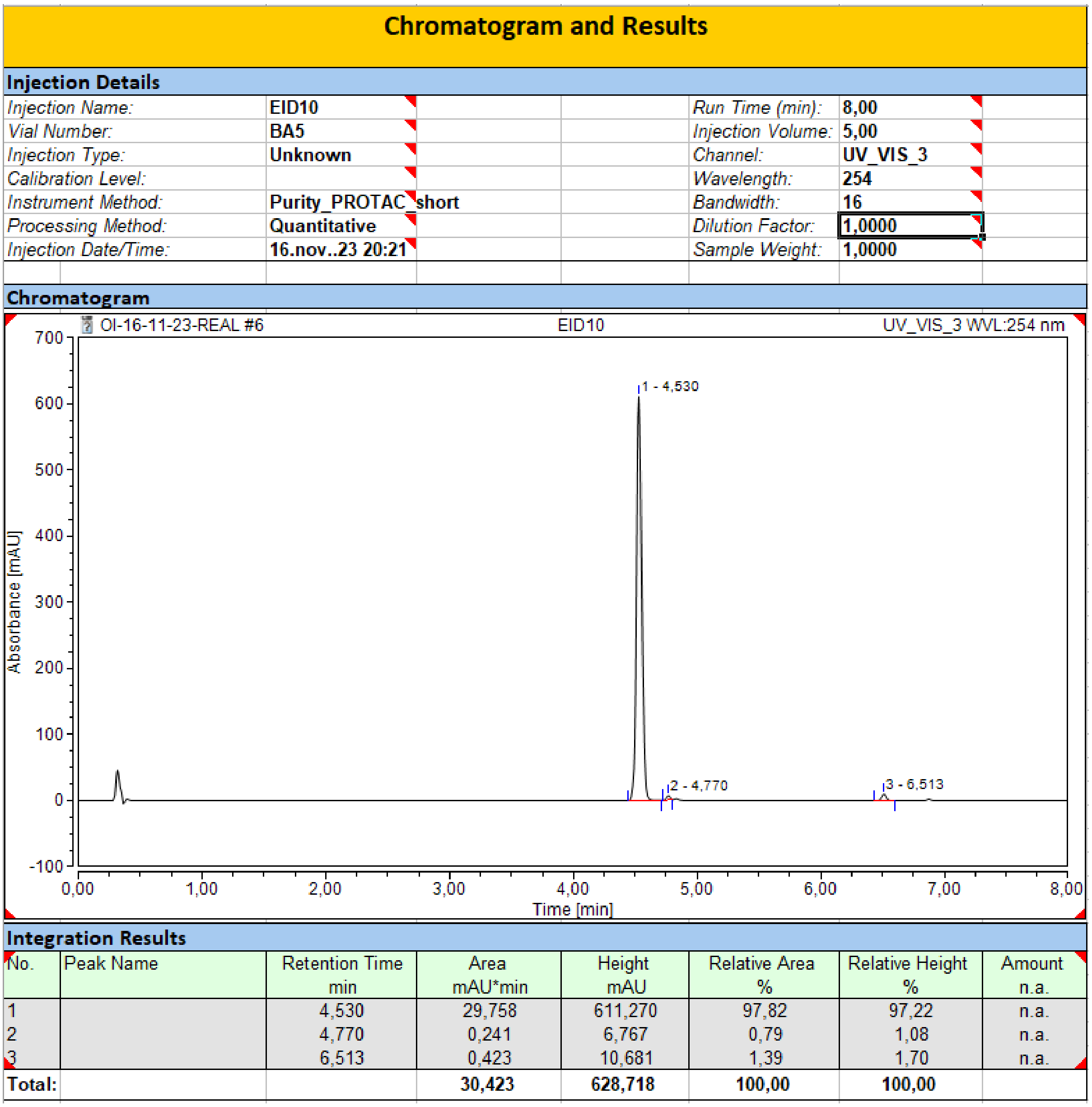

Integration Results

No.	Peak Name	Retention Time min	Area mAU*min	Height mAU	Relative Area %	Relative Height %	Amount n.a.
1		4,530	29,758	611,270	97,82	97,22	n.a.
2		4,770	0,241	6,767	0,79	1,08	n.a.
3		6,513	0,423	10,681	1,39	1,70	n.a.
**Total:**			**30,423**	**628,718**	**100,00**	**100,00**	

Figure S 119. Chromatogram of compound **6f**.

8a

Chromatogram and Results

Injection Details

Injection Name:	**EID-325**	*Run Time (min):*	**14,50**
Vial Number:	**RC7**	*Injection Volume:*	**1,00**
Injection Type:	**Unknown**	*Channel:*	**UV_VIS_3**
Calibration Level:		*Wavelength:*	**254**
Instrument Method:	**Purity_PROTAC**	*Bandwidth:*	**16**
Processing Method:	**Quantitative**	*Dilution Factor:*	**1,0000**
Injection Date/Time:	**03.okt..24 14:34**	*Sample Weight:*	**1,0000**

Chromatogram

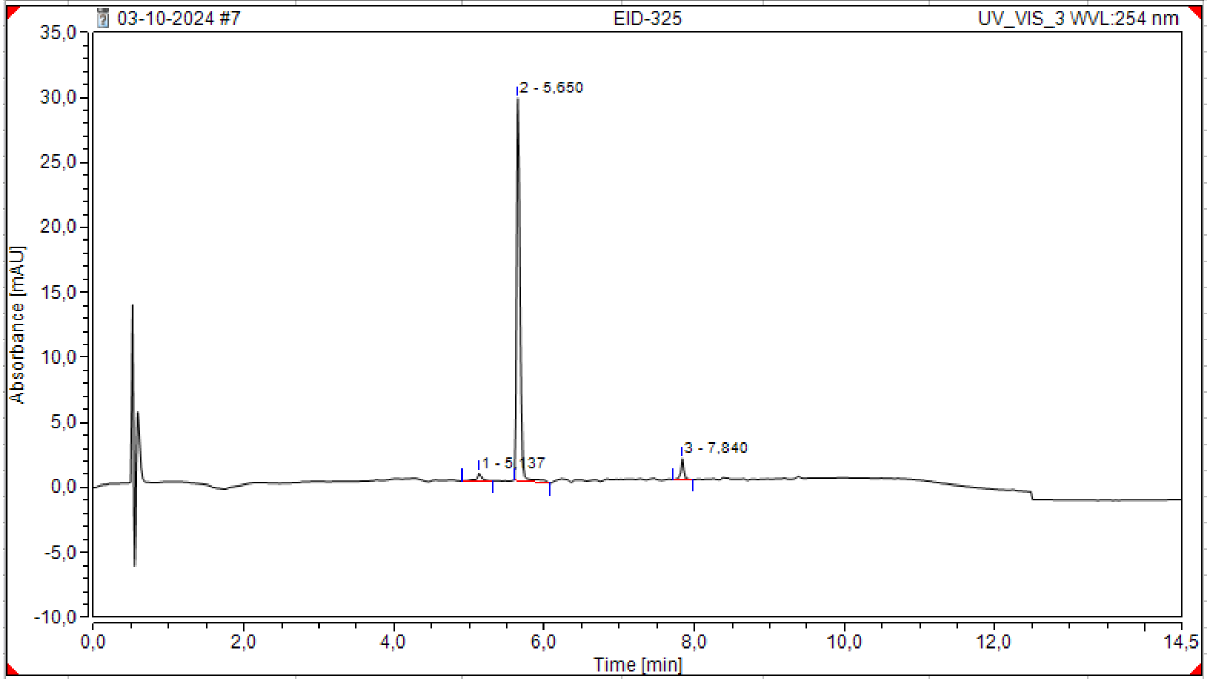

Integration Results

No.	Peak Name	Retention Time min	Area mAU*min	Height mAU	Relative Area %	Relative Height %	Amount n.a.
1		5,137	0,048	0,607	2,68	1,92	n.a.
2		5,650	1,659	29,406	92,99	92,98	n.a.
3		7,840	0,077	1,613	4,33	5,10	n.a.
**Total:**			**1,784**	**31,626**	**100,00**	**100,00**	

Figure S 120. Chromatogram of compound **8a**.

8b

Chromatogram and Results

Injection Details

Injection Name:	**EID-332**	*Run Time (min):*	**14,50**
Vial Number:	**RC8**	*Injection Volume:*	**1,00**
Injection Type:	**Unknown**	*Channel:*	**UV_VIS_3**
Calibration Level:		*Wavelength:*	**254**
Instrument Method:	**Purity_PROTAC**	*Bandwidth:*	**16**
Processing Method:	**Quantitative**	*Dilution Factor:*	**1,0000**
Injection Date/Time:	**03.okt..24 14:53**	*Sample Weight:*	**1,0000**

Chromatogram

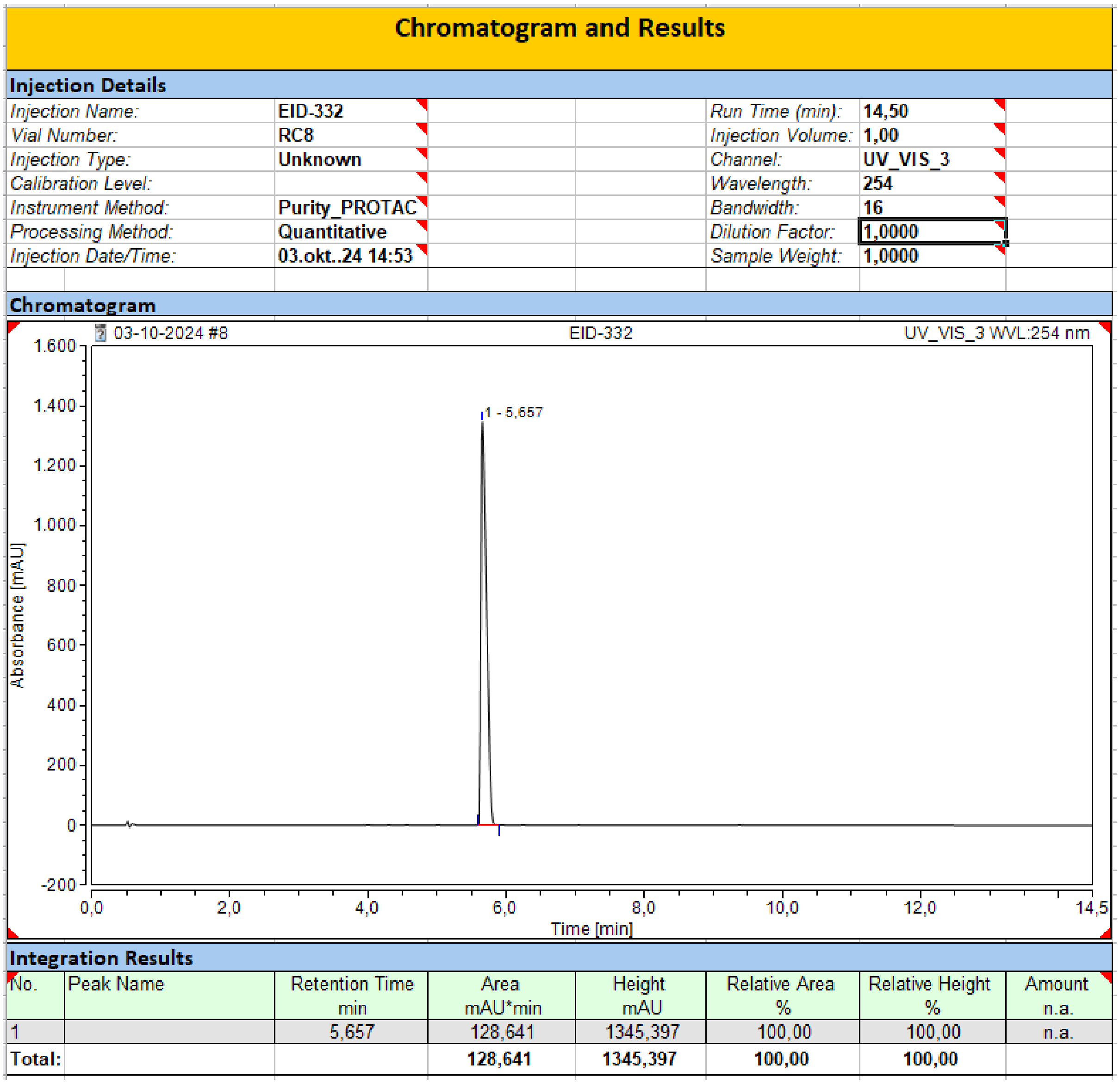

Integration Results

No.	Peak Name	Retention Time min	Area mAU*min	Height mAU	Relative Area %	Relative Height %	Amount n.a.
1		5,657	128,641	1345,397	100,00	100,00	n.a.
**Total:**			**128,641**	**1345,397**	**100,00**	**100,00**	

Figure S 121. Chromatogram of compound **8b**.

9a

Chromatogram and Results

Injection Details

Injection Name:	**EID-343**	*Run Time (min):*	**14,50**
Vial Number:	**BA7**	*Injection Volume:*	**0,20**
Injection Type:	**Unknown**	*Channel:*	**UV_VIS_3**
Calibration Level:		*Wavelength:*	**254**
Instrument Method:	**Purity_PROTAC**	*Bandwidth:*	**16**
Processing Method:	**Quantitative**	*Dilution Factor:*	**1,0000**
Injection Date/Time:	**29.okt..24 19:05**	*Sample Weight:*	**1,0000**

Chromatogram

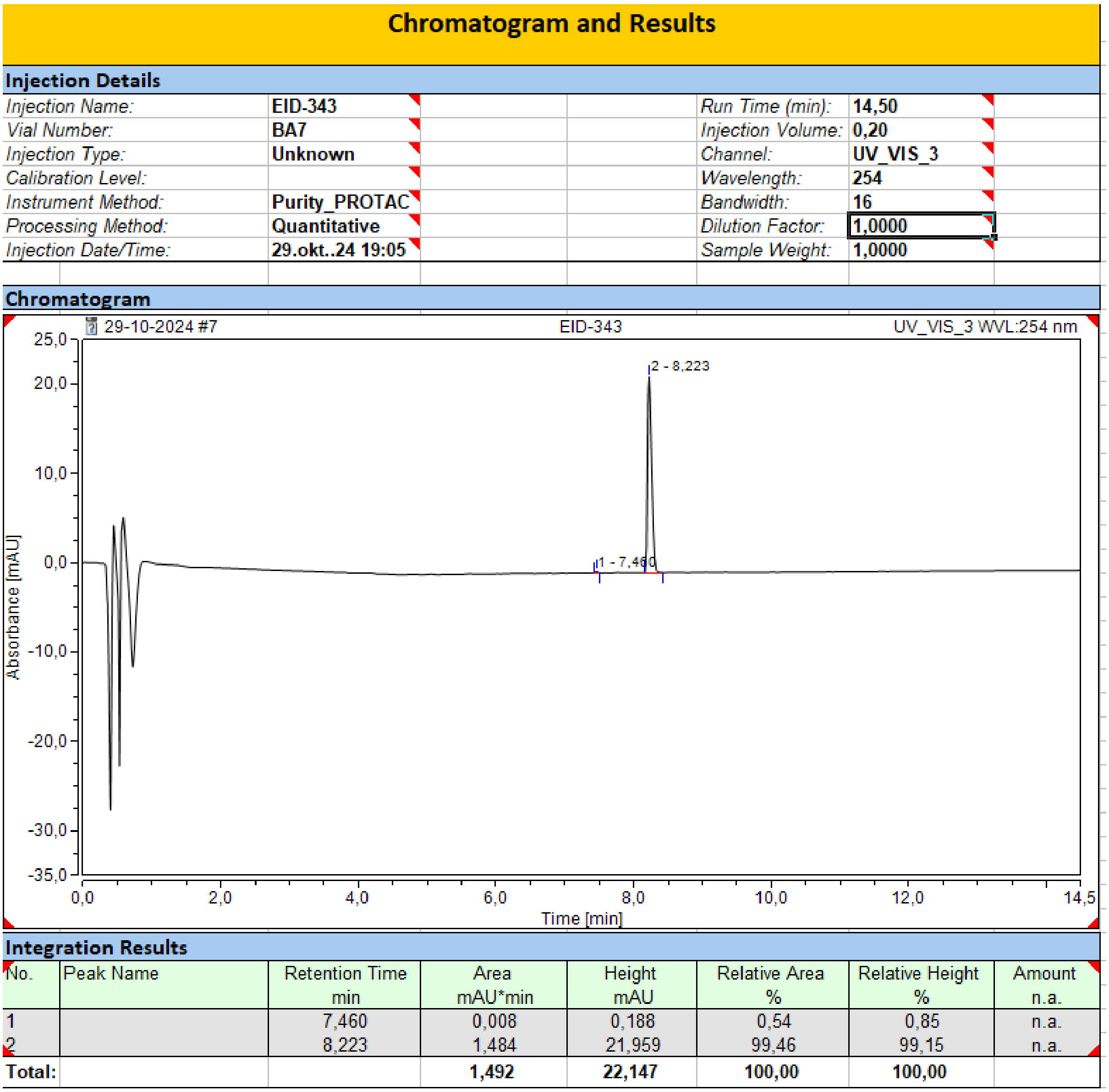

Integration Results

No.	Peak Name	Retention Time min	Area mAU*min	Height mAU	Relative Area %	Relative Height %	Amount n.a.
1		7,460	0,008	0,188	0,54	0,85	n.a.
2		8,223	1,484	21,959	99,46	99,15	n.a.
**Total:**			**1,492**	**22,147**	**100,00**	**100,00**	

Figure S 122. Chromatogram of compound **9a**.

9b

Chromatogram and Results

Injection Details

Injection Name:	**EID-342**	*Run Time (min):*	**14,50**
Vial Number:	**BA6**	*Injection Volume:*	**1,00**
Injection Type:	**Unknown**	*Channel:*	**UV_VIS_3**
Calibration Level:		*Wavelength:*	**254**
Instrument Method:	**Purity_PROTAC**	*Bandwidth:*	**16**
Processing Method:	**Quantitative**	*Dilution Factor:*	**1,0000**
Injection Date/Time:	**29.okt..24 18:46**	*Sample Weight:*	**1,0000**

Chromatogram

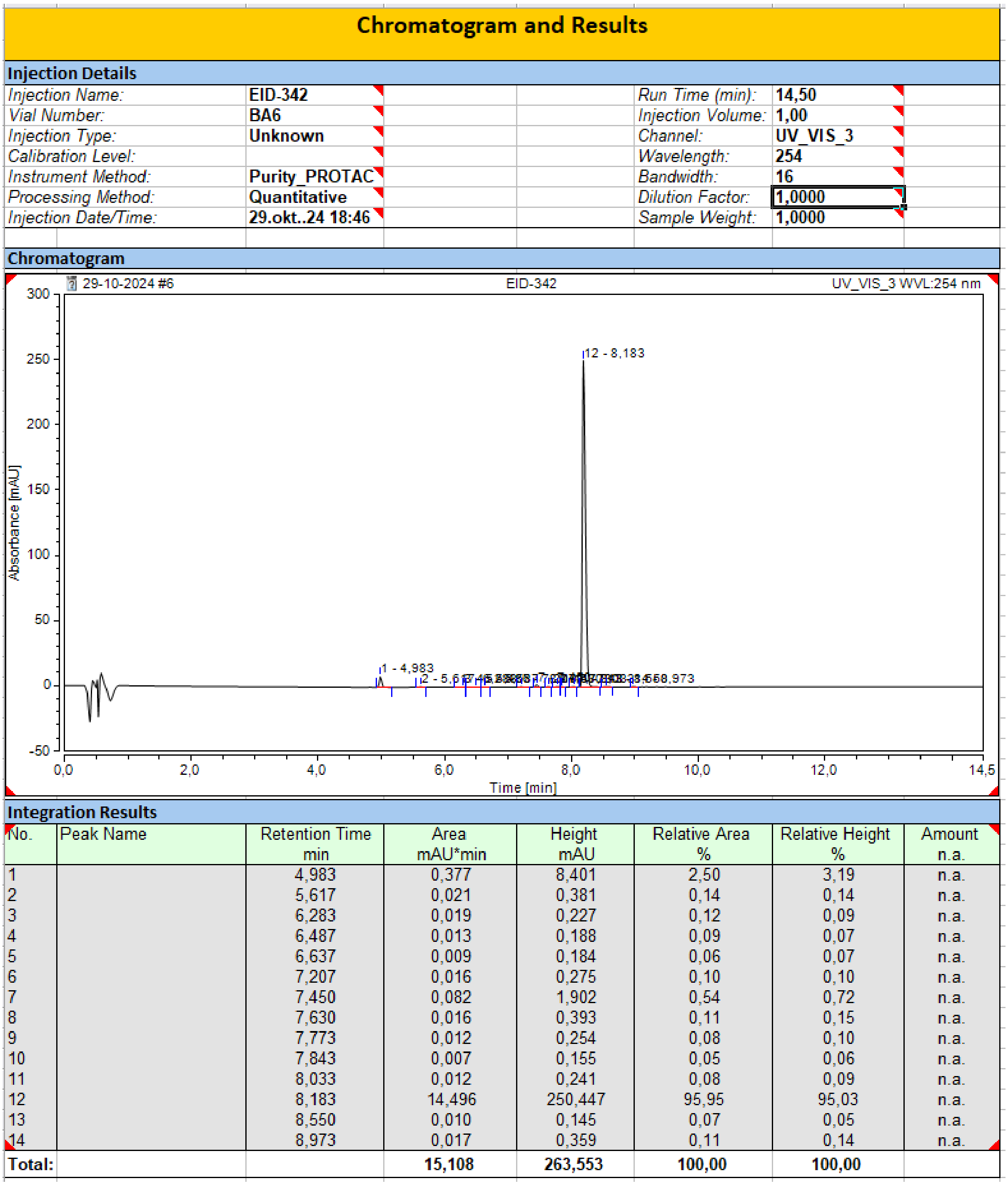

Integration Results

No.	Peak Name	Retention Time min	Area mAU*min	Height mAU	Relative Area %	Relative Height %	Amount n.a.
1		4,983	0,377	8,401	2,50	3,19	n.a.
2		5,617	0,021	0,381	0,14	0,14	n.a.
3		6,283	0,019	0,227	0,12	0,09	n.a.
4		6,487	0,013	0,188	0,09	0,07	n.a.
5		6,637	0,009	0,184	0,06	0,07	n.a.
6		7,207	0,016	0,275	0,10	0,10	n.a.
7		7,450	0,082	1,902	0,54	0,72	n.a.
8		7,630	0,016	0,393	0,11	0,15	n.a.
9		7,773	0,012	0,254	0,08	0,10	n.a.
10		7,843	0,007	0,155	0,05	0,06	n.a.
11		8,033	0,012	0,241	0,08	0,09	n.a.
12		8,183	14,496	250,447	95,95	95,03	n.a.
13		8,550	0,010	0,145	0,07	0,05	n.a.
14		8,973	0,017	0,359	0,11	0,14	n.a.
**Total:**			**15,108**	**263,553**	**100,00**	**100,00**	

Figure S 123. Chromatogram of compound **9b**.

HRMS spectra (Figures S124–S144)

1a

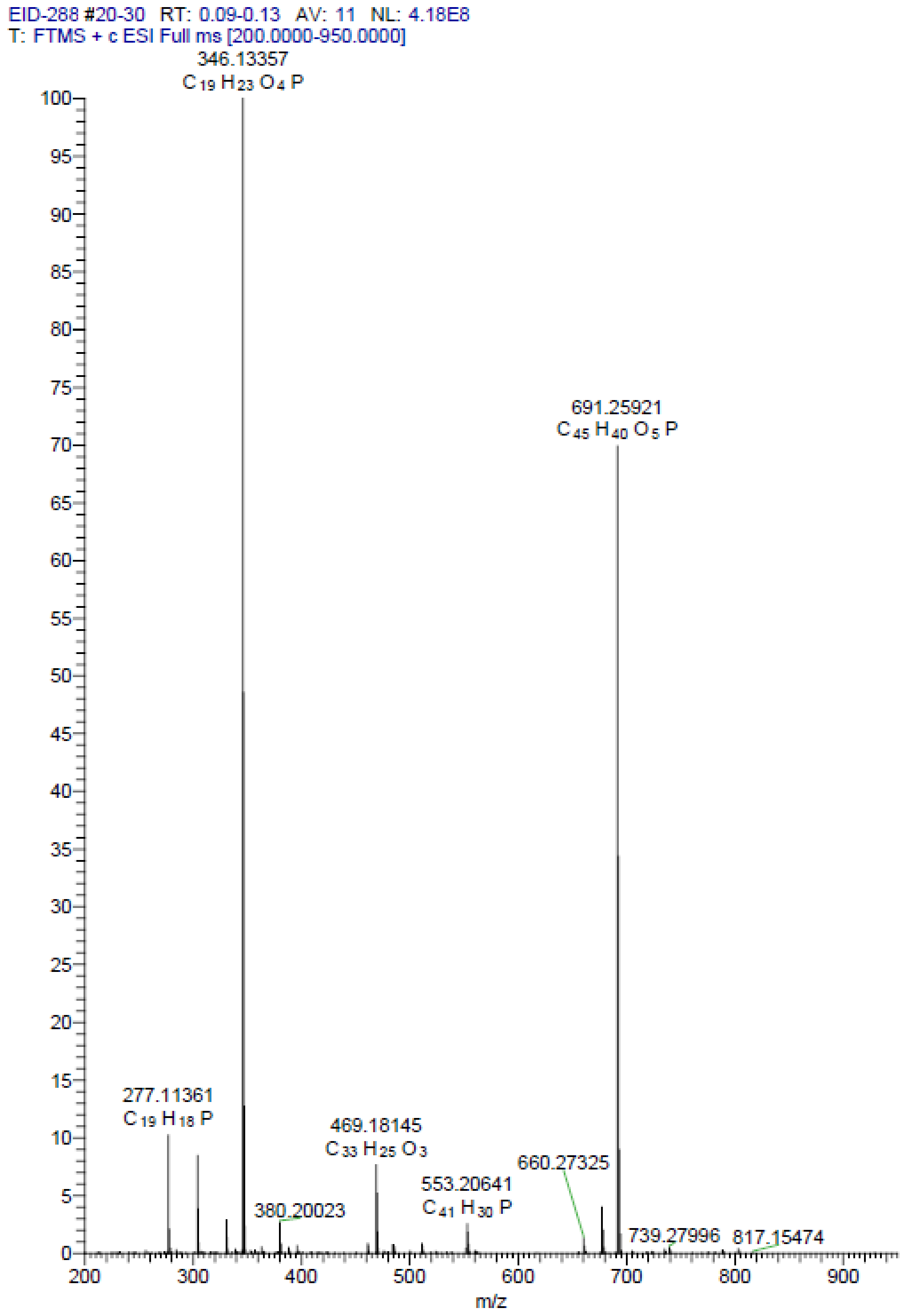

Figure S 124. HRMS spectrum of compound **1a**.

1b

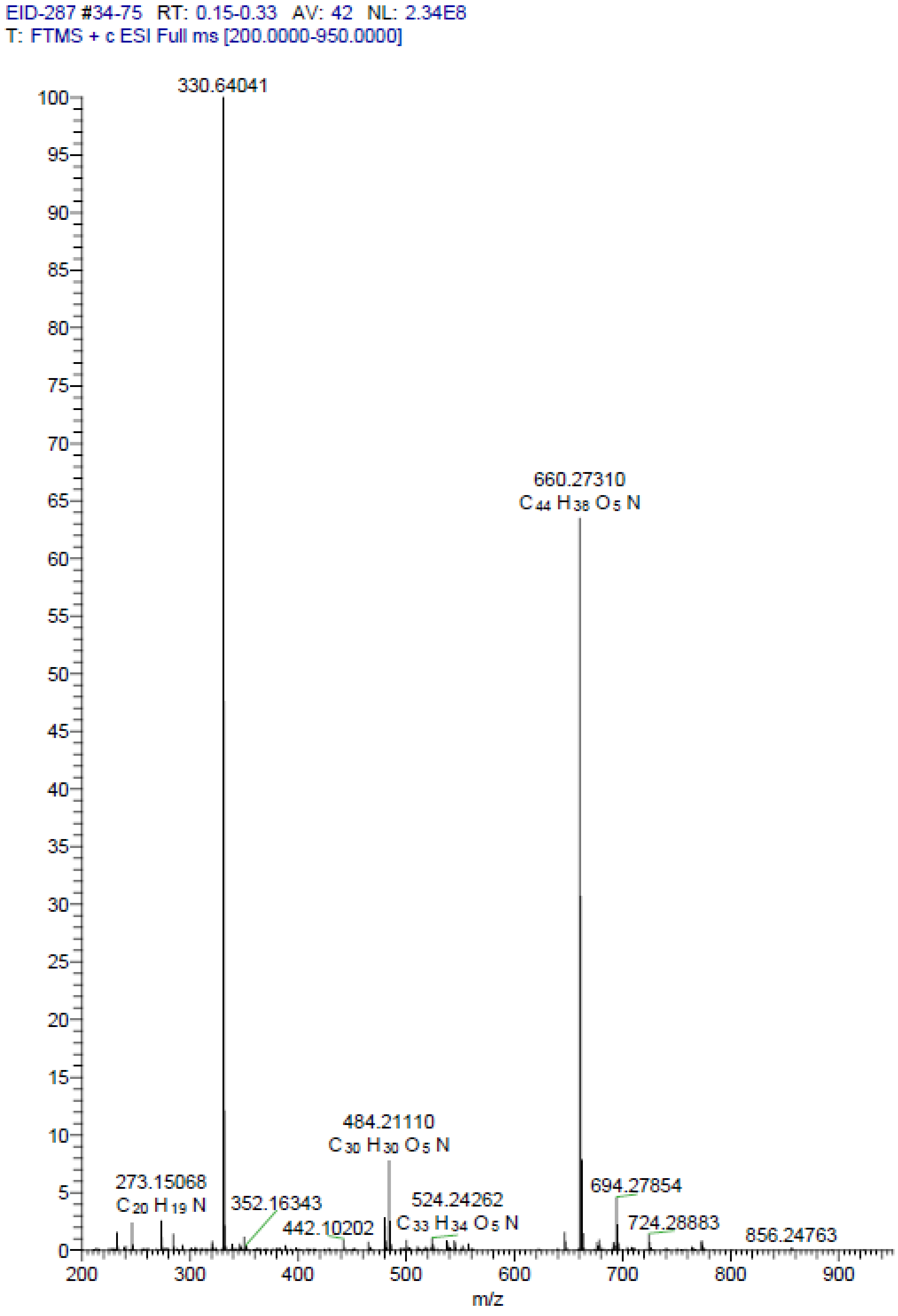

Figure S 125. HRMS spectrum of compound **1b**.

2

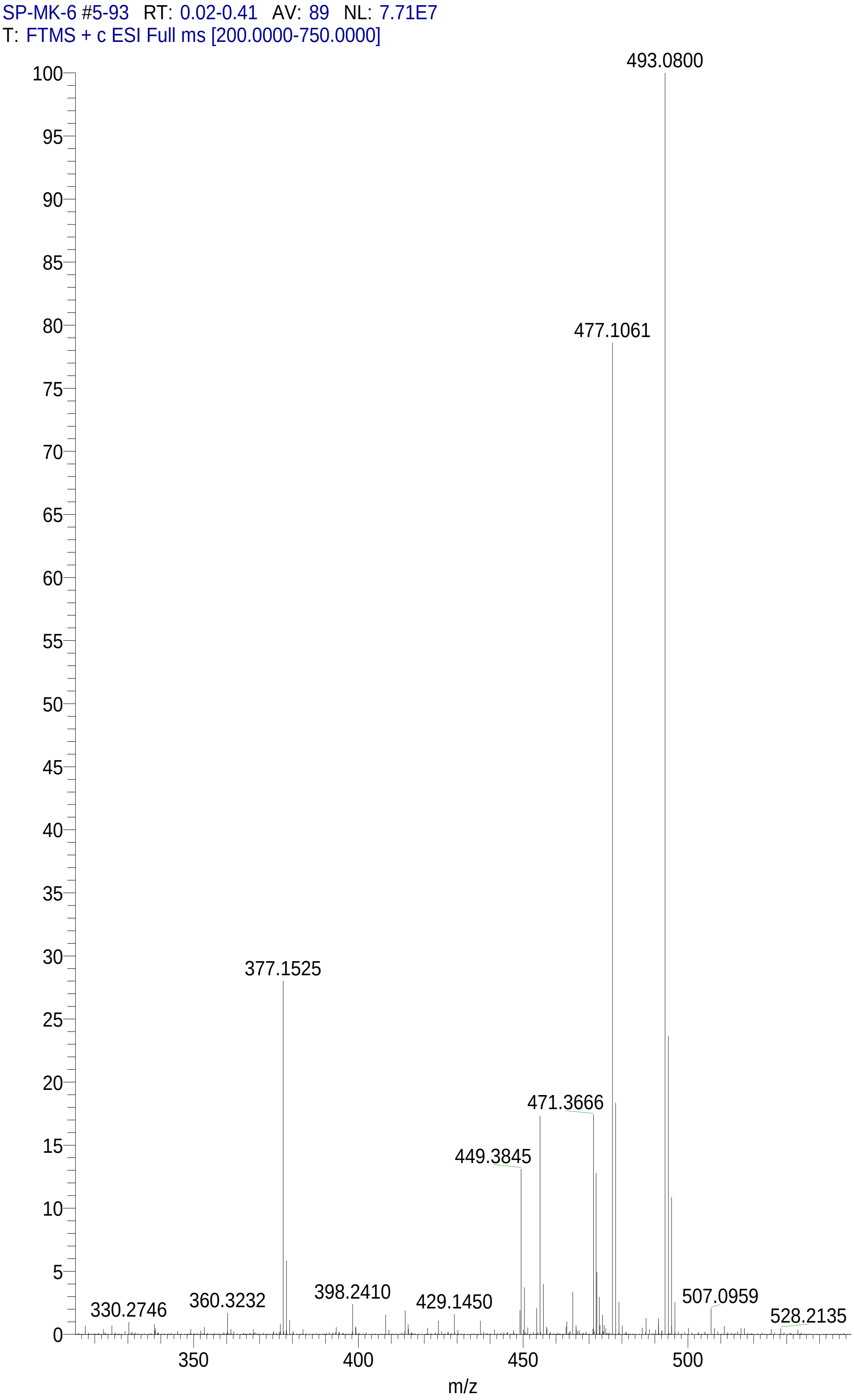

Figure S 126. HRMS spectrum of compound **2**.

3

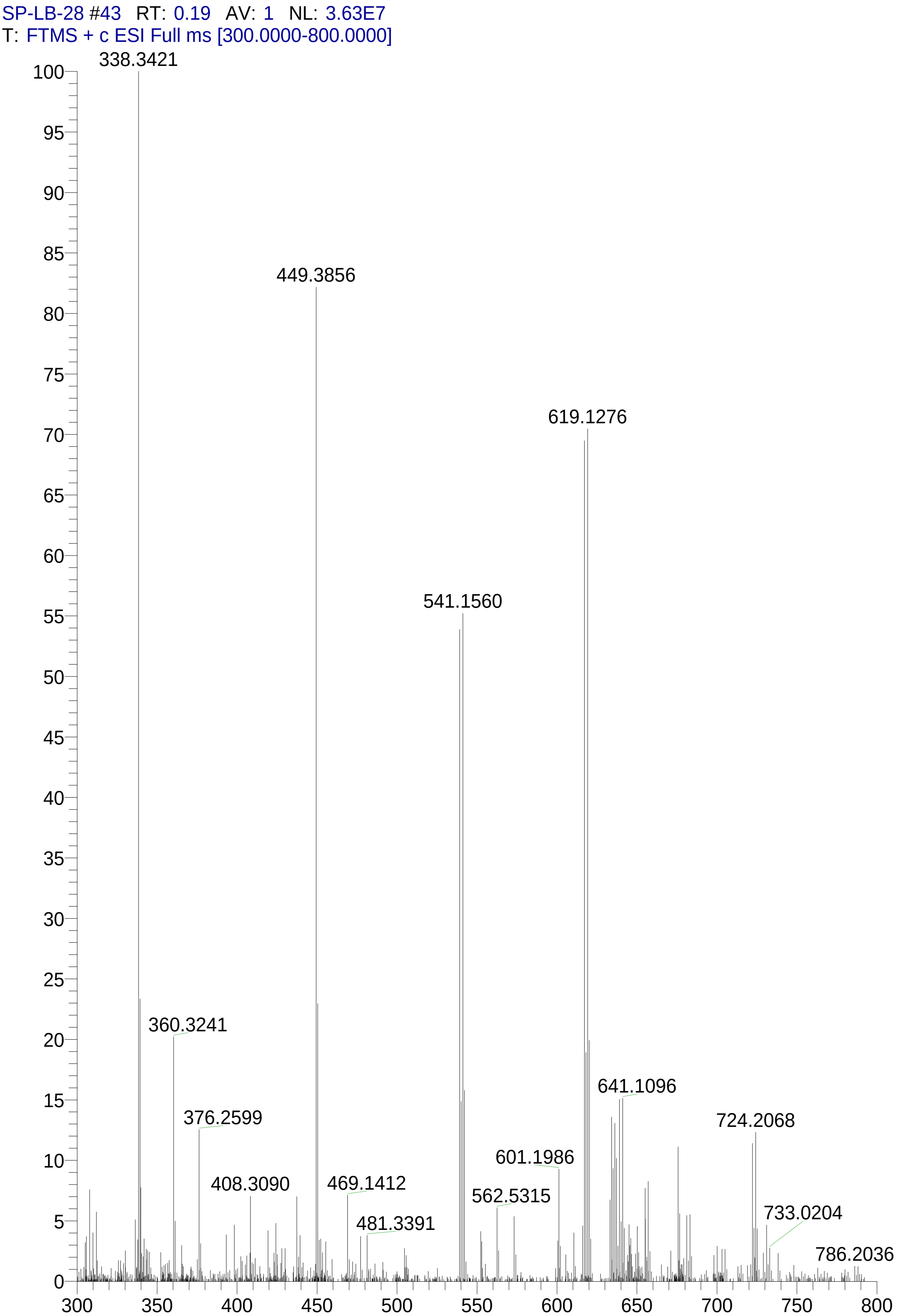

Figure S 127. HRMS spectrum of compound **3**.

4a

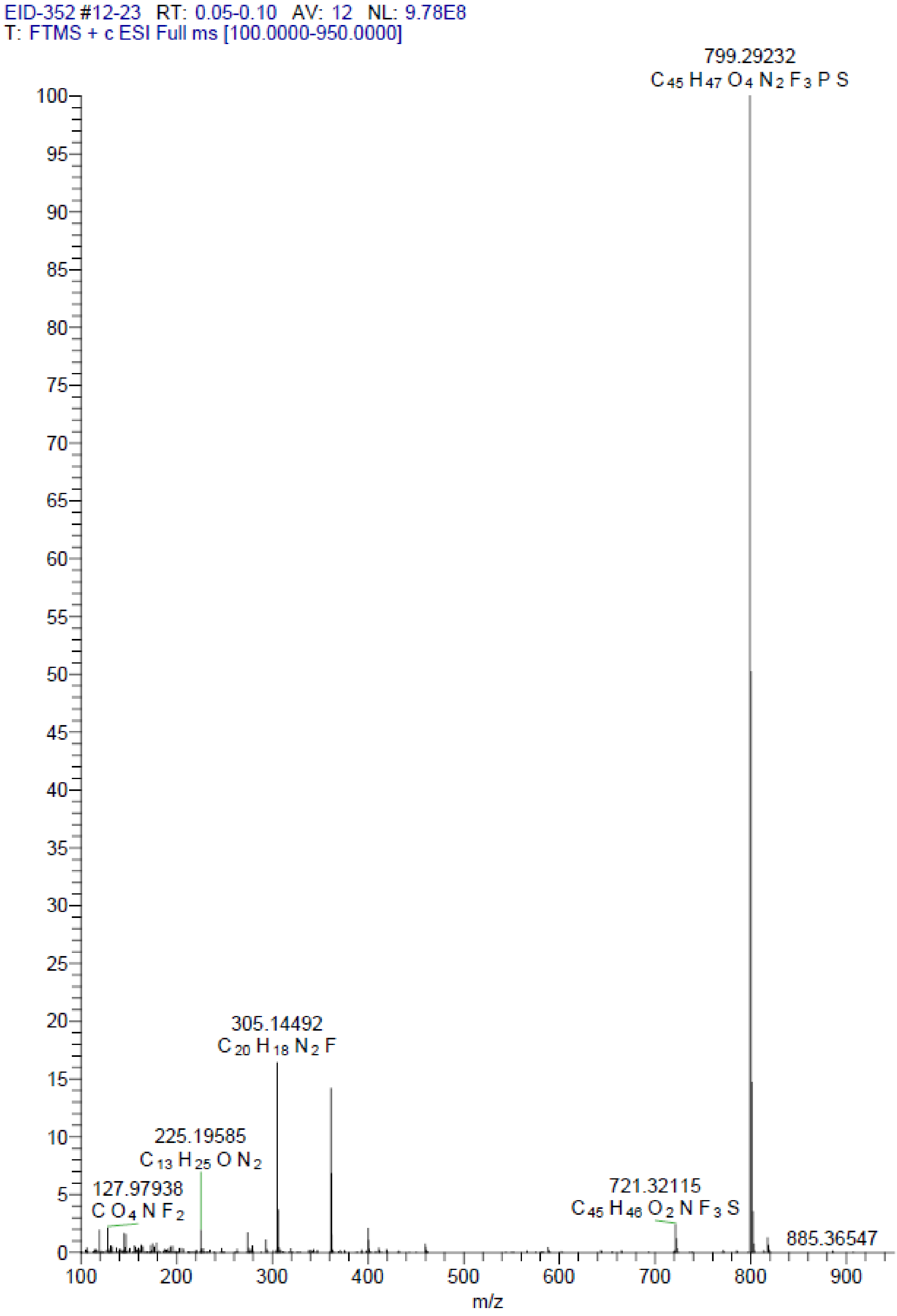

Figure S 128. HRMS spectrum of compound **4a**.

4b

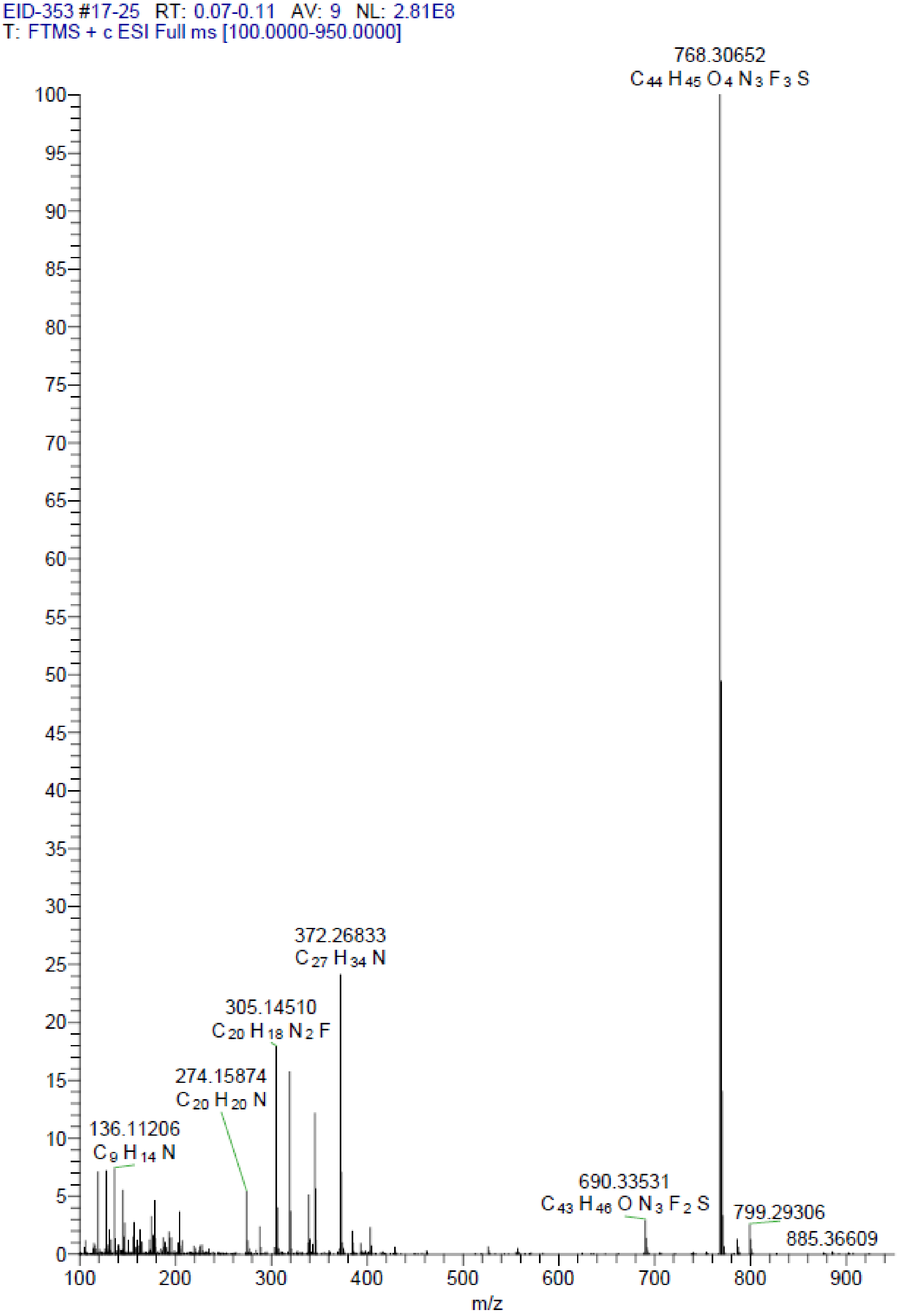

Figure S 129. HRMS spectrum of compound **4b**.

4c

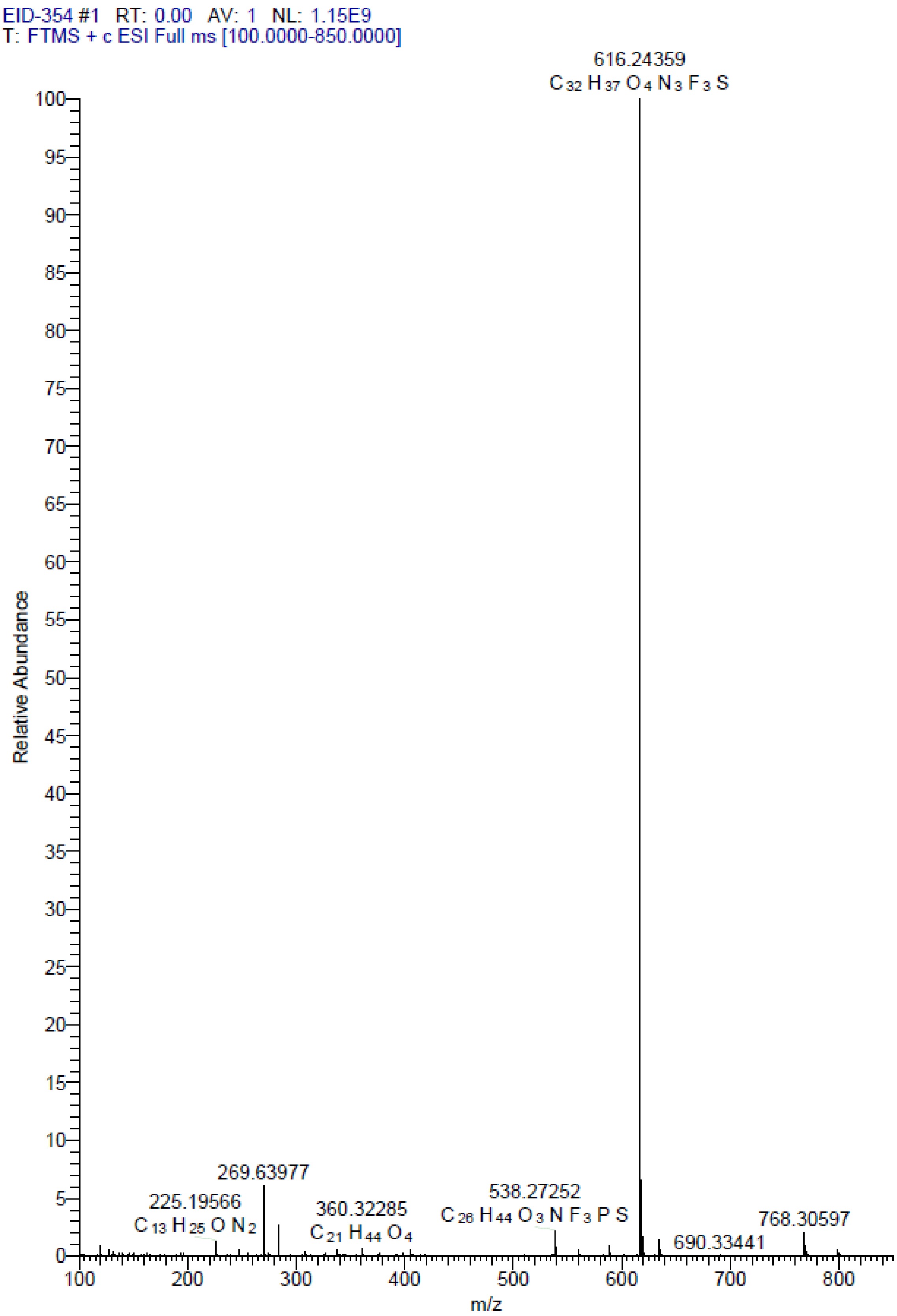

Figure S 130. HRMS spectrum of compound **4c.**

4d

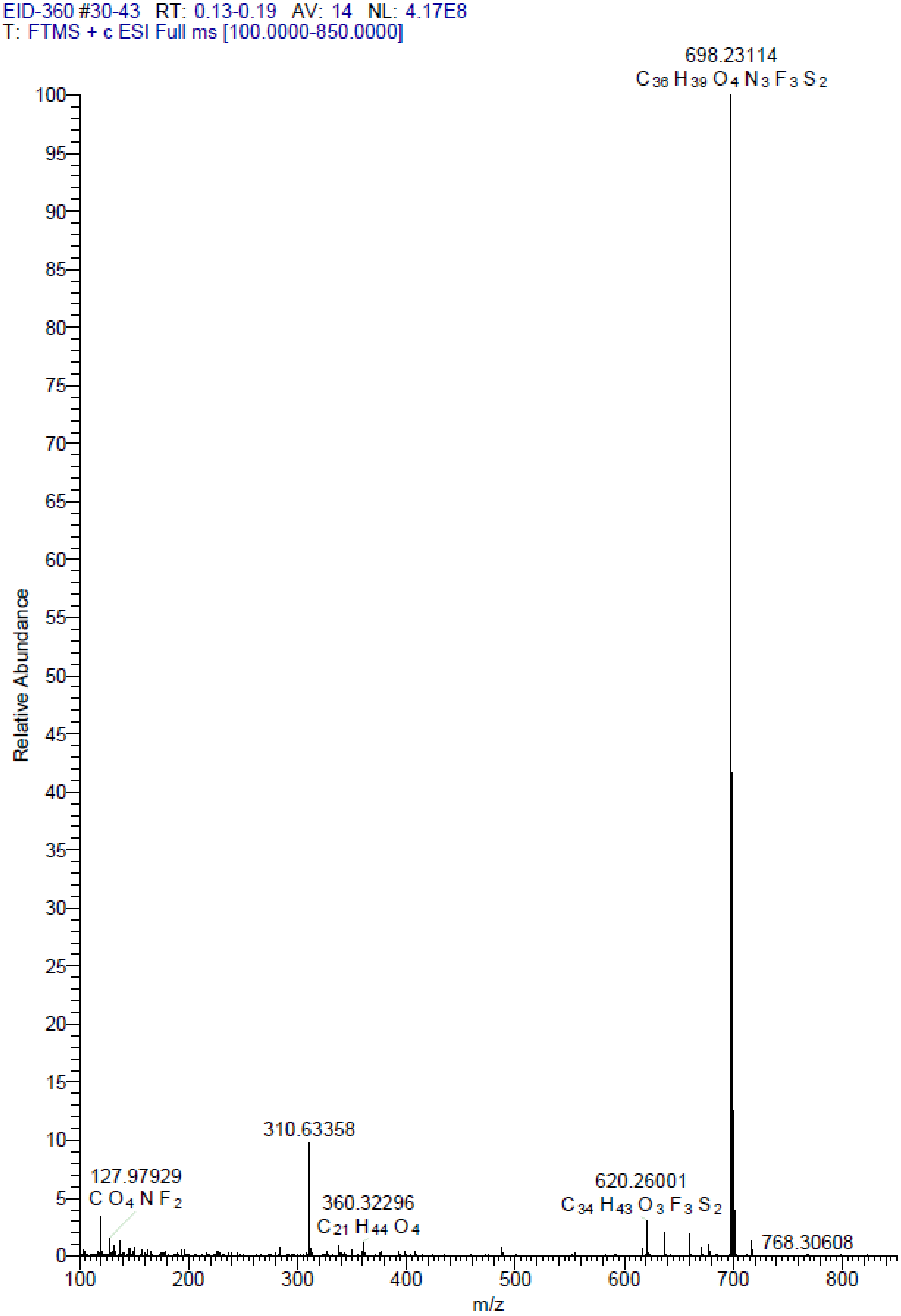

Figure S 131. HRMS spectrum of compound **4d**.

4e

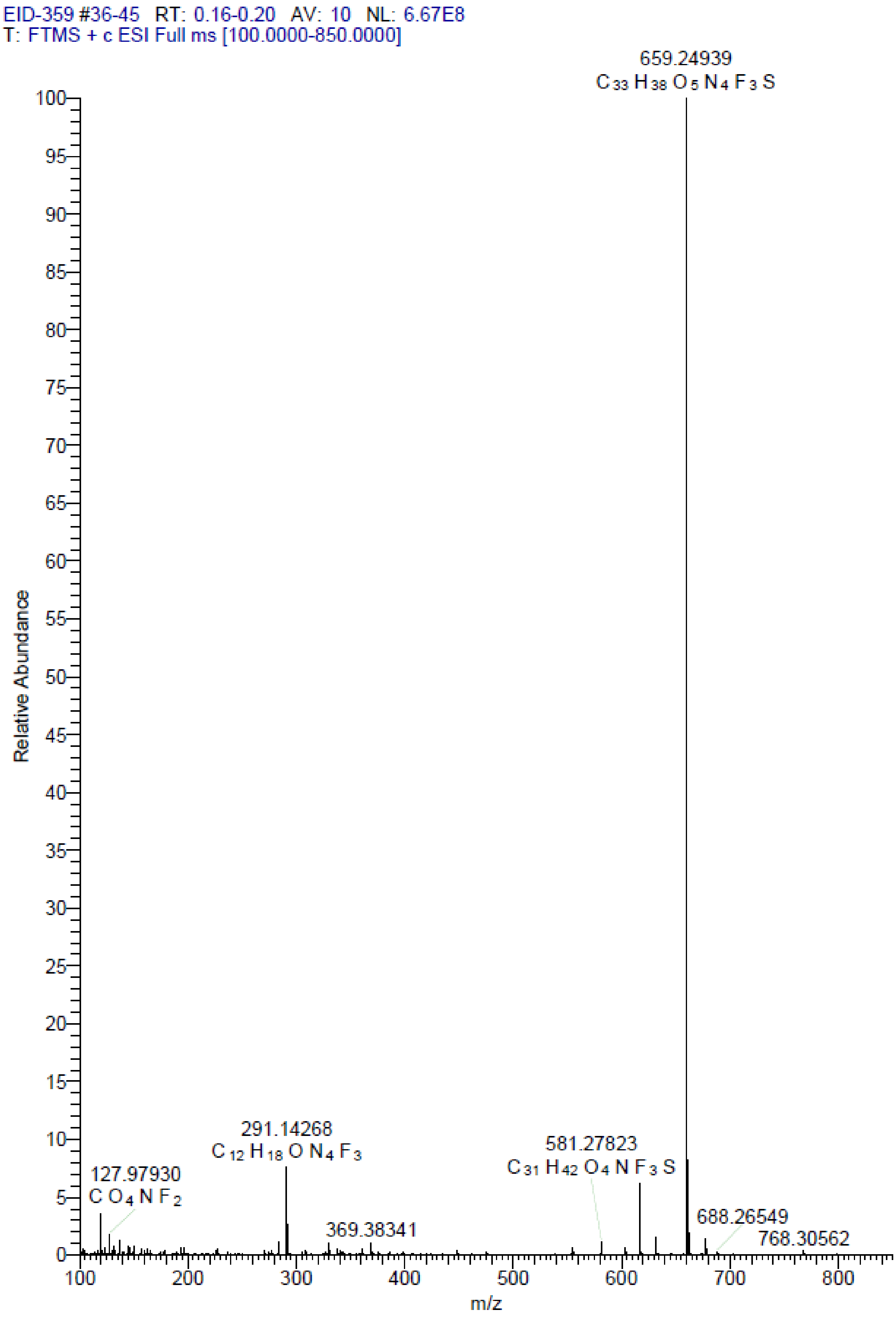

Figure S 132. HRMS spectrum of compound **4e**.

4f

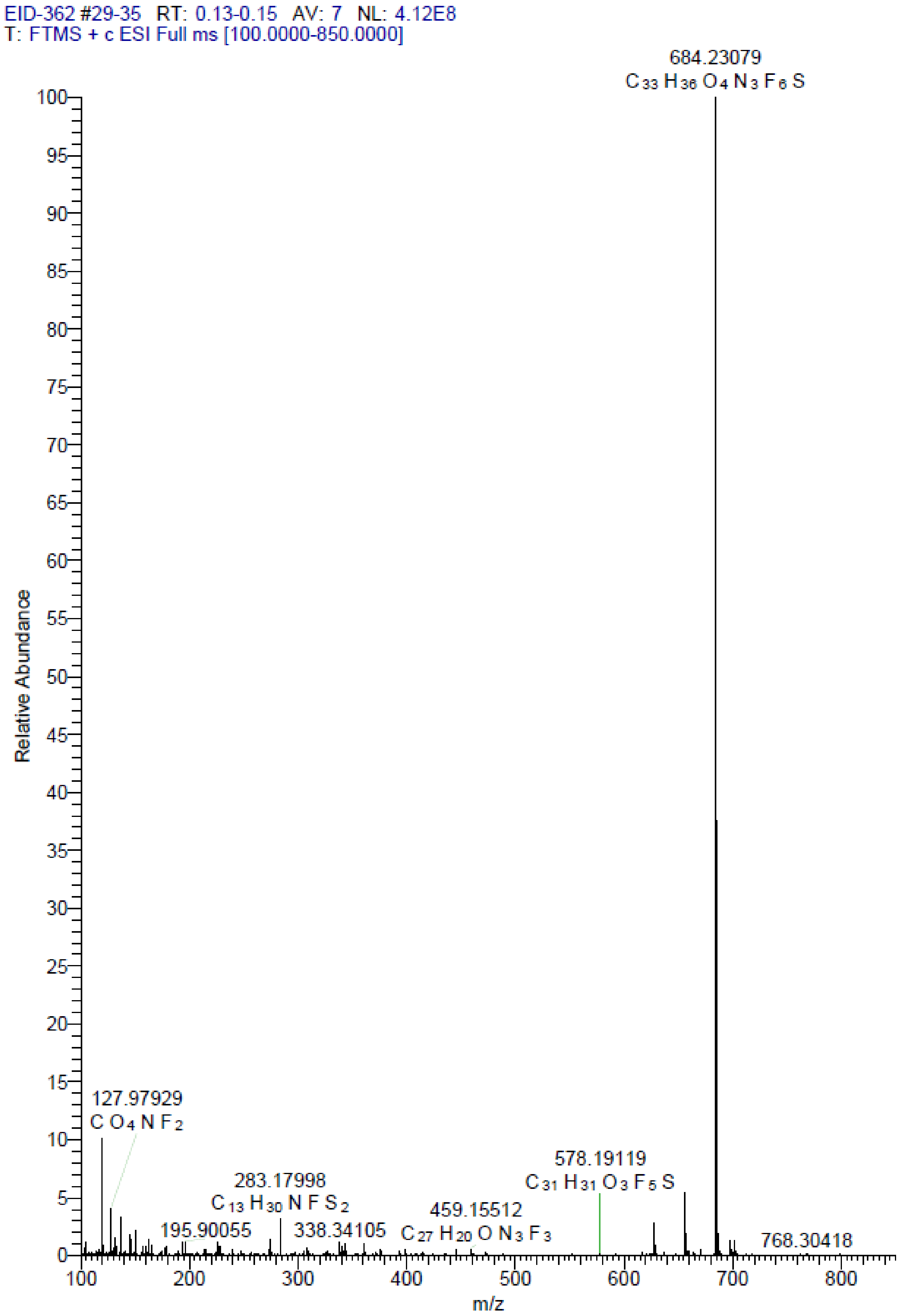

Figure S 133. HRMS spectrum of compound **4f**.

6a

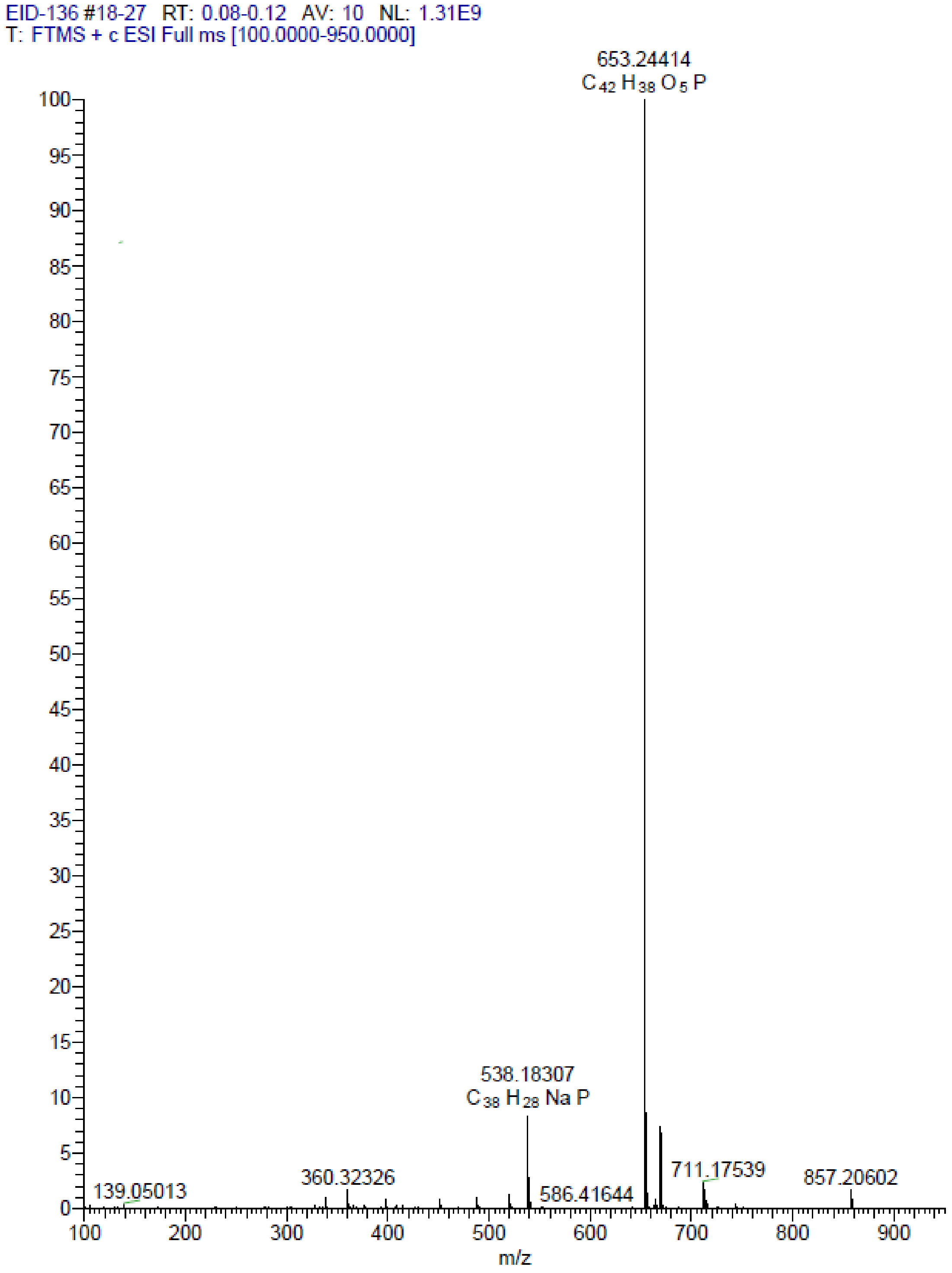

Figure S 134. HRMS spectrum of compound **6a**.

6b

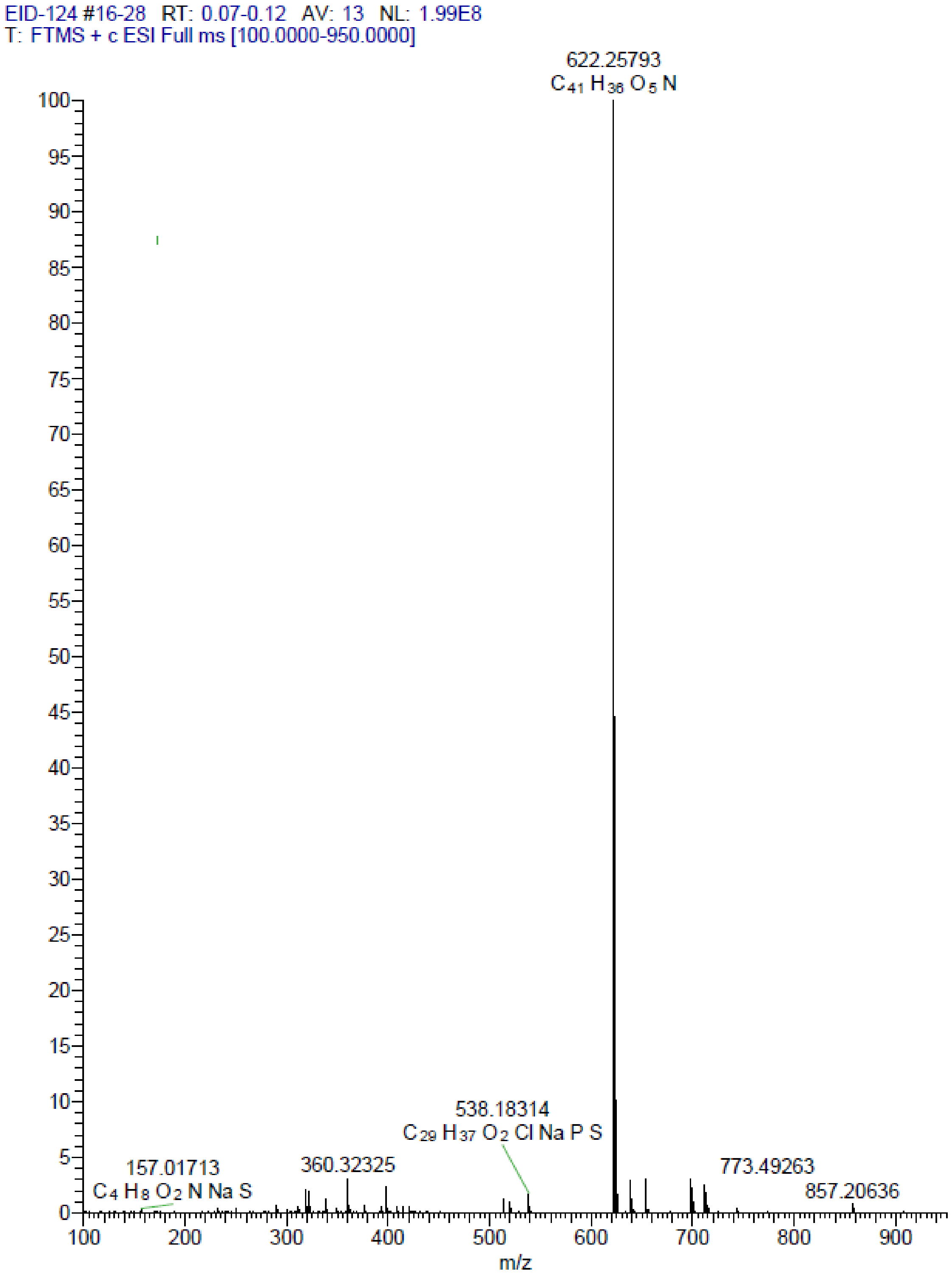

Figure S 135. HRMS spectrum of compound **6b**.

6c

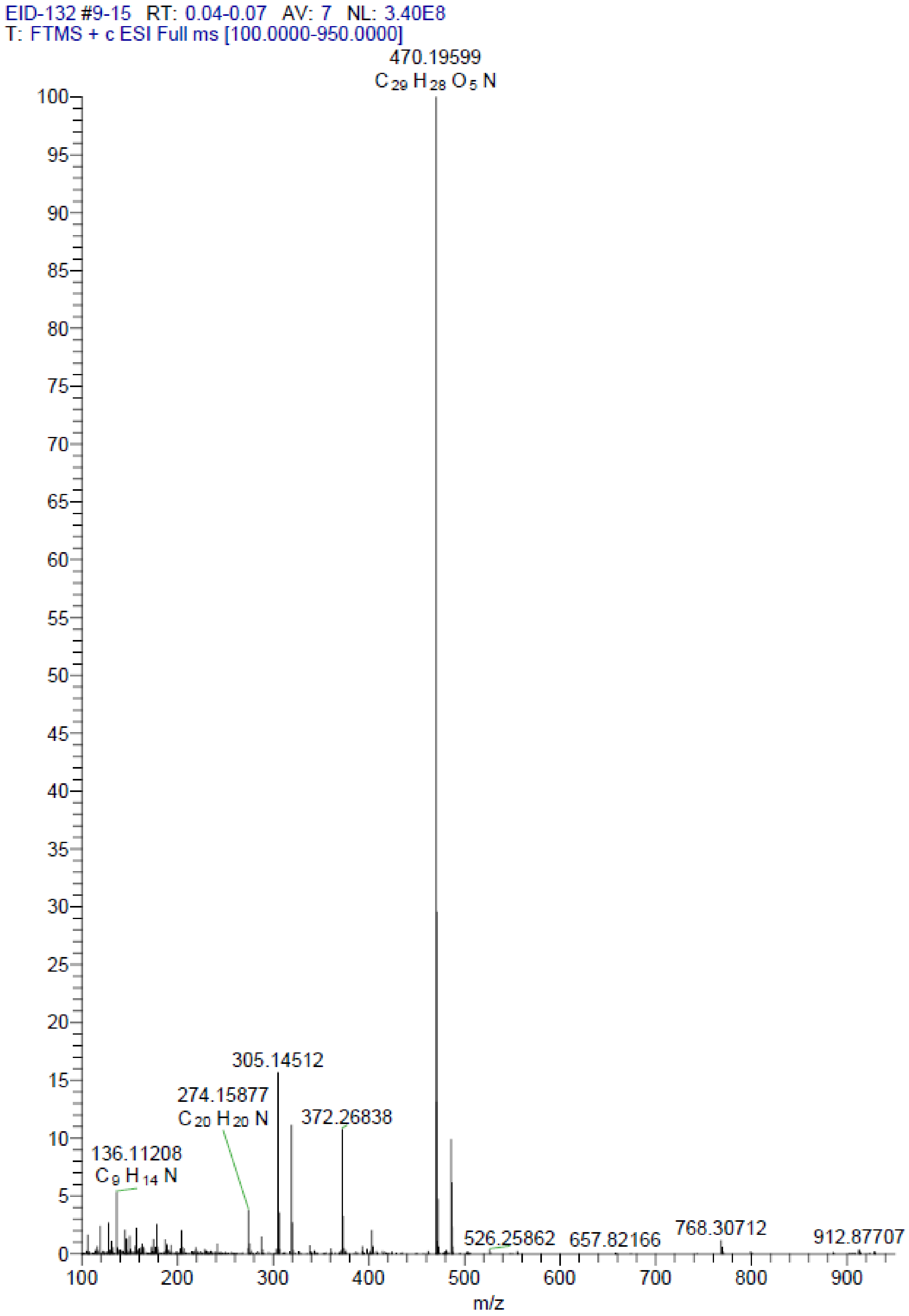

Figure S 136. HRMS spectrum of compound **6c**.

6d

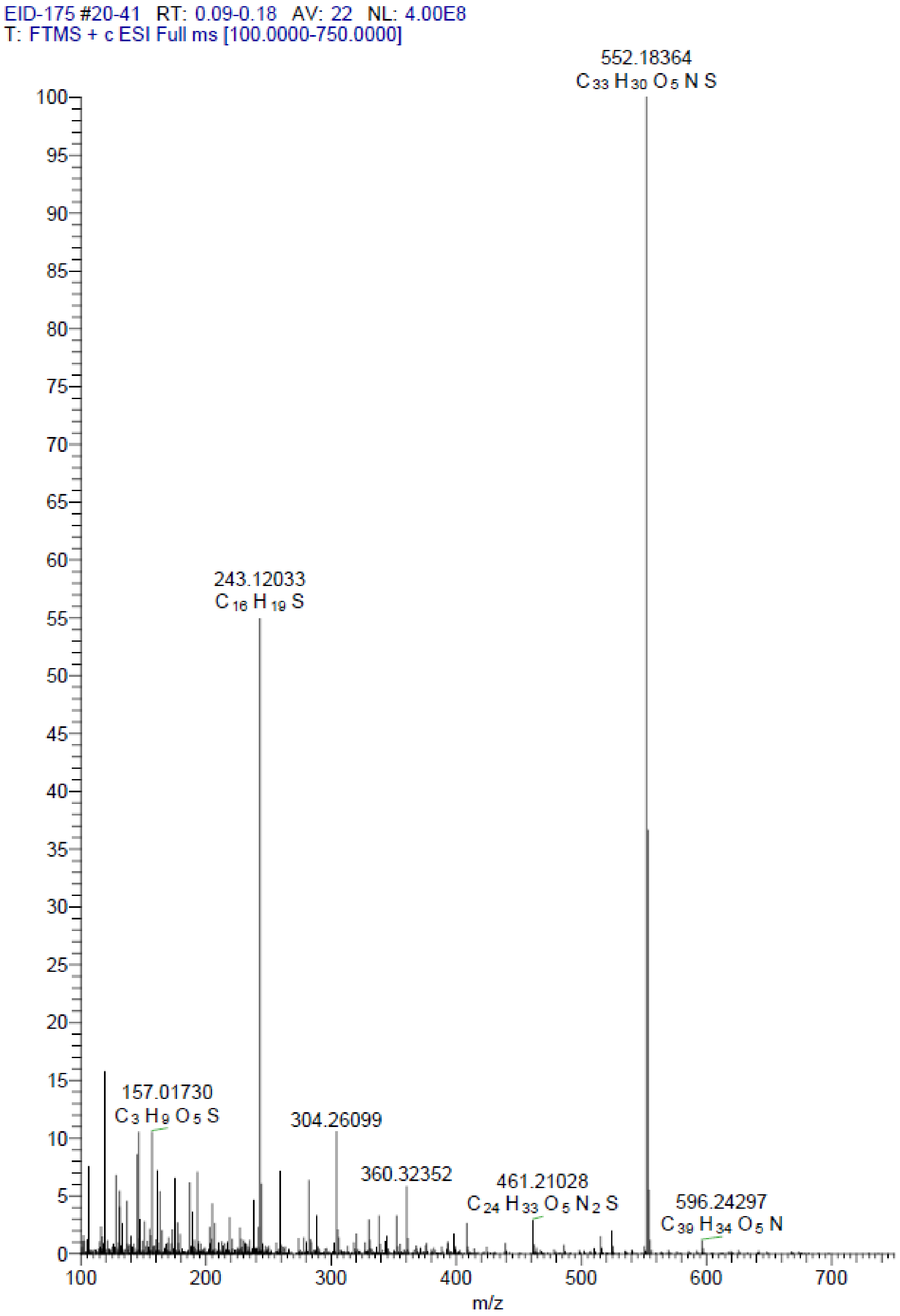

Figure S 137. HRMS spectrum of compound **6d**.

6e

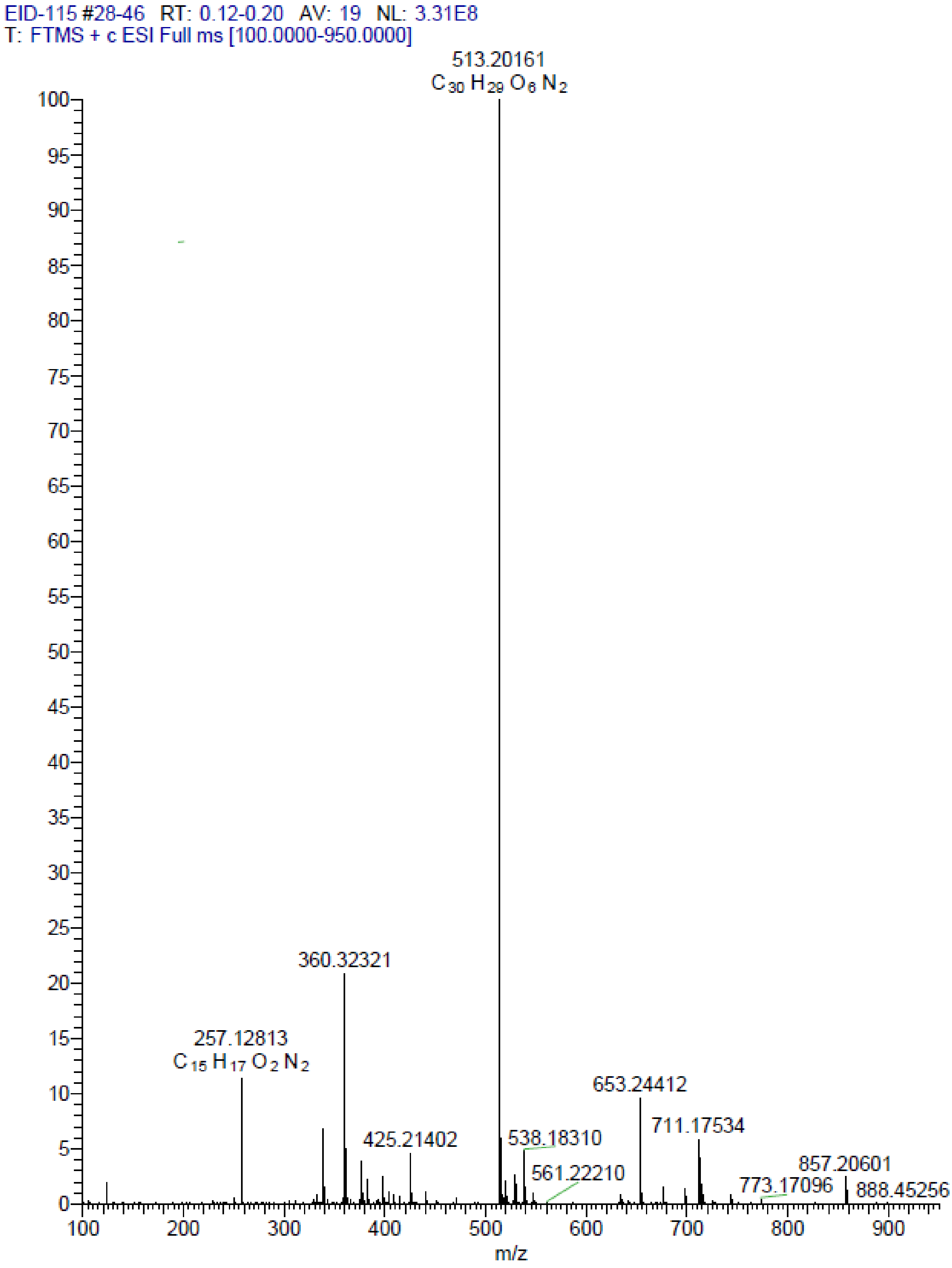

Figure S 138. HRMS spectrum of compound **6e**.

6f

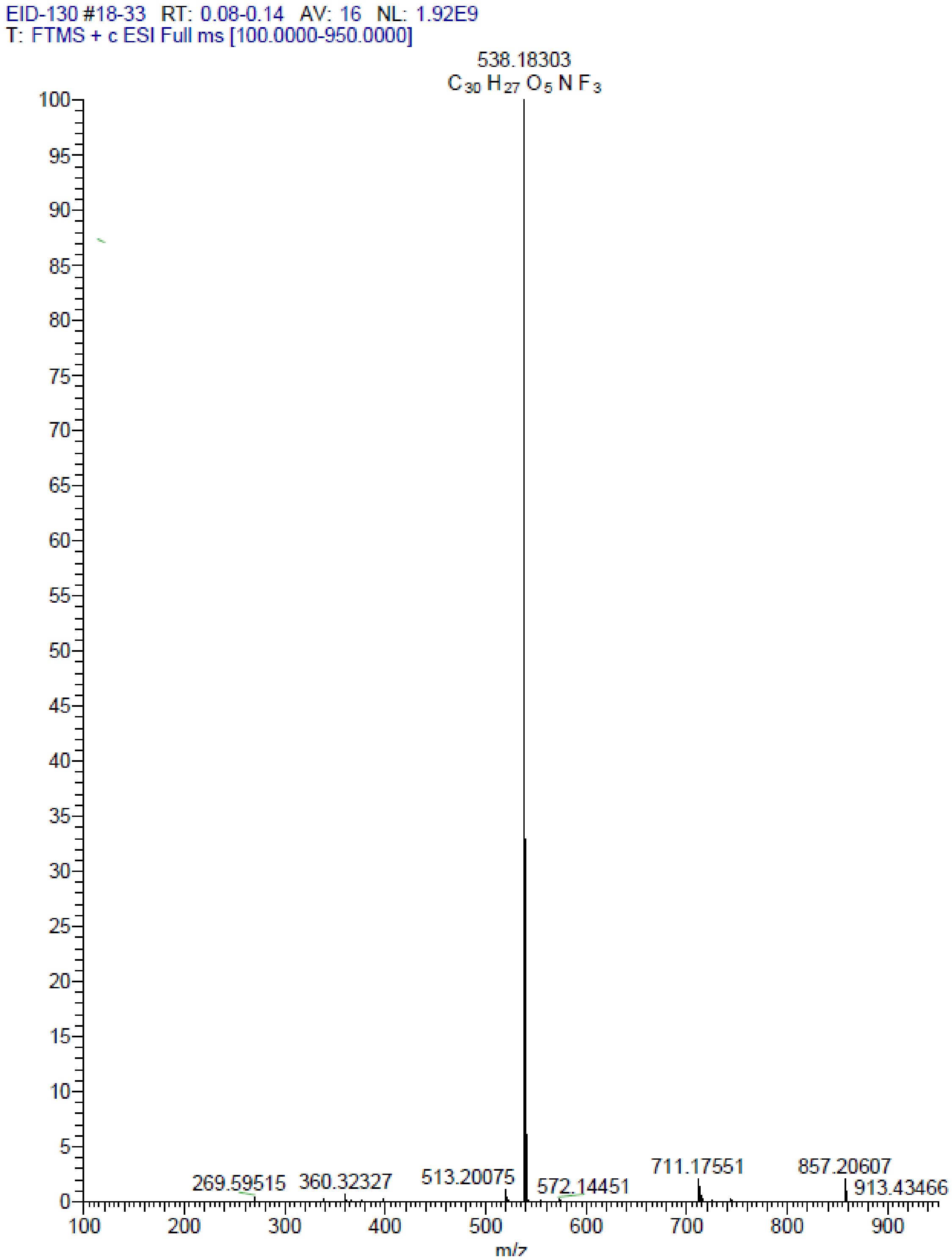

Figure S 139. HRMS spectrum of compound **6f**.

7b

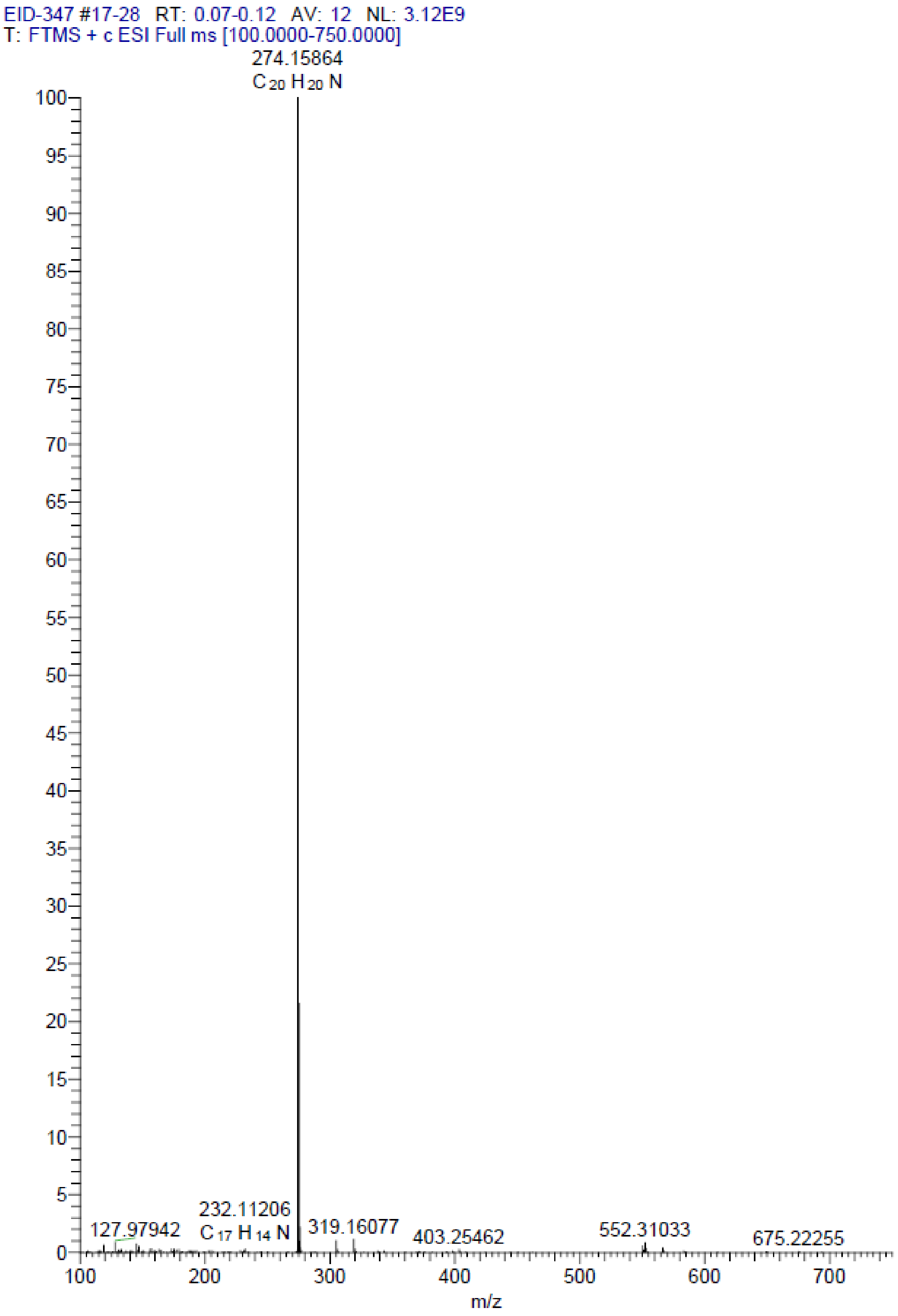

Figure S 140. HRMS spectrum of compound **7b**.

8a

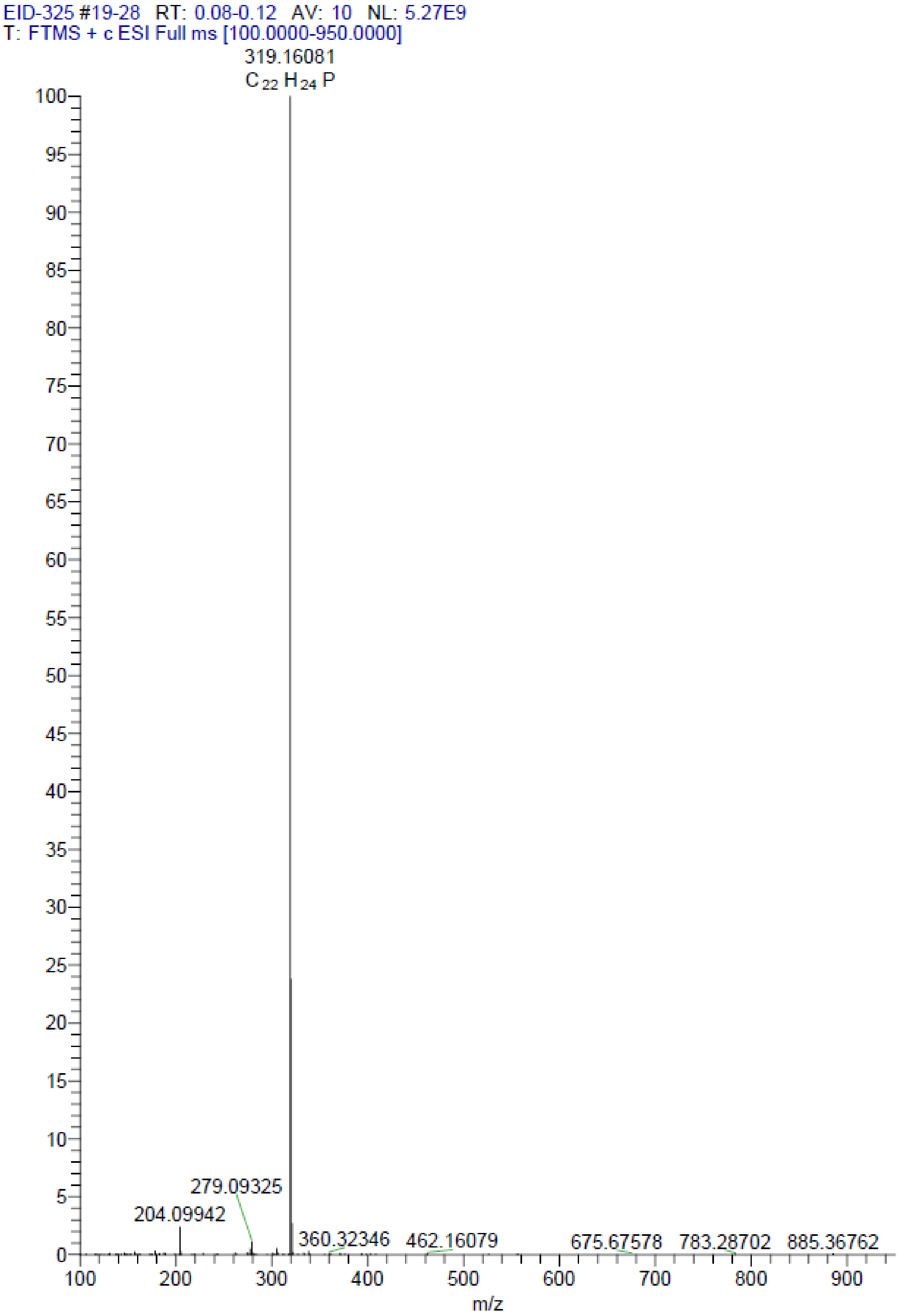

Figure S 141. HRMS spectrum of compound **8a**.

8b

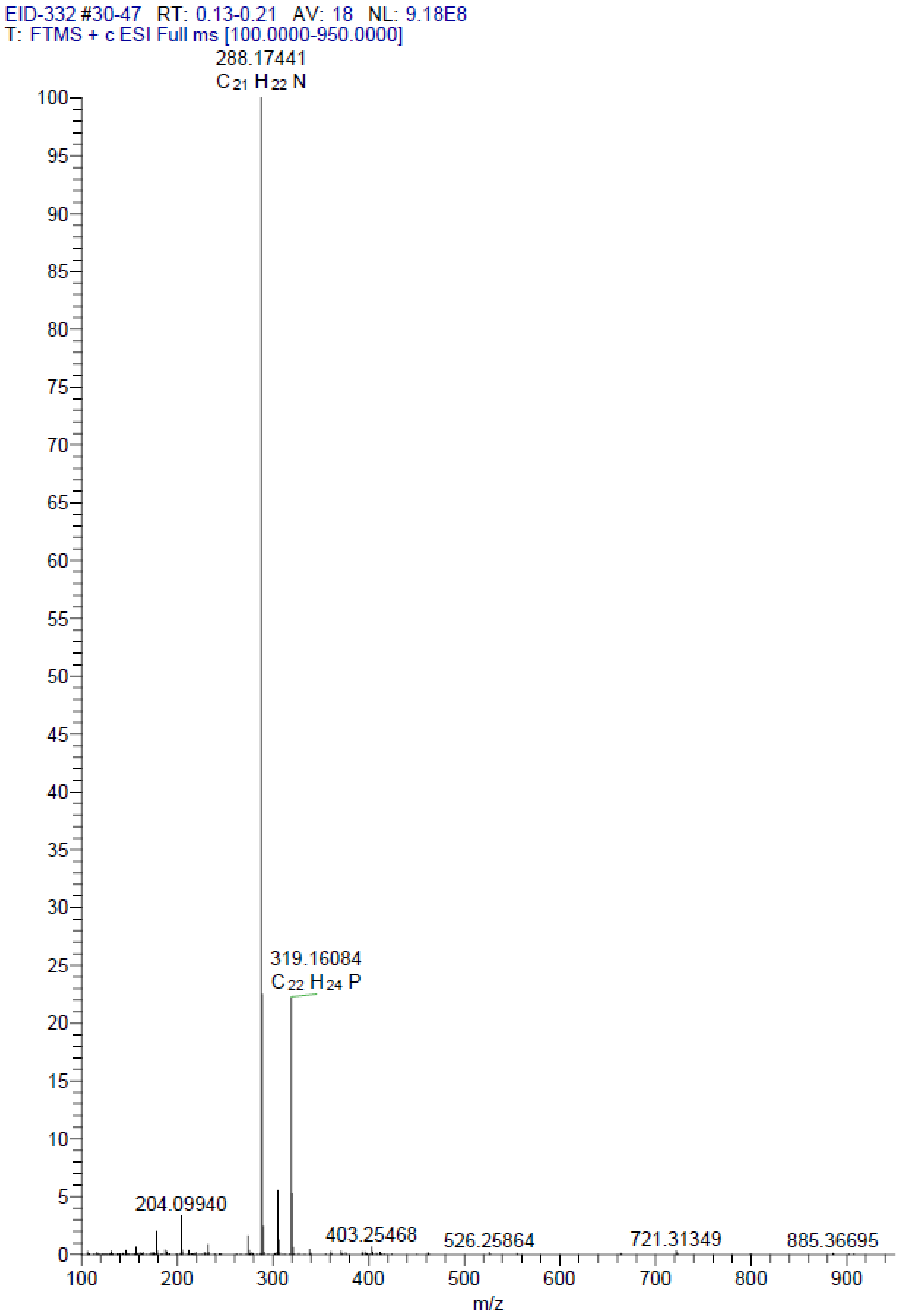

Figure S 142. HRMS spectrum of compound **8b**.

9a

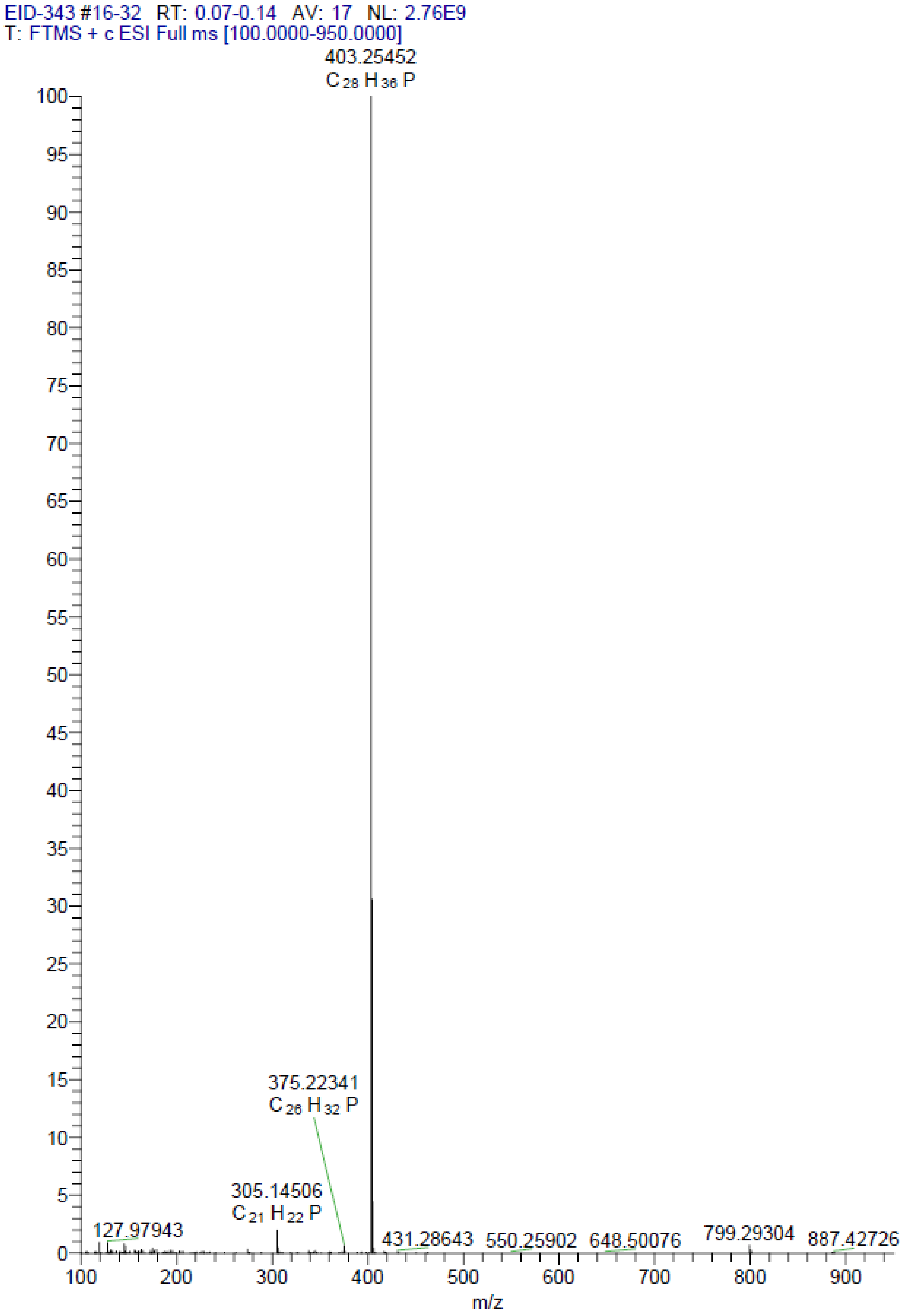

Figure S 143. HRMS spectrum of compound **9a**.

9b

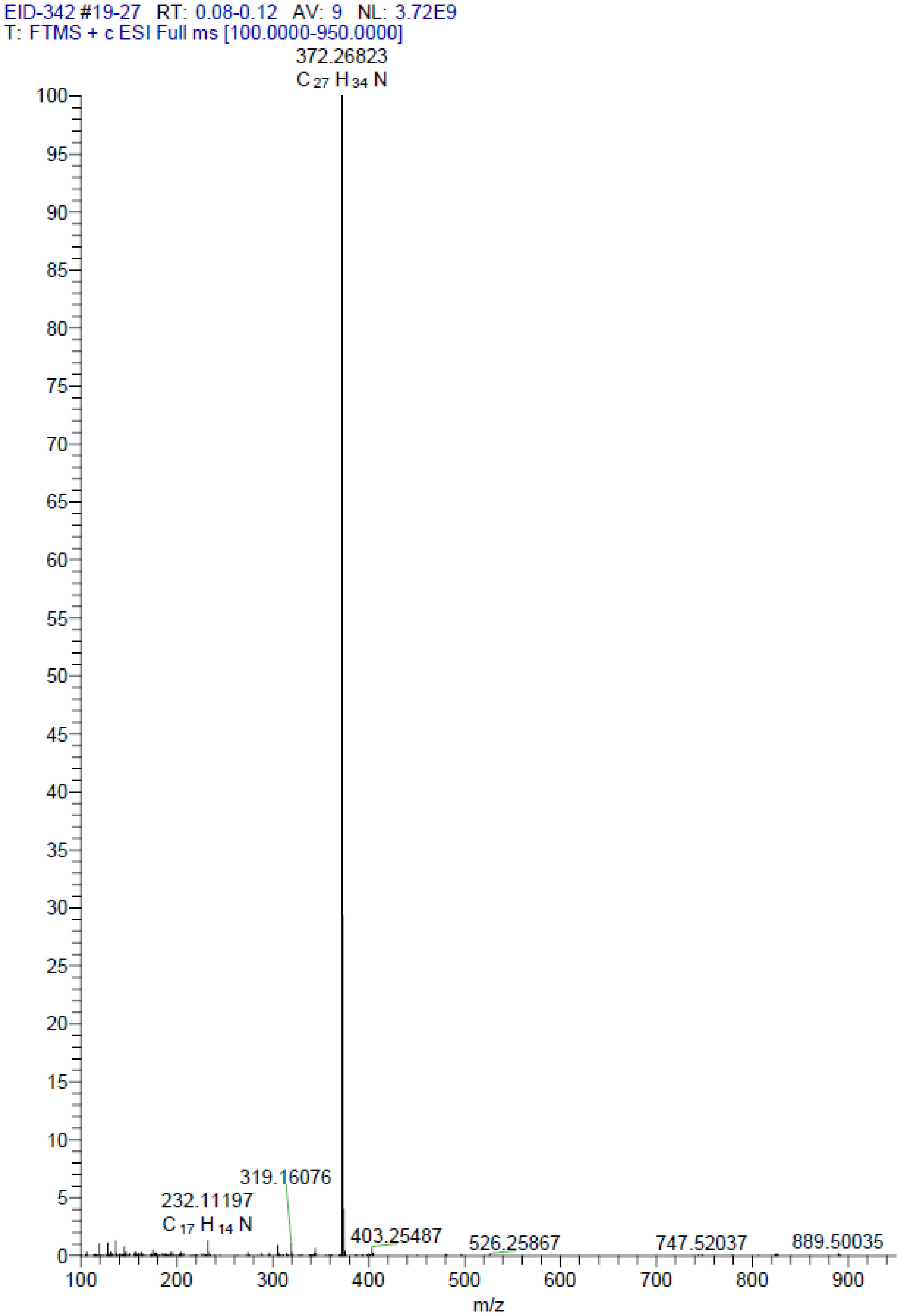

Figure S 144. HRMS spectrum of compound **9b**.

References

[1] T. Vervliet, J. Loncke, M. Sever, K. Ahuja, C. Van Den Haute, T. Luyten, G. E. Stutzmann, C. Verfaillie, T. Tomašič, G. Bultynck, “Inactive ryanodine receptors sustain lysosomal availability for autophagy by promoting ER-lysosomal contact site formation” *Nat Commun* **2026**, *17*, 1293.

[2] J. Schindelin, I. Arganda-Carreras, E. Frise, V. Kaynig, M. Longair, T. Pietzsch, S. Preibisch, C. Rueden, S. Saalfeld, B. Schmid, J.-Y. Tinevez, D. J. White, V. Hartenstein, K. Eliceiri, P. Tomancak, A. Cardona, “Fiji: an open-source platform for biological-image analysis” *Nat Methods* **2012**, *9*, 676–682.

[3] T. Yoshimori, A. Yamamoto, Y. Moriyama, M. Futai, Y. Tashiro, “Bafilomycin A1, a specific inhibitor of vacuolar-type H(+)-ATPase, inhibits acidification and protein degradation in lysosomes of cultured cells.” *Journal of Biological Chemistry* **1991**, *266*, 17707–17712.

[4] S. Ohkuma, B. Poole, “Fluorescence probe measurement of the intralysosomal pH in living cells and the perturbation of pH by various agents.” *Proceedings of the National Academy of Sciences* **1978**, *75*, 3327–3331.

[5] S. Bolte, F. P. Cordelières, “A guided tour into subcellular colocalization analysis in light microscopy” *Journal of Microscopy* **2006**, *224*, 213–232.

[6] N. Otsu, “A Threshold Selection Method from Gray-Level Histograms” *IEEE Trans. Syst., Man, Cybern.* **1979**, *9*, 62–66.

[7] J.-D. Chai, M. Head-Gordon, “Long-range corrected hybrid density functionals with damped atom–atom dispersion corrections” *Phys. Chem. Chem. Phys.* **2008**, *10*, 6615–6620.

[8] F. Weigend, R. Ahlrichs, “Balanced basis sets of split valence, triple zeta valence and quadruple zeta valence quality for H to Rn: Design and assessment of accuracy” *Phys. Chem. Chem. Phys.* **2005**, *7*, 3297–3305.

[9] T. Lu, F. Chen, “Multiwfn: A multifunctional wavefunction analyzer” *Journal of Computational Chemistry* **2012**, *33*, 580–592.

[10] J. Trnka, M. Elkalaf, M. Anděl, “Lipophilic Triphenylphosphonium Cations Inhibit Mitochondrial Electron Transport Chain and Induce Mitochondrial Proton Leak” *PLOS ONE* **2015**, *10*, e0121837.

[11] E. P. Bulthuis, C. Einer, F. Distelmaier, L. Groh, S. E. van Emst - de Vries, E. van de Westerlo, M. van de Wal, J. Wagenaars, R. J. Rodenburg, J. A. M. Smeitink, N. P. Riksen, P. H. G. M. Willems, M. J. W. Adjobo-Hermans, H. Zischka, W. J. H. Koopman, “The decylTPP mitochondria-targeting moiety lowers electron transport chain supercomplex levels in primary human skin fibroblasts” *Free Radical Biology and Medicine* **2022**, *188*, 434–446.

[12] A. Roca-Portoles, G. Rodriguez-Blanco, D. Sumpton, C. Cloix, M. Mullin, G. M. Mackay, K. O’Neill, L. Lemgruber, X. Luo, S. W. G. Tait, “Venetoclax causes metabolic reprogramming independent of BCL-2 inhibition” *Cell Death Dis* **2020**, *11*, 616.

[13] A. F. P. Alcântara, L. A. Fontana, V. H. Rigolin, Y. F. S. Andrade, M. A. Ribeiro, W. P. Barros, C. Ornelas, J. D. Megiatto Jr., “Olefin Cyclopropanation by Radical Carbene Transfer Reactions Promoted by Cobalt(II)/Porphyrinates: Active-Metal-Template Synthesis of [2]Rotaxanes” *Angewandte Chemie International Edition* **2018**, *57*, 8979–8983.

[14] S. Pivsa-Art, T. Satoh, Y. Kawamura, M. Miura, M. Nomura, “Palladium-Catalyzed Arylation of Azole Compounds with Aryl Halides in the Presence of Alkali Metal Carbonates and the Use of Copper Iodide in the Reaction” *bull. Chem. Soc. Jpn.* **1998**, *71*, 467–473.

[15] O. Rezazgui, P. Trouillas, S. Qiu, B. Siegler, J. Gierschner, S. Leroy-Lhez, “Synthesis and conformation of a novel fluorescein–Zn-porphyrin dyad and intramolecular energy transfer” *New J. Chem.* **2016**, *40*, 3843–3856.

[16] E. Deruer, S. Coulibali, S. Boukercha, S. Canesi, “Carbon–Phosphorus Bond Formation on Anilines Mediated by a Hypervalent Iodine Reagent” *J. Org. Chem.* **2017**, *82*, 11884–11890.

[17] C. Le Manach, J. Dam, J. G. Woodland, G. Kaur, L. P. Khonde, C. Brunschwig, M. Njoroge, K. J. Wicht, A. Horatscheck, T. Paquet, G. A. Boyle, L. Gibhard, D. Taylor, N. Lawrence, T. Yeo, S. Mok, R. T. Eastman, D. Dorjsuren, D. C. Talley, H. Guo, A. Simeonov, J. Reader, M. van der Watt, E. Erlank, N. Venter, J. W. Zawada, A. Aswat, L. Nardini, T. L. Coetzer, S. B. Lauterbach, B. C.

Bezuidenhout, A. Theron, D. Mancama, L. L. Koekemoer, L.-M. Birkholtz, S. Wittlin, M. Delves, S. Ottilie, E. A. Winzeler, T. W. von Geldern, D. Smith, D. A. Fidock, L. J. Street, G. S. Basarab, J. Duffy, K. Chibale, "Identification and Profiling of a Novel Diazaspiro[3.4]octane Chemical Series Active against Multiple Stages of the Human Malaria Parasite Plasmodium falciparum and Optimization Efforts" *J. Med. Chem.* **2021**, *64*, 2291–2309.

[18] J. E. T. Corrie, V. R. N. Munasinghe, W. Rettig, "Synthesis and fluorescence properties of substituted 7-aminocoumarin-3-carboxylate derivatives" *Journal of Heterocyclic Chemistry* **2000**, *37*, 1447–1455.

[19] L. Leanza, M. Romio, K. A. Becker, M. Azzolini, L. Trentin, A. Managò, E. Venturini, A. Zaccagnino, A. Mattarei, L. Carraretto, A. Urbani, S. Kadow, L. Biasutto, V. Martini, F. Severin, R. Peruzzo, V. Trimarco, J.-H. Egberts, C. Hauser, A. Visentin, G. Semenzato, H. Kalthoff, M. Zoratti, E. Gulbins, C. Paradisi, I. Szabo, "Direct Pharmacological Targeting of a Mitochondrial Ion Channel Selectively Kills Tumor Cells In Vivo" *Cancer Cell* **2017**, *31*, 516-531.e10.
